# Radiomitigators: Breakthroughs in Post-Radiation Recovery

**DOI:** 10.3390/antiox15030381

**Published:** 2026-03-18

**Authors:** Elena Obrador, José M. Estrela, Rafael López-Blanch, Paz Moreno-Murciano, Alegría Montoro, María Oriol-Caballo

**Affiliations:** 1 Scientia BioTech, 46002 Valencia, Spain; loblanch@alumni.uv.es (R.L.-B.); paz.moreno72@gmail.com (P.M.-M.); maria.oriol@uv.es (M.O.-C.); 2 Cell Pathophysiology Unit, Department of Physiology, Faculty of Medicine & Odontology, University of Valencia, 46010 Valencia, Spain; 3 IIS and Radioprotection Service, La Fe University and Polytechnic Hospital, 46026 Valencia, Spain; montoro_ale@gva.es

**Keywords:** ionizing radiation, radiomitigator and radioprotector, oxidative stress, inflammation, radiation fibrosis, tissue regeneration

## Abstract

Ionizing radiation (IR) exposure poses a significant biomedical challenge in clinical, occupational, and emergency contexts, highlighting the urgent need for effective medical countermeasures against acute radiation syndrome (ARS) and delayed effects of radiation exposure (DEARE). Depending on the timing of administration, radiation countermeasures are classified as radioprotectors, radiomitigators, or therapeutics. Among these, radiomitigators offer a critical advantage by attenuating IR-induced damage when administered after exposure, thereby expanding their applicability in unanticipated radiation incidents. This review provides an overview of the pathophysiological mechanisms underlying IR-induced injury and summarizes the current FDA-approved radiation countermeasures. It then focuses on radiomitigators that have demonstrated efficacy in preclinical animal models, together with available evidence from clinical studies, emphasizing their translational potential for both emergency preparedness and oncological settings. We examine routes of administration and key mechanisms of action, including modulation of oxidative and nitrosative stress, enhancement of DNA damage response pathways, preservation of mitochondrial function, regulation of inflammatory and immune signaling, attenuation of fibrotic remodeling, maintenance of vascular integrity, and promotion of tissue regeneration and repair. Finally, challenges associated with clinical translation and strategies to optimize radiomitigators for the management of radiation-induced injury are discussed. By integrating these insights and consolidating existing knowledge, this review aims to guide basic and clinical research toward more effective radiomitigative strategies and combination therapies to improve survival, limit tissue damage, and preserve long-term quality of life in individuals exposed to IR.

## 1. Introduction

Ionizing radiation (IR) damages cells either directly by inducing DNA strand breaks or indirectly through reactive oxygen species (ROS) generated by water radiolysis [1]. ROS can also interact with nitrogen-containing molecules to form reactive nitrogen species (RNS), and both induce oxidative/nitrosative stress on irradiated and non-irradiated cells (bystander effects) [2,3,4,5]. Together, these direct and indirect mechanisms initiate a cascade of molecular events that ultimately can result in cellular damage, repair, dysfunction, or death.

IR exposure can result in two principal categories of health effects: deterministic and stochastic. Deterministic effects—including skin erythema, alopecia, acute radiation syndrome (ARS), and long-term complications such as cataracts and cardiovascular disease—arise once a dose threshold is exceeded, with severity increasing as the dose rises [6,7]. By contrast, stochastic effects, such as carcinogenesis and heritable mutations, can occur even at low doses, with risk increasing linearly with exposure but without a defined threshold [8,9]. The biological impact of IR is influenced by multiple factors, including the type of radiation; exposure parameters such as dose, dose rate, and radiation quality; and environmental conditions such as the extent and route of exposure, for example total body irradiation (TBI) versus partial-body irradiation (PBI), or internal versus external contamination [7,9]. Host-specific variables such as genetic factors, age, sex, comorbid illnesses, tissue-specific radiosensitivity, and regenerative capacity also shape the severity and spectrum of injuries [10].

Humans are continuously exposed to background radiation from natural sources such as cosmic rays, terrestrial radionuclides, and internal isotopes, contributing to an average annual dose of 1.5–3.5 mSv [11]. Professional activities involving the handling of radioactive materials, space travel, and exposure to IR for diagnostic or therapeutic purposes (i.e., radiotherapy (RT) and chemoradiotherapy (CRT)) increase the risk of experiencing adverse radiation effects [11,12,13,14]. Despite strict radiation safety protocols, accidental IR exposures may still occur, and this risk is further heightened by the potential use of nuclear weapons, underscoring the urgent need for robust preparedness systems to monitor, prevent, respond to, mitigate, and treat the harmful consequences of IR exposure [15,16,17].

**Figure 1:**
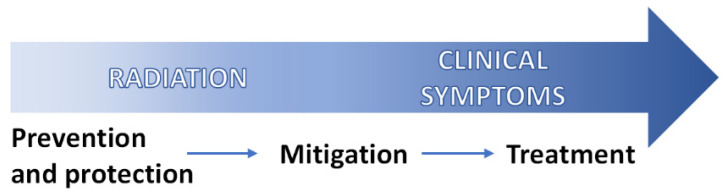
Pharmacologic modification of radiation injuries.

In the United States, the Departments of Defense and Health and Human Services use the term Radiation Medical Countermeasures (MCM) to describe agents that prevent, mitigate, treat, or aid recovery from radiation injury (Figure 1).

For research and clinical planning purposes, these radiation MCM are categorized by timing of administration into radioprotectors, radiomitigators, and therapeutics [18,19]. Because radiation-induced free radicals are highly reactive and short-lived, radioprotectors must be present at or before IR exposure to effectively prevent cellular damage [9,18,20]. Radiomitigators are administered shortly thereafter (within hours to days), but prior to symptom onset, to lessen the incidence and severity of both acute and delayed radiation injuries. Representative examples include hematopoietic growth factors such as granulocyte colony-stimulating factor (G-CSF) and granulocyte/macrophage colony-stimulating factor (GM-CSF), as well as thrombopoietin (TPO) mimetics, which are used to prevent post-irradiation neutropenia and thrombocytopenia, respectively [21,22]. Radiation therapeutic options encompass supportive care, prophylactic or replacement therapies, and palliative interventions, all of which are typically implemented after the onset of clinically significant post-radiation signs/symptoms. Supportive care includes essential measures such as fluid resuscitation, antiemetic therapy (e.g., loperamide, serotonin receptor antagonists), enteral nutrition, and pain management [23,24,25]. In this context, the primary therapeutic goal following accidental radiation exposure is to manage infectious and hemorrhagic complications while supporting hematopoietic recovery. For this purpose, empiric administration of antimicrobial agents and targeted therapies to restore blood cell counts are now considered standard of care for patients with ARS [23,24,25]. Administering growth factors that promote recovery across multiple hematopoietic lineages may help accelerate recovery and reduce ARS-related mortality [21,24,26,27]. Blood transfusions or hematopoietic stem cell transplantation (HSCT) may be used as replacement therapies, with HSCT reserved for cases in which the bone marrow (BM) recovery is unlikely despite growth factor support and in the absence of severe non-hematopoietic tissue injury [24,28,29]. Palliative care is indicated for irreversible, life-limiting radiation injury, prioritizing symptom relief and overall quality of life (QoL) [29,30].

Radioprotectors are administered prophylactically (Figure 1) in high-risk situations such as nuclear accidents, space travel, or cancer RT to protect normal tissues and potentially permit higher tumoricidal doses [31,32]. Their protective effects typically involve one or more mechanisms, including free-radical scavenging, metal chelation, antioxidant activity, enhancement of endogenous defenses, induction of transient hypoxia or chromatin compaction, cell cycle arrest, improvement of DNA repair, antiapoptotic or anti-inflammatory actions, and stabilization of cytoplasmic or mitochondrial membranes [9,31,33,34,35]. Radioprotectors such as aminothiols, superoxide dismutase (SOD) mimetics, vitamins C and E, polyphenols, and other compounds have shown modest efficacy in preclinical models, but their translation to clinical use has been limited by insufficient protection at clinically relevant doses, toxicity, and pharmacokinetic constraints [36,37,38,39]. Amifostine (formerly known as WR-2721) has been established as the standard reference in radioprotection, owing to its ability to prevent IR-induced damage in the BM and salivary glands across various animal models [38,40]. Clinical trials have validated its efficacy, particularly in reducing xerostomia in head and neck cancer (HNC) patients undergoing RT [41], leading to FDA (U.S. Food and Drug Administration) approval for this purpose. However, amifostine has significant limitations, including its inability to cross the blood–brain barrier (BBB) and thus provide central nervous system (CNS) protection, significant adverse effects (e.g., nausea, vomiting, hypotension, hypocalcemia, and cutaneous reactions), and inconsistent clinical trial results [38,40]. At present, no radioprotector has received FDA approval for use during nuclear emergencies [42].

Radiomitigators primarily act by reducing tissue damage and facilitating post-irradiation recovery through mechanisms, including modulating oxidative/nitrosative stress, enhancing DNA repair and cell survival, regulating inflammatory and immune responses, preserving vascular function, promoting tissue regeneration, and limiting the profibrotic processes that lead to maladaptive remodeling [21,22,43,44,45]. Several of these mechanisms of action, notably antioxidant activity and cytokine modulation, overlap with those of radioprotectors, reflecting that oxidative and inflammatory insults persist long after irradiation [45,46,47]. In scenarios of unexpected radiation exposure (e.g., nuclear accidents, radiological terrorism, or occupational incidents), radiomitigators are especially advantageous due to their efficacy when administered after exposure, compared with radioprotectors, which must be given in advance and may be unavailable in emergency settings [10,19,21]. Additionally, radiomitigators enable targeted intervention exclusively in exposed individuals, thereby offering a logistical and ethical advantage by avoiding unnecessary treatment of those ultimately unexposed. In oncology settings, post-RT radiomitigation aimed at preventing toxicities or DEARE should, in principle, carry a lower risk of compromising antitumor efficacy than radioprotectors [33,43,44,45].

The development of effective radiomitigators is essential not only to preserve public health in the event of radiological or nuclear emergencies—such as nuclear power plant accidents, radiological dispersal devices, or nuclear detonation scenarios—but also to protect first responders, critical infrastructure personnel, healthcare workers, and civilian populations who may be exposed to harmful levels of IR under accidental or hostile circumstances. Regulatory and public health authorities have recognized the critical need for effective MCM that reduce mortality associated with high-dose radiation exposure, given the limited efficacy of existing treatments for severe radiation syndromes [17,39,48,49]. In oncology practice, progress in this field is equally critical, as effective radiomitigators may reduce the adverse effects of RT, improve treatment adherence and prevent DEARE development [31,45].

The present review provides a comprehensive overview of recent advances in the pathophysiology of IR-induced damage to elucidate the mechanism of action of radiomitigators under investigation or already in clinical use. By integrating these insights and consolidating existing knowledge, it aims to inform basic and clinical research toward the development of more effective radiomitigative strategies and the rational design of combination therapies.

## 2. Pathophysiological Mechanisms of Radiation Damage

IR can cause direct DNA strand breaks or indirect molecular damage via ROS generated by water radiolysis, particularly hydroxyl radicals (HO^•^) [1,50,51]. Together, these mechanisms compromise cellular function and genomic integrity, underpinning the complex biological consequences of radiation exposure.

### 2.1. DNA Damage

**Figure 2:**
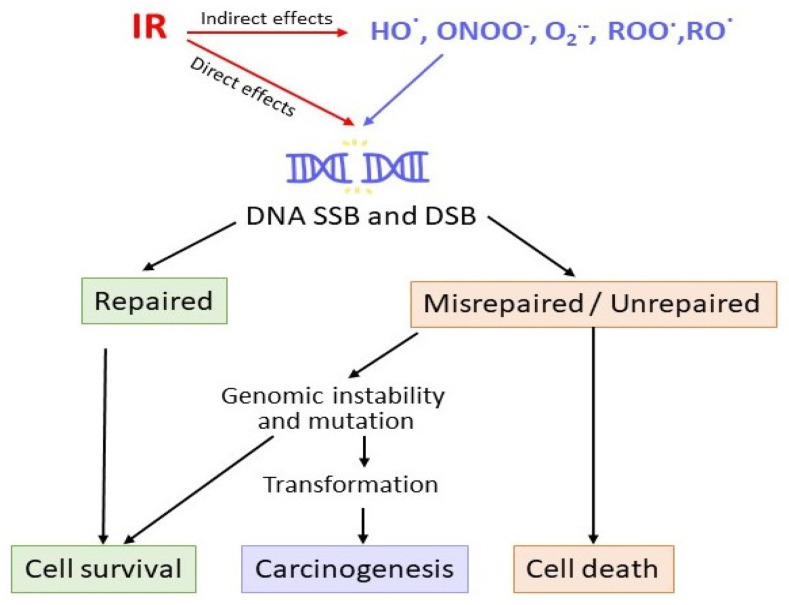
**Schematic representation of cellular outcomes following irradiation-induced DNA damage.** IR causes single-strand breaks (SSB) and double-strand breaks (DSB) both directly and indirectly (through the formation of free radical species). Depending on the efficiency and fidelity of DNA repair mechanisms, the damage may be accurately repaired, leading to cell survival, or misrepaired/unrepaired, resulting in cell death or mutations that can promote cellular transformation and carcinogenesis.

DNA is considered the primary target of IR, as demonstrated by Munro’s 1970 study, which showed that cells require a much higher radiation dose to be lethally damaged when irradiation is confined to the cytoplasm rather than to the nucleus [52]. High-energy radiation can directly ionize atoms and break chemical bonds (Figure 2), resulting in extensive structural damage to DNA, such as single- and double-strand breaks (SSBs and DSBs, respectively), base and sugar modifications, and various forms of DNA cross-linking [53,54]. Among these, DSBs are particularly lethal, as even fewer than two unrepaired DSBs can trigger apoptosis [55,56].

To preserve genomic integrity, cells rely on the DNA damage response (DDR), a coordinated network that detects lesions, transduces damage signals, and orchestrates DNA repair. DNA SSBs are primarily recognized by poly(ADP-ribose) polymerase 1 (PARP1) initiating repair through the base excision repair (BER) pathway. This process involves APE1 (endonuclease activity), DNA polymerase β (gap filling), and the XRCC1–ligase III complex (strand sealing). PARP1 activity also promotes local chromatin relaxation, facilitating access to the repair machinery. If unrepaired, SSBs can stall replication forks and ultimately convert into DSBs [54]. In contrast, DNA DSBs are detected by the MRN complex (MRE11–RAD50–NBS1), which activates ATM and triggers the DNA damage response, including H2AX phosphorylation (γ-H2AX) and recruitment of 53BP1 and BRCA1. Repair then proceeds via either non-homologous end joining (NHEJ), a fast but error-prone mechanism, or homologous recombination (HR), a high-fidelity pathway restricted to the S/G2 phases when a sister chromatid is available as a template [54,57]. Because DSBs involve disruption of both DNA strands, their accurate repair is inherently slower and more complex, and erroneous repair can lead to mutations, chromosomal aberrations, or cell death, making them more deleterious than SSBs [53,58]. The mismatch repair (MMR) system detects and repairs deletions, erroneous insertions, and base substitutions that have not been corrected by the proofreading function of DNA polymerase during DNA replication, making a major contribution to the maintenance of genome stability [5].

IR-induced DNA damage is a major trigger for the activation of DNA damage checkpoints, leading to cell cycle arrest at G1, S, and G2/M phases [56,59]. These checkpoints allow the cell to assess and repair damage before progressing through the cycle. Premature entry into the next phase without adequate checkpoint control or DNA repair can result in severe genomic instability or cell death. Consequently, checkpoint inhibitors have been investigated as a strategy to sensitize cancer cells to IR by impairing DNA repair, forcing cell cycle progression, and promoting cell death [58].

The number, complexity, and spatial clustering of IR-induced DNA lesions can overwhelm cellular repair mechanisms, resulting in the persistence of unrepaired or misrepaired damage that may progress to genomic instability, mutations, carcinogenesis, or cell death (Figure 2) [5,56]. When the extent of cell death exceeds the tissue’s regenerative capacity, it can lead to functional impairment or failure. For example, BM damage leads to pancytopenia, manifested by anemia, hemorrhage, and increased susceptibility to infection. Conversely, if the damage is sublethal but improperly repaired, surviving cells may accumulate mutations over time, thereby increasing the long-term risk of cancer [60].

### 2.2. Oxidative/Nitrosative Stress and Inflammation

Radiolysis of water causes ionization and electron ejection (H_2_O → H_2_O^+^ + e^−^), after which the ionized molecule rapidly undergoes further reactions with surrounding water to generate key reactive species, including HO^•^, H^•^ and H_2_O_2_. In oxygenated environments, secondary reactions generate additional ROS, including superoxide (O_2_^−•^) and hydroperoxyl radicals (HO_2_^•^), whose dismutation increases H_2_O_2_ levels and expands the pool of redox-active species, thereby amplifying radiation-induced oxidative damage [1,61]. Transition metals, particularly iron and copper, catalyze Fenton and Haber–Weiss reactions, accelerating the conversion of O_2_^−•^ and H_2_O_2_ into highly reactive HO^•^, thereby amplifying oxidative injury and contributing to ferroptosis through iron-dependent lipid peroxidation [62,63]. The overall amount of ROS generated from primary ionization events is further amplified via the intracellular activation of endogenous ROS-producing systems, such as the mitochondrial electron transport chain and NADPH oxidases (NOXs) [2,60,64].

DNA DSBs, ROS, and damage-associated molecular patterns (DAMPs) released from dying cells activate both ATM and toll-like receptors (TLRs) which converge to activate NF-κB signaling [65,66,67,68]. NF-κB drives the expression of more than 100 pro-inflammatory and stress-response genes, including inducible nitric oxide synthase (iNOS), pro-inflammatory cytokines (e.g., TNF-α, IL-1β, IL-6), chemokines (e.g., CXCL1, CXCL2, CXCL8), adhesion molecules (e.g., VCAM-1, ICAM-1), and enzymes involved in eicosanoid synthesis, such as lipoxygenases and cyclooxygenases (COX-1/2) [65,69,70]. iNOS-derived NO can exert protective effects such as vasodilation, but in oxidative environments it rapidly reacts with O_2_^−•^ to form peroxynitrite (ONOO^−^), which together with other RNS (NO_2_^•^ and N_2_O_3_), shifts the response toward damaging nitrosative stress that further exacerbates biomolecular damage [3,4,55]. Concurrently, COX-2, expressed at low basal levels under physiological conditions, produces large amounts of prostaglandins (particularly PGE_2_) and thromboxanes, promoting inflammation, oxidative stress, and thrombogenic responses that intensify IR-induced tissue injury [46,71,72].

IR-induced oxidative and nitrosative stress targets DNA, proteins, and lipids [1,51,73,74,75]. Highly reactive species such as HO^•^, ^1^O_2_, peroxyl radicals and ONOO^−^ (Figure 2) preferentially oxidize guanine residues in DNA and can cause additional DNA breaks, generating mutagenic lesions such as 8-hydroxy-2′-deoxyguanosine (8-OHdG) that contribute to genomic instability and carcinogenic transformation [76,77,78]. In parallel, ROS and RNS oxidize amino acid side chains, generating carbonyl groups, promoting disulfide cross-links, and cleaving peptide backbones, which leads to misfolded or inactivated proteins, including those critical for antioxidant defense, DNA repair, and mitochondrial respiration [54,60,74,79]. Protein carbonylation is considered irreversible and has been implicated in sublethal IR injury in both BM and cardiac tissue [60,74,80]. Simultaneously, HO^•^ and ONOO^−^ initiate lipid peroxidation of polyunsaturated fatty acids (PUFAs) in cellular and mitochondrial membranes [75,81]. This process generates lipid radicals and lipid hydroperoxides, which further break down into reactive aldehydes, such as 4-hydroxy-2-nonenal (4-HNE) and malondialdehyde (MDA) [73,81,82]. Primary and secondary lipid peroxidation products disrupt membrane structure, increase membrane permeability, compromise ion homeostasis, alter mitochondrial membrane potential, and impair ATP synthesis [73,83]. Both MDA and 4-HNE can form mutagenic adducts with DNA and proteins [82,84,85]. Although low concentrations of 4-HNE (below 5 μM) induce antioxidant enzyme expression and promote cell proliferation, higher levels trigger intrinsic and extrinsic apoptosis, either directly or indirectly, by forming covalent adducts with essential antioxidant molecules [60,81,85].

Due to its proximity to the electron transport chain, lack of protective histones, and limited repair capacity, mtDNA is particularly susceptible to oxidative damage [79,86]. Damage to mtDNA and mitochondrial proteins impairs the electron transport chain, causing electron leakage from complexes I and III, which drives excessive ROS generation, disrupts oxidative phosphorylation, and depletes cellular energy [87,88]. These alterations sustain the self-perpetuating “ROS-induced ROS” cycle, ultimately contributing to mitochondrial dysfunction and intrinsic apoptosis [87,88,89,90].

Under physiological conditions, cells maintain redox homeostasis via an integrated antioxidant network of non-enzymatic molecules—such as GSH (reduced glutathione), vitamins E and C, uric acid, and α-lipoic acid—and enzymatic systems that detoxify ROS and RNS [91]. SOD isoforms located in the cytosol (Cu/Zn-SOD, SOD1), mitochondria (MnSOD, SOD2), and extracellular space (Cu/Zn-SOD, SOD3) catalyze the dismutation of O_2_^−•^ into H_2_O_2_, which is subsequently decomposed into H_2_O and O_2_ by catalase (CAT), peroxiredoxins, or other peroxidases [88,92]. Upon elevation of oxidant levels, Nrf2 (the master regulator of antioxidant response) escapes proteasomal degradation, translocates to the nucleus, and binds to AREs in target gene promoters, inducing the transcription of SOD1, SOD2, CAT, GSH peroxidases (GPxs), heme oxygenase-1 (HO-1), glutathione S-transferases (GSTs), ubiquinone (coenzyme Q10), NAD(P)H:quinone oxidoreductase-1, γ-glutamylcysteine synthetase, peroxiredoxins, and other detoxifying enzymes [57,93,94]. Additionally, Nrf2 suppresses pro-inflammatory signaling pathways, including NF-κB, MAPK, NLRP3, and STAT3, thereby reducing TNF-α, IL-6, and IL-1β while promoting the anti-inflammatory cytokine IL-10 [70,95,96]. Nrf2-mediated Notch pathway activation improves hematopoietic stem and progenitor cell (HSPC) function and mitigates IR-induced myelosuppression and mortality in mice [97], highlighting the critical role of antioxidant defenses in the prevention and attenuation of IR-related damage [20,56].

IR can trigger multiple forms of cell death, including mitotic catastrophe, apoptosis, necrosis, ferroptosis, and autophagy [20,56,89,90,98,99,100]. Among these, ferroptosis appears to predominate in hematopoietic injury, likely due to iron accumulation in granulocyte–macrophage progenitors [63]. DNA damage occurring before or during mitosis can trigger mitotic catastrophe, resulting in binucleation, cell cycle arrest, and subsequent apoptosis, necrosis, or senescence [98]. Moderate oxidative stress typically induces apoptosis, a regulated process involving mitochondrial outer membrane permeabilization, cytochrome c release, and caspase activation, leading to controlled cell dismantling without inflammation [89,90,100,101]. In contrast, severe oxidative injury can deplete ATP and compromise membrane integrity, resulting in necrosis characterized by cell swelling, membrane rupture, and release of intracellular contents, which triggers inflammation in surrounding tissues [30,102]. Damaged cells upregulate autophagy to clear impaired organelles, but excessive activation can deplete essential cellular components and ultimately drive cell death [56,103,104]. Persistent sublethal oxidative stress can induce cellular senescence, a non-lethal state in which cells remain growth-arrested and adopt a senescence-associated secretory phenotype (SASP) that can drive inflammation and fibrogenesis [68,105,106,107].

The dogma that irradiation induces only a transient burst of oxidative stress has evolved. Accumulating evidence indicates that ROS and RNS generated by water radiolysis, NOXs, xanthine oxidase, NOS, and dysfunctional mitochondria sustain oxidative and nitrosative stress well beyond the initial exposure [1,64,88]. In microenvironments where protein oxidation impairs DNA repair and antioxidant defenses, a self-perpetuating cycle of inflammation and ROS/RNS production exacerbates mitochondrial dysfunction and genomic instability, ultimately leading to cell death or contributing to chronic radiation-induced tissue injury (Section 2.5) [45,51,68,108]. Collectively, these findings support the emerging concept that antioxidants and anti-inflammatory agents, traditionally considered as radioprotectors, may also act as radiomitigators in both ARS and DEARE [31,43,47,48,93,109].

### 2.3. Bystander and Abscopal Ionizing Radiation Effects

According to the 2006 report of the United Nations Scientific Committee on the Effects of Atomic Radiation (UNSCEAR), the radiation “bystander effect” (RIBE) refers to “the ability of irradiated cells to transmit manifestations of damage to neighboring cells that have not been directly irradiated”, while the “abscopal effect” is defined as “a significant response in a tissue that is physically distant from the region of the body exposed to radiation” [110].

RIBE is manifested in adjacent non-irradiated cells through a wide range of biological responses, including DNA damage (such as chromosomal aberrations, sister chromatid exchanges, and micronucleus formation), genomic instability, reduced clonogenic survival, cell cycle arrest, alterations in gene expression (e.g., p53 overexpression), changes in protein synthesis and cell proliferation, and even cell death [100,111,112,113]. Human evidence of RIBE has been demonstrated by the increased clastogenicity and micronucleus formation observed in cells exposed to sera from Chernobyl disaster survivors [114], as well as by the DNA damage detected in non-irradiated tissues of cancer patients undergoing RT [111]. Of particular significance to cancer risk, non-targeted oncogenic radiation effects have been reported in the cerebellum of radiosensitive mice when only the rest of their body was X-irradiated [115]. Furthermore, survivors of nuclear accidents and cancer patients treated with RT exhibit an increased risk of developing primary or secondary malignancies, respectively [14,116,117].

ROS, RNS, cytokines (such as IL-6, IL-8, TNF-α or TGF-β), and miRNAs released by irradiated cells are key mediators of RIBE [2,4,112,113,118,119,120]. Among these mediators, NO, whose hydrophobic nature enables intercellular diffusion, together with cytokines produced by irradiated macrophages and the activation of COX, plays a central role in triggering inflammatory responses that further heighten oxidative stress in neighboring non-irradiated tissues [3,69,118,121]. ONOO^−^ is particularly damaging due to its ability to diffuse within cells, traverse membranes via anion channels, and oxidize DNA. It also nitrates proteins, triggers lipid peroxidation, and disrupts mitochondrial function, thereby amplifying redox imbalance and propagating stress signals to neighboring non-irradiated cells [1,3,75]. Notably, RIBE can be attenuated by the administration of ROS scavengers (e.g., DMSO, GSH), SOD mimetics, NOS inhibitors (e.g., L-NAME), and COX-2 inhibitors [92,118,122].

IR can elicit immunologically active forms of tumor cell death characterized by the exposure and release of DAMPs, such as calreticulin, ATP, and HMGB1, along with enhanced presentation of tumor-associated antigens (TAAs) [123]. These signals promote dendritic cell recruitment and activation, thereby enhancing antigen cross-presentation and stimulating adaptive antitumor immune responses, thus providing a strong biological rationale for combining RT with immunotherapy [124,125]. Given that bystander signaling and inflammatory cascades may contribute to normal tissue toxicity, radioprotective strategies must be carefully tailored to avoid compromising RT or immunotherapy efficacy [113].

The term “abscopal effect” was first introduced by Mole in 1953 to describe cancer regression occurring outside the irradiated field [126]. During RT, DAMPs released as a consequence of necrosis stimulate monocytes to produce TNF-α, IL-1, IL-6, and IL-8, which in turn promote dendritic cell maturation and local inflammation, creating an immunostimulatory microenvironment that facilitates subsequent T-cell priming [127]. TAAs are then engulfed by antigen-presenting cells and presented to CD8^+^ T cells, which differentiate into cytotoxic T lymphocytes capable of migrating to tumor sites and eliminating tumor cells [128,129,130]. Collectively, these responses can promote immunogenic cell death in the primary tumor as well as in metastases, potentially leading to the abscopal effect (from ‘ab scopus’, that is, away from the target) [131,132]. Early reports of the abscopal effect, although rare (46 cases documented between 1969 and 2014 in a systematic review [133]), sparked considerable interest as well as skepticism, since locally applied RT is known to exert both local and systemic immunosuppressive effects [134,135,136,137]. More recently, abscopal responses have been confirmed in non-small cell lung carcinoma (NSCLC), kidney cancer, melanoma, lymphomas, and hepatobiliary cancers according to a recent meta-analysis [138]. Whether a high-dose single-fraction approach is superior to a moderate- or low-dose, multiple-fraction approach to enhance the abscopal effect and, in turn, avoid RIBE is still a matter of debate [110]. The abscopal effect provides a mechanistic basis for integrating immunotherapy to enhance the efficacy of RT. Preclinical and clinical studies have demonstrated the potential of harnessing the abscopal effect to elicit systemic antitumor immunity, offering a promising approach for the management of metastatic disease [139,140]. Strategies include the co-administration of RT with cytokines (e.g., IL-2 and GM-CSF) [134,141], immune-checkpoint inhibitors (e.g., anti-CTLA-4, anti-PD-1, and anti-PD-L1) [124,142,143,144], vaccines, and other immunomodulatory approaches [110,125,139]. In a proof-of-concept clinical trial, Golden et al. reported an abscopal response rate of 27% in patients with metastatic solid tumors treated with RT plus immunotherapy, accompanied by a marked prolongation of median overall survival (mOS: 21 vs. 8 months) [145]. Nevertheless, the probability of achieving a clinically meaningful abscopal response remains limited, and optimization of immunotherapy timing, sequencing, and dosing in conjunction with RT is essential to maximize therapeutic benefit, while minimizing immune-related adverse events.

### 2.4. Acute Radiation Syndrome

**Figure 3:**
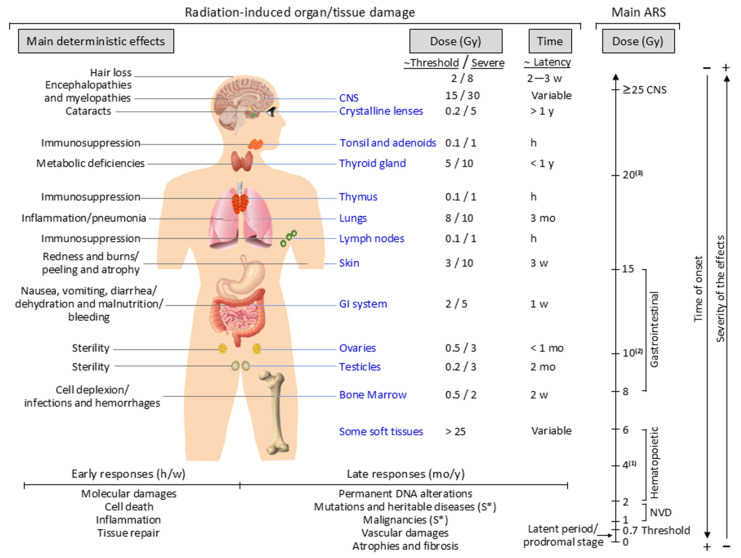
**Signs and Symptoms of ARS.** The main radiation-induced biological effects are displayed, indicating the differences between the threshold doses and those that cause a severe effect. (S*) indicates a main stochastic effect. Abbreviations: CNS, central nervous system; GI, gastrointestinal; h, hours; mo, months; w, weeks; y, years. Reproduced from Obrador et al. review [18] and used under CC BY 4.0.

ARS was first described after the atomic bombings of Hiroshima and Nagasaki and subsequently characterized through clinical analyses of nuclear accidents and criticality events [25]. The clinical expression and severity of ARS depend primarily on absorbed dose, dose rate, radiation quality (e.g., X-rays, gamma rays, protons, or neutrons), exposure geometry (e.g., TBI, PBI, or shielding), patient-related factors (e.g., sex, age, combined injuries, and comorbidities), and the time elapsed since exposure [146]. Although some abnormalities can be detected in blood cells after TBI at doses exceeding 0.7 Gy (Figure 3), ARS typically becomes clinically apparent only at doses ≥ 2 Gy [6]. The syndrome progresses through three phases: prodromal (0–2 days), latent (2–20 days), and manifest illness (21–60 days post-exposure) [25,28]. The prodromal phase is characterized by nonspecific symptoms such as fatigue, nausea or vomiting, diarrhea, and erythema [6]. The subsequent latency phase is clinically silent, with a duration inversely related to the absorbed dose and sometimes absent in cases of severe exposure [25]. The final manifest illness phase, often referred to as the “critical phase” is marked by overt and potentially dramatic clinical deterioration.

Based on the dose received (Figure 3), three overlapping ARSs are recognized: the hematopoietic acute radiation syndrome (H-ARS, 2–5 Gy), gastrointestinal acute radiation syndrome (GI-ARS, 6–8 Gy), and neurovascular acute radiation syndrome (NV-ARS, >8 Gy) [18,28]. For acute, largely uniform TBI exposures to low LET photons (e.g., gamma or X rays), the estimated human LD_50/60_ is approximately 3.5–4 Gy in the absence of supportive care. With supportive management, including antibiotics, transfusion support, and intensive care, the LD_50/60_ increases to approximately 4.5–7 Gy. Under optimal conditions with rapid access to intensive care units and HSCT, survivability may extend to dose ranges approaching 7–9 Gy, although outcomes at these levels are frequently limited by non-hematopoietic organ failure [28].

#### 2.4.1. Hematopoietic Acute Radiation Syndrome

The highly proliferative hematopoietic system is a primary target of radiation injury, and the capacity to compensate for hematological failure determines ARS prognosis [6,24]. At doses of 0.7 Gy and above, the onset of cytopenia depends on the radiosensitivity and lifespan of each hematopoietic cell line. Interestingly, although lymphocytes are mature cells, they are extremely radiosensitive, and a decline in peripheral lymphocyte counts typically occurs within the first 6 to 48 h after IR exposure [25,147]. This rapid depletion is, in fact, the most widely used laboratory marker for estimating radiation dose during the early phase following accidental exposure [148]. Reductions in circulating neutrophil and platelet counts, which also correlate with absorbed IR dose, typically appear within 1–2 days and 5–10 days post-exposure, respectively [24]. Clinical manifestations of H-ARS include fatigue, fever, impaired wound healing, hemorrhages, opportunistic infections, and, in severe cases, septicemia [22,23,149]. Granulocyte recovery typically occurs within 1–3 months, whereas lymphopenia may persist longer due to injury to lymphoid organs and lymphopoietic progenitors [150]. Lymphopenia has been correlated with increased risk of carcinogenesis after IR exposure [151].

#### 2.4.2. Gastrointestinal Acute Radiation Syndrome

GI-ARS develops following radiation-induced destruction of intestinal crypt stem cells, leading to impaired epithelial regeneration, mucosal barrier disruption, and severe gastrointestinal (GI) toxicity [34,152]. Clinical manifestations include nausea, vomiting, abdominal pain, and diarrhea (often bloody), frequently resulting in dehydration and electrolyte imbalance [153]. Due to immunosuppression, sepsis caused by translocation of enteric bacteria is a major cause of lethality, even at doses < 6 Gy [34].

Radiation also affects the oral, pharyngeal, and esophageal mucosa, resulting in dysgeusia (taste alterations), oral mucositis (OM), xerostomia, dysphagia, and esophagitis [154,155,156]. OM is a frequent adverse effect of RT, occurring in approximately 80–95% of patients with HNC treated with conventional fractionated schedules (60–70 Gy in 1.8–2 Gy fractions) and in those undergoing myeloablative HSCT conditioning regimens that include TBI (≈10–14 Gy) combined with high-dose chemotherapy (CT) [156,157]. In radiation-induced oral mucositis (RIOM), symptoms typically emerge during the second to third week of conventionally fractionated RT (after cumulative doses of approximately 20–30 Gy), peak toward weeks 4–5 as cumulative dose approaches 50–60 Gy, and generally resolve within 2–4 weeks after treatment completion, although healing may be delayed in patients receiving concurrent CT [156]. The evolution of RIOM follows four stages: initiation (ROS release and DNA damage in epithelial, vascular, and mesenchymal cells), amplification (NF-κB and ceramide pathway activation, release of TNF-α, IL-1β, IL-6, increased epithelial permeability, and basement membrane damage), ulceration (loss of mucosal barrier, secondary infections, severe inflammation), and healing (often with fibrosis) 6–8 weeks after the end of RT [156,158]. Xerostomia, caused by apoptosis of salivary gland acinar cells, aggravates RIOM by reducing saliva’s protective and antimicrobial functions [159,160,161]. Dysgeusia and severe oral pain impair oral intake, contributing to malnutrition which, together with reduced QoL and increased infection risk, may compromise RT/CRT tolerance and treatment completion, ultimately impacting prognosis and survival. Dysphagia remains one of the most critical CRT-related toxicities in head-and-neck cancer (HNC) patients, potentially leading to life-threatening complications such as aspiration and pneumonia [155,162,163].

#### 2.4.3. Neurovascular Acute Radiation Syndrome

Compared to H-ARS and GI-ARS, research on strategies to mitigate NV-ARS (cerebrovascular syndrome) remains limited, as it is considered almost invariably lethal in mass-casualty scenarios [150]. NV-ARS is characterized by an acute prodrome of nausea and vomiting, followed by neurological deterioration including papilledema, loss of deep tendon and corneal reflexes, headache, hypotension, fatigue, dizziness, disorientation, cognitive dysfunction, and lethargy, which may progress rapidly to coma and death [28,164]. Radiation-induced injury to endothelial and glial cells results in microvascular dysfunction, white matter demyelination, and chronic inflammation, which further aggravates ischemia and ultimately leads to delayed radiation necrosis (RN) [165]. Imaging studies (computed tomography and MRI) demonstrate CNS changes consistent with acute vascular injury: increased capillary permeability, BBB disruption, meningeal inflammation, cerebral edema, and microhemorrhages [164,166]. Even when microhemorrhages and thrombosis are not present, cerebral edema aggravates ischemia, creating a vicious cycle that ultimately leads to IR-induced brain necrosis, a condition often considered irreversible and difficult to treat [167]. Irradiation of the brain often leads to loss of neurogenesis, demyelination, depletion of oligodendrocyte progenitors, and atrophy of both white and gray matter, resulting in deficits in memory, attention, executive function, and motor and language skills [46,164,168].

#### 2.4.4. Cutaneous Acute Radiation Syndrome

The term cutaneous acute radiation syndrome (C-ARS) was introduced to describe the deterministic skin injuries resulting from acute IR exposure (≥3 Gy), typically including erythema, blistering, hair loss (epilation), moist desquamation, dermal necrosis, and ulceration [25,169,170]. C-ARS can appear within hours to days post-exposure (e.g., transient erythema within 1–2 days) and can be particularly severe when involving a large body surface area or in cases where necrotic ulceration develops [34,170]. For example, during the Chernobyl disaster, among 134 individuals with confirmed high-dose radiation exposure, 54 developed varying degrees of C-ARS, which was the primary cause of death in 16 of the 28 fatalities [171]. Radiation-induced dermatitis (RID) affects up to 90% of cancer patients undergoing RT, often manifesting as erythema, pain, dryness, pruritus, and desquamation, and in severe cases, ulcerations or infections that may necessitate treatment interruption, potentially compromising patient prognosis [172,173,174]. RID is particularly frequent and severe in the neck area due to thin skin, a dense microvascular network, and constant friction, which together heighten vulnerability to radiation damage [175]. Radiation induces cellular senescence and impairs the mitotic capacity of stem progenitor cells located in the basal layers of the epidermis, thereby disrupting epidermal and hair follicle renewal [174,176]. Additionally, radiation can disrupt skin microcirculation, leading to vascular permeability and vasoconstriction, resulting in skin tissue ischemia and hypoxia [177]. The upregulation of inflammatory mediators promotes the trans-endothelial migration of immune cells that can further exacerbate inflammation and worsen tissue damage [69,174,176]. Current standard skin care for patients undergoing RT includes gentle daily cleansing of the treated area and the use of hypoallergenic emollient moisturizers [178]. Although topical corticosteroids are commonly prescribed for the management of RID, their prophylactic use remains controversial due to potential adverse effects, including secondary infections, which are more likely to occur in immunosuppressed patients [179]. Administering systemic steroids for radiation burns, ulcers, or necrosis is strongly contraindicated [180]. If necrosis develops, the addition of antibiotic therapy may reduce the need for surgical intervention [23,174].

#### 2.4.5. Additional Tissues Involved in ARS

Although the lungs are considered highly radiosensitive, radiation-induced pulmonary injury (RILI) is not classified among the ARS subsyndromes, likely because it typically appears 4–12 weeks after IR exposure [181,182]. RILI is estimated to occur in over 50% of radiation accident victims, often in the context of multi-organ failure [181,183]. The risk of conventional CRT-induced pneumonitis correlates strongly with mean lung dose and the percentage of total lung volume receiving ≥20 Gy (V20). Meta-analyses indicate that a V20 > 40% is associated with ~35% symptomatic pneumonitis and >3% fatal pulmonary toxicity [184], although these rates have declined with modern techniques such as IMRT (intensity-modulated radiotherapy), SBRT (stereotactic body radiotherapy), and proton therapy. TBI doses sufficient to cause RILI would first result in hematopoietic or GI lethality, necessitating the use of PBI restricted to the thoracic region (10–15 Gy) to study RILI or radiation-induced heart disease (RIHD) and to evaluate potential radiomitigators [185]. Acute radiation-induced toxicity appears to primarily involve endothelial cells and alveolar epithelial type I (ATI) and type II (ATII) cells that show phenotypes of SASP [186,187]. DNA damage and SASP lead to macrophage recruitment and polarization toward the M1 phenotype, which increases the production of pro-inflammatory cytokines such as IL-1β, TNF-α, and IL-6 [188,189,190]. These pro-inflammatory conditions impair surfactant production, disrupt alveolar barrier integrity, and increase protein exudation into the alveolar space, producing interstitial edema and thinning of the alveolar septa (hallmarks of pneumonitis) [187]. Dyspnea or increased breathing frequency (tachypnea), fever, chest pain, and dry cough are initial symptoms that may progress to respiratory failure in severe cases [146,191]. Pneumonitis, considered the initial symptomatic phase of RILI, is typically treated with antibiotics, corticosteroids, oxygen therapy, or airway interventions [192,193]. Persistent inflammation can lead to an overlap between pneumonitis and the onset of radiation-induced lung fibrosis (RILF), which typically develops months to years later [189,191].

However, these “sub-syndromes” tend to oversimplify the clinical reality of ARS, as in most accidental exposures, IR-induced damage is further aggravated by trauma, burns, and hemorrhages. Such combined insults, collectively termed radiation combined injury (RCI), significantly delay wound healing, often lead to bacteremia, precipitate multiple organ dysfunction, and increase mortality [70,164,194,195]. These findings underscore the need to develop MCM that address systemic radiation injury as a whole, rather than focusing solely on organ-specific late tissue effects [164,196].

### 2.5. Delayed Effects of Acute Radiation Exposure

Survivors of ARS, as well as professionals and patients exposed to IR, are at risk of developing the delayed effects of acute radiation exposure (DEARE), a heterogeneous group of late-onset, multi-organ disorders that may manifest months to years after IR exposure [116,197,198,199,200,201,202,203,204]. The pathogenesis of DEARE involves a multifactorial interplay of persistent DNA damage, mutagenesis, loss of stem cell self-renewal potential, chronic oxidative stress, inflammation and fibrosis [45,197].

Carcinogenesis is regarded as a stochastic IR effect, with risk increasing with dose and no threshold of safety. Leukemia was the first cancer type observed in excess among survivors of the atomic bombings of Hiroshima and Nagasaki [199]. With longer follow-up, excess risks of various solid cancers, including lung, liver, colon, breast, stomach, esophagus, bladder, and ovary, have also been documented [116,199,200,201,205]. Given the long latency of cancer development and the high proliferative rate of tissues during early life, cancer risk is substantially greater when IR exposure occurs during pregnancy, childhood, or adolescence [13,204,205]. In utero, IR can be mutagenic, teratogenic or carcinogenic, depending on the level of exposure and stage of fetal development [10,206]. During blastogenesis, radiation doses above 0.1 Gy can cause implantation failure, whereas exposure during early fetal development most commonly results in growth restriction, microcephaly, and neurodevelopmental impairment [207,208]. Low birth weight (often a consequence of intrauterine growth restriction) has been associated with increased rates of prematurity and related complications [208].

Hematopoietic DEARE manifests as residual BM damage, characterized by depressed hematopoiesis due to hematopoietic stem cell (HSC) myeloid skewing, defective lymphocyte reconstitution, and immune insufficiencies [197,209]. Continuously diminished reserve and self-renewal capacity of BM HSCs, leading to subsequent immunosuppressive states, may account for epidemiological findings of increased cancer risk in ARS survivors, workers exposed to radioactive materials, and patients undergoing diagnostic imaging, as well as a heightened likelihood of developing secondary malignancies following RT or CRT [14,199,201,204,210].

Delayed radiation-induced brain injury develops within 4–6 months after irradiation and may progress over subsequent years [46]. Neural progenitor cells in the subventricular zone and the subgranular zone of the hippocampus are particularly radiosensitive, and radiation-induced suppression of neurogenesis is consistently associated with learning and memory deficits in both preclinical and clinical studies [211,212,213]. This damage is further compounded by impaired perfusion due to a significant reduction in hippocampal capillary density following irradiation [214]. In parallel, microvascular injury, glial activation, chronic neuroinflammation, and oligodendrocyte dysfunction lead to demyelination and impaired axonal conduction, which further exacerbate progressive neuronal dysfunction and cognitive impairment [215,216,217,218]. Leukoencephalopathy, characterized by demyelination, axonal degeneration, vascular injury and cortical atrophy, is substantially more frequent after conventional whole-brain or fractionated RT than after focal stereotactic approaches (~50% vs. 5%, at 2 years) [168,219]. Advances in RT techniques such as intensity-modulated RT, volumetric modulated arc therapy, and proton beam therapy, have enabled more precise dose sparing of critical brain regions [164,220]. In parallel, a range of pharmacological interventions—including inflammatory blockade, glucocorticoids, antiangiogenics, and neural stem cell replacement—have been evaluated in preclinical models for their potential to prevent or mitigate radiation-induced cognitive impairment [221,222,223,224,225,226,227], but few have reached clinical trials, with limited efficacy so far [167,228,229].

**Figure 4:**
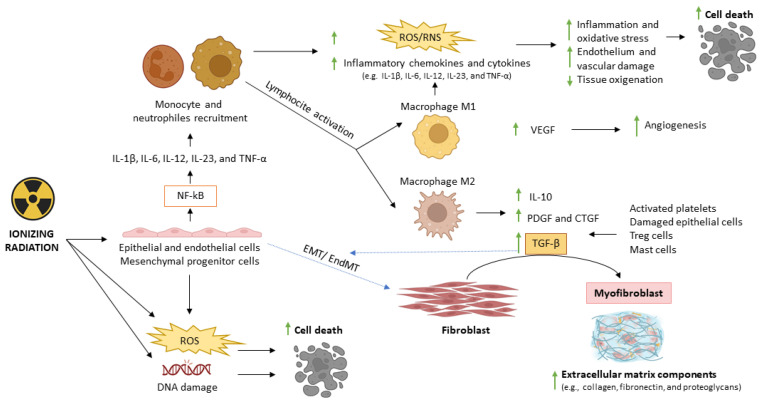
**Radiation-induced Fibrosis Pathophysiology.** Directly or indirectly, IR causes damage to nearly all tissues, but highly proliferative and endothelial cells are particularly vulnerable. Endothelial damage promotes plasma fibrinogen extravasation and fibrin deposition, forming a provisional matrix that supports immune cell infiltration. In the early phase, M1 macrophages release IL-1β, IL-6, and TNF-α, which promote inflammation and VEGF-mediated revascularization. In the subsequent reparative phase, M2 macrophages secrete TGF-β and other growth factors that drive fibroblast-to-myofibroblast transition, collagen synthesis, ECM deposition, and tissue remodeling. After IR exposure, chronic inflammation perpetuates oxidative stress generating a positive feedback loop that favors TGF-β overproduction culminating in RIF. Created in BioRender. López, R. (2026) https://BioRender.com/95jf9e7.

As previously noted, after IR exposure, DAMPs initiate a pro-inflammatory cascade by recruiting neutrophils and macrophages, which initially adopt N1 and M1 phenotypes, respectively (Figure 4) [47,230]. M1 macrophages, driven by IRF5 and NF-κB (p50–p65) activation, release TNF-α, IL-6 and IL-12, amplifying immune cell infiltration and inflammation and transiently exacerbating radiation injury by increasing oxidative/nitrosative stress and vascular damage [46,47,230,231,232,233]. As the response progresses, damaged cells are cleared and immune populations transition toward anti-inflammatory N2/M2 phenotypes, which secrete IL-10 and pro-repair growth factors such as vascular endothelial growth factor (VEGF), platelet-derived growth factor (PDGF), connective tissue growth factor (CTGF), and TGF-β1 [47,230,234,235,236,237]. IL-10 dampens pro-inflammatory signaling, VEGF and CTGF promote angiogenesis and vascular repair, and PDGF together with TGF-β1 supports fibroblast recruitment, differentiation into myofibroblasts, and extracellular matrix (ECM) synthesis, contributing to tissue remodeling and repair [232,234,235,236,238,239,240,241,242].

However, persistent oxidative/nitrosative stress, clinical or subclinical inflammation, microvascular damage, and hypoxia redirect TGF-β1-mediated repair toward maladaptive remodeling, culminating in radiation-induced fibrosis (RIF), one of the most frequent clinical manifestations of DEARE [45,191,234,235,243]. Within this context, TGF-β1 operates as the central profibrotic mediator through both SMAD2/3-dependent and non-SMAD signaling pathways [191,192,234,244,245]. Through canonical SMAD2/3 signaling, TGF-β1 upregulates NOX4-derived ROS production, promotes fibroblast recruitment and differentiation into myofibroblasts, enhances the synthesis of major ECM components such as collagen I/III and fibronectin, and suppresses ECM degradation by downregulating matrix metalloproteinases (notably MMP-2 and MMP-9), thereby accelerating matrix accumulation and fibrogenesis (Figure 4) [45,243,244,246,247,248]. TGF-β1 (Figure 4) promotes epithelial-to-mesenchymal and endothelial-to-mesenchymal transitions (EMT/EndoMT), expanding the pool of ECM-producing myofibroblasts derived from epithelial and endothelial cells [249], and in turn, ECM stiffness and myofibroblast contraction promote latent TGF-β1 activation [250]. In parallel, non-SMAD pathways—including NF-κB, MAPK (ERK, JNK, p38), PI3K/AKT, and Rho/ROCK—augment fibroblast proliferation and survival, increase ECM production, promote cytoskeletal remodeling and contractility, and sustain TGF-β1 production [245]. Other interconnected signals/mechanisms contributing to RIF perpetuation include sustained ROS production [45,246], lipid peroxidation products [81], HIF-α activation [186,246], TNF-α and IL-6 [188,233], sustained M2 polarization [251], PDGF and/or CTGF upregulation [239,241,252,253], SASP signaling [250,254], p53-dependent pathways [255], epigenetic alterations (including increased expression of miR-15b, miR-21, miR-34, and others) [256,257,258], and activation of the renin–angiotensin system (RAS) [259,260,261]. Ultimately, RIF results from a vicious cycle of inflammation, fibroblast activation, and ECM deposition, leading to progressive tissue stiffening, hypoxia, vascular rarefaction, loss of cellular function, and eventual organ atrophy or necrosis [45,47,191,243]. Understanding these interconnected molecular and cellular pathways, and their impact on tissues exposed to IR, is crucial for developing effective strategies to prevent or mitigate RIF, that typically manifests 4–12 months after IR exposure, tends to worsen over time, and can affect different organs and tissues [45,191].

It is estimated that 50% of patients subjected to abdominal or pelvic RT suffer from some degree of chronic intestinal dysfunction [262]. Late radiation enteropathy is characterized by mucosal atrophy, vascular sclerosis, and progressive intestinal wall fibrosis. Clinically, it presents with malabsorption, altered transit and dysmotility, which can progress to obstruction, fistula formation, and perforation [262,263,264]. Radiation-induced intestinal fibrosis is driven by multiple mechanisms, i.e., eosinophil interactions with α-smooth muscle actin-positive (α-SMA+) stromal cells that induce the secretion of TGF-β1 by eosinophils [265], and the fusion of BM-derived macrophages with intestinal stromal cells [266]. Additionally, Rho pathway activation promotes chronic production of CTGF that also induces fibrogenesis [267]. Beyond the intestine, RIF has also been involved in the development of vaginal stenosis, cystitis and bladder damage [191].

Cutaneous DEARE encompass an increased risk of skin cancers, chronic dermatitis, hyperkeratosis, pigmentary alterations, hair loss, telangiectasia, hemangiomas, fibrosis, and cutaneous atrophy [109].

Long-term respiratory complications of IR exposure include pneumonitis, chronic bronchitis, progressive pulmonary fibrosis, and an increased risk of lung cancer [182,192,268]. IR induces senescence of type II pneumocytes, impairing alveolar repair and surfactant production. Senescent cells release pro-inflammatory and pro-fibrotic cytokines, such as IL-6, and CCL2, TGF-β, that recruit and polarize macrophages toward an M2 phenotype, sustaining chronic inflammation and promoting pulmonary fibrosis [105,254,257]. Clinically, RILF presents with worsening dyspnea, progressive declines in pulmonary function, and interstitial fluid accumulation, which may ultimately progress to respiratory failure [182,251]. Although corticosteroids and other anti-inflammatory agents can mitigate symptoms of acute pneumonitis, non-pharmacologic interventions have shown efficacy in managing established RILF [192,251].

Potential late cardiac adverse effects of RT for lung or breast cancer include coronary artery disease, pericarditis, valvular injury, congestive heart failure, and myocardial infarction [203,269,270]. These effects arise because RT concurrently damages the macrovasculature (e.g., coronary arteries), the microvasculature, and the myocardium itself [106,271,272]. Radiation-induced DNA damage and telomere dysfunction promote endothelial senescence and chronic inflammation, accelerating monocyte recruitment, lipid deposition, and atherosclerotic plaque progression [106,272,273]. Progressive atherosclerosis and fibrosis in the coronary arteries compromise myocardial perfusion, further exacerbated by microvascular rarefaction and endothelial dysfunction [274,275,276]. Myocardial fibrosis is driven by sustained mitochondrial ROS overproduction and persistent elevation of TGF-β and PDGF. The resulting ventricular stiffening reduces compliance and impairs both systolic and diastolic function, ultimately leading to cardiac insufficiency [86,277], which is often accompanied by pulmonary congestion that further accelerates the progression to heart failure [269]. In the absence of effective radioprotectors or radiomitigators, minimizing cardiac dose through advanced RT techniques and ensuring systematic post-treatment surveillance remain the only evidence-based strategies to reduce heart DEARE.

Renal fibrosis is the terminal stage of radiation nephropathy, typically preceded by a long asymptomatic latent period, followed by chronic progression characterized by edema, azotemia, proteinuria, hypertension, anemia, and chronic kidney disease [278,279]. In radionuclide therapy, kidneys are particularly radiosensitive because ~3% of the administered activity is reabsorbed and retained by the proximal tubules, leading to prolonged local exposure [279,280]. Key mechanisms include cell death, oxidative stress, vascular dysfunction, cellular senescence, chronic inflammation, fibrogenesis, and RAS activation [279,281]. RAS inhibition with angiotensin converting enzyme inhibitors (ACEis) or angiotensin II receptor blockers (ARBs) has been shown to be a good strategy to prevent and attenuate IR-induced nephropathy even in subjects with normal baseline RAS activity [282].

Although effective therapeutic options for DEARE are lacking once clinical manifestations appear [24,34,48,197], administration of radiomitigators shortly after IR exposure has the potential to mitigate the risk of DEARE development [197,260,277].

## 3. Approved MCM for Radiation Emergencies: From FDA Standards to a Global Perspective

FDA-approved MCM used to protect individuals from unintended or unexpected radiation injury (see Table 1) include a limited number of radiomitigators used to attenuate H-ARS, as well as chelating agents intended to prevent absorption or accelerate the decorporation of specific radionuclides [15,39,42]. Aside from iodine prophylaxis, no radioprotective agents are currently part of the FDA-approved radiation MCM [283]. To ensure rapid response capability and adequate care for potential victims, the WHO recommends including most FDA-approved MCM in national stockpiles for radiological and nuclear emergencies [284].

### 3.1. Potassium Iodide (KI)

There are 36 known iodine radioisotopes (^108^I to ^144^I), mostly with half-lives under 60 days. ^129^I, ^131^I, and ^133^I are produced, in substantial quantities, as fission subproducts during uranium-fueled nuclear reactor operations. Among these isotopes, ^131^I (half-life: 8.02 days) is also routinely used in nuclear medicine for both diagnostic and therapeutic purposes [285,286,287]. In the event of a nuclear reactor accident, such as Chernobyl or Fukushima, radioactive iodine isotopes can be released into the atmosphere and subsequently internalized by the human body through inhalation or contaminated food [288,289]. The thyroid gland is the most exposed organ, as radioactive iodine is actively taken up and concentrated via the sodium/iodide symporter (NIS) [289]. Consequently, an increased incidence of papillary thyroid cancer and benign thyroid nodules, along with a higher prevalence of autoimmune thyroid diseases, has been observed after accidental exposures [288,290].

The uptake of iodine by the thyroid gland is regulated by TSH and by an intrinsic autoregulatory mechanism, whereby excess I^−^ in thyrocytes transiently downregulates NIS expression for ~48 h (*Wolff–Chaikoff effect*) [291]. Based on this physiological mechanism, the WHO recommends administering a single 130 mg dose of KI (≈100 mg iodine) to adults, adolescents, pregnant and breastfeeding women either within 24 h before anticipated radioiodine exposure or up to 2 h after exposure to saturate the thyroid and block radioactive iodine uptake [284,292]. KI should be given primarily to infants, nursing mothers and children because they are at greatest risk [292]. Thyroid protection is nearly complete when KI is administered within this period, but its efficacy is reduced to 80%, 40% and 7% when administered at 2, 8 or 24 h after exposure, respectively [293]. Pre-existing thyroid disorders and different nutritional habits affect the KI required for effective radioprotection [289,294,295]. In the case of prolonged radioiodine exposures, repeated KI dosing may be required to maintain effective thyroid protection [296]. Findings from the PRIODAC project support the potential safety and efficacy of daily dosing (2 × 65-mg KI tablets per day) for up to 7 days in individuals aged ≥12 years, under specific controlled conditions [297].

Perchlorate, like iodine, is a potent NIS blocker but is not subject to the saturation mechanism underlying the escape from the *Wolff–Chaikoff effect* [298,299]. A biokinetic two-compartment model indicates that, during acute radioiodine exposure, KI affords reduced thyroid protection in Japanese individuals compared with Caucasians due to delayed iodine saturation, whereas a single 1000 mg perchlorate dose appears more effective [295]. In cases of continuous radioiodine exposure, perchlorate provides better thyroid protection than KI in both Caucasian and Japanese populations [294,295], although the WHO has not revised its recommendations at this time [284].

### 3.2. Prussian Blue

Cesium radionuclides (^134^Cs and ^137^Cs) are major contaminants released during nuclear incidents. Among them, ^137^Cs poses particular concern: its widespread industrial availability makes it a high-risk candidate for misuse in “dirty bombs,” and poses long-term radiological hazards to human health through food chain contamination [15,300].

Prussian blue (PB), or ferric hexacyanoferrate (Fe_4_[Fe(CN)_6_]_3_·18H_2_O), is administered orally and acts as an insoluble cation-exchange compound that selectively binds monovalent cations, such as Cs and Tl radioisotopes—whether ingested or secreted into the intestine via the bile—thereby minimizing intestinal absorption/reabsorption and accelerating GI clearance [301]. PB is exclusively manufactured and supplied by a German company under the brand name of Radiogardase^®^ and has a limited availability in several countries. Although Pru-Decorp™ is authorized and marketed in India as a pharmaceutically equivalent formulation to Radiogardase^®^, the formulation has not been reviewed or approved by the FDA [302].

### 3.3. Ca-DTPA and Zn-DTPA

Incorporation of transuranic radionuclides, such as the isotopes of plutonium (^238^Pu, ^239^Pu, ^240^Pu), americium (^241^Am), and curium (^243^Cm and ^244^Cm), poses a substantial radiological hazard because their α-particle emissions chronically irradiate critical organs, primarily the lungs, liver, and bones, during decades of biological retention, thereby increasing the risk of late fibrosis and malignancy [303,304]. Clinical management of internal contamination with these actinides relies on the chelating agent diethylenetriaminepentaacetate (DTPA), which enhances their urinary excretion [28,305]. DTPA is not indicated for uranium or neptunium contamination, because it does not significantly increase their elimination and may exacerbate nephrotoxicity [306].

DTPA is available as Ca-DTPA and Zn-DTPA formulations for IV and aerosol administration. Ca-DTPA is about 10-fold more effective than Zn-DTPA in removing radionuclides from the body and is therefore preferred for initial administration in the first 24 h of exposure, followed by daily Zn-DTPA for maintenance therapy [180,307]. In pregnant women and pediatric patients, Zn-DTPA is preferred as the first-line treatment to reduce the risk of electrolyte imbalance, particularly zinc depletion, associated with Ca-DTPA [308]. Aerosolized Ca-DTPA, typically administered alongside IV dosing, is reserved for inhalation exposures to increase pulmonary decorporation efficacy [306]. For certain inhaled radionuclides (e.g., ^192^Ir, ^90^Sr, or ^210^Po), bronchoalveolar lavage may be considered as an alternative measure, as it is an established clinical procedure for removing particulate material from the distal airways [180]. Transuranic radionuclides do not readily cross intact skin, but prompt wound irrigation with Ca-DTPA is advised to limit topical absorption [306]. Prolonged Ca-DTPA treatment can lead to depletion of essential metals such as zinc and manganese or cause bronchospasm in asthmatic patients when administered as an aerosol, but these adverse effects are generally tolerable and outweighed by the therapeutic benefits [15,307].

Additional agents, including barium sulfate, calcium salts, sodium alginate, aluminum compounds, deferoxamine, and sodium bicarbonate, have been evaluated for reducing GI absorption or enhancing decorporation of internally deposited radionuclides [7,15,180]. However, supporting clinical evidence remains insufficient and none have been FDA-approved [42], underscoring the need for safe, effective, broad-spectrum countermeasures that do not rely on IV administration and can be rapidly deployed in emergency settings [307,309].

### 3.4. H-ARS Radiomitigators

Eleven FDA-approved H-ARS radiomitigators (Table 1) consist of genetically engineered hematopoietic growth factors originally developed for approved clinical indications and subsequently repurposed as MCM to promote hematopoietic recovery in patients with IR-induced myelosuppression [22].

G-CSF and GM-CSF stimulate the production and mobilization of granulocytes (neutrophils, eosinophils, and basophils) and monocytes/macrophages, which are critical for both fighting infections and tissue repair. Post-TBI (7.5 Gy) administration of rhG-CSFs analogs, Filgrastim (Neupogen^®^) or PEG-filgrastim (Neulasta^®^) accelerates neutrophil recovery, reduces the risk of infection and improves survival rates in mice and non-human primates (NHPs) [310,311,312,313]. Neupogen^®^ administered 24 h after LD50/60 TBI (7.5 Gy) reduced myelosuppression and improved survival in NHPs (78.3% vs. 40%) [310] but showed no survival benefit beyond 48 h [314]. PEG-filgrastim derivates possess longer half-lives and more potent hematopoietic properties than their non-PEGylated counterparts, providing the advantage of less frequent administration, a feature that may be particularly suitable for space exploration or large-scale radiation emergencies [17,26]. Filgrastim or PEG-filgrastim after a radiation dose >10 Gy accelerated neutrophil recovery only when a small amount of BM was shielded, but did not significantly improve NHP survival nor decrease incidence of infections [315]. Recent studies conclude that the use of Neupogen^®^ has no impact on acute or chronic IR-induced kidney injury nor on the latency, incidence, severity or progression of pneumonitis in NHP exposed to a PBI with minimal BM sparing [181]. Moreover, cases of respiratory failure have been reported in patients undergoing HSCT during G-CSF-driven neutrophil recovery [316,317]. This complication has been attributed to G-CSF-induced neutrophil sequestration in the lungs, which can exacerbate injury to pulmonary endothelial and epithelial cells previously compromised by repeated CT [316].

GM-CSF stimulates a JAK2 STAT1/STAT3 pathway, promoting the survival and activation of monocytes/macrophages, neutrophils, and myeloid-derived dendritic cells. RhGM-CSF treatment (sargramostim/Leukine^®^), starting 48 h after TBI at a 50–60% lethal-dose at day 60, increased day 60 survival of NHP to 78% vs. 42% in controls without intensive supportive care. Neutrophil, lymphocytes, and platelet recovery were accelerated and documented infections decreased, supporting the increasing NHP survival [318]. Additional delays in sargramostim administration at 72, 96, and 120 h post-irradiation were also effective [319]. Between 1986 and 2022, 28 ARS patients across seven radiation accidents received rhGM-CSF; 18 survived, and the therapy was well-tolerated [320]. Although there are no trials in humans comparing rates of granulocyte recovery between filgrastim, peg-filgrastim and sargramostim, they appear similar when tested in comparable clinical settings [321]. However, clinical use of filgrastim and pegfilgrastim is significantly greater than that of sargramostim, even though they have several disadvantages: (a) they must be administered no later than 24 h after irradiation [21], and (b) they are ineffective with minimal or no supportive care (including blood products and antibiotics) [310,311]. In contrast, sargramostim can be given up to 48 h after exposure and remains effective with moderate supportive care [319,322].

Beyond its hematopoietic effects, GM-CSF is a pleiotropic growth factor that promotes epithelial and mucosal repair by stimulating the production of pro-healing mediators and inducing endothelial and keratinocyte proliferation, as well as epidermal regeneration [323]. Results from clinical trials suggest that rhGM-CSF mouthwash is an effective strategy for RIOM prevention and attenuation [324,325,326], whereas the benefit of subcutaneous (SC) administration remains controversial [156,327,328,329].

As shown in Table 1, several filgrastim (Nypozi^®^, Zarxio^®^, Releuko^®^) and pegfilgrastim biosimilars (Udenyca^®^, Stimufend^®^, Ziextenzo^®^, Fylnetra^®^) have recently been approved by the FDA for the management of H-ARS [42]. When administered subcutaneously, they replicate the clinical effects of their reference products (Neupogen^®^ and Neulasta^®^) by restoring neutrophil counts and reducing the severity and duration of IR-induced myelosuppression. FDA approval followed the 351(k)-biosimilar pathway, which relies on demonstrating high analytical and functional similarity to the reference biologic, alongside pharmacokinetic and pharmacodynamic comparability in humans. Although these biosimilars are not yet included in WHO stockpile recommendations [284], their future incorporation would expand stockpile availability, reduce costs, and enhance preparedness for radiological or nuclear emergencies. None of these molecules promote recovery from lymphocyte depletion following IR exposure [210]. Incomplete restoration of T cell function increases the risk of infections, morbidity from radiation-induced multiorgan injuries, and DEARE manifestations, particularly an elevated risk of carcinogenesis [309].

Thrombocytopenia is a primary contributor to the morbidity and mortality of H-ARS, significantly increasing the risk of lethal hemorrhage [149]. Romiplostim (Nplate^®^), a TPO receptor agonist originally developed for the treatment of thrombocytopenia, is currently the only FDA-approved agent for H-ARS in mass casualty scenarios (Table 1) [42]. Yamaguchi et al. achieved a 100% survival rate in C57BL/6J mice exposed to a γ-rays LD (7 Gy) after intraperitoneal administration of three consecutive daily doses of romiplostim (50 µg/kg). By day 30, romiplostim-treated mice had fully recovered their platelet and BM HSCs counts, with evidence of healing in γ-irradiation-damaged GI tissues [330]. Similarly, in mice subjected to TBI (LD_70/30_), an SC single dose of romiplostim (30 µg/kg) administered 24 h after irradiation hastened platelet recovery and improved survival by 40% (57% versus 17% for control) [331]. In contrast, romiplostim had no notable effect on other blood cell counts and no further survival benefit was seen with higher (100 µg/kg) or more frequent dosing [331]. On the contrary, the coadministration of romiplostim with pegfilgrastim resulted in a further improvement of neutrophils in addition to the platelet response, suggesting a synergistic effect in NHPs [27]. Romiplostim administration also restored splenic hematopoiesis and BM cellularity, prevented liver atrophy, and suppressed the expression of specific miRNAs (miR-296-5p, miR-328-3p, and miR-486-5p) associated with radiation-induced chronic myeloid leukemia in mice exposed to 7 Gy of ^137^Cs γ-rays TBI [332]. Furthermore, romiplostim demonstrated hematological and survival benefits when combined with other growth factors such as G-CSF [333], pegfilgrastim [27] or erythropoietin [334].

### 3.5. Silverlon^®^

Silver ions exhibit potent antimicrobial activity and are widely incorporated into cream and wound dressings. Silverlon^®^, a silver-nylon dressing initially developed for burns and traumatic wounds, has recently received FDA clearance for the management of RID and cutaneous radiation injuries [42]. This approval was supported by early clinical studies in patients undergoing RT, which demonstrated that these dressings accelerate healing, relieve patient-reported symptoms such as itching and pain, and improve RID severity more effectively than silver sulfadiazine, corticosteroids, or humectants such as aloe vera [335,336]. Silver sulfadiazine cream (1%) can prevent RID and, once moist desquamation occurs, reduce the risk of secondary infections [337]. However, prophylactic use is not recommended due to hypersensitivity risk and potential antimicrobial resistance [178].

### 3.6. Global Research Contributions to Radiation MCM Development

Although the FDA regulatory framework serves as a global benchmark for radiation MCM approval, international research efforts, particularly in countries with advanced nuclear technologies, have made significant contributions in the advance of radiation injury prevention, mitigation, and clinical management.

In Russia, radioprotection has long been integrated into civil defense and emergency medical practice. Biodosimetry research and mechanistic advances have been prioritized alongside the development of pharmacologic strategies targeting ARS [338,339]. Notable examples include Indralin (B-190) and cytokine-based approaches such as rhIL-1β (Betaleukin), both approved for emergency management of IR-induced myelosuppression [61,340]. Indralin, an α1-adrenomimetic agent, limits oxygen-dependent radiation damage through transient vasoconstriction. In large animal TBI models (canines and NHP), indralin demonstrated a dose-reduction fraction (DRF) in the range of approximately 1.3–1.5, consistent with significant attenuation of H-ARS–related mortality [341,342,343]. In rats exposed to 9.5 Gy, combined prophylaxis with indralin and mexamine nearly eliminated mortality attributable to GI-ARS, which reached a mortality rate of 60% in untreated controls by day 7 after irradiation [344]. Protective effects have also been reported for early and delayed local radiation-induced skin injury, including combined radiation trauma [345,346]. Betaleukin has been proposed as a radiomitigator, as postexposure administration attenuated H-ARS in murine and canine models [347,348]. Combination studies further demonstrated enhanced protection when administered with indralin [349]. Nationally approved decorporation strategies include Pentacin and Zincacin as biosimilar formulations of Ca-DTPA and Zn-DTPA, as well as Ferrocin and Phosphalugel, which reduce intestinal absorption and promote fecal elimination of cesium and strontium [338,340].

In China, research has extensively evaluated bioactive compounds derived from traditional medicine, including plant extracts, polyphenols, alkaloids, and plant or microbial polysaccharides, among others, as well as synthetic radioprotective and radiomitigative candidates (see Section 4) [350,351]. Technological advances have further enabled nanoparticle (NP)-based delivery systems designed to enhance active compound bioavailability, improve tissue targeting, and increase therapeutic efficacy without impairing tumor control by RT [351].

Japanese initiatives have strengthened population-specific preparedness strategies, including optimized thyroid protection protocols and comprehensive emergency medical response systems for large-scale exposure scenarios [352,353]. In parallel, research has advanced the development of highly sensitive biodosimetry platforms and analytical tools for early detection and dose estimation, strengthening rapid triage capacity and exposure assessment [354]. Significant efforts have also focused on mechanistic and epidemiological evaluation of radiation effects at low and protracted dose levels, contributing to refinement of risk assessment models and to improved characterization of chronic inflammatory responses, genomic instability, and both cancer and non-cancer late effects [355].

However, advances in the field extend well beyond the priorities of any single country. International research efforts have collectively strengthened the development of more effective radiation MCM by deepening understanding of the molecular, cellular, and tissue-level mechanisms underlying both acute and chronic radiation injury.

## 4. Radiomitigators in Focus: In Vivo Preclinical Success and Clinical Trial Results

Only fifteen MCM are currently FDA-approved for radiation emergencies, many of which exhibit overlapping biological effects, particularly in mobilizing neutrophils and monocytes [42]. None provides specific efficacy against GI-ARS [356], and several require repeated administration under conditions of advanced supportive care that may not be feasible in mass-casualty scenarios. This limited portfolio contrasts sharply with decades of intensive research devoted to the development of radioprotectors, radiomitigators, and combined strategies aimed at preventing or attenuating IR-induced injury [283].

In this context, a critical evaluation of radiomitigator efficacy is essential to bridge mechanistic insights with translational applicability. Accordingly, this section synthesizes and discusses global scientific progress on radiomitigators, focusing specifically on agents that have demonstrated efficacy in preclinical in vivo models or clinical studies, as documented in peer-reviewed English- and Spanish-language publications, with emphasis on translational potential and mechanistic coherence. By examining experimental and clinical outcomes in parallel, this review seeks to clarify areas of convergence and discrepancy, identify persistent limitations, and highlight mechanistic and translational gaps that must be addressed to advance the development of more effective radiomitigation strategies.

In undertaking this analysis, we encountered recurrent inconsistencies in terminology and classification within scientific literature. Although the distinction between radioprotectors, radiomitigators, and radiotherapeutics is conceptually well established, scientific articles often use these terms interchangeably. It can be challenging to determine whether certain agents function as radioprotectors or radiomitigators if administered both during and after IR exposure. In such cases, we operationally define them as radiomitigators whenever continuation of treatment after exposure is considered essential to prevent or attenuate tissue injury. Furthermore, the distinction between radiomitigators and therapeutic agents may overlap, as treatment of ARS not only prevents clinical deterioration but may also reduce the risk or severity of subsequent DEARE. Notably, filgrastim and pegfilgrastim, formally classified as radiomitigators, have been administered both before and after the onset of hematopoietic manifestations of H-ARS in nuclear emergency settings, serving both mitigative and therapeutic roles, respectively (see previous section).

*Methods for literature selection*

The literature reviewed in this section was identified through a comprehensive search of PubMed/MEDLINE, Embase, and ClinicalTrials.gov, supplemented by manual searches of reference lists from relevant reviews, meta-analyses, and primary research articles. Search terms comprised combinations of the following keywords, using both full terms and their corresponding abbreviations: radiomitigator, radioprotector, radiation injury, radiation damage, acute radiation syndrome (ARS), delayed effects of acute radiation exposure (DEARE), radiation-induced fibrosis, total body irradiation (TBI), whole-body irradiation (WBI), partial body irradiation (PBI), radiotherapy (RT), chemoradiotherapy (CRT), as well as specific compound names identified during screening. The final search was updated on 25 December 2025.

To ensure translational relevance, eligible agents were required to demonstrate in vivo radiomitigative efficacy in established radiation injury models. Studies were considered if they reported at least one of the following outcomes: improved survival; prevention or attenuation of ARS; mitigation of radiation-induced tissue injury; preservation of organ function; or prevention or delay of DEARE, assessed using objective and quantifiable endpoints. Studies limited to in vitro systems were excluded, except when required to support or clarify mechanisms of action.

Regarding the selection of clinical trials, we included studies evaluating radiomitigators for the prevention or mitigation of RT- or CRT-induced toxicities, irrespective of whether administration occurred strictly post-exposure. This approach reflects the translational reality that agents demonstrating radiomitigative efficacy in preclinical models are frequently evaluated clinically in prophylactic, concomitant, or therapeutic contexts. Careful examination of registered trial identifiers, therapeutic protocols, study populations, publication timelines, completion dates, and authorship enabled the identification and exclusion of duplicate reports or overlapping datasets. For data presentation and analysis, only the most complete and methodologically robust dataset was retained. In addition, to strengthen the clinical relevance and scientific robustness of the evidence presented, we incorporated findings from systematic reviews, meta-analyses, and established clinical guidelines.

### 4.1. Thrombopoietin Mimetics or Receptor Agonists

IR-associated thrombocytopenia can lead to life-threatening complications, including intracranial and internal organ hemorrhage, particularly when platelet counts fall below 20 × 10^9^/L [357]. Initial treatment with recombinant TPO agonists and cytokines such as IL-11 achieved only transient platelet recovery and was limited by neutralizing antibody formation, which was generally associated with immune-mediated thrombocytopenia [149,358,359]. These limitations prompted the development of second-generation TPO receptor agonists, including eltrombopag, avatrombopag, and hetrombopag, which lack sequence homology to endogenous TPO, thereby minimizing cross-reactive immune responses.

A limited number of clinical trials and observational studies have evaluated second-generation TPO receptor agonists in IR-containing regimens. The earliest report described the use of eltrombopag in a glioblastoma (GB) patient who developed thrombocytopenia following CRT, with platelet recovery observed during treatment but declining after discontinuation [360]. In HSCT settings incorporating TBI-based conditioning, eltrombopag has been associated with accelerated platelet and leukocyte engraftment and reduced transfusion requirements, particularly in older recipients [361,362,363]. However, eltrombopag requires monitoring of liver function due to hepatotoxicity risk, and suboptimal responses have been reported despite full dosing [363,364].

Avatrombopag, which exhibits higher bioavailability and lower hepatotoxicity risk compared with eltrombopag, achieved an overall response rate of 58.8% at 6 months in a retrospective study of CRT-associated aplastic anemia, including patients previously refractory to eltrombopag [364]. Eltrombopag remains the only TPO receptor agonist authorized for severe aplastic anemia, whereas avatrombopag is approved for adults who have failed prior therapy [364]. In the allogeneic HSCT setting, including regimens that may incorporate TBI, hetrombopag accelerated platelet and neutrophil engraftment and reduced poor graft function and transfusion dependency [365], while in post-transplant thrombocytopenia refractory to rhTPO, response rates reached 81%, including 62% complete responses, with 71% maintaining their best response after discontinuation [366]. In CRT-associated thrombocytopenia, avatrombopag and hetrombopag also demonstrated rapid and sustained platelet recovery, with response rates exceeding 50% by 3–6 months for avatrombopag [364] and median time to platelet response of 7–8 days for hetrombopag [357,367,368]. Compared with eltrombopag, hetrombopag has demonstrated stronger activation of downstream signaling pathways, earlier onset of action, and more sustained efficacy, translating into improved clinical responses at equivalent doses and suggesting potential therapeutic advantages [357].

JNJ-26366821 is a PEGylated TPO mimetic developed to improve bioavailability while limiting immunogenicity [369] that has demonstrated both radioprotective and radiomitigative effects in the context of H-ARS [370,371]. Prophylactic SC administration 2–24 h prior to lethal ^60^Co γ-irradiation achieved 100% survival, with a DRF of approximately 1.36 and rapid correction of neutropenia and thrombocytopenia [371]. When administered 4–24 h after LD70/30 irradiation, survival improved by 30–90% compared with vehicle controls (DRF ≈ 1.11), with durable hematopoietic recovery persisting up to 6 months post-exposure [370]. Radiomitigation was further supported when given 24 h after 8.8 Gy ^137^Cs γ-irradiation, resulting in ≥45% higher 30-day survival compared with vehicle-treated controls, with consistent protection against H-ARS across sexes and mouse strains [359]. Mechanistically, efficacy was associated not only with enhanced megakaryopoiesis but also with preservation of BM vascular and stromal niches through attenuation of IR-induced endothelial injury [359]. These vascular effects extended beyond the hematopoietic compartment, as shown in a murine whole-thorax irradiation (WTI) model in which JNJ-26366821 reduced endothelial activation, RILI, and RILF and prolonged survival [372]. Importantly, it did not promote malignant myeloid proliferation [373], and exhibited a favorable clinical safety profile, producing dose-dependent platelet increases in healthy volunteers [369].

As will be discussed in subsequent sections, other growth factors and cytokines have also been shown to promote recovery from radiation-induced thrombocytopenia in NHPs, including IL-6 [374], IL-11 [375], IL-12 [376], and growth hormone (GH) [377].

### 4.2. Thrombomodulin

Thrombomodulin (TM) is a crucial regulator of intravascular coagulation, fibrinolysis, and inflammation. It inhibits leukocyte adhesion and activation on vascular endothelial cells and forms an inactivating complex with thrombin, thereby suppressing thrombin’s procoagulant and pro-inflammatory functions. The thrombin–TM complex also activates protein C (aPC), which in turn inactivates coagulation factors Va and VIIIa and inhibits plasminogen activator inhibitor-1 (PAI-1), resulting in both anticoagulant and profibrinolytic effects [378].

Exposure to radiation induces a profound (80–90%) and sustained reduction in endothelial TM activity. This decrease has been attributed to the downregulation of TM gene expression driven by oxidative stress and pro-inflammatory cytokines such as TNF-α and TGF-β, as well as to increased proteolytic shedding of TM from the endothelial surface into the circulation [379,380]. Local TM deficiency compromises thrombin clearance and impairs the activation of protein C, thereby promoting increased vascular permeability, leukocyte adhesion, tissue edema, and thrombosis [381,382]. Consistent with these findings, in vivo studies have shown that radiation-induced intestinal damage in mice, as well as in patients undergoing RT, is accompanied by endothelial upregulation of PAI-1, whereas PAI-1 knockdown increases endothelial cell survival after irradiation [383]. Persistent TM depletion favors fibrinogenesis and the progression of chronic radiation-induced organ dysfunction, underscoring its critical role in both the acute and delayed phases of radiation injury [380,382].

Systemic administration of soluble recombinant human TM (rhTM) or recombinant aPC to lethally irradiated mice has been shown to accelerate the recovery of hematopoietic progenitor activity in the BM and significantly improve survival. Although TM is abundantly expressed in the microvasculature of normal intestine, there is sustained deficiency of endothelial TM in intestines from patients who have received abdominal RT [381]. TM administration significantly ameliorated radiation-induced intestinal injury, evidenced by a decrease in myeloperoxidase activity, TGF-β immunoreactivity, collagen-I deposition and intestinal serosal thickening [384]. Within this context, it is important to highlight that the increase in TM has been shown to play a significant role in the radiomitigative effects of tocotrienols [385,386] and statins [387,388,389].

### 4.3. Growth Factors and Interleukins

#### 4.3.1. Palifermin

The epithelia of the oral cavity and GI tract are highly radiosensitive due to rapid cellular turnover, making mucositis a frequent complication of RT characterized by epithelial atrophy, ulceration, and barrier disruption. Keratinocyte growth factor (KGF/FGF7), produced by mesenchymal cells, signals through FGFR2b expressed on epithelial cells, activating downstream MAPK/ERK, PI3K/AKT, and STAT pathways. These signaling cascades promote epithelial proliferation, enhance DNA damage repair, limit apoptosis, and support oxidative stress responses, thereby accelerating mucosal restitution and preserving barrier integrity [390,391,392].

In preclinical models, rhKGF (palifermin, Kepivance^®^) mitigated IR-induced epithelial injury in oral, intestinal, and pulmonary tissues, reduced inflammatory amplification, and improved survival [390,393,394,395,396]. A single 15 mg/kg dose of rhKGF administered 10 min after completion of lung irradiation (40 Gy in five fractions) significantly attenuated pneumonitis and RILF [394]. Beyond epithelial cytoprotection, KGF preserved thymic epithelial niches and enhanced thymopoiesis and peripheral T-cell reconstitution following HSCT in murine [397] and NHP models [398], suggesting broader tissue-protective and immunorestorative effects.

Clinically, palifermin (IV, 60 μg/kg/day for three consecutive days before and after conditioning) has demonstrated its most consistent benefit in HSCT settings. In the pivotal phase III randomized controlled trial (RCT) in patients undergoing autologous HSCT with TBI-based conditioning (NCT00041665), palifermin significantly reduced grade 3–4 RIOM (63% vs. 98%), shortened the median duration (3 vs. 9 days), and decreased opioid analgesic and total parenteral nutrition requirements without adversely affecting engraftment kinetics, relapse, or mOS [399]. These findings were supported by patient-reported outcome analyses demonstrating reduced mouth and throat soreness and improved functional status [400]. Subsequent retrospective analyses indicated that benefit was more pronounced in TBI-based regimens compared with CT-only conditioning [401], contributing to FDA approval for prevention of severe RIOM in patients undergoing myelotoxic therapy requiring HSCT [22]. More recent prospective and retrospective studies in adult and pediatric HSCT populations confirmed reductions in severe RIOM recurrence, opioid requirements, and duration of parenteral nutrition, together with improvements in QoL metrics [402,403,404,405]. Collectively, these data underpin the MASCC/ISOO guideline recommendations supporting palifermin use in patients with hematologic malignancies undergoing autologous HSCT with high-dose CT and TBI-based conditioning [406]. By contrast, although RCTs in HNC (NCT00101582, NCT00131638) demonstrated reductions in the incidence and duration of severe RIOM [407,408,409], the overall body of evidence, particularly regarding secondary endpoints such as patient-reported outcomes, treatment interruptions, and multiplicity adjusted analyses, was not considered sufficiently robust to justify inclusion of palifermin in the MASCC/ISOO guidelines for patients with HNC [156,406].

Beyond HSCT and HNC populations, a phase II RCT (NCT00094861) in unresectable NSCLC treated with concurrent CRT demonstrated that palifermin significantly reduced grade ≥ 2 dysphagia and improved treatment compliance, without adversely affecting progression-free or OS [154]. In a single-center retrospective study of non-HSCT pediatric patients with solid tumors and hematologic malignancies receiving intensive CT and/or RT, palifermin was associated with a significant reduction in grade ≥ 3 OM, shorter duration of mucosal lesions, decreased opioid use and hospitalization time, without new safety concerns [410]. Consistent with these findings, a recent meta-analysis including patients with solid tumors and hematologic malignancies treated with CT and/or RT estimated an approximately 30% relative reduction in severe OM with palifermin, although heterogeneity across tumor types and treatment regimens was noted [411]. Importantly, despite concerns regarding KGF receptor-expressing tumors, long-term follow-up across clinical trials has not demonstrated increased tumor progression, secondary malignancies, or adverse survival outcomes [154,400,407,408,409].

In clinical practice, palifermin is administered prophylactically during fractionated RT and, in some settings, continued into the immediate post-treatment period, rather than being used as a classical post-exposure radiomitigator. Although its epithelial protective properties provide mechanistic rationale for potential relevance in radiation emergency scenarios, its efficacy as a true radiomitigator would require dedicated clinical validation. Moreover, the need for IV administration could represent a practical limitation in large-scale nuclear or radiological emergencies.

#### 4.3.2. IL-11, Neumega^®^ and BBT-059^®^

Interleukin-11 (IL-11) exerts pleiotropic hematopoietic and cytoprotective effects. It directly promotes megakaryocytopoiesis, modulates inflammatory signaling, and enhances epithelial regeneration in part through induction of epidermal growth factor (EGF), thereby accelerating mucosal healing [412,413]. Its recombinant form, oprelvekin (rhIL-11, Neumega^®^), has evidenced significant protection against both H-ARS [375,412] and GI-ARS [414,415,416] in mouse and NHP models. Parenteral administration attenuated radiation-induced myelosuppression and accelerated platelet and leukocyte recovery, improving survival outcomes [375,412]. In a murine TBI model, oral IL-11 administered by gavage after irradiation significantly improved intestinal recovery and survival (25% vs. 70% at 9 Gy; 0% vs. 50% at 10 Gy), although it did not enhance hematopoietic reconstitution, likely due to limited systemic absorption [416]. In contrast, its parenteral administration counteracted radiation-induced myelosuppression and accelerated platelet and leukocyte recovery in rhesus macaques [375].

RhIL-11 reduced RIOM in hamsters by attenuating local inflammatory responses and preserving epithelial integrity [414]. Clinically, SC administration of rhIL-11 reduced pain and OM severity in patients with nasopharyngeal carcinoma receiving CRT (NCT03720340) [417]. Neumega^®^ is FDA-approved for the treatment of CT-induced thrombocytopenia and has been shown to accelerate platelet recovery after peripheral HSCT with TBI-based conditioning regimens [358]. Despite these radiomitigative properties, translation to large-scale ARS management remains limited. Clinical use requires repeated daily dosing and is associated with dose-limiting toxicities, including fluid retention, edema, arrhythmias, and hypokalemia [413]. Moreover, emerging evidence suggests that IL-11 signaling may exert pro-fibrotic effects in certain contexts, raising concerns regarding potential contributions to late radiation sequelae involved in DEARE [418].

BBT-059^®^ is a PEGylated IL-11 analog with enhanced lipophilicity and biological activity, coupled with reduced renal clearance, which permits effective administration at less frequent intervals [419]. A single SC dose of BBT-059^®^ administered 4 or 24 h after 9.35 Gy TBI increased 30-day survival from 25% in controls to 96% and 75%, respectively, with concomitant robust multilineage hematopoietic recovery [419]. Importantly, BBT-059^®^ exhibited survival comparable to PEG-G-CSF and PEG-GM-CSF at moderate TBI and outperformed both at higher doses [26]. Administered in combination 24 h after LD_90/30_ irradiation (9.27 Gy) at one-tenth of the standard dose, the three growth factors increased survival to 80%, compared with 7.5% in vehicle controls [420]. Similarly, BBT-059^®^ combined with PEGylated G-CSF, PEGylated muGM-CSF, and lisinopril (an ACEi) improved survival in rat models following 7.5 Gy TBI or 13 Gy PBI. Beyond accelerating hematopoietic recovery, this multimodal regimen also mitigated IR-induced pneumonitis and renal injury [17], representing one of the few studies to demonstrate an effective polypharmacy strategy targeting both ARS and DEARE.

#### 4.3.3. IL-12 and HemaMax™

IL-12, primarily produced by monocytes, macrophages, and dendritic cells, promotes the differentiation of naïve CD4^+^ T cells into Th1 cells, enhances the cytotoxic activity of NK and CD8^+^ T lymphocytes, and stimulates IFN-γ production, thereby strengthening immune defenses against intracellular pathogens and tumor cells [421,422]. Low-dose IL-12 has been shown to facilitate endogenous hematopoietic recovery and stem cell engraftment after irradiation, significantly improving mouse survival without causing GI toxicity [421]. In RCI models, intradermal IL-12 improved skin barrier function, preserved dendritic cells, and accelerated burn repair [423].

HemaMax™ (rhIL-12), developed to address the unmet need for effective H-ARS treatments, has shown significant survival benefits in mice and NHPs when administered after TBI [376,424,425]. In both models, survival correlated with increased plasma IFN-γ and EPO levels, as well as recovery of BM hematopoiesis and peripheral blood cell counts [424,425]. Following 7 Gy TBI (LD_90/60_), a single SC injection of rhIL-12 promoted multilineage hematopoietic recovery and enhanced NHP survival without supportive care, outperforming G-CSF (56% vs. 31%, 10 μg/kg for 18 days) [376]. In addition to attenuating the depth of blood cell nadirs, rhIL-12 reduced the incidence of severe infections and hemorrhages [425]. Hematological changes observed in a phase Ib/II safety trial (NCT01742221) in healthy subjects suggest that HemaMax™ induces transient trafficking of peripheral blood cells as part of normal immune surveillance, without evidence of undue toxicity [426].

### 4.4. Stem Cell and Extracellular Vesicle-Based Therapies

HSCT is currently recommended only as a last-resort intervention for patients with severe BM aplasia who fail to respond to hematopoietic growth factors [25,28]. Its application following accidental IR exposure is severely limited by donor availability and, critically, by the inability of patients with multi-organ injury to tolerate conditioning regimens [427], which is reflected in the poor median survival reported in several clinical series [427]. In this context, mesenchymal stromal cells (MSCs) have emerged as particularly attractive alternatives. MSCs can be isolated from multiple tissue sources, including BM, adipose tissue, umbilical cord, placenta, and peripheral blood, and can be efficiently expanded ex vivo and cryopreserved, ensuring rapid availability for clinical use [427,428]. Their immunoprivileged phenotype substantially reduces the risk of immune rejection, while their ability to migrate toward injured tissues favors their use in allogeneic settings [429,430].

Extensive preclinical studies have demonstrated that bone marrow–derived mesenchymal stromal cells (BM-MSCs) administration accelerates hematopoietic recovery, enhances survival, and mitigates H-ARS by restoring the BM microenvironment [431,432,433,434]. Comparable benefits have been reported using MSCs derived from placenta or adipose tissue, including improved recovery from severe weight loss and increased survival after lethal irradiation [435,436]. In addition, MSCs have been shown to enhance HSC engraftment and support hematopoietic reconstitution [437] and are clinically used as second-line therapy for steroid-refractory acute graft-versus-host disease [438,439].

Beyond the hematopoietic system, MSCs have shown significant protective and regenerative effects in IR-induced GI syndrome [440,441,442,443,444], RIOM [445], mandibular osteoradionecrosis [446], IR-induced pneumonitis and fibrosis [447,448,449,450], neurological deficits [451,452,453], liver injury [454,455], skin fibrosis [456,457,458], and vascular damage [455,459], ultimately improving long-term survival in mice subjected to PBI and TBI regimens. For example, a single intraperitoneal injection of MSCs within 24 h after sublethal TBI significantly reduced mortality by accelerating intestinal crypt regeneration and improving barrier function [433], while local injection of MSCs into perilesional sites of IR-induced skin injuries promoted epithelial regeneration [460]. MSC efficacy in accelerating wound healing was also observed in preclinical RCI models, with a potential reduction in secondary infections [458,461]. Mechanistically, MSCs not only promote tissue regeneration but also suppress radiation-induced inflammatory cascades, oxidative stress, endothelial dysfunction, and profibrotic signaling pathways, including TGF-β-dependent responses [444,456,462,463].

Initial assumptions attributed MSC therapeutic efficacy to direct differentiation and replacement of damaged cells; however, subsequent in vivo studies demonstrated minimal long-term engraftment, with differentiated cells accounting for less than 5% of regenerated tissue. These observations shifted the paradigm toward paracrine and endocrine mechanisms as the principal drivers of MSC-mediated radioprotection, including the secretion of growth factors, antioxidant enzymes, mRNA, and immunomodulatory mediators [444,456,462,463].

This conceptual shift has fueled growing interest in MSC-derived extracellular vesicles (MSC-EVs) as a cell-free therapeutic alternative with reduced risks of tumorigenicity and immune rejection [464,465]. MSC-EVs promote multilineage hematopoietic recovery in irradiated mice, likely by reversing radiation-induced DNA damage and apoptosis in hematopoietic cells. Both murine and human MSC-EVs have been shown to reverse radiation-induced damage to BM cells, stimulate hematopoietic proliferation and maturation, and retain biological activity after prolonged cryostorage [466,467]. MSC-EVs also alleviated radiation-induced bone loss by reducing oxidative stress and osteogenic senescence [468], and markedly enhanced intestinal epithelial regeneration, reducing mortality by up to 85% in lethal irradiation models [443,469]. The beneficial effects of MSC-EVs have also been attributed to the transfer of regulatory miRNAs that modulate DNA damage responses, inflammation, apoptosis, and tissue regeneration [465,469,470], representing an initial step toward designing, for example, miR-214-3p-enriched MSC-EVs to attenuate RILI [469].

Although clinical translation remains limited, early clinical trials and compassionate-use studies provide encouraging evidence regarding the safety and feasibility of MSC-based therapies in irradiated patients. In the context of RT-related xerostomia, multiple clinical trials have confirmed the safety of intraglandular injection of autologous adipose tissue-derived MSCs (AT-MSCs) and IFNγ-stimulated BM-MSCs. In particular, treatment with AT-MSCs was associated with alleviation of subjective dry mouth symptoms, although no significant improvements were observed in sticky saliva, swallowing difficulties, or overall xerostomia scores (NCT04776538) [471,472]. Administration of IFNγ-primed BM-MSCs facilitated restoration of salivary gland secretory function (NCT04489732) [473], whereas AT-MSC treatment was associated with increased expression of regeneration-related proteins in irradiated glands (NCT03874572) [474], supporting their local biological activity.

Treatment with autologous MSCs in patients with RILF showed no evidence of disease progression at one-year follow-up [475], findings further supported by a recent phase I trial (NCT02277145) [447]. Administration of MSCs in prostate cancer patients with severe RT-related intestinal lesions was associated with analgesic, anti-inflammatory, and anti-hemorrhagic effects [476] and, in human case studies, patients with confirmed osteonecrosis reported radiological evidence of osteogenesis on CT scans after autologous transplantation of MSCs isolated from BM or dental pulp [446]. Finally, MSCs are increasingly being used successfully to treat cutaneous injuries following accidental IR exposures, as recently reviewed by Sproull et al. [458].

MSC-based strategies have advanced from simple cell transplantation to genetically engineered MSCs overexpressing SOD3 [477], SOD2 [478], or EGFR [479], designed to maximize radiomitigative and regenerative effects. These findings represent only a prelude to the potential of MSCs or MSC-derived EVs in repairing IR-induced damage. Indeed, MSC- and EV-based therapies represent promising regenerative strategies for radiation-induced toxicities due to their anti-inflammatory, immunomodulatory, and pro-repair properties. However, several critical challenges must be addressed before widespread clinical application, including the standardization of cell and EV production; optimization of dosing, timing, and administration routes; long-term safety evaluation; and rigorous assessment of efficacy in well-powered, controlled clinical trials.

Importantly, because molecular pathways involved in tissue repair partially overlap with those implicated in tumor survival [480], systemic use of these agents cannot be considered entirely risk-free. Although current evidence does not demonstrate compromised oncologic outcomes, available data remain limited and largely preclinical. Therefore, cautious clinical implementation—preferably favoring localized or post-treatment administration—with long-term oncologic surveillance is warranted until adequately powered RCTs clarify their safety profile and therapeutic window.

### 4.5. SOD, SOD Mimetics and Nitroxides

The first clinical evidence supporting the radioprotective potential of SOD dates back to 1983, when liposomal Cu/Zn SOD reduced radiation-induced inflammation and fibrosis in two patients receiving high-dose pelvic RT [481]. Despite these promising early observations, clinical translation of native SOD has been limited by its rapid inactivation at acidic pH and proteolytic degradation within the GI tract [482]. To overcome these pharmacologic limitations, a broad range of SOD mimetics with improved stability, bioavailability, and catalytic redox activity has been developed. These compounds have demonstrated significant radiomitigative effects in both preclinical models and clinical settings (Table 2) [92,93,225,482,483,484,485,486,487,488,489,490,491,492,493,494,495,496,497,498,499,500,501,502].

Post-irradiation administration of SOD has been shown to prevent radiation-induced skin injury primarily through attenuation of oxidative stress [487,510], and to effectively treat so-called “irreversible” RIF in both porcine [483] and murine models [482]. Cu/Zn SOD reduces ROS accumulation and suppresses TGF-β1 expression in myofibroblasts, suggesting that myofibroblast differentiation and ECM remodeling may be partially reversible [511]. Consistent with this mechanism, long-term IM liposomal Cu/Zn SOD or topical Cu/Zn SOD significantly reduced established skin RIF in clinical settings [483,485], with associated reductions in pain scores in breast cancer patients [485]. In contrast, a three-month topical application of Sodermix^®^ failed to improve established fibrosis in HNC patients (NCT01771991) [506], underscoring the importance of formulation, delivery route, and disease stage.

EUK-207, a combined SOD and CAT mimetic, administered subcutaneously beginning one week after 11 Gy WTI (single-dose), significantly attenuated radiation-induced pneumonitis and fibrosis and improved survival in female rats [92,493]. Although adolescent rats developed pneumonitis earlier than adults, EUK-207 demonstrated comparable efficacy across age groups [493], and its efficacy was further increased when it was co-administered with captopril [261]. Beyond pulmonary protection, EUK-207 mitigated RID, reduced vascular damage, promoted wound healing [492], and alleviated cognitive impairment following high-dose cranial irradiation [225].

Mn porphyrins-SOD mimetics (MnTE-2-PyP^5+^, MnTDE-2-ImP^5+^, MnTnHex-2-PyP^5+^ and MnTnBuOE-2-PyP^5+^) represent some of the most potent agents in this class and exhibit additional CAT-, GPx-like, and peroxynitrite-scavenging activities [512]. MnTE-2-PyP^5+^ (AEOL-10113 or BMX-010) protected the skin, prostate, testes, and penile tissues from irradiation-induced damage and prevented the loss of erectile function caused by RT [513]. It mitigated RILI when administered (SC) within 12 h post-WTI [484,489] and reversed established injury when initiated eight weeks post-exposure [489]. These radiomitigative effects were mediated through suppression of oxidative stress and downregulation of proangiogenic and profibrotic mediators, including TGF-β [484,489]. Additional antifibrotic effects were demonstrated by reduced α-smooth muscle actin-positive fibroblasts two months after irradiation, and decreased collagen deposition in skin and bladder tissues, six months post-exposure [498]. BMX-010 ameliorated both acute and chronic radiation-induced proctitis under normoglycemic and hyperglycemic conditions, significantly reducing mucosal ulceration, inflammatory infiltrates, and late fibrotic remodeling [93,491,514]. These protective effects were mediated through activation of the Nrf2 signaling pathway, upregulation of endogenous antioxidant defenses including SOD2, and enhanced sirtuin activity, resulting in attenuation of oxidative DNA damage and suppression of inflammatory cytokine signaling in irradiated normal tissues [93,491]. In diabetic contexts, Mn porphyrin treatment further reduced insulin resistance and dampened NF-kB-driven pro-inflammatory signaling, thereby counteracting the heightened susceptibility to radiation injury observed in hyperglycemic conditions [514,515]. Importantly, BMX-010 increased the therapeutic index of RT by protecting normal tissues while enhancing tumor control in prostate cancer xenografts, resulting in improved survival in treated mice [516]. These findings are particularly relevant given that diabetic patients exhibit a higher prevalence of prostate cancer, poorer oncologic outcomes, and increased vulnerability to radiation-induced normal tissue toxicity [514]. Extending its radiomitigative profile to the hematopoietic compartment, BMX-010 reduced ROS generation and DNA damage in HSCs following TBI, thereby improving long-term engraftment capacity [490].

MnTnHex-2-PyP^5+^ outperformed MnTnBuOE-2-PyP^5+^ in rodent and NHP models of RILI, achieving comparable protection at approximately 120-fold lower doses, an effect attributed to its enhanced lipophilicity and mitochondrial accumulation [495]. In both WTI models, post-exposure SC administration of MnTnHex-2-PyP^5+^ attenuated pulmonary inflammation and delayed or reduced the severity of pneumonitis and subsequent fibrotic remodeling without detectable signals of systemic toxicity [495,499]. A structurally related Mn porphyrin, MnTDE-2-ImP^5+^ (AEOL-10150), has likewise demonstrated significant efficacy against RILI across species [494,496,497,517]. In murine and NHP WTI models, MnTDE-2-ImP^5+^ (daily, SC) significantly preserved pulmonary function and reduced pneumonitis-associated morbidity. These effects were attributed to attenuation of oxidative stress and modulation of innate inflammatory responses [494,497,517]. Compared with controls, MnTDE-2-ImP^5+^–treated macaques exhibited lower plasma TGF-β1 levels and required less dexamethasone support, supporting mitigation of both acute inflammatory injury and subsequent fibrotic progression [494].

MnTnBuOE-2-PyP^5+^ (BMX-001) enhances BM-HSCs number and function through activation of Nrf2 and upregulation of endogenous antioxidant enzymes [518]. At low SC doses, BMX-001 mitigates RT-induced damage to the oral mucosa and salivary glands, with protective effects persisting for up to 12 weeks post-exposure [519,520]. BMX-001 has also shown long-term radioprotective effects in the brain of mice [521] while simultaneously enhancing the antitumor efficacy of RT and RCT in GB models [522,523]. Similar dual protective and radiosensitizing effects have been reported across multiple tumor models, including head and neck, colon, prostate, and thyroid cancers, further supporting its potential utility in oncologic settings [516,519,524,525]. Early-phase clinical studies support the safety and translational potential of BMX-001, with no indication of compromised tumor control [503,504,505]. In patients with locally advanced HNC receiving definitive CRT, BMX-001 administration was associated with a reduction in the severity and duration of RIOM [503]. Transient hypotension was the most notable adverse event, which has been mitigated through the development of a MnTnBuOE-2-PyP^5+^ nanoformulation that preserves SOD-like catalytic activity while minimizing SNS inhibition, thereby improving hemodynamic tolerability [526].

Relative to other SOD mimetics, avasopasem manganese (GC4419) exhibits favorable pharmacologic properties and functions as a true catalytic agent, undergoing redox cycling without being consumed during O_2_^−•^ dismutation [527]. In murine high-dose focal lung irradiation model, daily GC4419 dosing initiated 24 h after 54 Gy single-fraction significantly reduced late pulmonary fibrosis, with greater mitigation observed with extended post-irradiation treatment [528]. It also reduced the extent of epithelial cell layer degradation of mouse tongue irradiated with a single dose of 17 Gy and reduced radiation recall when a second dose of radiation of 12 or 17 Gy was given two weeks later [529]. Clinically, avasopasem has generated the most advanced late-phase evidence among SOD mimetics [501,502,507]. In lung cancer patients receiving CRT (AESOP trial, NCT04529850), avasopasem reduced grade ≥ 3 esophagitis [501]. In a randomized phase IIb trial in patients with locally advanced HNC receiving definitive IMRT (60–70 Gy) with cisplatin, avasopasem significantly reduced the incidence of severe OM (43% vs. 64%) and shortened severe OM duration from 19 days to 8 days [530,531]. In the subsequent phase III ROMAN trial, the incidence of severe OM (54% vs. 64%) was not reduced to the extent predicted by the phase IIb trial; however, a statistically significant reduction in the duration of severe OM (8 vs. 18 days) was confirmed, without evidence of impaired tumor control [502,532,533]. Despite the consistent and clinically meaningful reduction in RIOM incidence and severity, the overall benefit–risk assessment in the ROMAN trial was considered insufficient to support FDA approval [507], and several important limitations must be considered before introducing GC4419 in clinical practice. To be effective, it must be administered prior to the onset of OM symptoms, implying that approximately 30–35% of patients may receive treatment unnecessarily, given that severe RIOM develops in ~65% of cases. In addition, GC4419 requires a one-hour IV infusion before each RT session, which may limit patient acceptance [530,533].

It is important to note that, similarly to other MnSOD mimetics, avasopasem has been shown to enhance the antitumoral effects of RT in different tumor models, including NSCLC, HNC, soft tissue sarcoma, prostate, and pancreatic cancer [516,527,531,534,535,536], a dual profile that can be mechanistically explained. Rather than scavenging HO^•^, which is primarily responsible for direct RT-induced DNA damage, SOD mimetics catalyze rapid O_2_^−•^ dismutation to H_2_O_2_. In normal tissues, efficient peroxide-detoxifying systems buffer this shift and limit superoxide-amplified inflammatory cascades involved in IR damage. In contrast, elevated basal ROS levels are characteristic of cancer cells, and RT/CRT further enhances oxidative and nitrosative stress. Within the TME rapid O_2_^−•^ dismutation promotes H_2_O_2_ accumulation, thereby further enhancing RT-induced cytotoxicity [516,537,538]. These data support a model in which SOD mimetics function as redox modulators that preferentially blunt superoxide-amplified cascades of normal tissue injury, while exploiting tumor redox vulnerabilities (notably peroxide handling and dysregulated redox signaling) to preserve, and, in some contexts potentiate, RT tumoricidal activity.

Nitroxides, including tempol and JP4-039, are membrane-permeable stable radicals containing a nitroxyl moiety (>NO^•^). By limiting radiation-induced ROS propagation and downstream oxidative injury, they have been shown to confer both radioprotective and radiomitigative effects across preclinical models and early-phase clinical studies (Table 2) [159,539,540,541,542,543,544]. Beyond SOD-mimetic activity, nitroxides exert radioprotection through oxidation of reduced transition metals, scavenging of oxy- and carbon-centered radicals, and attenuation of lipid peroxidation [509,545]. Tempol protects hematopoietic tissues by limiting DNA damage and lipid peroxidation and by enhancing DNA repair capacity [540]. Chronic tempol supplementation after non-lethal TBI (3 Gy) reduced carcinogenesis and improved mice survival [546], while also decreasing DSBs and clustered DNA lesions in normal tissues of tumor-bearing animals [547]. Topical formulations such as MTS-01 reduced IR-induced alopecia in preclinical models [539] and also in a phase I clinical study [508]. However, it had no effects on CRT-related anal dermatitis [509].

To enhance mitochondrial targeting, tempol was conjugated to a gramicidin-S fragment, yielding JP4-039, which exhibits superior efficacy compared with tempol and other antioxidants [548]. Systemic administration of JP4-039 24 h after TBI promoted recovery of BM progenitors, intestinal barrier integrity, and stem cell function, significantly improving mice survival [541,542,543]. The mechanisms of action of JP4-039 involve protection of cardiolipin from oxidative damage, prevention of mitochondrial cytochrome-C release, and apoptosis inhibition [99]. The attenuation of GI-damage was associated with rapid and selective induction of tight junction proteins and cytokines including IL-10, IL-17α, IL-22, TGF-β, and Notch signaling [543]. JP4-039 also prevented radiation-induced skin injury [548] and mitigated fetal radiation injury when administered maternally, preserving brain integrity and improving offspring survival [544]. Notably, this represents one of the few preclinical studies demonstrating effective and apparently safe mitigation of fetal radiation injury. Delayed administration of necrostatin-1 after JP4-039 improved survival following 9.25 Gy TBI compared with either agent alone, likely due to their complementary mechanisms of action (necroptosis vs. apoptosis inhibition) [99].

### 4.6. COX Inhibitors and Benzydamine

Indomethacin and diclofenac, non-selective COX inhibitors, promoted hematopoietic recovery when administered before, during, or after irradiation but did not improve survival in mice, likely due to impaired PGE_2_-mediated mucosal protection and aggravated GI injury [549,550,551,552]. Conversely, post-irradiation indomethacin mitigated PGE_2_-driven inflammation and restored parotid gland function after single-dose 5-Gy head-and-neck irradiation by inhibiting PGE_2_–JNK signaling, normalizing amylase expression, and recovering calcium-dependent salivary secretion [553]. Clinically (Table 3), an early study suggested that indomethacin might delay the onset and reduce the severity of RIOM [554]. However, these findings were not confirmed in a subsequent RCT (JORTC-PAL04), in which topical indomethacin provided only transient analgesic benefit in HNC patients without significant impact on mucositis severity or duration [555].

In contrast, selective COX-2 inhibitors, including meloxicam and celecoxib, have attracted greater interest as radiomitigators because they limit inflammatory prostaglandin production while largely preserving COX-1-mediated GI protection [568]. In murine models exposed to sublethal (approximately 7 Gy) and near-lethal (8–9 Gy) γ-irradiation, meloxicam administered shortly after exposure enhanced hematopoietic recovery and improved 30-day survival at doses approaching the LD50/30, effects associated with increased endogenous G-CSF production and modulation of PGE_2_-dependent hematopoietic signaling [569,570,571,572,573]. Additional preclinical studies demonstrated mitigation of radiation-induced fibrotic remodeling of the anal sphincter in rats subjected to localized pelvic irradiation [574] and attenuation of IR-induced brain injury via preservation of vascular endothelial integrity [575].

By inhibiting the IR-induced inflammatory response, celecoxib reduced paw edema in irradiated rats [72] and attenuated RID in mice exposed to a single 50 Gy localized skin dose [576]. Importantly, Liang et al. demonstrated that celecoxib reduced chemokine expression in irradiated normal skin but not in irradiated mammary tumors, suggesting preferential protection of normal tissues without compromising tumor radiosensitivity [576]. However, in combined-injury models involving TBI plus trauma, COX-2 inhibition failed to improve survival and, in some cases, worsened outcomes, likely due to suppression of adaptive inflammatory pathways required for multi-organ regeneration [356]. Although these findings have not been re-examined, more recent studies indicate that celecoxib effectively attenuates IR-induced vascular injury involved in brain damage [227] and lung injury [577].

Clinically (Table 3), celecoxib has been reported to reduce pain, pruritus, pneumonitis, and severe skin toxicity in patients receiving RT or CRT for rectal, lung, prostate, and breast cancers [561,562,564,565,578]. Although an early study failed to demonstrate efficacy in preventing RIOM [566], a more recent randomized phase II trial in patients with locally advanced HNC showed that celecoxib delayed the onset of RIOM and significantly reduced the incidence of grade 3 mucositis (1.6% vs. 21.3%), without compromising local tumor control [567]. Moreover, the clinical use of celecoxib is further supported by evidence that elevated COX-2 and PGE_2_ signaling within the TME promote tumor proliferation, angiogenesis, and immune evasion [579,580]. Preclinical studies have shown that celecoxib suppresses VEGF-mediated angiogenesis and enhances radiation-induced apoptosis, thereby increasing tumor radiosensitivity [580,581,582]. These mechanistic findings align with clinical observations indicating that the addition of celecoxib to standard RT regimens is associated with improved locoregional control and OS in selected clinical settings [562,579,580]. Collectively, these data suggest that COX-2 inhibition may mitigate RT-related toxicities while preserving, and in some contexts potentially enhancing, oncologic outcomes.

Benzydamine hydrochloride suppresses the local release of pro-inflammatory cytokines (e.g., TNF-α, IL-1 and MCP-1), inhibits polymorphonuclear leukocyte degranulation and monocyte recruitment, and thereby attenuates the IR-induced inflammatory response and edema [156,556,560,583]. Beyond its anti-inflammatory activity, it modulates nociceptor excitability and peripheral pain transmission, contributing to effective local analgesia and disruption of the inflammatory pain cycle characteristic of RIOM [583]. Multiple randomized clinical trials (Table 3) in patients with HNC receiving conventional fractionated RT (typically 60–70 Gy delivered in 1.8–2 Gy fractions) have demonstrated that benzydamine mouthwash reduces both the incidence and severity of RIOM [156,556,557,558,559,560,584], supporting its recommendation in the MASCC/ISOO clinical practice guidelines for mucositis management [406]. Its widespread adoption as a standard supportive care intervention is further reflected by its frequent use as a comparator in placebo-controlled trials [560,584,585,586], underscoring its established clinical acceptance and favorable safety profile.

### 4.7. Bevacizumab

Late-onset adverse effects of stereotactic brain radiosurgery and RT, including intracranial edema and RN, are largely driven by radiation-induced vascular injury [167,587]. Microvascular damage impairs oxygen diffusion, leading to tissue hypoxia and increased HIF-1α expression, which in turn stimulates reactive astrocytes to secrete VEGF. Because VEGF is also overexpressed by tumor cells, cranial RT frequently results in aberrant neovascularization characterized by fragile, highly permeable vessels, BBB disruption, cerebral edema, and intracranial hypertension, which further exacerbate hypoxia and RN development [56,167,588].

Clinically, approximately 50% of patients with radiologically confirmed cerebral RN develop neurological symptoms that significantly impair neurocognitive function and performance status. Corticosteroids, particularly dexamethasone, remain the standard first-line therapy despite their limited efficacy, substantial adverse effects, and potential interference with concurrent anticancer treatments, including immunotherapy [30,588]. In this context, bevacizumab, a recombinant human monoclonal antibody that binds VEGF and blocks its interaction with endothelial receptors (Flt-1 and KDR), has emerged as a rational preventive and therapeutic alternative by reducing vascular permeability, stabilizing the BBB, and limiting brain edema [167,589]. Preclinical studies have shown that prophylactic administration of bevacizumab delays onset and reduces RN severity [102,589], findings corroborated in a recent single-center retrospective study [590].

In the therapeutic setting, bevacizumab administered at 5–7.5 mg/kg every two weeks for 4–6 cycles has shown efficacy in patients with corticosteroid-refractory RN [30]. As a primary therapy for RN, it provided superior symptomatic improvement and radiographic response compared with corticosteroids (NCT01621880) [587], supporting that it can be an advantageous alternative with added antitumor benefits.

### 4.8. Trace Elements

#### 4.8.1. Selenium

Selenium is an essential micronutrient involved in DNA synthesis and redox homeostasis, largely through its role as a cofactor for at least 25 selenoenzymes including GPxs and thioredoxin reductases (TrxR) [591]. Both inorganic forms (e.g., sodium selenite) and organic derivatives (e.g., L-selenomethionine and Se-NPs) of selenium have shown promising radioprotective/radiomitigative activity at pre-clinical stages [592].

Administration of 100 µg/day of selenium (as sodium selenite or L-selenomethionine), corresponding to ~3 times the nutritional requirement, immediately following TBI and continuing for 21 weeks significantly attenuated IR-induced nephropathy, as reflected by marked reductions in blood urea nitrogen levels (from 124 mg/dL to 67 mg/dL) and improved renal histopathology, including decreased fibrosis [592]. Dose escalation to 200 µg/day further enhanced renal protection, even when supplementation was limited to 2–3 months, whereas delaying treatment initiation by 1 week reduced -but did not abolish- the radiomitigative benefit [593]. Selenomethionine also attenuated radiation-induced pneumonitis, myocardial fibrosis, and RILF by suppressing IL-4-dependent inflammatory pathways and NOX family members [594,595]. Notably, owing to higher bioavailability, Se-NPs consistently outperformed sodium selenite in mitigating radiation-induced damage to BM, liver, and kidney tissues, without inducing overt toxicity [596,597,598,599].

Clinically, selenium levels are frequently reduced at cancer diagnosis and decline further during RT [591,600,601,602]. Oral selenium supplementation (300–500 µg/day for 10 days to 6 months) was generally well tolerated, maintained circulating selenium concentrations, reduced RT-associated toxicities (Table 4), and improved QoL without impairing RT efficacy [591,600,601,602,603,604,605,606,607,608].

Se attenuated diarrhea in patients subjected to pelvic RT [601,604], reduced the incidence and duration of severe RIOM in HSCT recipients [605], protected salivary glands from ^131^I-induced injury [607] and minimized myelosuppression in NSCLC patients undergoing CRT [610]. By contrast, clinical benefits were less consistent in HNC patients, with limited effects on taste alterations [600] and no significant reduction in RIOM severity reported in some trials [609,611].

Although both preclinical and clinical studies highlight the anticancer properties of selenocompounds and their potential synergy with established anticancer therapies [59,631], none of the clinical trials summarized in Table 4 demonstrated additional antitumor efficacy or improvements in patient survival.

#### 4.8.2. Zinc

Zinc is an essential trace element that plays a central role in cellular defense, tissue repair, and immune regulation, all of which are critical for recovery following IR exposure [632]. It functions as a structural or catalytic cofactor for more than 300 enzymes and transcription factors, including SOD1, DNA and RNA polymerases, and multiple zinc-finger proteins, thereby supporting antioxidant defenses, DNA repair, and cell proliferation [633,634,635]. Zinc also induces metallothioneins, stabilizes protein sulfhydryl groups, and limits ROS propagation by displacing redox-active metals such as iron and copper [636,637]. In parallel, zinc exerts anti-inflammatory effects through inhibition of NF-κB signaling and downstream cytokines, including TNF-α and IL-1β, while promoting the expression of zinc-dependent anti-inflammatory mediators such as A20 and PPAR-α [634,638]. Finally, zinc supports wound healing and epithelial regeneration by enhancing collagen synthesis, angiogenesis, and ECM remodeling, providing a strong mechanistic basis for its radiomitigative efficacy [632,638].

These complementary mechanisms underlie the radioprotective and radiomitigative effects reported for zinc sulfate (ZnSO_4_) and zinc–L-carnosine (polaprezinc) in preclinical models [626,627,639,640,641,642,643,644,645]. As examples, zinc, acting as an antioxidant, prevented H-ARS [646], protected lens [642] and brain [644], and attenuated ^131^I-induced toxicity [640]. ZnSO_4_ delayed the onset and reduced the severity of RID and hair follicle atrophy in a rat model [639], effects that were further enhanced when combined with GH [639,643]. Compared with inorganic zinc salts, polaprezinc consistently showed superior efficacy, likely due to improved zinc bioavailability and the intrinsic antioxidant and wound-healing properties of L-carnosine [626,641,647]. Polaprezinc protected intestinal epithelium from radiation-induced apoptosis by suppressing the expression of p53, p21, and Bax genes, while also attenuating the inflammatory response [647]. Notably, daily rectal administration initiated after irradiation significantly reduced mucosal injury and accelerated tissue repair, supporting its classification as a radiomitigative agent [641].

Recent studies have expanded zinc-based radiomitigation strategies through nanotechnology. Functionalized zinc nanocomposites, including zinc coumarate and zinc caffeinate NPs, enhanced antioxidant capacity and mitigated γ-radiation-induced hepatic injury, indicating synergistic interactions between zinc ions and phenolic ligands [645]. Similarly, ZnO-NPs administered after IR exposure reduced oxidative stress, inflammation, and metabolic dysregulation in multiple organs, including the liver, kidneys, spleen, and cardiovascular system, while preserving tissue architecture and normalizing biochemical injury markers [635,648,649]. However, reports of potential metabolic disturbances associated with certain ZnO-NP formulations warrant caution and currently limit their clinical translation [650].

In clinical settings (Table 4), oral zinc supplementation delayed the onset and reduced RID severity in patients with HNC [629] and breast cancer [621]. The beneficial effects of ZnSO_4_ or zinc-L-carnosine in reducing RIOM incidence, duration, and severity in HNC patients undergoing RT or CRT are well supported by numerous retrospective studies and clinical trials [613,618,619,620,622,623,624,625,629,630,651] and a recent systematic review [652]. The attenuation of RIOM contributed to avoiding RT/CRT interruption and promoting cancer patient prognosis [625] without affecting tumor control [623,624]. However, some trials reported no significant benefit of zinc supplementation for RIOM prevention in HNC patients [616,618] or in HSCT recipients [628], highlighting heterogeneity in patient populations, zinc formulations, dosing regimens, and routes of administration. To overcome swallowing difficulties associated with mucositis, alternative zinc delivery strategies have been explored. When administered as mouthwashes [620,625], oral rinses [623,624,651], or adhesive pastes [630], zinc improved tolerability while maintaining clinical efficacy. Notably, polaprezinc rinses also delayed the onset and reduced the incidence of grade ≥ 2 esophagitis in both breast cancer patients [627] and lung cancer patients receiving CRT [651]. Although direct comparative efficacy studies are lacking, formulation-dependent differences likely contribute to different outcomes. The increased viscosity of polaprezinc suspensions containing polyacrylate [651] or sodium alginate [623,625,626,653] may prolong mucosal contact and enhance local zinc bioavailability. This could explain the clinical benefit observed with alginate-based polaprezinc suspensions in HSCT patients [653], in contrast to the lack of efficacy reported with the standard formulation tested in the same patient population [628]. In addition, sodium alginate itself promotes wound healing through its hemostatic properties and has been shown to improve RT-induced pharyngeal mucositis [654].

Zinc-dependent enzymes are essential for taste bud function, providing a biological rationale for zinc supplementation. Clinical studies indicate that ZnSO_4_ and polaprezinc rinses attenuate RT-related dysgeusia in HNC patients, although results remain inconsistent across trials [612,617,623,624].

Current evidence supports the safety and efficacy of zinc supplementation and mouthwashes, particularly for the prevention of OM and dysgeusia in cancer patients undergoing RT or CRT. Compared to other interventions, zinc administration (orally or topically) offers several advantages: it is cost-effective, well-tolerated, supports tissue maintenance and repair and enhances immune responses [620,632]. Nevertheless, larger RCTs are still needed to determine the optimal zinc formulation and dosage for clinical use.

### 4.9. Vitamins

#### 4.9.1. Vitamin A

Retinoic acid, the active metabolite of vitamin A, is a master regulator of cell proliferation, differentiation, immune response, development, reproduction, and cellular metabolism. Several preclinical studies have confirmed that IR exposure decreases retinoic acid levels in multiple tissues (e.g., plasma, intestine, lungs, and heart) in mice and NHPs due to a decrease in biosynthesis and increased degradation [655]. This reduction can compromise the epithelial barrier integrity and immune cell differentiation, thereby enhancing IR-induced damages. In murine models, vitamin A supplementation prevented acute radiation-induced defects in wound healing [656] and protected the small intestine from acute 20 Gy radiation injury by reducing inflammation [657]. Furthermore, by decreasing IL-6 and TGF-β1 production, retinoic acid reversed intestinal fibrosis and RILF, significantly improving mouse survival [658,659].

In a small clinical trial (11 patients), daily topical application of tretinoin cream (vitamin A 0.25 mg) from the start of BM transplantation (BMT) conditioning until engraftment reduced the severity of RIOM [660]. However, conclusions were limited by the small sample size, and irritant effects of retinoic acid on the skin and mucosa may restrict its clinical use in this setting.

#### 4.9.2. Vitamin C

The antioxidant properties of ascorbic acid (vitamin C) underlie its radioprotective effects against DNA, lipid, and protein damage induced by IR [287,661]. High-dose ascorbic acid (3 g/kg), administered immediately after IR exposure, significantly reduced BM apoptosis, restored hematopoietic function, and improved mouse survival after 7–8 Gy TBI. Doses below 3 g/kg or delayed administration beyond 24–36 h post-irradiation were ineffective, whereas higher doses (≥4 g/kg) were harmful [662]. When administered before, during, and after 13 Gy abdominal irradiation, ascorbic acid achieved 100% survival and reduced GI injury, highlighting the importance of dose optimization and treatment timing [663]. Additionally, as a cofactor necessary for collagen biosynthesis, ascorbic acid enhanced wound healing and protected against IR-induced sickness [664].

At pharmacologic concentrations (≥20 mM), ascorbate may exert pro-oxidant effects by generating H_2_O_2_ through metal-catalyzed oxidation [665]. Cancer cells, which often exhibit impaired antioxidant defenses and elevated levels of labile iron, are particularly susceptible to H_2_O_2_-induced DNA damage and cell death. This redox imbalance underlies the dual potential of pharmacologic ascorbate as a radioprotector of normal tissues and also as a radiosensitizer of tumors [665], and also provides a mechanistic rationale for its synergy with SOD mimetics, which similarly increase H_2_O_2_ generation [666,667]. Ascorbate (IV) has been shown to enhance the radiosensitivity of pancreatic tumors while protecting normal tissues by mitigating anemia, intestinal injury, and collagen deposition. In pancreatic cancer patients enrolled in a phase I/II trial (NCT01852890), ascorbate administered concurrently with gemcitabine and RT was associated with reduced radiation-induced intestinal toxicity and collagen deposition. Although the study was not prospectively powered to detect survival differences, treatment with ascorbate was associated with a longer mOS (21.7 vs. 12.7 months; *p* = 0.08) compared with historical controls [668], consistent with findings from a similar study in which patients received ascorbate in combination with gemcitabine [669].

Administration of vitamin C in patients undergoing computed tomography scans and radioiodine therapy has been shown to reduce DNA damage and oxidative stress [287,670,671]. It also improved QoL in breast cancer patients during CRT and follow-up [672], and alleviated inflammation and RIOM severity in HNC patients undergoing RT or CRT [673,674]. In combination with vitamin E, vitamin C has also been shown to prevent radiation-induced xerostomia in patients with HNC [160].

Although vitamin C has been shown to reduce RT-induced toxicities, some studies suggest that low concentrations may promote radioresistance [675,676]. Therefore, careful dose optimization and rigorous evaluation are essential before clinical translation.

#### 4.9.3. NAD^+^ Precursors

NAD^+^ precursors, such as nicotinamide riboside (NR) and nicotinamide mononucleotide (NMN), display radiomitigating activity by restoring intracellular NAD^+^ levels, a central coenzyme required for energy metabolism, redox homeostasis, and the cellular response to DNA damage. IR-induced DNA damage leads to hyperactivation of PARP enzymes, causing rapid NAD^+^ consumption and subsequent impairment of cellular energy metabolism and DNA repair capacity. Replenishment of NAD^+^ through NR or NMN supplementation maintains PARP-mediated DNA repair and sirtuin-dependent metabolic regulation, thereby limiting IR-induced cellular dysfunction, premature senescence, and loss of tissue regenerative capacity in normal tissues [677,678].

NR (400 mg/kg/day) administered via gavage for 21 days, with initiation 8 weeks after 6.0 Gy γ-ray TBI, temporarily restored the regenerative capacity of HSCs [677]. NR mitigated GI-ARS by reducing oxidative stress, suppressing aberrant mTORC1 activation, and limiting senescence in intestinal crypt cells, thereby preserving epithelial integrity and regenerative function [107,679]. Beyond direct effects on irradiated cells, NR partially restored gut microbial homeostasis after irradiation, increasing beneficial mucus-associated commensals while reducing opportunistic pathogens, which contributed to lower inflammation and improved mucosal repair [679]. Similarly, long-term NMN supplementation attenuated IR-induced intestinal fibrosis, an effect associated with microbiota remodeling and reduced chronic inflammatory signaling [680]. Although relatively few studies have directly assessed the radiomitigative potential of NAD^+^ precursors, their central role in coordinating DNA repair, metabolism, and tissue homeostasis strongly supports their candidacy as effective radiomitigating agents.

#### 4.9.4. Vitamin E Family Members

Multiple studies have demonstrated the antioxidant, anti-inflammatory, and anticarcinogenic efficacy of different members of the vitamin E family, including tocotrienols (δ and γ) and tocopherols (α, γ, and δ). Consistently, these compounds have shown significant radioprotective effects on the GI, vascular, and hematopoietic systems in both murine and NHP models [386,681,682,683]. Although less extensively investigated, a growing body of evidence indicates that selected vitamin E vitamers also exert radiomitigative effects when administered after IR exposure [684,685,686,687,688,689,690,691].

Early proof of post-exposure efficacy was provided by Roy et al. (1988), who showed that SC administration of α-tocopherol within 15 min after 9 Gy TBI significantly improved 30-day survival in mice, yielding a DRF of 1.11 [684]. Consistently, α-tocopherol reduced micronuclei formation and chromosomal aberrations in BM cells when administered immediately or up to 2 h after irradiation, demonstrating superior efficacy compared with vitamin C [685]. Daily intraperitoneal α-tocopheryl acetate administration (1.1 mg/day, equivalent to ~400 mg oral/day in a 70-kg adult), initiated immediately after irradiation and maintained for 12 weeks, significantly protected rats against RILF [686]. In contrast, higher doses administered over shorter periods failed to prevent RILF [692]. This lack of efficacy may be due to excessive vitamin E dosing, which depletes vitamin A stores [686], a micronutrient known to inhibit IR-induced pneumonitis [693], underscoring the importance of dose and treatment duration for mitigation strategies.

Among vitamin E family members, tocotrienols have emerged as more promising radiomitigators than tocopherols, largely due to their higher antioxidant potency, superior tissue distribution, and more efficient intestinal absorption [694]. Beyond direct free-radical scavenging, tocotrienols activate Nrf2-dependent antioxidant pathways [94,691,695], suppress radiation-induced apoptosis [696,697], and enhance DNA damage response mechanisms, including modulation of RAD50 and p53 signaling [698,699]. In parallel, tocotrienols attenuate IR-driven inflammatory cascades by inhibiting NF-κB activation and downstream mediators such as PGE_2_, TNFα, IL-1, TGF-β, and COX-2 [682,687,700] and promote intracellular degradation of HMG-CoA reductase, contributing to reduced cholesterol synthesis, preservation of endothelial function, and vascular integrity, which have been demonstrated to be essential in the attenuation of GI-ARS [696,701].

γ-Tocotrienol (GT3) and δ-tocotrienol markedly increased circulating G-CSF, which was essential for recovery from IR-induced pancytopenia [688,689,702,703]. Accordingly, δ-tocotrienol administered either before or 2–12 h after 8.75 Gy TBI provided equivalent protection against IR-induced mortality in mice, achieving 100% survival at 30 days compared with 18% in controls [687,688]. GT3-loaded liposomes, administered either 24 h before or after TBI, significantly accelerated hematopoietic recovery following exposure to high-energy radiation emitters, increasing 30-day survival to 80% compared with 0% in untreated controls [690]. In a murine model of oxygen ion (^16^O) irradiation, GT3 administered starting 3 days post-exposure attenuated cardiac dysfunction, reduced mitochondrial damage and collagen remodeling, and limited immune cell infiltration, indicating mitigation of delayed cardiovascular injury [691].

Although evidence for the radiomitigative efficacy of GT3 remains limited, its radioprotective effects in murine and NHP models of H-ARS have been highly promising. In NHPs, a single administration of GT3 given without any supportive care was equivalent in improving hematopoietic recovery to multiple doses of Neupogen^®^ and two doses of Neulasta^®^ with full supportive care, including blood products [704].

Clinical trials indicate that vitamin E formulations provide modest but consistent mitigation of radiation-induced toxicities (Table 5). In HNC patients, α-tocopherol reduced the incidence and severity of RIOM [705], while oral GT3 and δ-tocotrienol administered for 6 months improved mouth opening and subjective symptoms associated with established RIF [706]. Vitamin E (mouthwash) reduced RIOM duration in patients undergoing allogeneic HSCT [707], and oral vitamin E protected salivary gland and attenuated xerostomia in thyroid cancer patients treated with radioiodine [286,708]. Importantly, although high-dose α-tocopherol combined with β-carotene reduced acute RT toxicity, a trend toward increased local recurrence was observed [709]. This is not an isolated finding, as vitamin E has been reported to promote tumor progression and potentially compromise RT efficacy in both clinical and preclinical settings [675,710]. Taken together, these data suggest that while vitamin E supplementation may contribute to the prevention or attenuation of ARS, its use in oncology patients requires careful risk–benefit assessment.

### 4.10. Pentoxifylline Alone or in Combination with Vitamin E

Pentoxifylline (PTX) is a non-selective phosphodiesterase inhibitor initially developed to improve microcirculation by reducing blood viscosity and enhancing tissue oxygenation. Beyond these hemodynamic effects, PTX exerts potent anti-inflammatory and antifibrotic actions by suppressing TNFα, IL-1, FGF, and TGF-β1 signaling, inhibiting fibroblast activation, and promoting collagen degradation [715,730,731]. These properties make PTX particularly suitable as a radiomitigator, especially for late radiation-induced injuries dominated by chronic inflammation and fibrosis, as demonstrated in both preclinical models [731,732,733] and clinical trials [712,715].

Vitamin E coadministration further enhanced the antifibrotic effects of PTX in the skin, heart, and lungs of rats, rabbits, and pigs [734,735,736], findings that were subsequently supported by clinical studies in which the combination attenuated and, in some cases, reversed RIF in breast cancer patients [716,718,719,720]. Sustained treatment appears critical, as maximal responses occur after prolonged administration, and premature discontinuation increases the risk of relapse [717].

Despite attenuating RILI [731,732], PTX did not confer a survival benefit in murine models of H-ARS and GI-ARS [737]. By contrast, its combination with GT3 significantly improved survival compared with GT3 alone, providing complete protection even after high-dose TBI (12.5 Gy) [737]. Moreover, PTX combined with α-tocopherol significantly attenuated radiation-induced left ventricular dysfunction and reduced myocardial interstitial fibrosis in preclinical models, with efficacy observed both when administered prophylactically and after the establishment of cardiac injury, indicating modulation of late radiation-induced cardiac remodeling [738]. More recently, the combination of PTX and vitamin E has demonstrated antifibrotic activity in radiation-induced renal injury, although concerns about long-term tolerability have emerged due to weight loss observed in mouse models [739]. Clinical trials (Table 5) indicate that PTX mitigates RT-induced lung injury [714], with more consistent and durable benefits observed when combined with vitamin E [721,722].

Osteoradionecrosis can be a late terminal sequela of irradiation. When used alone, neither PTX nor tocopherol was able to reverse osteoradionecrosis, but their combination demonstrated a positive synergistic effect on lesion resolution and prevention of recurrence in both preclinical studies and clinical trials [726,740]. For the more severe cases, clodronate (a bisphosphonate that inhibits osteoclastic bone destruction) was added to the medication regimen with an impressive 59% healing rate at 9 months [726,727]. Notably, infected necrotic sites responded poorly unless antibiotic therapy was initiated first, emphasizing the importance of infection control [734]. Lumbosacral polyradiculopathy also showed clinical improvement following triple-combination therapy [741].

PTX combined with vitamin E reduced the severity and duration of RIOM and dysphagia in HNC patients [163]. In a retrospective analysis, 15 of 21 patients with chronic radiation proctitis or enteritis experienced symptom relief after 6 months of combined therapy [742]. However, no significant benefit was observed in late pelvic RT toxicity [724] or radiation-induced plexopathy even with the addition of clodronate [729]. Andreyev et al. evaluated Tocovid SupraBio^®^ and PTX in patients with persistent GI adverse effects arising after RT. Levels of EGF, PDGF and FGF were reduced consistently with trends of reduced inflammation, but no clinical benefit was demonstrated which was attributed to the inadequate absorption of tocotrienol in most participants [725]. This limitation may partly explain the heterogeneous outcomes observed across clinical studies (Table 5), because in most of them, the absorption of vitamin E was not evaluated.

### 4.11. Amino Acids and Amino Acid Precursors

#### 4.11.1. Glutamine

L-Glutamine (Gln) plays a central role in cellular metabolism, immune regulation, and tissue repair. As a GSH precursor, Gln supports antioxidant defenses [743] and contributes to NAD^+^ regeneration by directly or indirectly fueling the tricarboxylic acid cycle through glutaminolysis-derived α-ketoglutarate [744]. Serving as a primary energy substrate for intestinal epithelial cells, lymphocytes, and macrophages [745], Gln enhances tight junction integrity, preserves intestinal barrier function, and reduces bacterial translocation and systemic infections in animal models exposed to pelvic irradiation or TBI [746]. However, its efficacy in preventing or attenuating radiation enteritis or related symptoms (diarrhea, blood in stool) remains uncertain, as clinical trials (Table 6) and meta-analyses report inconsistent results [747,748,749,750].

Cancer patients frequently present nutritional deficiencies at diagnosis, which are further exacerbated by RT- and CRT-related toxicities such as anorexia, vomiting, and dysphagia [774,781]. Accordingly, Gln supplementation (Table 6) has been associated with improvements in nutritional status, immune function, tissue repair, and overall treatment tolerance during RT or CRT [770,771,781,782,783].

Small randomized trials have shown that Gln supplementation attenuates RID severity in HNC patients [753,762] and in breast cancer patients receiving adjuvant RT [774,775]. Although a larger placebo-RCT did not confirm these benefits [763], a recent meta-analysis suggests that oral Gln at doses of 20–30 g/day may reduce overall RID incidence and decrease the risk of moderate-to-severe cases [784].

Oral Gln has also been reported to mitigate RT-induced esophagitis and dysphagia in lung cancer patients undergoing CRT [771,772,773]. In HNC patients, multiple randomized studies indicate that oral or parenteral Gln supplementation reduces the incidence, severity, and duration of RIOM [156,158,162,751,752,753,755,756,757,758,759,760,761], which was associated with fewer RT/CRT interruptions and reduced opioid use [162,751,752,756,758,760,761,783,785]. Based on these clinical findings and systematic reviews [786,787,788], MASCC/ISOO guidelines recommend oral Gln for prevention of RIOM in HNC patients receiving RT or CRT [156,406,787]. In contrast, inconsistent or negative findings in HSCT populations [764,765,766,769], including reports of increased relapse and mortality in one randomized study using parenteral alanyl-glutamine supplementation [767], have precluded similar recommendations in transplant settings [406].

Although some experimental data suggest that Gln may promote tumor growth or attenuate RT/CRT efficacy in specific contexts [789], neither the clinical trials summarized in Table 6 nor the preclinical tumor models reviewed here have demonstrated increased tumor progression or compromised treatment response with Gln supplementation. Moreover, our group showed that dietary Gln increased mitochondrial glutamate in tumor cells, impaired mitochondrial GSH import, and enhanced sensitivity to RT and CT in preclinical models [790,791,792]; findings consistent with the antitumor effects of Gln reported in other experimental studies [744,793]. Nevertheless, the complex interplay between Gln metabolism, redox regulation, and treatment response warrants careful investigation, and adequately powered prospective RCTs are needed to validate these observations [744,784]. To date, Gln supplementation for the prevention or mitigation of RT-induced toxicity has not received FDA approval.

#### 4.11.2. Arginine

L-arginine (Arg) supplementation has been shown to reduce intestinal damage, oxidative stress, and inflammation. By supporting mucosal immune balance, it promotes wound healing by stimulating collagen synthesis, modulating inflammation, and enhancing fibroblast proliferation—key processes in tissue repair and regeneration [752].

In randomized trials involving HNC patients receiving RT or CRT, oral Arg supplementation, alone or combined with Gln, reduced RIOM severity and attenuated RT-associated declines in body mass index [751,752]. Although no statistically significant differences were observed between Arg and Gln monotherapy in overall mucositis severity, a higher proportion of patients receiving Arg achieved complete mucosal healing by week 7, suggesting a potential delayed therapeutic benefit [752].

Combined supplementation with Arg, Gln, and β-hydroxy-β-methylbutyrate (Arg/Gln/HMB) has demonstrated clinically relevant benefits in HNC patients undergoing concurrent CRT (Table 6). In phase II studies, this regimen slowed RIOM progression, accelerated mucosal recovery, improved nutritional parameters, including maintenance of body weight and attenuation of treatment-related cachexia [754,755], and was also associated with a reduced incidence and severity of RID in the same clinical setting [753]. Collectively, these findings support Arg, particularly in combination regimens, as a potential adjunct for RIOM mitigation. An ongoing trial (NCT07020754) is evaluating whether Arg or Gln supplementation enhances EGF production and accelerates mucosal recovery.

#### 4.11.3. N-Acetylcysteine

N-Acetylcysteine (NAC), a cysteine precursor, scavenges ROS and restores intracellular GSH pools, strengthening antioxidant defenses in irradiated tissues [104]. When administered prior to or concurrently with IR, NAC attenuated hematopoietic, GI, neurologic, cutaneous, ovarian, and cardiac radiation injury in multiple preclinical models [794,795,796,797,798,799,800]. It demonstrated efficacy comparable to amifostine in selected models of H-ARS and RID, with the notable advantage of improved tolerability [801,802].

Consistent with its radiomitigative activity, NAC administered 2 h after single-dose abdominal irradiation (20 Gy) attenuated intestinal crypt loss and mucosal injury, reduced weight loss, and improved survival to approximately 50%, compared with <5% in vehicle controls [794]. Similarly, initiation of an antioxidant-enriched diet containing NAC 24 h after 8 Gy TBI reduced BM damage and overall lethality, achieving a DRF of approximately 1.18 [803]. Beyond survival benefits, delayed NAC administration enhanced radiation-impaired anastomotic integrity and wound healing by promoting inflammation resolution, re-epithelialization, and neovascularization [804,805]. NAC also facilitated resolution of RIOM by inhibiting autophagy in irradiated buccal keratinocytes [104].

Clinically (Table 6), two placebo-controlled phase II trials in HNC patients receiving RT or CRT demonstrated that NAC reduced RIOM severity and xerostomia [778,779], while a prospective non-randomized study further reported improved QoL with NAC inhalation therapy during RT [780]. Ongoing RCTs are currently evaluating the radioprotective potential of NAC in the context of RIOM prevention in HNC patients (NCT06354712) and X-ray-guided catheter ablation procedures [806].

Despite its radioprotective and radiomitigative potential, the use of NAC in cancer patients remains controversial. As a potent antioxidant and precursor of GSH, NAC may attenuate ROS-mediated cytotoxicity induced by RT or CRT. Preclinical studies suggest that NAC supplementation can, in certain tumor contexts, promote tumor progression or reduce therapeutic efficacy by dampening oxidative stress-dependent cell death pathways [807,808,809]. Although the preclinical and clinical studies summarized here have not demonstrated reduced tumor control with NAC, these investigations were not powered to assess long-term oncologic outcomes. Given the central role of ROS/RNS in RT/CRT efficacy and tumor redox biology, systemic NAC supplementation during or after active cancer therapy warrants careful evaluation to define an appropriate benefit–risk balance.

### 4.12. ACE Inhibitors, Angiotensin II Antagonist and Ang-(1–7) Agonists

**Figure 5:**
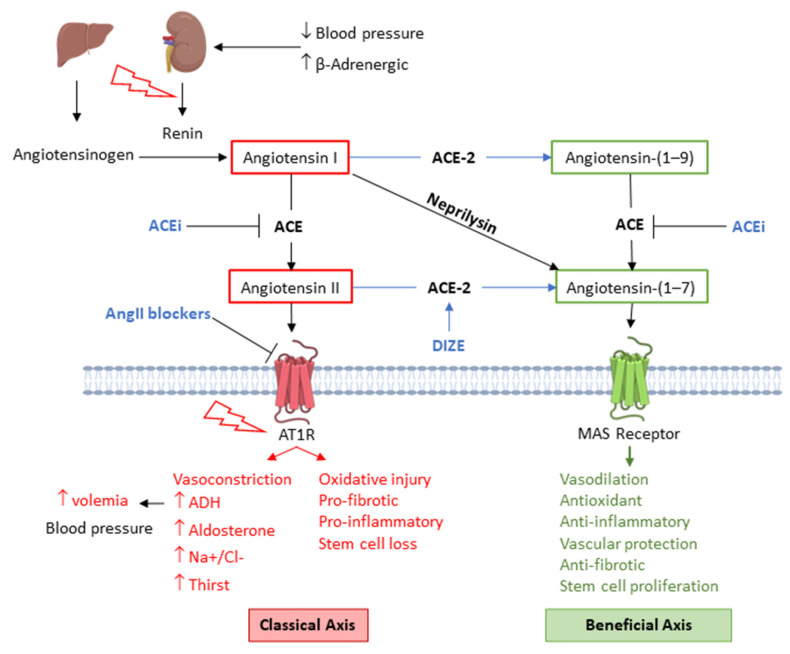
**Renin–Angiotensin system: dual axes and pharmacological approaches against IR damage.** Renin converts angiotensinogen into Ang I, and this is transformed by ACE into Ang II, which activates the AT1R, driving the classical axis. Alternatively, ACE-2 and neprilysin generate Ang-(1–7), which binds to the MAS receptor to stimulate the potential beneficial axis, promoting vasodilation, anti-inflammatory, and anti-fibrotic effects. Potential pharmacological interventions (ACEi, Ang II blockers, DIZE) are highlighted in blue. Abbreviations: ACE, angiotensin-converting enzyme; ACEi, angiotensin-converting enzyme inhibitor; Ang, angiotensin; AT1R, angiotensin II receptor type 1; DIZE, diminazene aceturate. Created in BioRender. López, R. (2026) https://BioRender.com/izwucsh and adapted from [817].

In addition to playing a key role in blood volume homeostasis, Ang II is clearly involved in IR-induced damage [810,811,812,813,814,815]. As shown in Figure 5, a drop in renal perfusion and/or sympathetic stimuli triggers renin release from the kidneys, which converts hepatic angiotensinogen to angiotensin I. Angiotensin-converting enzyme (ACE) catalyzes the synthesis of Ang II, which, through AT1 receptors (AT1R), induces vasoconstriction, stimulates thirst, and promotes aldosterone and ADH release, to restore blood volume and arterial pressure [816]. Independent of the volemic status, exposure to IR increases Ang II levels that via AT1R further enhance oxidative stress, inflammation (mediated by NOX activation), TGF-β production, collagen deposition and fibrogenesis [817,818]. ACE2 has recently been identified as a key counter-regulatory component of the classical RAS, promoting Ang-(1–7) formation with anti-inflammatory, antioxidant, vasodilatory, antifibrotic, and natriuretic effects, primarily through the Mas receptor, a G protein-coupled receptor expressed in the kidney, heart, brain, and vascular system [819,820,821,822].

Many oncology patients receive antihypertensive medications, including ACEis (e.g., captopril, enalapril, lisinopril, ramipril) or ARBs (e.g., losartan), concomitantly with RT or CRT. This overlap has prompted numerous retrospective cohort studies (summarized in Table 7), suggesting that RAS axis inhibition may prevent or mitigate RT-induced adverse effects, particularly RILI [818,823,824,825,826,827].

Captopril and enalapril markedly mitigated acute pneumonitis and RILF, resulting in improved survival in murine models exposed to TBI and WTI [71,189,811,812,814,815,838,839,840,841]. Both suppressed the release of IL-1β and TNF-α, decreased recruitment of macrophages, and downregulated TGF-β-driven fibroblast activation and collagen production [71,189,811,814,815,838]. However, captopril and L-158,809 (an AT1R antagonist) were more effective than enalapril in protecting the lung parenchyma from radiation-induced inflammation and subsequent fibrosis [71]. Mortality following 12 Gy WTI was completely prevented by administration of captopril or losartan (an AT_1_R antagonist) initiated a few hours after exposure and continued for eight weeks. In this study, captopril was more efficacious than losartan in mitigating pulmonary vasoreactivity and symptoms of post-radiation pneumonitis [812]. In the rat 13 Gy leg-out PBI model, lisinopril started 7 days after exposure and continued long-term significantly mitigated delayed radiation injuries in the lung and kidney, reducing morbidity and increasing survival through at least 150 days when given with antibiotics and hydration supportive care [842]. Additionally, lisinopril reduced the transient pulmonary hypertension observed during radiation pneumonitis and prevented cardiac remodeling and right ventricular fibrosis, enhancing OS compared with irradiated controls [843]. Delayed treatment also decreased pneumonitis severity, attenuated vascular remodeling and pulmonary fibrosis, and improved cardiopulmonary function and survival in both juvenile and aged rats [844]. Similarly, captopril reduced pericardial effusion, cardiac fibrosis and elevated left ventricular end-diastolic pressure in a high-precision proton irradiation model targeting the heart, effects associated with reduced pulmonary inflammation, cellular infiltration, and interstitial edema [841].

Consistent with these preclinical findings, several retrospective cohort studies (Table 7) in lung cancer patients receiving thoracic RT or SBRT have reported a lower incidence of symptomatic (grade ≥ 2) radiation pneumonitis among individuals treated with ACEi or ARBs [824,825,826,827,833]. Although clinical studies such as RTOG 0123 (NCT00077064) and Alliance MC1221 (NCT01880528) have suggested captopril and lisinopril may mitigate radiation-associated pulmonary toxicity and improve respiratory symptoms in lung cancer patients undergoing thoracic RT or CRT, both trials were limited by early closure or low accrual [845,846]. Two ongoing clinical trials are currently recruiting to assess the potential of losartan in preventing radiation-induced heart failure (NCT05607017) and RIF in breast cancer patients (NCT05637216).

Kidney irradiation, even at sublethal doses, often results in chronic hypertension, elevated blood urea nitrogen levels, proteinuria, progressive renal nephropathy, and fibrosis. Radiation nephropathy was a major complication of BMT when TBI was used as part of the treatment regimen, being considered inevitable, progressive, and untreatable [847]. At doses overlapping those clinically used for treatment of hypertension, captopril, enalapril and lisinopril are effective for both mitigation and treatment of established clinical IR-induced nephropathy [278,281,282,844,848,849,850]. In a DEARE model using PBI with BM shielding (13 Gy X-rays), lisinopril initiated seven days post-exposure mitigated radiation-induced nephropathy in both juvenile and aged male and female rats, improving renal function and survival [844]. Similarly, enalapril administered for three months following therapeutic [^177^Lu]-DOTATATE exposure (approximately 40 MBq) attenuated progressive renal impairment and preserved renal function [850]. The nephroprotective effect exerted by RAS inhibition is primarily mediated through the reduction in Ang II activity, leading to decreased intraglomerular hypertension, attenuation of tubulointerstitial injury, reduction in proteinuria and suppression of TGF-β1 signaling [281,282,851].

Renal injury, including chronic kidney disease, was recognized as a clinically significant late complication of TBI-based conditioning for HSCT, a setting in which a RCT (NCT00004230) demonstrated that captopril mitigated radiation nephropathy and was associated with improved OS. However, the survival benefit appeared to be driven predominantly by a reduction in pulmonary mortality (11% vs. 26% at 4 years; *p* = 0.15), rather than by a decrease in chronic renal failure [847,848]. Consistent with a broader protective role of RAS inhibition, retrospective analyses further showed that prostate cancer patients receiving ACE inhibitors were significantly less likely to develop radiation-induced proctitis, hematuria, or rectal bleeding following pelvic RT compared with non-users [836,837].

Captopril treatment (110 mg/kg/day), initiated 1–4 h after TBI (7.5 or 8.25 Gy) and continued for 30 days, significantly attenuated H-ARS and improved mouse survival [852,853,854]. Unlike other pro-proliferative agents, captopril induced a transient growth arrest in hematopoietic precursors, allowing DNA repair and a delayed but robust recovery of progenitor populations [852,854]. Similar protective effects were observed in the Göttingen minipig model, where oral captopril (0.96 mg/kg twice daily for 12 days post-TBI) improved survival (87.5% vs. 62.5% in controls) and mononuclear cell recovery [855]. Captopril also reduced brain micro-hemorrhages at 21 days post-irradiation, likely by attenuating severe thrombocytopenia [852,854]. In rats exposed to high doses of radiation (30 Gy), captopril significantly decreased both early and late phases of moist desquamation, as well as the incidence and severity of IR-induced skin tumors [811,856].

Ramipril and L-158,809, which cross the BBB, have been shown to attenuate NV-ARS [222,857,858,859,860]. When administered 24 h post-TBI (10 Gy), ramipril modestly reduced radiation-induced apoptosis and mitotic catastrophe among neural progenitors [861]. Both agents (24 h post-TBI at doses of 10 and 15 Gy) demonstrated efficacy in preventing perirhinal cortex-dependent cognitive deficits in animal models [222,858]. These effects were further enhanced when ramipril was co-administered with atorvastatin [859]. Chronic ramipril, started 2 weeks after stereotactic brain irradiation (30 Gy), reduced functional and histopathological markers of optic neuropathy at 6 months, but was ineffective when initiated 4 weeks post-irradiation [857]. In contrast, when administered immediately after IR and maintained throughout follow-up, it delayed onset and reduced the rate of paralysis and myelopathy in rats [860]. The putative mechanisms involved in neuroprotection include: (a) blockade of Ang II/NOX-mediated oxidative stress, (b) downregulation of VEGF expression and reduced microglial infiltration, and (c) restoration of the balance between Ang II and Ang-(1–7) peptides [222,860,862,863].

Managing RT-related cerebral edema in patients with intracranial tumors remains a major challenge in neuro-oncology. Across multiple malignancies, including GB, activation of the Ang II/AT1R axis has been associated with VEGF overexpression, promoting angiogenesis, vasogenic edema, necrosis, tumor cell proliferation, and immune evasion [828,830,864]. In retrospective analyses, angiotensin receptor blockade was associated with a reduced risk of symptomatic RN following stereotactic radiosurgery for brain metastases [831] and with improved outcomes in GB patients [828,829,830]. However, these findings have not been confirmed in prospective RCTs evaluating losartan or ramipril for GB CRT [229,865].

IR markedly reduces ACE2 activity and Ang-(1–7) expression [819,820,821,822], highlighting the importance of enhancing Ang-(1–7) to mitigate IR-induced damage (Figure 5) [819,820,822,863,866,867]. Consistently, daily administration of Ang-(1–7) following TBI (2–7 Gy) protected BM HSCs and restored thrombocytopenia, resulting in reduced bleeding time by day 30 and improved mice survival (from 60% to 92–97%) [866]. Ang-(1–7) attenuated RIF, stiffening, and production of profibrotic cytokines (TGF-β and CTGF) that were elevated in mouse skeletal muscles after RT for extremity sarcoma [867]. Diminazene aceturate (DIZE) markedly increased ACE2 expression (Figure 5), shifting the balance from Ang II toward Ang-(1–7), which, through the Mas receptor, ameliorated biomarkers and histopathological features of IR-induced renal injury [821] and improved survival in rat models of H-ARS and multi-organ DEARE [820,822]. Like Neulasta^®^, DIZE recovered BM cellularity and functional colony-forming capacity at both early and late time points after TBI (7.75 Gy) [822]. By upregulating IL-10 and downregulating TGF-β, DIZE also reduced late morbidities associated with IR-induced pulmonary and renal failure [822]. Given the neuroprotective properties of Ang-(1–7) [868], strategies to elevate its CNS levels may offer a potential approach to prevent radiation-associated cognitive impairment.

During the symptomatic phase of radiation pneumonitis, the ACE/ACE2 ratio is higher in males than females [820]. Consistent with this sex-specific imbalance, systemic DIZE reduced morbidity in both sexes, increased survival more markedly in males, and fully prevented morbidity in male rats during GI-ARS [820,869]. Similarly, continuous administration of captopril or valsartan (an AT1R antagonist) significantly decreased IL-6 and TNF-α secretion and mitigated X-ray-induced lung injury by preserving the balance between the ACE/Ang II/AT1R and ACE2/Ang-(1–7)/MasR axes [819]. Both treatments reduced the inflammatory response and attenuated radiation-induced pneumonitis via inhibition of MAPK and NF-κB pathways [71,189,833,838,840].

Overall, preclinical and retrospective data suggest that ACEis may attenuate radiation-induced pneumonitis and limit progression to RILF without impairing antitumor efficacy [824,825,826,827,833,845,846,847,848], thereby supporting their continued use during RT in hypertensive patients [818]. However, this recommendation cannot be extended to normotensive individuals, given potential confounding factors in observational studies and the lack of RCTs in this population. Moreover, in mass-casualty scenarios where IR-exposed patients are at risk of dehydration (e.g., from burns, vomiting, or hemorrhage), ACEis or Ang II blockers could exacerbate hypovolemic shock, limiting their applicability as a universal radioprotective strategy.

### 4.13. Statins

IR causes endothelial injury and increases serum cholesterol levels, promoting atherosclerosis progression and cumulative vascular damage [274,275]. In this context, statins (simvastatin, atorvastatin, lovastatin, and pravastatin), widely used to treat hypercholesterolemia via HMG-CoA reductase inhibition, have emerged as promising radiomitigators due to their pleiotropic vascular protective effects, including preservation of endothelial function and anti-inflammatory and antithrombotic activities [870,871,872,873]. They also inhibit TGF-β–Smad and Rho/ROCK signaling, thereby attenuating fibroblast activation and ECM deposition associated with RIF [874,875,876,877,878].

Consistent with these actions, statins exert antioxidant and anti-apoptotic effects, preserve NO-dependent endothelial relaxation, and suppress profibrotic signaling, collectively improving cardiac structure and function in irradiated rodent models [870,873,875,877,879,880]. For example, simvastatin (10 mg/kg/day), initiated 9 days after 10 Gy TBI, reduced dyslipidemia, peri-arterial fibrosis, cardiac dysfunction, and myocardial infarction severity in rats [879], while atorvastatin prevented radiation-induced conduction abnormalities and left ventricular systolic dysfunction [880]. Prolonged treatment with atorvastatin and simvastatin was required to limit cardiac fibrosis, largely through RhoA/ROCK inhibition [875,877,879]. Similarly, lovastatin and simvastatin mitigated vascular leakage, pulmonary edema, and interstitial fibrosis after WTI, improving survival in mice [387,878,881]. These effects were linked to inhibition of NF-κB and Rho/ROCK signaling, reduced TNF-α and IL-6 expression, and suppression of oxidative stress [387,878]. Clinical relevance is supported by multiple retrospective and observational studies reporting associations between statin use during or after RT and reduced rates of major adverse cardiovascular and cerebrovascular events, including stroke, and, in some cohorts, improved OS [276,882,883,884,885,886]. However, these findings remain susceptible to confounding from baseline cardiovascular risk, concomitant cardioprotective therapies, lifestyle factors, and cardiac radiation dose, underscoring the need for prospective RCTs to confirm these results.

Beyond the cardiovascular system, pravastatin and atorvastatin attenuate acute radiation-induced enteropathy by preserving endothelial function—primarily via maintenance of TM activity and suppression of PAI-1 expression—thereby limiting vascular inflammation, preventing microvascular dysfunction, and ensuring adequate nutrient delivery to support epithelial regeneration [388,389,872,887]. Additional protection conferred by atorvastatin derives from its antioxidant activity and activation of autophagy [103], whereas pravastatin mitigates IR-induced intestinal fibrosis by inhibiting the Rho/ROCK/CCN2/ECM signaling cascade [267,876]. Importantly, pravastatin did not compromise RT antitumor efficacy [267,876] and, in some models, delayed tumor growth [872].

Combination strategies further support the radiomitigative effects of statins. In mouse and minipig models, pravastatin plus metformin enhanced intestinal regeneration, increased stem cell activity, reduced inflammation, and improved survival after irradiation [888]. Notably, sex-dependent effects have been reported, as simvastatin improved hematopoietic and GI recovery in male mice but worsened outcomes in females, potentially implicating microbiota-dependent mechanisms [889]. A retrospective analysis of 308 patients treated for various pelvic malignancies found that statin use, either alone or in combination with ACEis, was associated with reduced GI toxicity both acutely and at 1 year after RT [835].

By reducing adhesion molecule expression and local chemokine and cytokine levels, pravastatin limited radiation-induced leukocyte infiltration and endothelial dysfunction, decreasing the severity of erythema and moist desquamation in mice [871]. Clinically, the phase II PRAVACUR trial demonstrated that oral pravastatin (40 mg/day for 12 months) improved radiation-induced cutaneous and SC fibrosis in HNC patients, suggesting partial reversibility of established fibrosis (NCT01268202) [874]. Consistently, topical and oral atorvastatin reduced RT-induced skin toxicity and improved breast cancer patient-reported symptoms (IRCT2016062219423N2 and IRCT20181005041239N1) [890,891], whereas lovastatin failed to reduce rectal toxicity in prostate cancer patients undergoing RT (NCT00580970) [892].

Taken together, preclinical and clinical evidence indicates that statins, administered at doses of 5–30 mg/kg/day in experimental models and 20–40 mg/day in patients, can mitigate IR-induced cardiovascular, pulmonary, GI, and cutaneous injury. Their favorable safety profile, oral bioavailability, and extensive clinical use support their repurposing as radiomitigators, with the added advantage of reported antitumor effects [886,893]. However, heterogeneity in statin type, dose, timing, and patient populations underscores the need for well-designed prospective RCTs to confirm efficacy, optimize treatment parameters, and identify patients most likely to benefit. Ongoing clinical studies are evaluating statins for managing RT-associated dysphagia in HNC patients (NCT07217938) and for improving outcomes in locally advanced rectal cancer undergoing CRT (CTRI/2018/11/016459).

### 4.14. Antifibrotic Agents

As discussed in Section 2.5, TGF-β1 is a central mediator of RIF, an important mechanism underlying both RT-related morbidity and cancer recurrence [45,192,197]. Supporting the role of TGF-β1 in RIF, intraperitoneal injection of an adenoviral vector encoding the TGF-β2 receptor in rats exposed to thoracic irradiation reduced lung TGF-β1 availability and decreased pulmonary damage [894], with similar effects in a radiation-induced liver fibrosis model [895].

Subsequently, several TGF-β receptor kinase inhibitors, including SM16 [896], SKI2162 [897], galunisertib (LY2157299) [239], LY2109761 [260], vactosertib (EW-7197) [898], P144 [899] and IPW-5371 [900,901], have been shown to specifically block TGF-β signaling and thereby suppress RIF. For instance, P144 reduced radiation-induced muscle fibrosis in rabbits [899], and a 4-week course of LY2109761 beginning 24 h after WTI (20 Gy) improved survival and reduced RILF in C57BL/6 mice [260]. Likewise, IPW-5371, administered orally for 140 days starting 24 h post-irradiation, preserved cardiopulmonary function and achieved 180-day survival rates comparable to non-irradiated controls [900]. Notably, delaying IPW-5371 administration until 15 days post-irradiation remained effective in mitigating DEARE-associated lung and kidney injury [901]. Moreover, by blocking TGF-β signaling, vactosertib not only attenuated radiation-induced oxidative stress and fibrosis but also reduced cancer stem cell properties and tumor volume in a murine model of breast cancer [898].

Single kinase inhibitors targeting PDGFR, VEGFR, SCFR, and TGFβR attenuated radiation-induced pulmonary inflammation and reduced histological markers of lung injury and fibrosis. However, simultaneous pathway inhibition resulted in a significantly greater reduction in radiation-induced pneumonitis and improved OS compared with single- or dual-agent inhibition [902]. Similarly, PDGF antagonists (SU14816 or SU9518) or the TGF-β inhibitor galunisertib sharply reduced leukocyte infiltration, fibroblast invasion, collagen deposition, and other hallmarks of pulmonary fibrosis. However, combined PDGF and TGF-β inhibition was more effective than single-pathway blockade in improving mice survival [239].

Halofuginone, a plant-derived alkaloid, inhibits T helper cell differentiation, interferes with the TGF-β signaling pathway, and suppresses collagen α1 gene expression [903]. In preclinical models, it showed efficacy in reducing RIF and limb contracture without compromising the antitumor efficacy of RT [903]. In Wistar–Albino rats exposed to 12 Gy, doses of 2.5 and 5 μg improved RILI, with 5 μg needed to prevent fibrosis at 16 weeks post-irradiation [904].

Pirfenidone (PFD, 5-methyl-1-phenyl-2-[1H]-pyridone) is a broad-spectrum antifibrotic agent that inhibits M2 polarization, myofibroblast differentiation, collagen and fibronectin synthesis, and deposition of ECM [905]. It is one of two recommended therapies for treatment of idiopathic pulmonary fibrosis. In a mouse model receiving 16 Gy thoracic irradiation, post-treatment with PFD mitigated RILF and significantly prolonged median survival (>140 vs. 73 days in controls) [248,906]. Similarly, PFD also prevented radiation-induced intestinal fibrosis in rats [907] and showed a notable mitigation in external anal sphincter thickness, concomitant with reduction in collagen deposition and preservation of muscular tissue [574]. PFD inhibited angiotensin II (Ang II)-induced left ventricular hypertrophy, by attenuating the mRNA expression of atrial TGF-β1, natriuretic peptide, and mineralocorticoid receptors [259]. A pilot clinical study confirmed that PFD is effective in ameliorating the motor disability associated with RILF (NCT00020631) [908], and two additional clinical studies demonstrated its capacity to attenuate RILI and improve lung function (ChiCTR2100043032 and NCT03902509) [909,910]. Currently, two ongoing clinical trials (NCT05801133 and NCT05704166) are investigating the potential of PFD as a prophylactic strategy against acute RILI in patients with lung and breast cancer, respectively.

Nintedanib (BIBF 1120) is a triple angiokinase inhibitor targeting VEGFR, PDGFR, and FGFR, intersecting key pathways involved in inflammation and fibrosis [911]. In acute RILI, nintedanib reduced serum TNF-α and IL-6 levels, disrupting NF-κB-mediated immune cell recruitment and oxidative stress. Its administration improved the overall health and survival of irradiated mice, while histological and longitudinal CT analyses confirmed attenuation of lung remodeling [911]. During the chronic phase, nintedanib suppressed TGF-β1 expression and Smad2 activation, with early administration proving to be essential in limiting fibrosis progression and maximize protection against RILF [911,912]. However, in patients with unresectable NSCLC undergoing CRT, oral nintedanib did not reduce the incidence of pneumonitis (NCT02496585) [913]. In contrast, the combination of nintedanib and prednisone demonstrated improvement in RT-related pulmonary fibrosis (NCT02452463) [914].

CTGF, also called CCN2, is used to indicate the severity of pulmonary fibrosis because it acts downstream of TGF-β1 and both cooperate in fibrogenesis. Although TGF-ß1 can induce fibrosis independently of CTGF, CTGF increases the activity of TGF-β1, resulting in a worsening of pulmonary fibrosis [253]. Pamrevlumab (FG-3019), a human antibody to CTGF, prevented (~50–80%) or reversed (~50%) lung remodeling, improved lung function, and rescued mice from lethal irradiation [241]. Although nintedanib and PFD are the main medications currently utilized in clinical settings for the treatment of pulmonary fibrosis, FG-3019 was more effective than any of them in the treatment of RILF [915]. Moreover, FG-3019 was the only monotherapy that significantly increased mouse survival, although it had the notable drawback of requiring intraperitoneal administration [915].

These studies highlight the complexity and extensive crosstalk among the mechanisms driving RIF. Direct inhibition of TGF-β1 can effectively suppress RIF but carries a high risk of systemic toxicity and immune disruption. In contrast, CTGF blockade provides a downstream, more tissue-selective strategy to reduce RIF with fewer systemic effects. [241]. Notably, modulation of TGF-β1 signaling also contributes to the radiomitigative actions of several agents, including somatostatin analogues, pentoxifylline, vitamin E, captopril, statins, and palifermin [197,700,715,845,875,916].

Beyond its role in fibrosis, TGF-β1 overexpression fosters tumor progression, metastasis, immune evasion, and treatment resistance, predicting poor outcomes. Consequently, TGF-β1 antagonists, neutralizing antibodies, antisense oligonucleotides, and downstream inhibitors are being clinically evaluated to enhance the efficacy of CT and RT [898,917,918]. For example, adding galunisertib to neoadjuvant CRT in locally advanced rectal cancer was well tolerated and increased complete response rates to 32% [919].

### 4.15. Hormones and Hormone Analogs

#### 4.15.1. 5-Androstenediol

5-androstenediol (5-AED, Neumune) is an endogenous weak androgen extensively studied as a potential MCM for ARS in rodents and NHPs [920,921,922,923,924,925,926,927]. In mice exposed to 3 Gy TBI, a 5-AED injection 1 h post-exposure improved IR-induced neutropenia and thrombocytopenia, promoting granulocyte/monocyte progenitor proliferation without affecting lymphocytes or erythrocytes [920]. A single SC injection (50 mg/kg) 1 day before 5 Gy γ-irradiation showed a transient therapeutic effect, while sequential post-irradiation doses provided more sustained myelosuppression mitigation [925] even at 9 Gy TBI [926].

The survival benefits of 5-AED are G-CSF-dependent, inducing multilineage BM progenitor reconstitution (neutrophils, erythroid, megakaryocytes) and improving infection resistance [920,928]. Additional mechanisms include cell cycle arrest, DNA damage reduction, and inhibition of AIM2 inflammasome-mediated pyroptosis [926,928]. Combined treatment with 5-AED and rhTPO resulted in a 20.1-fold increase in marrow CFU-S, demonstrating strong synergy compared to 3.7- and 3.1-fold increases with 5-AED or TPO alone [929].

In NHPs, post-irradiation 5-AED (15 mg/kg microparticle/NP formulations) after 4 Gy ^60^Co TBI reduced pancytopenia severity and duration, with 5-day IM or weekly SC regimens being most effective [923]. Deaths were primarily linked to opportunistic infections originating from gut and skin flora, progressing to sepsis or hemorrhagic sepsis [922,923]. In a second study with 6 Gy γ-irradiation, 5-AED reduced early IR-related mortality (12.5% vs. 32.5%) in NHPs, which were not provided with clinical support [924].

A double-blind RCT confirmed that 5-AED is safe and effective in enhancing innate immunity in healthy adults [930].

#### 4.15.2. GH and IGF-1

Growth hormone (GH) is a pituitary-derived hormone with central roles in somatic growth, metabolic regulation, and maintenance of tissue homeostasis. GH activates canonical JAK2/STAT5 signaling, leading to transcriptional regulation of multiple downstream targets, including induction of insulin-like growth factor 1 (IGF-1) production predominantly in the liver and, to a lesser extent, in peripheral tissues [931]. Many of the proliferative and anabolic actions of GH are mediated indirectly through IGF-1, whereas GH can also exert direct effects that are partially independent of IGF-1, particularly in metabolic regulation and tissue homeostasis. In this context, the well-established pro-survival, anti-apoptotic, and regenerative properties of the GH/IGF-1 axis provide mechanistic basis for its potential involvement in normal tissue recovery following IR exposure.

The clinical relevance of this axis in oncology is highlighted by the high incidence of GH deficiency and subsequent growth impairment following cranial radiotherapy, particularly in pediatric and adolescent populations [931]. In adults, radiation-induced GH deficiency presents more subtly, manifesting as reduced basal metabolism, decreased cardiac output, hypoglycemia, and impaired quality of life, highlighting the importance of early recognition when considering GH replacement therapy [931,932].

Beyond their role in hormone replacement, accumulating preclinical evidence indicates that both GH and IGF-1 can function as potent radiomitigators by limiting apoptosis and promoting the regeneration of IR-damaged tissues [377,643,933,934,935,936]. Post-irradiation administration of recombinant human GH significantly increased survival in lethally irradiated mice (60.7% vs. 10.7%) and NHP by enhancing hematopoietic and immune reconstitution [377,933]. In the context of HSCT, GH enhanced thymic cellularity and accelerated reconstitution of functional T and B lymphocytes without exacerbating graft-versus-host disease, supporting its immunorestorative capacity [937].

Beyond the hematopoietic system, GH exerts protective effects on radiation-sensitive epithelial tissues. Short-term GH administration mitigated IR-induced intestinal injury by enhancing epithelial proliferation, suppressing apoptosis, and downregulating p53 expression, while preserving the antitumor efficacy of RT [936,938,939]. Mechanistically, GH upregulates intestinal trefoil factor, a key peptide involved in mucosal protection and repair, thereby reducing radiation-induced intestinal mucositis [936]. GH also reduced the severity and delayed the onset of RID in experimental models, effects that were further enhanced when combined with zinc supplementation [643,940].

IGF-1 has been demonstrated to improve mice survival by attenuating both H-ARS and GI-ARS [934,941,942]. Mechanistic studies indicate that IGF-1 enhances DNA double-strand break repair through both NHEJ and HR pathways, while limiting IR-induced stem and progenitor cell apoptosis, thereby facilitating the recovery of radiation-damaged tissues [934,941,943,944,945]. Activation of mTORC1 signaling has been shown to be essential for IGF-1-induced crypt regeneration after IR exposure [946], while stimulating VEGF production promotes the angiogenesis necessary to ensure adequate vascular support for tissue repair and regeneration [935,947]. IGF-1 also restored salivary flow rates post-IR by maintaining aPKCζ activation and attenuating the inflammatory response involving macrophages and T cells [948,949].

Although GH and IGF-1 show promise in mitigating radiation-induced toxicity in vital organs, their clinical use remains controversial due to potential promotion of cancer recurrence and resistance to RT and CT [931,935,950]. Consequently, no clinical trials have yet assessed their radiomitigative potential in humans.

#### 4.15.3. Ghrelin

Ghrelin, commonly referred to as the hunger hormone, is primarily synthesized by P/D1 cells in the GI tract in response to starvation. Anti-inflammatory, anti-apoptotic, and regenerative actions appear to underlie ghrelin’s radiomitigative activity [70,951,952,953,954], although a contributory protective role mediated through stimulation of pituitary growth GH secretion cannot be excluded [70,951].

Preclinical studies demonstrate that ghrelin improves hematopoietic and GI outcomes following lethal IR exposure. In mice exposed to ^60^Co-γ-radiation (9.5 Gy), ghrelin treatment increased hemoglobin and hematocrit levels, prevented splenomegaly, and significantly improved survival [951]. Consistently, intraperitoneal ghrelin administration after TBI (10 Gy) reduced intestinal epithelial apoptosis, preserved gut barrier integrity, and decreased serum endotoxin levels and bacterial translocation, resulting in an improvement in 30-day survival from approximately 30% to 70% compared with irradiated controls [952,954]. Mechanistically, ghrelin promoted proliferative responses within intestinal crypts and restored the intestinal stem cell compartment through upregulation of the Notch target genes *Hes1* and *Olfm4* [954,955]. Ghrelin also normalized IR-induced oxidative and inflammatory responses, including dysregulated cytokine profiles and redox-sensitive NF-κB–AKT–MAPK signaling networks, thereby creating a permissive environment for epithelial repair and tissue homeostasis [70].

Importantly, ghrelin has also demonstrated efficacy in models of RCI, including radiation accompanied by sepsis, skin wounds, or burns. In these complex injury settings, ghrelin treatment significantly improved survival outcomes, mitigated body weight loss, and accelerated wound healing [70,951,956,957,958]. Moreover, ghrelin attenuated hematopoietic failure and splenocytopenia and restored leucocyte and thrombocyte accounts by sustaining G-CSF and keratinocyte chemoattractant levels [953]. Notably, combined therapy with pegylated G-CSF further enhanced radiomitigative efficacy by reducing platelet depletion and preventing brain hemorrhages induced by IR and RCI [957,958].

#### 4.15.4. Somatostatin Analogs: Octreotide and Pasireotide

Surgical reduction in pancreatic secretion attenuates acute mucosal injury and improves survival in irradiated dogs and rats, suggesting a key role of pancreatic enzymes in intestinal radiation toxicity [959,960]. Somatostatin and its synthetic analogs, including octreotide and octreotide acetate, inhibit pancreatic and GI secretions and slow intestinal transit, representing a promising strategy to attenuate IR-induced diarrhea [916,961]. Indeed, preclinical studies have demonstrated that octreotide conferred dose-dependent protection against delayed small bowel radiation toxicity, ameliorating RIF by reducing acute mucosal injury [916].

However, the short half-life of octreotide limits its practical application in mass-casualty scenarios, highlighting the potential of the more stable somatostatin analog SOM230 (pasireotide). Administration of SOM230 before or up to 4 h after TBI, with treatment continued for 14 days, significantly improved survival in mice with GI-ARS [961,962]. As SOM230 lacked direct cytoprotective effects on BM or intestinal tissues, its protective benefits were largely attributed to the reduction in proteolytic enzyme secretion into the intestine [961,962].

Octreotide alleviates diarrhea in patients with carcinoid syndrome and has received FDA approval for this indication [963]. However, its efficacy in managing acute diarrhea induced by RT or CRT remains clinically controversial [963,964,965]. Clinical studies have yielded conflicting results. SC octreotide outperformed conventional therapy (diphenoxylate plus atropine) in controlling acute diarrhea and reducing RT interruptions [964]. However, results remain inconsistent, as another randomized trial (NCT00033605) found that octreotide administered SC or IM did not improve pelvic RT-related diarrhea and even worsened some GI adverse effects. [963]. While IM octreotide acetate failed to reduce the incidence or severity of diarrhea in anorectal cancer patients undergoing CRT (NCT00075868) [965]. Moreover, although a meta-analysis by Ma et al. concluded that octreotide is superior to conventional therapy for CRT-induced diarrhea [966], it cannot be considered an effective prophylactic measure [963,967].

#### 4.15.5. Melatonin

Melatonin (N-acetyl-5-methoxytryptamine) is a hormone primarily synthesized by the pineal gland, classically associated with circadian timing and sleep regulation [968]. Beyond its physiological roles, melatonin exerts potent radioprotective and radiomitigative effects by scavenging ROS and RNS, enhancing antioxidant enzymes such as SOD, GPx, and GR, and inhibiting pro-oxidant pathways [969,970,971]. In parallel, it enhances DNA repair by activating BER and NER pathways, thereby improving genomic stability and protecting normal tissues from IR-induced apoptosis [969,970,971,972,973,974,975]. Melatonin-mediated attenuation of oxidative stress and apoptosis underlies tissue preservation following IR, including protection of gonadal function [976,977], mitigation of brain edema, necrosis, neuronal degeneration [978], and prevention of cataractogenesis [979]. Consistently, post-irradiation administration of melatonin mitigated myelosuppression in rodent models by restoring BM and splenic hematopoiesis and normalizing peripheral blood counts, particularly platelets [971,980], and preserving intestinal mucosal integrity in both the small and large intestine [971,973,981]. In experimental models of RILI, melatonin attenuated both acute and late pulmonary damage by reducing oxidative stress-mediated vascular and alveolar injury, in part through downregulation of pro-oxidant enzymes such as NOX1, NOX2, NOX4, Duox1, and Duox2 [982] and by suppressing the NLRP3-mediated inflammatory response involved in histopathological tissue injury [983]. In line with these findings, inhibition of NF-κB/NLRP3 signaling has been identified as a key mechanism underlying melatonin-mediated attenuation of RIOM and GI-ARS [973,984]. Collectively, melatonin-mediated mitigation of H-ARS and GI-ARS, together with improved survival in irradiated animals, highlights its potential utility in radiation emergencies, particularly given its efficacy when administered after radiation exposure [971,985,986,987,988]. Despite compelling preclinical evidence, clinical data remain limited and heterogeneous (Table 8).

Oral melatonin, administered prior to abdominal–pelvic CT, significantly reduced γ-H2AX foci, indicating decreased DNA DSBs [972], but failed to prevent lymphopenia or pancytopenia in cancer patients undergoing RT or CRT [990,995]. Regardless of administration timing, melatonin attenuated IR-induced histological damage to the parotid and submandibular glands in rats [998], and its protective effects on RIOM progression have been confirmed in HNC patients [992,993], particularly when applied topically as a mucoadhesive oral gel [994]. Similarly, topical melatonin reduced the incidence and severity of RID in early breast cancer trials [991], although more recent studies reported only modest or delayed protective effects [997].

Cancer diagnosis and treatment are frequently associated with psychological stress, sleep disruption, and reduced endogenous melatonin production [999,1000]. Although some discrepancies exist [1001], several RCTs indicate that melatonin supplementation during RT reduces fatigue, anxiety, and depression, while improving QoL in breast cancer patients [999,1000], which may indirectly enhance treatment tolerance and OS.

Importantly, melatonin combines radioprotection of normal tissues with antitumor activity, reinforcing its appeal as an adjuvant in cancer therapy [988,1002,1003]. These dual effects may partly explain the improved responses to CT and RT, as well as increased OS observed in selected aggressive malignancies, including GB, pancreatic, lung, breast, and prostate cancers [989,996,1004,1005,1006]. Consistent with this profile, melatonin has generally been well tolerated in patients receiving RT and/or CT, with predominantly mild adverse effects. Clinical evidence does not indicate that melatonin compromises CT or RCT efficacy; oncologic outcomes have been neutral or, in some cases, favorable, although trials are frequently small and heterogeneous [1004,1007]. Nevertheless, recent analyses suggest that these benefits may be context-dependent or limited to specific clinical settings [1002,1003], underscoring the need for optimized dosing strategies and validation in larger, well-designed prospective RCTs.

### 4.16. Metformin

Metformin (1,1-dimethylbiguanide hydrochloride) is the first-line therapy for type 2 diabetes and has attracted considerable interest as a potential radiomitigator. By inhibiting mitochondrial complex I, it reduces mitochondrial O_2_^−•^ production and ATP synthesis, leading to AMPK activation and subsequent induction of endogenous antioxidant enzymes, including GPx, SOD, CAT, and TrxR [1008,1009]. These antioxidant effects, together with AMPK-dependent upregulation of DNA repair pathways, are thought to play a central role in metformin radiomitigative activity [1010,1011,1012,1013,1014,1015].

Consistent with these mechanisms, metformin attenuated TBI-induced BM injury by inhibiting HSC senescence, thereby mitigating long-term HSC damage [1008]. In murine models, metformin increased survival when administered either prior to 6 or 8 Gy TBI [1016] or shortly after 8 Gy TBI [1010]. Moreover, when combined with NAC, MESNA, or captopril, metformin enhanced survival by 2.6-, 2.8-, and 2.4-fold, respectively, effects comparable to or better than those achieved with a single prophylactic dose of amifostine [1017].

Post-irradiation administration of metformin effectively attenuated radiation enteropathy [888,1012,1018], RILI and RILF [982,1019], as well as RID and radiation-induced skin fibrosis [1014,1020,1021]. Notably, combined treatment with pravastatin produced a more pronounced attenuation of acute intestinal injury in both mouse and minipig models [888]. Early post-irradiation treatment with metformin or adipose-derived stem cells prevented fibrosis, whereas their combination offered no additional benefit. In contrast, once fibrosis was established, the combined therapy demonstrated stronger antifibrotic and anti-inflammatory effects [1014]. Metformin has also been shown to preserve salivary gland structure and function after irradiation by reducing IR-induced DNA damage and inflammation [161], and to promote neural precursor regeneration and improve cognitive recovery following cranial irradiation in both preclinical models and a pilot clinical trial (NCT02040376) in pediatric brain tumor survivors [1022].

The widespread clinical use of metformin has enabled retrospective analyses of its potential radioprotective effects in humans [1023,1024,1025]. In breast cancer patients, metformin use was associated with a reduced risk of radiation-induced cardiac toxicity [1025], while improved OS was reported in hepatocellular carcinoma patients treated with RT [1023]. Metformin also contributed to the maintenance of blood cell counts in thyroid cancer patients undergoing ^131^I therapy [1024] and reduced acute urinary complications of RT in prostate cancer patients (IRCT20211213053377N1) [1026], but failed to prevent RT-related adverse effects in several clinical trials and retrospective studies (NCT01996696 and NCT02115464) [1027,1028].

Metformin induces apoptosis in cancer cells, attenuates the inflammatory response within the tumor microenvironment, and improves T-cell function, thereby sensitizing cancer cells to CR and RT. In addition, its metabolic effects, including reductions in glucose availability, ATP production, and insulin levels, further contribute to the inhibition of tumor growth [1011,1029,1030]. Across CRT/RT oncology trials, clinical evidence generally shows no signal that metformin compromises efficacy, with several studies reporting neutral outcomes (no survival benefit) and acceptable tolerability [1027,1031]. However, in OCOG-ALMERA randomized clinical trial (LA-NSCLC), metformin was associated with higher severe toxicity and inferior efficacy, and the investigators suggested toxicity may have limited CRT dose delivery, providing a plausible mechanism for reduced treatment success [1028]. Therefore, larger prospective clinical studies are required to define optimal dosing regimens and therapeutic windows to fully establish metformin’s radiomitigative and radiosensitizing effects.

### 4.17. Toll-like Receptor Agonists

TLRs play a central role in innate and adaptive immune responses and have been shown to contribute to basal resistance against IR–induced injury [1032]. TLR5 agonists such as entolimod and KMRC011 have demonstrated potent radioprotective and radiomitigative effects by suppressing apoptosis and fibrosis through modulation of oxidative stress and regulation of pro- and anti-apoptotic pathways, primarily via ERK1/2- and MKP-7-dependent signaling mechanisms [1033,1034,1035]. Entolimod (CBLB502), a flagellin-derived TLR5 agonist, activates a broad transcriptional program of downstream TLR5-dependent effectors, including hematopoietic cytokines (G-CSF, IL-6, IL-8), anti-inflammatory mediators (IL-10), antioxidant enzymes (SOD2), anti-apoptotic factors (IAPs and Bcl-2), MMP-9, and antimicrobial peptides, which collectively mediate its radioprotective and radiomitigative activity [1033,1036,1037,1038,1039,1040,1041]. A single injection conferred significant protection against both GI- and H-ARS in mice and NHPs, leading to improved survival in the absence of intensive supportive care [1036,1038,1041]. Following exposure to LD_50–70/40_ of TBI, entolimod promoted morphological and functional recovery of hematopoietic and immune organs, mitigated the severity and duration of pancytopenia, and concurrently reduced apoptosis while enhancing intestinal crypt regeneration in NHP [1036,1038]. In addition, it ameliorated RILI and RILF, protected against IR-induced testicular damage, and attenuated RIOM and RID in murine models [1033,1034,1042]. Beyond normal tissue protection, entolimod has also been shown to enhance the tumoricidal efficacy of RT in mouse cancer models, suggesting that TLR5-based strategies may improve therapeutic outcomes in oncology settings [1043]. Although entolimod was generally well tolerated in healthy volunteers, transient elevations in serum transaminases observed in clinical studies may pose challenges for its further clinical development [1044].

KMRC011 is being developed in Korea as radiation MCM [1045]. In mice exposed to lethal TBI (11 Gy), KMRC011 markedly improved survival from 10% in controls to up to 90% with repeated dosing, primarily by mitigating intestinal injury which was associated with suppression of NF-κB and NLRP3 signaling, reduced apoptosis, and upregulation of genes involved in DNA repair and antioxidant defense, including Rad21, Gadd45b, Sod2, and Irg1 [1035]. Consistent with the U.S. FDA Animal Rule, which requires efficacy to be demonstrated in more than one animal species, a single dose of KMRC011 administered within 25 h after TBI (5.5 Gy) significantly reduced mortality in monkeys (80% vs. 40%) [1045] and contributed to attenuate RIOM in beagle dogs [1046]. Translational relevance was further supported by a first-in-human study (NCT03585803) demonstrating good tolerability and dose-dependent increases in G-CSF, IL-6, and absolute neutrophil counts, consistent with its potential role in ARS prevention [1047].

In addition to TLR5 agonists, other TLR-targeting strategies have shown promise as radiomitigators. Pre-irradiation administration of the TLR9 agonist CpG preserved body weight, maintained stool consistency, and significantly improved survival after 8.4, 9.4, and 10.4 Gy TBI (*p* < 0.002, *p* < 0.008, and *p* < 0.03, respectively). Post-irradiation administration conferred limited radiomitigation, with survival benefit observed only at 8.4 Gy (*p* < 0.01) [1048]. Notably, CpG reduced apoptosis, promoted rapid restitution of intestinal villi, and increased the therapeutic ratio of abdominal irradiation in a colorectal cancer xenograft model, suggesting potential applicability in combined RT settings [1048].

Compared with TLR5 (flagellin), TLR4 (MPLA, FG-4592), and TLR9 agonists, FSL-1 showed superior efficacy in enhancing hematopoietic recovery in preclinical models [1049]. Post-IR (24 h) SC administration of FSL-1 substantially reduced radiation lethality across multiple mouse strains, age groups, and sexes, as well as in NHPs [1049,1050,1051]. Its radiomitigative effects are mediated through MyD88-dependent signaling and a robust induction of systemic G-CSF, leading to accelerated hematopoietic cell proliferation and mobilization [1049,1050]. While FSL-1 administration in mice promotes recovery of all major blood cell lineages [1049], in NHPs exposed to sublethal radiation it primarily enhances hematocrit recovery [1050]. Importantly, no significant toxic effects have been reported in animal models, and FSL-1 has the notable advantage of being effective after a single SC dose administered either 24 h before or after irradiation [1049,1050,1051].

Despite substantial preclinical evidence, advancing TLR agonists as radiation MCM requires further investigation, as prolonged or excessive TLR activation may promote inflammatory responses that contribute to the development of DEARE [50,1032].

### 4.18. Polyphenols

Polyphenols are plant-derived secondary metabolites characterized by the presence of phenolic (aromatic) rings, substituted with one or more hydroxyl groups, often linked through carbon–carbon or carbon–oxygen bridges to form diverse chemical classes, such as flavonoids (apigenin, daidzein, genistein, epicatechin, epigallocatechin-3-gallate(EGCG), hesperidin, quercetin, anthocyanidins), phenolic acids (caffeic and ferulic acids), curcuminoids (curcumin) stilbenes (resveratrol and pterostilbene), and lignans [1052]. Polyphenols confer radioprotective effects through multifaceted mechanisms, including direct scavenging of free radicals, metal ion chelation, and regeneration of endogenous antioxidant systems [1053]. Furthermore, they modulate key cellular pathways involved in DNA damage repair, inflammation (e.g., NF-κB signaling), and cell death, thereby enhancing cellular resilience to IR [37,1054,1055,1056,1057,1058,1059]. Due to their broad range of therapeutic actions, polyphenols have been extensively studied as promising radiomitigators and/or radioprotectors in clinical trials (Table 9).

#### 4.18.1. Isoflavones

Soybean isoflavones, particularly genistein, daidzein, and glycitein, exert radiomitigative effects by reducing early endothelial cell death and suppressing inflammatory signaling. These actions limit macrophage and neutrophil recruitment and activation in the lungs, thereby attenuating radiation-induced pneumonitis and subsequent fibrosis [1057,1098,1099,1100]. They also protect mice against radiation-induced heart injury [1101], prevent skin damage and hair loss [1102], and reduce the severity of radiation-related esophagitis by mitigating basal cell hypertrophy in the mucosal epithelium and smooth muscle injury [1103]. Soy isoflavones have been found to sensitize prostate, renal, and lung cancer cells to RT, while reducing collateral toxicity [1098,1099]. Prostate cancer patients supplemented with isoflavones during and after RT showed greater reductions in prostate-specific antigen (PSA) levels and a decreased incidence of RT-related adverse events affecting urinary, GI, and erectile function compared with placebo-treated patients [1097]. Most radioprotective effects of isoflavones are primarily attributed to genistein, a phytoestrogen with high affinity for estrogen receptor β (ER-β) [493,1104,1105,1106,1107,1108]. By increasing G-CSF levels at 4 and 24 h after sham or γ-irradiation, genistein accelerated neutrophil and platelet recovery and improved survival (97% vs. 31% in controls) [1055,1105], with enhanced efficacy when co-administered with captopril [853].

Genistein, the major isoflavone present in soybeans, ameliorated intestinal morphological changes, such as decreased crypt survival, villus shortening, and increased basal lamina thickness 3.5 days after a 10 Gy irradiation, while simultaneously inhibiting CT26 tumor growth [1107]. Post-irradiation genistein administration delayed onset and reduced the severity of radiation pneumonitis, improving mouse survival by suppressing TNF-α, IL-1β, and TGF-β levels, and reducing oxidative stress markers and collagen deposition [493,1104]. To address genistein’s limited solubility and bioavailability, Humanetics Pharmaceuticals formulated BIO 300^®^, a synthetic nanosuspension developed as a radiation medical countermeasure, available as an injectable (BIO 300^®^ IS) or oral (BIO 300^®^ OS) formulations. Notably, oral BIO 300^®^ IS provided radioprotection against H-ARS in mice comparable to Neulasta^®^ but was ineffective when administered as a single dose after radiation exposure [1108,1109]. When administered 2 h after IR, BIO 300^®^ mitigated radiation-induced erectile dysfunction while maintaining the efficacy of RT on prostate cancer xenografts [1110]. At 400 mg/kg/day for 4–6 weeks, starting 24 h after 11–12.5 Gy WTI, BIO 300^®^ OS improved survival and reduced pneumonitis and fibrosis morbidity, primarily by attenuating the acute inflammatory response to irradiation. Treated animals also maintained body weight more effectively, particularly when the radiation field included the GI tract, while tumor xenografts were radiosensitized [1111]. Based on promising results in murine models, BIO 300^®^ is advancing to NHP studies, showing efficacy when given prophylactically to mitigate ARS [1112,1113].

#### 4.18.2. Epigallocatechin-3-gallate

EGCG, a major green tea flavonol, attenuates IR-induced damage through multiple mechanisms, including free radical scavenging, activation of Nrf2-dependent antioxidant pathways, reduction of mitochondrial dysfunction and apoptosis, and promotion of DNA repair processes [1053,1059,1114]. Notably, it displays stronger antioxidant activity than resveratrol and vitamins C and E and has been shown to mitigate hematopoietic radiation injury by preserving bone marrow colony-forming capacity in experimental models [1114,1115]. In lethally irradiated mice, EGCG preserved intestinal stem cell regeneration by scavenging ROS and inhibiting apoptosis and ferroptosis, thereby mitigating intestinal damage, restoring body weight, and improving survival [1059,1114]. Its anti-inflammatory and microbiota-modulating properties have also been shown to be essential in the attenuation of radiation enteritis [1116]. Compared with dexamethasone, EGCG was more effective in attenuating RILI, preventing RILF, and significantly improving survival rates [1117]. Self-assembled EGCG NPs showed enhanced cellular uptake, superior ROS scavenging, and greater DNA protection than free EGCG and were more effective than amifostine in attenuating IR-induced damage, accelerating wound healing, and preserving tissue integrity [1118].

More importantly, clinical trials have shown that EGCG mitigates a range of IR-induced toxicities, including OM [1090], enteritis [1091,1092], dermatitis [1087,1088] and can even reverse IR-induced skin ulcers (grade 3 RID) [1089]. Prophylactic administration of EGCG significantly reduced IR-induced esophagitis, pain, nausea, and dysphagia in patients with lung or esophageal cancer undergoing RT or CRT [1083,1084,1085,1086], while therapeutic administration was less effective [1084,1085]. A phase II double-blind trial (NCT06875791) evaluating the safety and efficacy of TV5M01 (5% green tea extract + 0.1% morphine gel) for RIOM in HNC patients is ongoing. Two additional trials are currently enrolling participants to assess catechins for RID (NCT07149506) and for the prevention of RT-induced dysphagia (NCT06398405).

#### 4.18.3. Grape Seed Proanthocyanidins

Grape seed proanthocyanidins (GSPs), the main components of grape seed extracts, scavenge free radicals and chelate metals via their o-diphenol groups, providing greater protection against oxidative stress than vitamins C, E, or β-carotene [1119]. Their radioprotective/radiomitigative effects have been confirmed across multiple tissue types, including BM, testes, intestines, lungs, and eyes [190,1120,1121,1122]. Supplementation with oligomeric proanthocyanidins reduced ROS levels and increased the activities of xanthine dehydrogenase, SOD, GPx, and CAT, contributing to the attenuation of IR-induced intra-retinal hemorrhages, corneal swelling, and epithelial necrosis [1120]. Pre- and/or post-TBI (8 Gy) administration of GSPs accelerated the recovery of white blood cell counts and significantly improved mouse survival [1121]. Suppression of the MAPK pathway is involved in the radiomitigative effects of GSPs in the lung, testes, and intestine [190,1122], which at the same time radiosensitizes lung cancer cells [190].

RayGel^®^ (a topical formulation containing GSH and anthocyanins) provided skin protection and reduced discomfort, facilitating more consistent completion of RT courses in breast cancer patients [1060], whereas oral GSP extract showed no benefit for pain or breast fibrosis [1061].

#### 4.18.4. Curcumin

Curcumin (CURC), the main polyphenol extracted from turmeric (*Curcuma longa*), has demonstrated radioprotective and radiomitigative effects by reducing oxidative stress, apoptosis, inflammation, and fibrogenesis [1056,1123,1124,1125,1126,1127]. Consistent with these effects, CURC markedly reduced post-irradiation cataractogenesis, lowering its incidence from 100% to 40% [1056]. Like resveratrol, silibinin, and EGCG, CURC significantly inhibits IR-induced activation of p53- and MAPK-mediated death signaling, thereby preventing IR-induced apoptosis in normal cells [350].

Nevertheless, the clinical translation of CURC is hindered by poor solubility, limited absorption, and rapid metabolic clearance, highlighting the need for advanced delivery systems, particularly CURC-based nanocarriers, to enhance bioavailability and optimize radiomitigative efficacy [1127,1128,1129]. Both free CURC and CURC-loaded nanoparticles upregulate peroxisome proliferator-activated receptor gamma (PPAR-γ) while suppressing NF-κB signaling and TGF-β production, thereby attenuating IR-induced inflammation and fibrosis in murine models of hepatic, renal, cardiac, and pulmonary injury [1125,1130,1131,1132,1133,1134,1135]. Consistent with these findings, CURC nanomicelles significantly mitigated small intestinal injury in mice exposed to 7 Gy TBI [1127], and ROS-responsive CURC-inulin micelles ameliorated radiation-induced enteritis in mice, both attenuating inflammation and preserving gut microbiota [1128].

CURC, administered orally or topically, accelerated wound healing after IR exposure by enhancing re-epithelialization and collagen synthesis in both mouse and minipig models [1124,1136]. Clinically, although an initial pilot study suggested reduced RID severity with prophylactic oral CURC [1075], these findings were not confirmed in subsequent larger trials [1076,1078]. Topical CURC formulations produced only modest reductions in skin reactions and pain without significant clinical benefit [173,1079], whereas Vicco^®^, a cream containing turmeric and sandalwood oil, applied during and after RT, significantly reduced the incidence and severity of RID and RIOM in breast and HNC patients, respectively [1067,1077].

Supported by preclinical studies [1137], CURC, nanoCURC, and turmeric CURC-based formulations (mouthwash, oral solutions, capsules, creams, pastes, and gels) have demonstrated good tolerability and efficacy in alleviating RIOM severity in HNC patients [584,585,1062,1064,1065,1066,1069,1070,1071,1072,1073,1074]. Nanomicellar formulations, such as SinaCurcumin^®^, improved bioavailability and reduced RIOM incidence and severity [1070,1073,1074]. Treated patients also experienced less pain, reduced weight loss, and higher compliance with RT/CRT [1064,1070,1072,1073,1074], which may result not only from faster tissue healing but also from higher CURC doses enhancing monoamine release (serotonin, dopamine, norepinephrine), thereby reducing nociceptive signaling and improving morphine efficacy [1074,1138]. The consistency of clinical trial results is supported by recent systematic reviews and meta-analyses, which provide moderate to strong evidence that turmeric and CURC, in various formulations, delay the onset and reduce the severity of RIOM [1139,1140,1141].

In prostate cancer patients treated with RT, CURC boosted total antioxidant capacity and relieved urinary symptoms (frequency and urgency) without affecting PSA/MRI outcomes [1080] whereas nanoCURC had minimal impact on the prevention of RT-induced proctitis [1081]. Through the inhibition of NF-κB and MAPK signaling pathways, CURC suppresses carcinogenesis, angiogenesis, tumor progression, and metastasis, reinforcing CURC’s dual promise as both an antitumoral and a radioprotective/radiomitigative agent in oncologic settings [350,1126,1132].

#### 4.18.5. Silymarin

Silymarin, a flavonolignan extract from *Silybum marianum*, has demonstrated radiomitigative potential, largely through modulation of oxidative stress, inflammation, and apoptosis via inhibition of NF-κB-dependent signaling [1142,1143]. Preclinical studies have shown that silymarin attenuates IR-induced injury in hematopoietic tissues, liver, kidneys, and testes, contributing to improved tissue integrity and functional recovery following irradiation [1135,1144,1145,1146]. Notably, the efficacy of silymarin in attenuating H-ARS was further enhanced when combined with IV administration of MSCs one day after a γ-irradiation (4 Gy) in male rats, suggesting a synergistic interaction between antioxidant and regenerative mechanisms [1147]. CUR and silymarin significantly mitigated nephrotoxicity in rats exposed to 8 Gy γ-irradiation by reducing oxidative and inflammatory damage, improving renal function, and restoring tissue architecture, with additive protective effects observed following combined treatment [1135]. In clinical studies, topical application of silymarin-based cream or gel delayed the onset and reduced the severity of RID [1094,1095], while oral administration initiated at the start of RT and continued for six weeks significantly slowed the progression of RIOM in patients with HNC [1096].

In a TBI model exposed to a LD_50/30_ dose (7.2 Gy), silibinin, the major bioactive component of silymarin, in combination with pterostilbene achieved 100% survival at 30 days and 70% at 60 days, clearly outperforming either compound alone and other pterostilbene-based combinations, including those with EGCG, CURC, genistein, quercetin, gallic acid, naringenin, delphinidin, phloridzin, or luteolin [678]. Consistently, by suppressing inflammatory responses and TGF-β1/Smad2/3 signaling, silibinin mitigated radiation-induced intestinal fibrosis [1148] and was also effective in attenuating IR-induced pneumonitis and fibrosis, improving survival from 50% to 100% in mice exposed to 13 Gy TBI [1142].

#### 4.18.6. Resveratrol

Resveratrol exhibits robust antioxidant and anti-clastogenic properties that contribute to genomic stability and cancer prevention [1149], and preclinical evidence indicates that it also exerts radiomitigative effects against both H-ARS and GI-ARS [1150,1151,1152,1153]. In the GI tract, resveratrol attenuated radiation-induced mucosal inflammation and structural damage, including villus shortening and injury to goblet cells and intestinal glands [1151,1152,1153]. Notably, delivery of resveratrol via polymeric nanoparticles significantly improved survival following TBI (7.2 Gy, X-rays), suggesting enhanced bioavailability may potentiate its radiomitigative efficacy [1153]. Mechanistically, protection against GI-ARS has been associated with direct antioxidant activity, reinforcement of endogenous antioxidant defenses, activation of SIRT1/FOXO3a signaling, and inhibition of PI3K/Akt/mTOR pathways, collectively reducing oxidative stress and pro-inflammatory mediator production in irradiated intestinal tissues [1058,1152,1154,1155].

Resveratrol preserved the ovarian follicle pool and restored ovarian function through upregulation of PPAR-γ and SIRT1 expression [1156], and it attenuated IR-induced pneumonitis and RILF by suppressing NF-κB-mediated pulmonary inflammation and immune-cell infiltration [1157,1158]. Its radiomitigative effects were further enhanced by coadministration with alpha-lipoic acid [1158], a potent antioxidant with established radioprotective properties [1159]. Notably, both compounds have also been shown to inhibit RAS, which may contribute to their radiomitigative effects [1158].

However, resveratrol’s short biological half-life limits its clinical effectiveness, and only a small study has reported protective effects against RID [1093]. In this regard, pterostilbene, a methoxylated resveratrol analog with higher bioavailability, has shown superior efficacy in improving post-irradiation survival in mice [678,1160].

Given their low toxicity and pleiotropic biological effects, polyphenols represent attractive candidates for inclusion in combination therapies aimed at preventing ARS and DEARE. For example, pterostilbene and silibinin upregulate Nrf2-related antioxidant defenses and improve survival in mice exposed to 7.2 Gy γ-TBI (LD_50/30_) [678]. Despite outperforming other polyphenol combinations, they failed to achieve long-term survival. In contrast, their combination with two radiomitigators, NR and FSL-1 (single IP dose), conferred 100% long-term survival after lethal γ-irradiation, without compromising RT efficacy in murine cancer models [678]. In addition to demonstrating the greater efficacy of combined strategies, these findings highlight the need for caution when assigning radioprotective or radiomitigative efficacy based solely on short-term survival outcomes.

### 4.19. Probiotics, Prebiotics, Synbiotics and Fecal Microbiota Transplantation

RT can disrupt the skin, pulmonary, oral, and GI microbiomes, leading to a loss of beneficial species and an overgrowth of potentially harmful bacteria [1161,1162,1163]. Such dysbiosis is directly linked to RT-related adverse effects, particularly to the severity of IR-induced enteritis [1162,1164,1165]. In light of this evidence, dietary supplementation with probiotics, prebiotics, and synbiotics, as well as fecal microbiota transplantation (FMT), is being investigated in preclinical and clinical studies (Table 10) as a means to mitigate IR-induced damage [1163,1166].

#### 4.19.1. Probiotics

Probiotics benefit patients undergoing RT by competing with pathogenic or opportunistic microorganisms, enhancing the production of short-chain fatty acids (SCFAs) such as butyrate and propionate, promoting epithelial regeneration, and modulating immune responses [1169,1170,1171,1173,1200,1201]. The majority of preclinical and clinical investigations aimed at preventing IR-induced enteritis have focused primarily on the benefits of *Lactobacillus* and *Bifidobacterium* probiotics [1162,1165,1168,1175,1178,1179,1190,1192,1202,1203,1204,1205]. Continuous intragastric administration of a probiotic cocktail from these genera during acute radiation enteritis partially protected the intestinal epithelium, promoted crypt cell proliferation, reduced weight loss, and improved survival in mice exposed to 9 Gy TBI [1162]. *Lactobacillus plantarum* decreased IR-induced inflammation and improved colonic anastomotic repair by promoting collagen deposition [1202,1205]. Lipoteichoic acid from *Lactobacillus rhamnosus* GG activates macrophage TLR2, inducing CXCL12 secretion that inhibits epithelial stem cell apoptosis and promotes crypt regeneration, thereby protecting the intestine from IR-induced damage [1206]. Unfortunately, clinical trial data assessing this probiotic for the prevention of CRT-related side effects (NCT01790035) are still unavailable.

Polyphenols also promote the expansion of health-associated bacteria while limiting the growth of pathogenic taxa [1116]. Consistent with this concept, EGCG partially restored the Firmicutes/Bacteroidetes ratio disrupted by IR, thereby reducing DNA damage, apoptosis, and ferroptosis in Ki67^+^ and Lgr5^+^ intestinal stem cells and ultimately prolonging survival in lethally irradiated mice [1059,1116].

Metformin reshaped the microbiota, increasing *Lactobacillus* abundance in patients undergoing abdominal RT, a shift that correlated with reduced diarrhea [1018]. This clinical observation aligns with mechanistic evidence showing that *Lactobacillus plantarum* and metformin directly activate FXR signaling, upregulate tight-junction proteins and mucins, expand goblet-cell populations, and thicken the mucus layer, thereby reinforcing epithelial barrier integrity [1018,1205]. IR-induced reductions in the commensal bacterium *Akkermansia muciniphila* (*A. muciniphila*) have also been inversely correlated with diarrhea duration in both preclinical and clinical settings [1207,1208]. *A. muciniphila* supplementation restored intestinal epithelial integrity and improved survival in irradiated mice by secreting propionic acid, which activated GPR43 signaling, resulting in enhanced tight-junction protein expression and mucin production [1208]. Similarly, metformin and several polyphenols (e.g., EGCG, CURC, and resveratrol) protected intestinal barrier function by increasing the relative abundance of *A. muciniphila* [1208,1209,1210].

Importantly, results from multiple RCTs suggest that probiotic supplementation, particularly formulations containing *Lactobacillus* and *Bifidobacterium* species, may reduce the incidence and severity of radiation enteritis [1176,1177,1179,1180,1181,1182,1183,1192], a finding further supported by recent meta-analytic evidence [1211]. Moreover, beyond preserving mucosal morphology, probiotic administration also mitigated neuronal apoptosis and inflammation in mice exposed to sublethal TBI [1212], suggesting a broader radioprotective potential mediated through the gut–brain axis.

Interleukin-22 (IL-22) is an anti-inflammatory cytokine, mainly produced by intestinal resident lymphocytes, that supports mucosal healing by stabilizing Paneth cells and Lgr5^+^ intestinal stem cells. Systemic administration of IL-22 rapidly restores BM function and protects against intestinal crypt damage caused by abdominal irradiation, underscoring the importance of maintaining IL-22 levels in environments where intestinal resident lymphocytes are severely affected [1213,1214]. Engineered probiotics such as *Lactobacillus reuteri* expressing IL-22 (LR-IL-22) markedly enhanced epithelial barrier integrity and improved survival rates in mice subjected to TBI (9.5 Gy) and whole-abdomen irradiation (19.75 Gy) [1204,1213,1214]. Notably, these benefits were observed even when LR-IL-22 was administered 24–72 h post-irradiation, with optimal survival seen at 24–48 h [1213]. LR-IL-22 also reduced multiple biomarkers of intestinal, immune, and BM injury [1204] and modestly improved survival in an ovarian cancer model subjected to 15 Gy abdominal irradiation [1214].

Radiation disrupts the oral microbiota directly and indirectly through hyposalivation and mucosal injury. Probiotic administration attenuates RIOM through multiple complementary mechanisms, including competitive inhibition of oral pathogens, reinforcement of mucosal barrier function, and suppression of pro-inflammatory cytokines implicated in epithelial apoptosis and ulceration [1171,1173,1196,1215]. In addition, probiotics enhance mucosal defense by promoting the production of antimicrobial peptides (e.g., lysozyme and IgA) and salivary glycoproteins while attenuating the radiation-induced decline in circulating CD3^+^, CD4^+^, and CD8^+^ T-cell populations [1169,1170]. As shown in Table 10, several clinical trials in HNC demonstrate that probiotic supplementation alleviates RIOM severity and improves patient tolerance to CRT, with beneficial effects reported for multiple *Bifidobacterium* and *Lactobacillus* spp. [1167,1169,1170,1171,1175]. Oral probiotic supplementation reduced oral *Candida* spp. counts, a key finding since candidiasis is the most common opportunistic infection in HNC patients receiving RT [1171]. *Bacillus clausii* UBBC07 spores have also been shown to be highly effective in preventing high-grade RIOM [1172] compared with other clinical studies with *Lactobacillus brevis* CD2 lozenges [1167,1168]. Recently, topical application of *Streptococcus salivarius K12* (SsK12) and K12@Lip@GSH (SsK12 encapsulated in liposomes functionalized with GSH) restored oral microbial balance, promoted epithelial regeneration, and reduced RIOM severity in mouse models [1174,1216], findings that have been corroborated in HNC patients treated with CRT [1173,1174]. In addition, SsK12 administration significantly reduced the population of Gram-negative bacteria (*Selenomonas* and *Acinetobacter*) involved in the progression of OM and the development of opportunistic infections during RT [1173]. Two ongoing RCTs in recruiting phase (NCT06285591, NCT06390176) are evaluating the effectiveness of *Lactobacillus reuteri* and *Lactobacillus rhamnosus* for RIOM in HNC patients undergoing CRT.

Collectively, these findings underscore that correcting post-irradiation dysbiosis and preserving microbial homeostasis represent promising and effective approaches to mitigating IR-induced tissue damage. Probiotics comprising multiple strains and species generally outperform single-strain formulations, likely due to complementary metabolic and immunological mechanisms. Despite robust preclinical and clinical data, no probiotic is currently FDA-approved for attenuating IR-induced damage, underscoring the need for better strain characterization, dose/administration optimization, and larger, rigorously designed clinical trials.

#### 4.19.2. Prebiotics

Prebiotics are typically non-digestible carbohydrates (such as oligosaccharides, resistant starch, inulin, and pectin) that can be metabolized by the gut microbiota, enhancing its maintenance and diversity and serving as precursors for SCFA production [1217,1218,1219]. Preclinical studies have shown that certain prebiotics, such as mannan-oligosaccharides, can significantly improve survival in irradiated animals by preserving intestinal structure and supporting the recovery of blood-forming tissues [1219,1220]. More specifically, konjac glucomannan slightly outperformed amifostine in inhibiting epithelial apoptosis, promoting crypt regeneration, and preserving gut permeability, with the notable advantage of supporting intestinal microbiota and SCFA production [1219].

Pectin, a highly fermentable soluble dietary fiber found in fruits and vegetables, prevents IR-induced damage to intestinal stem cells, facilitates crypt regeneration, and ultimately promotes survival in mice [1221]. It also reduced radiation-induced EMT and intestinal fibrosis possibly by modulating gut microbiota composition and SCFA production [1217]. In an immunodeficient mouse model, psyllium (a soluble fiber) plus inulin slowed tumor growth and significantly reduced IR-induced intestinal toxicity [1222], which is in agreement with the results of a trial in which Metamucil (a psyllium bulking agent) significantly decreased the incidence and severity of diarrhea in cancer patients subjected to pelvic RT [1184].

Emerging clinical evidence (Table 10) suggests that dietary fiber supplementation may help restore the gut microbiota [1161,1188] and mitigate pelvic RT-related adverse effects [1161,1184,1185,1191]. In addition to attenuating RT-induced diarrhea, enteral supplementation with Gln and prebiotics was associated with reduced weight loss, prevention of *Enterococcus* spp. translocation, and improved 100-day survival (100% vs. 77.3% in controls) in HSCT patients [1223]. However, other studies report heterogeneous [1187] or inconclusive results [1186], and evidence on the optimal timing of fiber intervention (before or during RT) remains limited, highlighting the need for more robust trials before adoption in clinical practice [1218].

#### 4.19.3. Synbiotics

Clinical data on the use of synbiotics for the prevention of radiation-induced damage remain limited but suggest a protective effect on the mucosa through the reduction in inflammation (Table 10) [1191,1192,1193,1195,1196,1198,1199,1224]. Synbiotic administration prevents rectal inflammation and improves QoL in cancer patients subjected to pelvic RT [1193], reduces dry mouth and taste loss in patients treated with radioiodine [1197], and shortens the duration and severity of RIOM and diarrhea [1191], with efficacy also demonstrated when used as a mouthwash [1196,1198]. The phase II trial by Scartoni et al. was the first to demonstrate that Dixentil (a synbiotic supplement that also contains zinc and vitamins) significantly reduced pelvic RT-induced diarrhea in cancer patients compared with historical controls [1194].

#### 4.19.4. Fecal Microbiota Transplantation

Recently, FMT has emerged as a potential treatment for radiation-induced enteritis, involving the transfer of bacterial communities from healthy donors as fresh stool preparations or frozen capsules administered via oral, nasogastric, or rectal routes under highly stringent donor selection criteria [1163]. In vivo, FMT shows promising outcomes when performed shortly after irradiation in mice, as it increased survival rates, elevated peripheral white blood cell counts, and improved intestinal epithelial integrity and GI function [1225]. Mice that survived 9.2 Gy TBI harbored a protective microbiota enriched in *Lachnospiraceae* and *Enterococcaceae* [1226]. FMT from these survivors to irradiated germ-free mice significantly improved survival, likely through microbial metabolites such as SCFAs (mainly propionic acid) and tryptophan pathway metabolites, which preserved gut integrity and promoted hematopoietic recovery [1226]. FMT elevated levels of the microbially derived metabolite indole-3-propionic acid (IPA) in fecal pellets from irradiated mice. Later, oral IPA replenishment attenuated H-ARS and GI injury and enhanced survival without precipitating tumor growth in both male and female mice [1227].

In a pilot study, five female patients with chronic radiation enteritis were treated with FMT, and three of them showed improvement in diarrhea, rectal hemorrhage, and abdominal/rectal pain [1228]. These results are supported by a case report [1229] and a clinical study in which perioperative FMT combined with nutritional support was effective in improving early postoperative nutritional status and QoL in patients with IR-induced enteritis complicated by intestinal obstruction [1230]. Improvements in body mass index, total protein, prealbumin, and serum albumin levels were maintained at 3 and 6 months of follow-up [1230]. However, the safety of FMT remains a concern, and further clinical studies with larger patient cohorts are needed. A clinical trial currently in the recruitment phase (NCT06776029) will evaluate the therapeutic benefits of FMT for hemorrhagic radiation-induced rectal injury.

### 4.20. Other Topical Interventions for RID Prevention and Mitigation

The most widely accepted recommendation for patients with RID is to keep the affected area clean using mild soap and water and to allow the skin to heal naturally. In addition, several topical interventions, including corticosteroids, trolamine, aloe vera, hyaluronic acid, and film-forming barrier products, have been evaluated in clinical trials for the prevention and attenuation of RID. The mechanisms of action of these interventions and the outcomes reported in systematic reviews [179,1231,1232,1233,1234,1235,1236] are discussed below.

Hyaluronic acid/hyaluronan-based products have demonstrated efficacy in preventing non-severe RID (Grade ≤ 1) due to the hydrating properties that enhance skin tolerance to radiation and relieve dryness-related discomfort. However, clinical trials yielded contradictory results for more severe RID, and beyond its cosmetic benefits, no clear mechanism of action supports its potential radiomitigating efficacy [1232,1234,1235,1237]. Similarly, Trolamine (triethanolamine) emulsion (Biafine^®^) maintains a moist and oxygenated microenvironment that enhances dermal perfusion and favors the migration and proliferation of fibroblasts, leading to increased tissue formation and improved collagen organization. These effects indirectly stimulate keratinocyte re-epithelialization by restoring an optimal dermal substrate. Trolamine was found to be effective in preventing Grade ≤ 1 RID, but it was inefficient to prevent more severe cutaneous radiation injuries [175,1231,1234,1235].

Aloe vera contains aloin, emodin, vitamins (A, C, E) and other biologically active constituents with proven antioxidant and anti-inflammatory properties [1233,1238,1239]. Aloe vera extracts provided protection against IR-induced intestinal injury, skin reactions, hepatic renal, and testis damage [1238,1239]. Topical application of aloe vera promoted wound healing, reduced inflammatory cell infiltration, and increased epidermal thickness and collagen deposition. Wang et al. systematic review supports its prophylactic application during RT to reduce the incidence and severity of RID (notably grade 2–3), but heterogeneity between trials, variable product formulations, and possible publication bias limit the certainty of the conclusions [1233].

Olive oil is widely used in cosmetic formulations and contains several bioactive compounds with antioxidant and anti-inflammatory properties, including oleic acid, tocopherols, hydroxytyrosol, tyrosol, oleuropein, squalene, and linoleic acid, making it a promising agent for the prevention of RID [1240,1241]. Prophylactic application reduced the severity of acute RID in patients with nasopharyngeal carcinoma [1240], and comparable benefits were reported for olive oil and calcium hydroxide emulsions in breast cancer patients undergoing RT [1241].

Barrier films, mainly Hydrofilm^®^ and Mepitel^®^ film, have been standard of care in wound management for several decades. Both can be applied directly to the skin (or wound) and protect it from friction and excess moisture by providing a semi-permeable mechanical barrier over the damaged skin layer. Hydrofilm^®^ has stronger adhesive potential and could be preferred in complex anatomical regions whereas Mepitel^®^ film is easier to remove and might be preferred in patients with more sensitive or brittle skin. Mepitel^®^ Film applied throughout RT significantly reduced the incidence of grade 2–3 acute RID and moist desquamation, decreased pain and burning sensations, and lowered the need for antibiotic use in breast cancer patients undergoing CRT [1242]. It was also effective in those at high risk for acute RID, such as patients with large breasts, post-lumpectomy, or post-mastectomy undergoing adjuvant RT [1243]. The use of Mepitel^®^ Film in HNC patients was associated with a 2.24-fold reduction in the risk of developing grade 2 RID and was preferred over Biafine^®^ cream by treated patients [1244,1245]. Negative aspects in Mepitel^®^ Film studies include poor adherence, discomfort during hot weather and showering, and itchy skin underneath [1244,1245].

According to the latest MASCC Oncodermatology guidelines and international Delphi consensus recommendations, topical corticosteroids, particularly betamethasone and mometasone furoate, are strongly recommended for preventing acute RID and associated symptoms such as burning and itching, whereas evidence supporting hydrocortisone, beclomethasone, and methylprednisolone remains insufficient [178,179,1236]. Despite these recommendations, clinicians remain cautious about prescribing topical corticosteroids for RID prevention due to potential adverse effects and inconsistent results from RCTs, particularly in HNC patients [175,178,1236]. The consensus also supports the use of Hydrofilm^®^, Mepitel^®^ Film, and olive oil in breast cancer patients [178]. Other topical interventions discussed in this review, including PTX (with or without vitamin E), EGCG and CURC-based formulations, Gln-based therapies, and additional radiomitigative approaches, are not included in the MASCC guidelines because of insufficient evidence or lack of consensus, underscoring the need for further research [1234,1235].

Recently, carbon-based nanomaterials, including fullerenes, fullerenols, and metallofullerenols, have shown great promise as radioprotectants and radiomitigators [1246]. Compared with fullerenes, fullerenols (C_60_(OH)_n_, *n* = 12–26) have a polyhydroxylated structure that confers greater solubility and stability. Regarded as “free radical sponges”, fullerenols outperform conventional antioxidants (e.g., SOD mimetics, amifostine, vitamin E) by scavenging a wide range of reactive species, including HO^•^, O_2_^−•^ and RNS [1246,1247]. Their metal-ion chelating capacity also prevents Fenton and Haber–Weiss reactions involved in HO^•^ formation [1248]. Fullerenol@pectin@chitosan gel microspheres significantly reduced colonic inflammation and epithelial tight junction damage, increased the beneficial-to-harmful bacteria ratio, and significantly reduced IR-induced colitis in mice [1249]. A sprayable oral fullerenol hydrogel inhibited apoptosis and enhanced antioxidant enzyme activity, including SOD, CAT, and GPx, thereby protecting mucosal epithelial cells and preventing the progression of RIOM [1250], and similar results were obtained when a sodium hyaluronate hydrogel loaded with C_60_(OH)_20_ was assayed to prevent RID [1247]. Compared with trolamine cream, topical fullerenols more effectively reduce radiation-induced epidermal thickening, collagen deposition, and skin appendage damage, while promoting hair regeneration 35 days after exposure to 30 Gy of X-ray irradiation [1251]. Transdermal application of fullerenol emulsion prevented IR-induced endothelial damage and alleviated skin injury by upregulating VEGF, improving tissue perfusion, and modulating TGF-β to prevent chronic fibrosis [1248]. A recent clinical study (ChiCTR2400079800) showed that fullerene cream application delayed and reduced the grading of acute RID in breast cancer patients and improved patients’ QoL [1252]. The results of a completed trial in HNC patients (NCT06484166) have not been published yet.

## 5. Conclusions and Future Directions

Radiomitigators represent a cornerstone of MCM against IR injury, particularly because they can remain effective when administered after exposure. This post-exposure therapeutic window is essential in both mass-casualty scenarios and clinical oncology, where mitigation strategies must be deployable after biodosimetric assessment and without compromising tumor control.

Despite substantial progress in preclinical development, only a limited number of agents have received FDA regulatory approval, primarily within the hematopoietic space. Unlike neutrophil and monocyte support, for which several countermeasures are available, romiplostim remains the only FDA-approved agent to promote platelet recovery. Emerging TPO receptor agonists such as BBT-059^®^, eltrombopag, avatrombopag, hetrombopag, and JNJ-26366821 are therefore especially promising, as their introduction could expand the range of platelet-targeted MCMs available for stockpiling and emergency preparedness, potentially through an Animal Rule pathway similar to that used for romiplostim [362,419,1253]. Although JNJ-26366821 is still in preclinical development, it has shown efficacy in preventing RILI and may offer improved bioavailability compared with other TPO receptor agonists [370,371,373].

Given the central role of inflammatory activation in radiation injury, COX inhibitors have been explored as radiomitigators in both preclinical and clinical settings. While several studies report benefits in specific tissues or contexts, overall efficacy has been variable and clinical utility has most often been limited to pain relief and improvements in QoL [561,562,564,565,578]. This heterogeneity has been attributed in part to strong inhibition of prostaglandin E2, which exerts protective effects on the intestinal mucosa and supports innate immune responses required for post-radiation leukopoiesis. In contrast, polyphenols combine antioxidant and anti-inflammatory properties with favorable safety profiles and have demonstrated radiomitigative effects in preclinical models by attenuating mucosal injury, vascular dysfunction, and late fibrotic changes, as well as clinical benefits in reducing RIOM and RID [1059,1135,1254,1255,1256], with the additional advantage of exhibiting antitumor activity. Their main limitation remains their low bioavailability, although this constraint is increasingly being addressed through the development of nanobased delivery systems.

Endothelial/vascular injury is a key driver of radiation-inducedGI, pulmonary, renal, and cardiovascular damage, yet no radiomitigator targeting these mechanisms has been FDA-approved to date. Several candidates—including MnSOD mimetics, statins, ACE inhibitors, melatonin, metformin, and polyphenols—have demonstrated promising radiomitigative efficacy in experimental settings. Among them, statins, melatonin, and metformin show multi-organ radiomitigative effects in preclinical models by preserving epithelial and endothelial barriers and reducing inflammatory and profibrotic signaling. Their synergistic effects further support their potential use in rational polypharmacy strategies [161,387,877,879,880,971,982,987,1012]. However, in emergency situations involving hypoglycemia, hypoxia, renal impairment, or circulatory collapse, which are common after severe radiation exposure, metformin should be used cautiously because of the risk of worsening hypoglycemia or precipitating lactic acidosis. ACEis have demonstrated preclinical efficacy as radiomitigators across multiple tissues, with particular relevance for cardiopulmonary and renal injuries [71,189,811,812,815,840,841,847,854]. However, clinical evidence is largely derived from retrospective studies in hypertensive populations, introducing confounding related to baseline cardiovascular status and the multifactorial nature of symptoms. In mass-casualty scenarios, where dehydration due to burns, vomiting, or hemorrhage is common, administration warrants caution because of increased risk of hypotension, electrolyte imbalance, and acute kidney injury. Under these conditions, pharmacologic strategies aimed at enhancing Ang-(1–7) signaling may represent a safer alternative [819,820,822,866,867].

Overall, antioxidant-based interventions, including vitamin C and E, NAC, SOD mimetics, and polyphenols, have generally demonstrated greater efficacy as radioprotectors than as radiomitigators. As radiomitigators, SOD mimetics have shown efficacy in attenuating IR-induced pneumonitis, intestinal injury, and fibrosis in preclinical models [92,494,496,497,514,517,527]. In addition, avasopasem has shown clinical benefit in Phase II/III trials through attenuation of RIOM [498]. Prolonged administration of vitamin E in combination with PTX has shown substantial clinical benefit in both the prevention and treatment of RILI, fibrosis, and osteoradionecrosis [686,691,722,726,728,736,738]. Despite these encouraging outcomes, caution is warranted when using antioxidants or growth factors in oncology patients, as some studies suggest potential interference with antitumor efficacy. In contrast, antifibrotic strategies, particularly those targeting TGF-β and PDGF signaling [239,260,906,910], have consistently attenuated RIF and, in some cases, enhanced tumor radiosensitivity in both preclinical and clinical settings.

Nutritional support is a fundamental component of recovery following ARS, particularly in patients with GI-ARS, where early parenteral supplementation may be essential to sustain metabolic demands and preserve immune and epithelial function during periods of compromised absorption. As GI integrity improves, key nutrients such as selenium, zinc, Gln, and Arg can be transitioned to oral administration to support ongoing tissue repair and functional recovery. Selenium is an essential component of antioxidant enzymes [591,1257]; zinc supports DNA repair, immune competence, and mucosal integrity [613,627,639,641,647]; and Gln serves as a primary metabolic substrate for enterocytes and immune cells, promoting barrier restoration and regeneration [770,771,781,782,783]. Among these, Gln and zinc supplementation have shown the most consistent benefit in clinical trials, particularly in reducing the severity and duration of RIOM [786,1258]. In parallel, microbiome-directed interventions, particularly probiotics, are increasingly supported by mechanistic and translational evidence, highlighting their potential to preserve epithelial barrier function, modulate immune responses, and enhance functional recovery following radiation exposure [1211,1215]. Taken together, nutritional support can provide a foundational element for the development of multifaceted strategies to mitigate ARS, integrating pharmacologic and microbiome-based interventions to optimize tissue protection and recovery.

Cell-based therapies, including MSC and EV-based approaches, hold exceptional promise as regenerative medicine strategies for radiation injury, with the potential to restore tissues that would otherwise be irreversibly damaged [438,440,441,442,443,444,447,469,473]. These therapies act through multiple complementary mechanisms, including immunomodulation, reduction in oxidative stress and inflammation, preservation of vascular integrity, and stimulation of tissue regeneration across multiple tissues. Their compatibility with cryopreservation and rapid deployment further underscores their relevance for emergency preparedness. While challenges related to standardization, scalability, dosing, and long-term safety remain, the transformative potential of these approaches highlights the urgency of rigorous clinical investigation.

Topical interventions such as corticosteroids, trolamine, aloe vera, hyaluronic acid, olive oil, CURC, and EGCG have shown clinical efficacy in managing RID, although results vary across studies [1234,1235]. Topical corticosteroids are effective but require monitoring with prolonged use and are contraindicated in the presence of skin infection or ulceration [179,1236]. Trolamine, aloe vera, and hyaluronic acid primarily provide hydration and symptomatic relief, whereas olive oil, polyphenols, and barrier films act through mechanisms consistent with radiomitigation, including antioxidant and anti-inflammatory effects and promotion of tissue repair. To advance clinical translation, future studies should incorporate standardized endpoints and clearly distinguish true radiomitigative effects from supportive care, while defining optimal timing and treatment regimens.

Beyond therapeutic development itself, several translational barriers continue to limit progress in the field. Recurring methodological challenges include inconsistent use of the terms radioprotector and radiomitigator, heterogeneous compound nomenclature, and incomplete reporting of key trial design elements such as treatment timing, route of administration, and registration identifiers. These issues hinder evidence synthesis, reproducibility, and cross-study comparison, ultimately slowing translational advancement. In addition, biological variables known to influence radiation response, particularly age and sex [820,844,1259,1260,1261,1262,1263], remain underrepresented in preclinical study designs. Likewise, commonly used efficacy endpoints (typically 30-day survival in rodents and 60-day survival in NHP) only partially capture the complexity of radiation injury. Future studies should extend beyond short-term survival to assess long-term outcomes, DEARE development, and potential interactions with tumor control in RT settings. Finally, victims of accidental radiation exposure often develop RCI, a setting in which several agents effective against isolated radiation injury fail to demonstrate benefit. Notably, ghrelin and DIZE remain among the few candidates showing partial efficacy in murine CRI models, underscoring the need for expanded research in this area [70,822,956].

Against this backdrop of biological complexity and translational heterogeneity, pragmatic prioritization becomes essential. To guide practical interpretation, we propose a focused shortlist (Table 11) of the most promising radiomitigators demonstrating a 24–48 h post-exposure therapeutic window (or even longer), selected based on: (a) feasible routes of administration (oral administration preferred for emergency deployment); (b) established human safety profiles; (c) minimal evidence of tumor protection; and (d) evidence of preclinical survival and/or clinical benefits. This shortlist is not exhaustive but strategically centers on agents that combine mechanistic complementarity, translational feasibility, and favorable safety characteristics, thereby supporting rational polypharmacy approaches in future mitigation-oriented research. Growth factors primarily targeting neutrophil and monocyte recovery were not prioritized, given that multiple approved countermeasures already address this component of hematopoietic injury.

Despite substantial progress in the development of radiomitigators, translating preclinical success into clinically validated therapies remains a critical unmet need. Advancing our understanding of the natural history and molecular drivers of multiorgan injury and DEARE will be essential for designing interventions that not only improve early survival but also reduce long-term morbidity and restore function following radiation exposure.

Given the multifactorial and multi-organ nature of radiation injury, the future of radiomitigation will depend on moving beyond single-agent approaches toward mechanism-driven combination therapies. Although more complex to design and validate, rational combination strategies are more likely to yield clinically meaningful benefits. In this context, integrating artificial intelligence-based platforms to prioritize drug combinations based on mechanistic complementarity, toxicity profiles, and predicted clinical outcomes may accelerate progress in the field.

## Figures and Tables

**Table 1:** FDA-Approved Drugs to Mitigate Ionizing Radiation Injury in Radiological or Nuclear Emergencies (Limited Use) [42].

Name	Mechanism of Action	Approval
**Potassium Iodide**	Blocks radioactive iodine uptake by the thyroid	December 2001
**Radiogardase^®^ (Prussian Blue)**	Reduces Cs/Tl intestinal absorption	October 2003
**Ca-DTPA or Zn-DTPA**	Chelating agents that form hydrophilic complexes with radionuclides (Am, Cm and Pu), thereby enhancing renal elimination	August 2004
**Silverlon^®^**	Treatment of cutaneous radiation injury	October 2022
**Neupogen^®^ (G-CSF, Filgrastim)**	Stimulate granulopoiesis and promote mobilization of granulocytes, particularly neutrophils, thereby enhancing the body’s innate immune response	March 2015
**Nypozi^®^ (Filgrastim-txid)**		June 2024
**Zarxio^®^ (Filgrastim-sndz)**		October 2024
**Releuko^®^ (Filgrastim-ayow)**		April 2025
**Neulasta^®^ (Pegfilgrastim)**		November 2015
**Udenyca^®^ (Pegfilgrastim-cbqv)**		November 2022
**Stimufend^®^ (Pegfilgrastim-fpgk)**		September 2023
**Ziextenzo^®^ (Pegfilgrastim-bmez)**		February 2024
**Fylnetra^®^ (Pegfilgrastim-pbbk)**		April 2025
**Leukine^®^ (Sargramostim, GM-CSF)**	Induces the production and mobilization of granulocytes and monocytes, supporting immune function	March 2018
**Nplate^®^ (Romiplostim, rhTPO)**	Promotes thrombopoiesis, preventing hemorrhages	January 2021

**Table 2:** Completed Clinical Trials of SOD mimetics and Nitroxides in Patients Receiving RT or CRT. Abbreviations: CRT, chemoradiotherapy; HNC, head and neck cancer; IM, intramuscular; IMRT, intensity-modulated radiotherapy; IV, intravenous; mOS, median overall survival; RID, radiation-induced dermatitis; RIOM, radiation-induced oral mucositis; RT, radiotherapy; SC, subcutaneous; SOD, superoxide dismutase.

Treatment	Clinical Setting	Key Clinical Outcome	Trial Number	Ref.
**BMX-001** (SC)	HNC—RT/CRT	Lower rates of severe RIOM and xerostomia; exploratory signal requiring further validation	NCT02990468	[503]
**BMX-001** (SC)	High-grade glioma—CRT	Reduced white-matter damage and cognitive decline; increased mOS	NCT02655601	[504]
**BMX-001** (SC)	Anal cancer—CRT	Decreased acute CRT-related toxicities compared with historical controls	NCT03386500	[505]
**Cu/Zn SOD** (IM)	Mixed cancers—RT	Progressive reduction in skin fibrosis beginning week 3 and maximal by 2 months		[483]
**Cu/Zn SOD** (topical)	Breast cancer—RT	Reduced established radiation-induced skin fibrosis and pain		[485]
**SOD** (topical)	HNC—RT	No clinical benefit in the treatment of established fibrosis	NCT01771991	[506]
**GC4419** (IV)	Lung cancer—CRT	Reduced incidence of grade ≥ 3 esophagitis	NCT04529850	[501]
**GC4419** (IV)	HNC—IMRT/CRT	Reductions in severe RIOM incidence and duration	NCT03689712	[502,507]
**Tempol** (topical)	Cranial RT	Reduced incidence of RT-induced alopecia	NCT00713154	[508]
**Tempol** (topical)	Anal cancer—CRT	Did not reduce RID severity	NCT01324141	[509]

**Table 3:** Completed Clinical Trials of Benzydamine and COX inhibitors in Patients Receiving RT or CRT. Abbreviations: CRT, chemoradiotherapy; HNC, head and neck cancer; NSCLC, non–small cell lung cancer; OM, oral mucositis; RID, radiation-induced dermatitis; RIOM, radiation-induced oral mucositis; RT, radiotherapy.

Treatment	Clinical Setting	Key Clinical Outcome	Trial Number	Ref.
**Benzydamine** (oral rinse)	HNC—RT	Effective and well tolerated for RIOM prophylaxis		[556]
**Benzydamine** (oral rinse)	HNC—RT	Effective and well tolerated for RIOM prophylaxis		[557]
**Benzydamine** (oral rinse)	HNC—RT	Effective and well tolerated for RIOM prophylaxis	ISRCTN07033239	[558]
**Benzydamine** (oral rinse)	HNC—CRT	Reduced OM incidence and severity		[559]
**Benzydamine** (oral rinse)	HNC—RT	Effective and well tolerated for RIOM prophylaxis	NCT04685395	[560]
**Celecoxib** (oral)	Rectal cancer—CRT	Reduced pain	NCT00931203	[561]
**Celecoxib** (oral)	Colorectal cancer—CRT	Reduced incidence of severe CRT-related skin toxicity	NCT00250835	[562]
**Celecoxib** (oral)	Rectal cancer—CRT	Improved sphincter preservation, CRT compliance, and complete response rates		[563]
**Celecoxib** (oral)	Breast cancer—RT	Reduced itching and pain without significant reduction in RID severity	IRCT2014100619423N1	[564]
**Celecoxib** (oral)	NSCLC—CRT	Reduced incidence of symptomatic pneumonitis	NCT01503385	[565]
**Celecoxib** (oral)	HNC—RT	No attenuation of RIOM severity, dietary compromise, or opioid use	NCT00698204	[566]
**Celecoxib** (oral)	HNC—CRT	Delayed RIOM onset, reduced severity, and improved local tumor control	NCT00603759	[567]

**Table 4:** Completed Clinical Trials of Selenium and Zinc Supplementation in Cancer Patients subjected to RT or CRT. Abbreviations: CRT, chemoradiotherapy; HNC, head and neck cancer; HSCT, hematopoietic stem cell transplantation; NSCLC, non–small cell lung cancer; OM, oral mucositis; RID, radiation-induced dermatitis; RIOM, radiation-induced oral mucositis; RT, radiotherapy; TBI, total body irradiation.

Treatment	Clinical Setting	Key Clinical Outcome	Trial Number	Ref.
**Sodium selenite** (oral)	Breast cancer—RT	Reduced severity of RT-associated lymphedema		[603]
**Sodium selenite** (oral)	HNC—RT	Lower rates of ageusia and dysphagia		[600]
**Sodium selenite** (oral)	Gynecologic cancer—Pelvic RT	Lower diarrhea incidence and severity		[601,604]
**Selenium** (oral)	HSCT ± TBI	Reduced incidence and duration of severe RIOM	NCT01432873	[605]
**Sodium selenite** (oral)	HNC—RT	Improved wound healing		[606]
**Selenomethionine** (oral)	HNC—CRT	No significant reduction in severe RIOM incidence	NCT01682031	[609]
**Selenomethionine** (oral)	Inoperable stage III NSCLC—CRT	Reduced myelosuppression	NCT00526890	[610]
**Selenium** (oral)	Thyroid cancer—^131^I ablation therapy	Reduced salivary gland injury; lower serum amylase levels	IS14OISI0029	[607]
**Selenium** (oral)	HNC—RT	No effect on RIOM incidence or severity	IRCT2014072718612N1	[611]
**ZnSO_4_** (oral)	HNC—RT	Preserved taste acuity during RT		[612]
**ZnSO_4_** (oral)	HNC—RT	Delayed onset and reduced severity of RIOM		[613]
**ZnSO_4_** (oral)	HNC—RT/CRT	No significant effect on taste alterations		[614]
**ZnSO_4_** (oral)	HNC—RT	Reduced RIOM severity; no significant effect dysphagia		[615]
**ZnSO_4_** (oral)	HNC—RT	No reduction in RIOM or esophagitis incidence		[616]
**ZnSO_4_** (oral)	HNC—RT	Mitigated RT-induced taste alterations		[617]
**ZnSO_4_** (oral)	HNC—RT	No beneficial effect on RIOM or pharyngitis		[618]
**ZnSO_4_** (oral)	Oropharyngeal cancer—CRT	Delayed and attenuated oropharyngeal mucositis		[619]
**ZnSO_4_** (oral)	HNC—RT	Reduced severity of RIOM	IRCT20190123042475N2	[620]
**ZnSO_4_** (oral)	Breast cancer—RT	Reduced severity of RID	IRCT20200621047857N1	[621]
**ZnSO_4_** (oral)	HNC—RT	Delayed oropharyngeal mucositis onset and reduced severity	IRCT201106116734N3	[622]
**Polaprezinc** (rinsed or swallowed)	HNC—RT/CRT	Reduced incidence and severity of RIOM without compromising RT efficacy		[623]
**Polaprezinc** (oral rinse)	HNC—CRT	Attenuated OM severity		[624]
**Polaprezinc** (oral)	HNC—RT	Reduced incidence of severe (grade 3) RIOM		[625]
**Polaprezinc** (oral)	NSCLC—CRT	Delayed onset and reduced incidence of grade ≥ 2 esophagitis. No impact on tumor response		[626]
**Polaprezinc** (oral)	Breast cancer—RT	Delayed esophagitis onset, reduced dysphagia and steroid use	NCT03997188	[627]
**Polaprezinc** (mouthwash)	HSCT + TBI	No benefit in the incidence of severe (grade 3–4) RIOM	ACTRN12320001188921	[628]
**Pro-Z** (oral)	HNC—RT	Delayed onset and decreased severity of RIOM and RID		[629]
**Zinc oxide** (topical)	HNC—CRT	Improved RIOM severity		[630]

**Table 5:** Completed Clinical Trials Evaluating Vitamin E and/or Pentoxifylline in Cancer Patients Undergoing RT or CRT. Abbreviations: DFS, disease-free survival; FGF2, fibroblast growth factor 2; GI, gastrointestinal; GT3, gamma-tocotrienol; HNC, head and neck cancer; HSCT, hematopoietic stem cell transplantation; NPs, nanoparticles; OS, overall survival; PTX, pentoxifylline; QoL, quality of life; RIF, radiation-induced fibrosis; RID, radiation-induced dermatitis; RILI, radiation-induced lung injury; RIOM, radiation-induced oral mucositis; RN, radionecrosis; RT, radiotherapy; TBI, total body irradiation.

Treatment	Clinical Setting	Key Clinical Outcome	Trial Number	Ref.
**α-Tocopherol** (topical)	HNC—RT	Reduced incidence of symptomatic RIOM		[705]
**Vitamin E** (oral)	Thyroid cancer—^131^I	Preserved salivary gland function and reduced xerostomia		[708]
**α-Tocopherol + GT3** (oral)	HNC—post-RT fibrosis/trismus	Improved mouth opening.		[706]
**α-Tocopherol + β-carotene** (oral)	HNC—RT	Reduced severity of acute RT adverse effects; a trend toward higher local recurrence		[709]
**Vitamin E** (oral)	Thyroid cancer—^131^I	Preserved parotid/submandibular gland function		[286]
**Vitamin E** (mouthwash)	HSCT + TBI	Reduced duration of RIOM	IRCT20180416039325N1	[707]
**Vitamin E NPs** (topical)	Breast cancer—RT	No significant improvement in RID	UTN-U1111-1201-5923	[711]
**PTX** (oral)	HNC—RT-related RN	Improved soft-tissue RN symptoms	NCT01508221	[712]
**PTX** (oral)	Nasopharyngeal carcinoma—RT	Modest therapeutic effect in trismus		[713]
**PTX** (oral)	Breast or lung cancer—RT	Reduced early and late RILI		[714]
**PTX** (oral)	Mixed—RIF	Improved symptoms of established RIF, reducing edema, pain, and FGF2	NCT00001437	[715]
**PTX + tocopherol** (oral)	Breast cancer—RIF	Improved superficial established RIF with gradual maximal response over ~2 years	NCT00188552	[716,717]
**PTX + vitamin E** (oral)	Mixed—RIF	Reported benefit for established subcutaneous RIF		[718]
**PTX + vitamin E** (oral)	Breast cancer—RT	Greater reduction in RIF vs. vitamin E alone	ISRCTN39143623	[719]
**PTX + vitamin E** (oral)	Breast cancer—RT	Attenuated RIF; no difference in OS or DFS	NCT00583700	[720]
**PTX + tocopherol** (oral)	Lung cancer—RT	Reduced RILI		[721]
**PTX + tocopherol** (oral)	Lung cancer—RT	Reduced incidence of grade 3 radiation pneumonitis	NCT06634056	[722]
**PTX + α-tocopheryl acetate** (oral)	Breast cancer after RT	No improvement in lymphedema-associated fibrosis or QoL	NCT00022204	[723]
**PTX + α-tocopheryl acetate** (oral)	Pelvic RT-late effects	No meaningful changes in functional status or late side-effects		[724]
**PTX + vitamin E** (oral)	HNC—RT	Reduced RIOM severity and duration, with reduced dysphagia	NCT02397486	[163]
**PTX + Tocovid SupraBio** (oral)	Pelvic cancer—chronic GI toxicity post-RT	Decreased inflammatory biomarkers without clear clinical benefit	NCT02230800	[725]
**PTX + tocopherol + clodronate** (oral)	Mandibular osteoradionecrosis	High healing rates within 6–8 months; mucosal ulceration improved by 3–6 months	NCT02368457	[726,727]
**PTX + tocopherol** (oral)	RT-induced osteoradionecrosis	Reduced incidence of osteoradionecrosis after dental extractions		[728]
**PTX + tocopherol + clodronate** (oral)	RT-induced brachial plexopathy	No benefit in brachial plexopathy	NCT01291433	[729]

**Table 6:** Completed Clinical Trials Evaluating Amino Acid Supplementation in Patients Undergoing RT or CRT. Abbreviations: Arg, arginine; BMT, bone marrow transplantation; CRT, chemoradiotherapy; GI, gastrointestinal; Gln, glutamine; HMB, β-hydroxy-β-methylbutyrate; HNC, head and neck cancer; HSCT, hematopoietic stem cell transplantation; IMRT, intensity-modulated radiotherapy; IV, intravenous; NAC, N-acetylcysteine; NSCLC, non–small cell lung cancer; QoL, quality of life; RID, radiation-induced dermatitis; RIOM, radiation-induced oral mucositis; RT, radiotherapy; SCT, stem cell transplantation; TBI, total body irradiation; TGF-β1, transforming growth factor beta 1.

Treatment	Clinical Setting	Key Clinical Outcome	Trial Number	Ref.
**Arg and Gln** (oral)	HNC—IMRT	Reduced severity of stomatitis, pain and dysphagia		[751]
**Arg or Gln** (oral)	HNC—RT	Both agents significantly reduced RIOM severity, weight loss and improved QoL	NCT06764420	[752]
**Arg/Gln/HMB** (oral)	HNC—CRT	Reduced incidence and duration of grade 1–2 RID; no effect on grade ≥ 3 RID		[753]
**Arg/Gln/HMB** (oral)	HNC—CRT	Did not prevent severe OM but accelerated recovery	UMIN000016453	[754]
**Arg/Gln/HMB** (oral)	HNC—CRT	Prevented progression to grade 3 RIOM and attenuated treatment-related cachexia	UMIN000050011	[755]
**Gln** (swish solution)	HNC—RT Treatment	Reduced severity and duration of established RIOM		[756]
**Gln** (IV)	HNC—CRT	Reduced severity and duration of established RIOM		[757]
**Gln** (oral)	HNC—RT	Delayed onset and reduced incidence and duration of grade 3–4 RIOM		[758]
**Gln** (oral)	HNC—CRT	Reduced frequency and severity of RIOM	UMIN000003991	[759]
**Gln** (oral)	HNC—RT/CRT	Reduced odynophagia and mucositis severity; decreased RT interruptions		[760]
**Gln** (oral)	HNC—CRT	Reduced RIOM and dysphagia incidence; improved treatment compliance	CTRI/2017/02/007772	[162]
**Gln** (oral)	HNC—RT	Reduced salivary TGF-β1 levels and improved pain	NCT05856188	[158]
**Elental^®^ diet containing Gln**	HNC—CRT	Reduced RIOM severity and improved CRT completion rates	UMIN000008338	[761]
**Gln** (oral)	HNC—CRT	Reduced RID; no significant effect on RIOM	2009-018103-40	[762]
**Gln** (oral)	HNC—CRT	No significant reduction in RIOM or RID	NCT03015077	[763]
**Gln** (oral)	BMT	Reduced stomatitis severity in BMT patients		[764]
**Gln** (parenteral)	BMT	No significant reduction in mucositis, infections, or hospital stay		[765]
**Gln** (parenteral)	SCT + TBI	No significant reduction in RIOM; limited overall clinical benefit		[766]
**Gln** (parenteral)	HSCT + TBI	Reduced diarrhea duration but associated with increased mucositis severity and adverse outcomes		[767]
**Gln** (oral)	Pediatric HSCT + TBI	No significant reduction in RIOM incidence or severity	NCT00003898	[768]
**Gln** (parenteral)	Pediatric SCT + TBI	No reduction in mucosal morbidity or transplant-related complications		[769]
**Gln** (oral)	Esophageal cancer—CRT	Preserved lymphocyte counts and mitogenic response during CRT		[770]
**Gln** (oral)	NSCLC—CRT	Delayed onset and reduced esophagitis severity; limited weight loss		[771]
**Gln** (oral)	Thoracic malignancies—RT	No significant reduction in acute esophagitis severity	NCT01952847	[772]
**Gln** (oral)	Thoracic/upper aerodigestive cancers—RT	Reduced stomatitis, esophagitis, and weight loss	NCT05054517	[773]
**Gln** (oral)	Breast cancer—RT	Reduced radiation-related morbidity during breast RT		[774]
**Gln** (enteral)	Breast cancer—RT	Reduced severity of RID		[775]
**Gln** (oral)	Mixed—Pelvic RT	No reduction in acute radiation-induced diarrhea	NCT00003170	[747]
**Gln** (oral)	Rectal cancer—preoperative CRT	No reduction in CRT-induced diarrhea		[748]
**Gln** (oral)	Mixed—Pelvic RT	No prophylactic effect on radiation enteritis	NCT00828399	[749]
**Gln-oligomeric diet**	Rectal cancer—CRT	Reduced GI toxicity		[776]
**Gln-enriched diet**	Colorectal cancer—CRT	Reduced diarrhea and mucositis; decreased RT interruptions		[777]
**NAC polymer** (oral rinse)	HNC—RT	Reduced incidence of severe RIOM	NCT00230191	[778]
**NAC** (oral rinse)	HNC—CRT	Improved xerostomia and secretion scores	NCT02123511	[779]
**NAC** (inhalation)	HNC—RT	Improved patient-reported QoL during RT		[780]

**Table 7:** Impact of ACEi/ARB on cancer patients receiving RT or CRT: retrospective data. Abbreviations: ACEi, angiotensin-converting enzyme inhibitor; ARB, angiotensin II receptor blocker; CRT, chemoradiotherapy; GI, gastrointestinal; OS, overall survival; PFS, progression-free survival; RN, radionecrosis; RT, radiotherapy; SBRT, stereotactic body radiotherapy.

Clinical Setting	Main Clinical Findings	Ref.
**Glioblastoma—CRT**	Reduced corticosteroid requirements without impact on OS	[828]
	Improved 6-month functional independence, PFS, and OS	[829]
	Reduced incidence and severity of vasogenic peritumoral edema	[830]
**Brain Metastases—RT**	Reduced incidence of symptomatic RN	[831]
**Lung Cancer—Thoracic RT or CRT**	No significant reduction in symptomatic pneumonitis	[832]
	Reduced incidence of grade ≥ 2 pneumonitis, particularly in elderly patients	[833]
	No overall pneumonitis reduction	[823]
	Reduced incidence of grade ≥ 2 pneumonitis	[827]
**Lung Cancer—Thoracic SBRT**	Reduced incidence of symptomatic (grade ≥ 2) pneumonitis	[824,825,826]
	Improved OS and recurrence-free survival with ARB (not ACEi)	[834]
**Mixed—Pelvic RT or CRT**	Reduced acute GI toxicity with statin use alone or in combination with ACEi	[835]
**Prostate Cancer—Pelvic RT**	Lower incidence of grade ≥ 2 radiation proctitis and a shorter duration of symptoms	[836]
	Reduction in acute GI toxicity in users of statins (alone) or in combination with ACEi	[837]

**Table 8:** Clinical Outcomes of Melatonin in Patients Undergoing RT or CRT. Abbreviations: CRT, chemoradiotherapy; GB, glioblastoma; HNC, head and neck cancer; RID, radiation-induced dermatitis; RIOM, radiation-induced oral mucositis; RT, radiotherapy; γ-H2AX, phosphorylated histone H2AX.

Treatment	Clinical Setting	Key Clinical Outcome	Trial Number	Ref.
Melatonin (oral)	GB—RT	Improved 1-year survival, with fewer infections and alopecia		[989]
Melatonin (oral)	Mixed—Pelvic RT	Did not prevent RT-related lymphocyte decline		[990]
Melatonin (topical)	Breast cancer—RT	Reduced incidence and severity of RID	NCT00840515	[991]
Melatonin (gargle + capsules)	HNC—CRT	Delayed onset and reduced incidence of RIOM; no significant effect on xerostomia	NCT02430298	[992]
Melatonin (oral)	HNC—RT	Reduced RIOM severity and treatment-related pain	NCT03833570	[993]
Melatonin (mucoadhesive gel)	HNC—RT	Reduced RIOM incidence and duration	NCT02630004	[994]
Melatonin (oral)	Abdominopelvic computed tomography	Reduced RT-induced γ-H2AX foci formation in lymphocytes		[972]
Melatonin (oral)	Rectal cancer—CRT	No significant protection against CRT-induced cytopenias	IRCT2016021626586N1	[995]
Melatonin (oral)	Hyperthyroidism—^131^I therapy	Non-significant decrease in micronuclei frequency; increased therapeutic response rate	IRCT2014090419045N1	[996]
Melatonin (cream)	Breast cancer—RT	No significant reduction in RID	NCT03716583	[997]

**Table 9:** Clinical Outcomes of Polyphenols in Patients Undergoing RT or CRT. Abbreviations: CRT, chemoradiotherapy; CURC, curcumin; EGCG, epigallocatechin-3-gallate; GI, gastrointestinal; GSH, glutathione; HNC, head and neck cancer; OM, oral mucositis; OS, overall survival; PFS, progression-free survival; RID, radiation-induced dermatitis; RIF, radiation-induced fibrosis; RIOM, radiation-induced oral mucositis; RT, radiotherapy.

Treatment	Clinical Setting	Key Clinical Outcome	Trial Number	Ref.
**RayGel^®^** (anthocyanins and GSH)	Breast cancer—RT	Provided superior skin protection vs. standard care	NCT00266331	[1060]
**Proanthocyanidin grape extract** (oral)	Breast cancer—RT	No benefit in RIF reduction or patient-reported outcomes	NCT00041223	[1061]
**Turmeric** (gargle)	HNC—RT/CRT	Delayed onset and reduced RIOM severity		[1062]
**Meriva^®^** (CURC delivery system)	Mixed—RT/CRT	Alleviated RT-associated epithelial damage		[1063]
**CURC** (mouthwash)	HNC—CRT	Reduced RIOM severity and improved CRT compliance		[1064]
***Curcuma longa*** (oral gel)	HNC—RT	Decreased RIOM severity	IRCT138904064255N1	[1065]
**CURC** (oral gel)	HNC—RT/CRT	Reduced RIOM severity		[1066]
**CURC and sandal wood oil**	Breast cancer—RT	Delayed onset and reduced severity of RID		[1067]
**CURC** (oral)	HNC—RT/CRT	Reduced RIOM incidence, severity, improved RT/CRT compliance		[1068]
**CURC** (oral gel)	HNC—RT	Reduced RIOM severity and promoted mucosal healing	NCT05982197	[1069]
**CURC nanomicelles** (oral)	HNC—RT	Reduced incidence and severity of RIOM	IR.mums.sd.REC.1394.14	[1070]
**BCM-95^®^** (turmeric extract capsules)	HNC—RT/CRT	Reduced OM severity from week 3 of CRT onward	ISRCTN13817594	[1071]
**BCM-95^®^** (turmeric extract capsules)	Oral cancer—CRT	Reduced severe OM and improved CRT-tolerance	CTRI/2015/12/006413	[1072]
**SinaCurcumin^®^** (capsules)	HNC—RT/CRT	Delayed RIOM onset but did not reduce incidence/severity	CTRI/2018/04/013362	[584]
**SinaCurcumin^®^** (capsules)	HNC—RT/RCT	Effective in the prevention and treatment of RIOM	IRCT20100101002950N6	[1073]
**SinaCurcumin^®^** (capsules) or **CURC** (mouthwash)	HNC—RT	Both treatments reduced RIOM severity	IRCT20190810044500N17	[1074]
**Turmeric** (mouthwash)	HNC—RT/CRT	Reduced RIOM severity	CTRI/2018/06/014367	[585]
**Curcumin C3 Complex^®^** (oral)	Breast cancer—RT	Reduced RID severity	NCT01042938	[1075]
**Curcumin C3 Complex^®^** (oral)	Breast cancer—RT	Did not reduce RID severity	NCT01246973	[1076]
**Psoria-Gold^®^** (CURC gel)	Breast cancer—RT	Did not attenuate RID but improved severe skin reactions	NCT02536632	[173]
**CURC and sandal wood oil** (topical)	HNC—RT	Reduced incidence and severity of RID		[1077]
**CURC nanomicelles** (oral)	Breast cancer—RT	Trend toward reduced RID; not statistically significant	IRCT20200513047427N1	[1078]
**CURC** (topical)	Breast cancer—RT	Reduced RID and associated pain	IRCT20181208041882N3	[1079]
**CURC** (oral)	Prostate cancer—Pelvic RT	Reduced proctitis severity	NCT01917890	[1080]
**NanoCURC** (oral)	Prostate cancer—Pelvic RT	Reduced proctitis severity	NCT02724618	[1081]
**CURC** (oral)	Colorectal cancer—Pelvic CRT	No benefit for radiation enteritis	IRCT20220429054699N1	[1082]
**EGCG** (oral)	Lung cancer—RT/CRT	Alleviated acute esophagitis	NCT01481818	[1083]
**EGCG** (oral)	Lung cancer—RT	Alleviated acute esophagitis. No adverse impact on PFS or OS.	NCT02577393	[1084,1085]
**EGCG** (oral)	Esophagus cancer—RT	Reduced esophagitis severity	NCT05039983	[1086]
**EGCG** (topical)	Breast cancer—RT	Reduced severity of RID		[1087]
**EGCG** (topical)	Breast cancer—RT	Reduced incidence/severity of RID	NCT02580279	[1088,1089]
**EGCG** (mouthwash)	HNC—RT	Attenuated RIOM and associated pain		[1090]
**EGCG** (oral)	Mixed—Pelvic RT	Reduction in intestinal adverse effects	ChiCTR2100053703	[1091]
**Green tea** (oral)	Mixed—Pelvic RT	Lower incidence of diarrhea and some benefit for vomiting	IRCT2013052213433N1	[1092]
**Resveratrol, lycopene, vitamin C and anthocyanin** (oral)	Breast cancer—CRT	Lower incidence of moderate-severe RID		[1093]
**Silymarin-based cream**	Breast cancer—RT	Attenuated RID severity		[1094]
**Silymarin** (topical gel)	Breast cancer—RT	Delayed onset and decreased severity of RID	IRCT2016110730760N1	[1095]
**Silymarin** (oral)	HNC—RT	Delayed onset and reduced incidence of severe RIOM	IRCT2015050622132N1	[1096]
**Soy isoflavones** (oral)	Prostate cancer—Pelvic RT	Attenuated urinary, GI, and erectile toxicity with preserved RT efficacy	NCT00243048	[1097]

**Table 10:** Clinical Trials of Microbiome-Targeted Strategies for Preventing and Managing RT- and CRT-Induced Toxicities. Abbreviations: B., *Bifidobacterium*; CNS, central nervous system; CRT, chemoradiotherapy; GI, gastrointestinal; GVHD, graft-versus-host disease; HNC, head and neck cancer; HSCT, hematopoietic stem cell transplantation; L., *Lactobacillus*; OM, oral mucositis; QoL, quality of life; RIOM, radiation-induced oral mucositis; RT, radiotherapy; TBI, total body irradiation.

Probiotics				
Treatment	Clinical Setting	Key Clinical Outcome	Trial Number	Ref.
***L. brevis CD2 lozenges*** (lozenges)	HNC—CRT	Decreased incidence and RIOM severity. Enhanced CRT completion rate	CTRI/2008/091/000117	[1167]
***L. brevis CD2 lozenges*** (lozenges)	HNC—RT/CRT	No difference in grade 3–4 RIOM, pain, dysphagia, weight loss, or QoL	NCT01707641	[1168]
***B. longum, L. lactis* and *Enterococcus faecium*** (capsules)	Nasopharyngeal carcinoma—CRT	Reduced RIOM by modulating gut microbiota and enhancing immunity	NCT03112837	[1169]
***B. animalis, L. plantarum, L. rhamnosus*, and *L. acidophilus*** (capsules)	HNC post—RT	Significantly reduced *Candida* spp. colonization	CTRI/2018/02/011812	[1170]
***L. acidophilus, L. rhamnosus, B. longum*, and *S. boulardii*** (rinsed and swallowed)	HNC—RT/CRT	Delayed onset and reduced incidence and duration of high-grade RIOM		[1171]
***Bacillus clausii* UBBC07 spores** (oral)	HNC—RT	Reduced severe RIOM incidence and duration	NCT05918224	[1172]
***Streptococcus salivarius* K12** (lozenges)	HNC—RT	Reduced severe RIOM and opportunistic oral pathogens	NCT06446180	[1173]
**K12@Lip@GSH** (lozenges)	HNC—RT	Reduced RIOM incidence and severity in HNC patients	NCT03552458	[1174]
***Limosilactobacillus reuteri*** (droplets)	Pelvic malignancies—Pelvic RT	Improved diarrhea grade and stool consistency		[1175]
***L. rhamnosus*** (sachets)	Mixed—Pelvic RT	Reduced diarrhea incidence and severity		[1176]
**VSL#3 Probiotic preparation** (sachets)	Gynecologic cancers—Pelvic RT	No reduction in diarrhea incidence but improved stool consistency		[1177]
***L. casei* DN-114 001 **(oral)	Cervical cancer—Pelvic CRT	Reduced diarrhea incidence and severity		[1178]
***L. acidophilus* + *B. bifidum*** (capsules)	Pelvic cancers—Pelvic RT	Reduced diarrhea at end of RT and 2 weeks post-RT, but not throughout RT	NCT01839721	[1179]
***L. acidophilus* + *B. longum*** (capsules)	Cervical cancer—Pelvic RT	Reduced diarrhea incidence and severity	TCTR20170314001	[1180]
***L. acidophilus* LA-5 + *B. animalis lactis* BB-12** (capsules)	Gynecologic cancers—Pelvic RT	Reduced diarrhea incidence and severity	NCT02351089	[1181]
***Lactiplantibacillus plantarum* HEAL9 and 299** (capsules)	Pediatric CNS tumors—RT	Alleviated RT-related GI symptoms		[1182]
***Bacillus licheniformis*** (capsules)	Pelvic malignancies—Pelvic RT	No preventive effect on RT-induced enteropathy	NCT03978949	[1183]
**Prebiotics**				
**Treatment**	**Clinical Setting**	**Key Clinical Outcome**	**Trial Number**	**Ref.**
**Psyllium husk (Metamucil**^®^**)** (oral)	Pelvic malignancies—Pelvic RT	Reduced diarrhea incidence and severity		[1184]
**Water-soluble rice bran fiber** (oral)	Cervical cancer—CRT	Trend toward reduction in diarrhea; limited by small sample size	UMIN000004350	[1185]
**Resistant starch** (oral)	Cervical cancer—Pelvic RT/CRT	No significant benefit for radiation proctitis or diarrhea	CTRI/2010/091/000427	[1186]
** High-fiber diet **	Pelvic malignancies—Pelvic RT	Reduced acute and 1-year GI toxicity	NCT01170299	[1187]
**Partially hydrolyzed guar gum** (oral)	Mixed—Pelvic RT	Modestly improved bowel tolerance during RT	ISRCTN17271186	[1188]
**Fiber and lactose diet**	Prostate cancer—Pelvic RT	Reduced bloody stools, flatulence, and loss of appetite		[1189]
**Inulin and fructo-oligosaccharide diet**	Mixed—Abdominal RT	Improved microbiome recovery post-RT	NCT01549782	[1190]
**Inulin and fructo-oligosaccharide diet**	Gynecologic cancer—RT	Reduced number of days with watery stool (Bristol score 7)	NCT01549782	[1161]
**Resistant starch, polydextrose, lactosucrose and Gln** (oral)	HSCT + TBI	Shortened duration of OM and diarrhea and reduced incidence/severity of acute GVHD	UMIN000027563	[1191]
**Synbiotics**				
**Treatment**	**Clinical Setting**	**Key Clinical Outcome**	**Trial Number**	**Ref.**
***L. acidophilus* and lactulose** (oral)	Mixed—RT	Lower diarrhea rates, increased flatulence attributed to lactulose		[1192]
***L. reuteri* and soluble fiber** (sachets)	Prostate cancer—RT	Reduced proctitis symptoms and rectal inflammation; improved QoL	NCT01901042	[1193]
**Dixentil** (oral)	Mixed—pelvic RT	Reduced diarrhea incidence and severity		[1194]
**Probiotics plus hydroxypropyl methyl cellulose** (capsules)	Rectal cancer—CRT	Improved QoL and reduced inflammatory biomarkers	IRCT201503181197N18	[1195]
**Synbiotic** (mouthwash)	Oral cancer—RT	Prevented RIOM and reduced its severity	IRCT20201106049288N1	[1196]
***Streptococcus thermophilus*, L. and B. spp. plus fructooligosaccharides** (capsules)	Thyroid cancer—^131^I therapy	Limited improvement in dry mouth/taste	IRCT20220226054126N1	[1197]
**Synbiotic** (mouthwash)	HNC—RT	Delayed onset and reduced severity of RIOM	IRCT20230624058564N1	[1198]
***L. plantarum*, blueberry husks and fiber diet**	Rectal cancer—RT	Preserved gut microbiota diversity and reduced rectal mucosa inflammation	NCT03420443	[1199]

**Table 11:** Strategic Prioritization of Radiomitigators Based on Preclinical and Clinical Evidence. Abbreviations: ACEis, angiotensin-converting enzyme inhibitors; ARS, acute radiation syndrome; DEARE, delayed effects of acute radiation exposure; EV, extracellular vesicle; GI-ARS, gastrointestinal acute radiation syndrome; H-ARS, hematopoietic acute radiation syndrome; MSC, mesenchymal stem cells; PTX, pentoxifylline; RID, radiation-induced dermatitis; RIF, radiation-induced fibrosis; RILF, radiation-induced lung fibrosis; RILI, radiation-induced lung injury; RIOM, radiation-induced oral mucositis; RT, radiotherapy; SC, subcutaneous; TBI, total body irradiation; TPO, thrombopoietin.

Radiomitigator	Primary Scenario(s)	Route Feasibility	Human Safety	Risk of Tumor Protection	Preclinical Survival and Clinical Outcomes
**TPO-receptor agonists** (rhIL-11, eltrombopag, avatrombopag, hetrombopag, and JNJ-26366821)	ARS; RT-induced myelosuppression (preclinical and clinical)	Oral/SC	High (aligned with Nplate^®^; potential Animal Rule pathway)	Low	Improved survival in murine models of H-ARS. Except for JNJ-26366821, all are in clinical use for the management of thrombocytopenia in oncology and hematologic settings.
**MSC/EV-based approaches**	ARS; RT toxicity DEARE; (preclinical)	IV/locoregional	Moderate (clinical safety in other indications)	Uncertain	Improved survival in H- and GI-ARS models. Prevention of DEARE, including RILI and RILF. Clinical improvement of RT-induced xerostomia.
**Melatonin**	ARS (preclinical); RT toxicity (clinical)	Oral	Excellent (widely used)	Low (survival benefits in some cancers)	Mitigation of RILI and RILF and improved survival in murine models of H- and GI-ARS. Attenuation of RIOM and RT-induced skin toxicity in clinical studies.
**Statins** (simvastatin and atorvastatin)	RT toxicity (clinical); DEARE (preclinical)	Oral	Excellent (widely used)	Low (survival benefits in some cancers)	No survival benefits in ARS. Attenuation of endothelial and vascular damage, contributing to the prevention of DEARE (pulmonary fibrosis, cardiac dysfunction, and renal injury) with clinical evidence of benefit in RID.
**Vitamin E** ± **PTX**	ARS and DEARE (preclinical); RT toxicity (clinical)	Oral	Excellent (widely used)	Uncertain (antioxidant may compromise tumor control)	Increased survival in murine models of H-ARS, with mitigation of RILI. Attenuation and partial regression of established RIF (cutaneous, pulmonary, and mandibular) in clinical trials.
**ACEis** (Captopril, Lisinopril and Enalapril)	ARS (preclinical); RT toxicity (clinical); DEARE (preclinical)	Oral	Excellent (widely used)	Low (survival benefits in some cancers)	Increased mouse survival following TBI mainly by mitigating RILI and nephropathy, key contributors to late mortality. Observational studies suggest reduced incidence of RILI.

## Data Availability

No new data were created or analyzed in this study.

## Abbreviations

The following abbreviations are used in this manuscript:

5-AED	5-androstenediol
ACEi	angiotensin-converting enzyme inhibitor
Ang II	angiotensin II
aPC	activated protein C
ARB	angiotensin II blocker
Arg	arginine
ARS	acute radiation syndrome
AT-MSCs	adipose tissue–derived MSCs
AT1R	angiotensin ii receptor type 1
BBB	blood–brain barrier
BM	bone marrow
BM-MSCs	bone marrow–derived mesenchymal stromal cells
BMT	bone marrow transplantation
C-ARS	cutaneous acute radiation syndrome
CAT	catalase
CCN2	(CTGF) connective tissue growth factor
CNS	central nervous system
COX	cyclooxygenase
CRT	chemoradiotherapy
CT	chemotherapy
CTGF	connective tissue growth factor
CURC	curcumin
DAMPs	damage-associated molecular patterns
DEARE	delayed effects of acute radiation exposure
DRF	dose-reduction fraction
DSBs	double-strand breaks
DTPA	diethylenetriaminepentaacetate
ECM	extracellular matrix
EGCG	epigallocatechin-3-gallate
EGF	epidermal growth factor
EMT/EndoMT	epithelial-to-mesenchymal/endothelial-to-mesenchymal transitions
EVs	extracellular vesicles
FDA	U.S. Food and Drug Administration
FMT	fecal microbiota transplantation
GB	glioblastoma
G-CSF	granulocyte colony-stimulating factor
GI	gastrointestinal
GI-ARS	gastrointestinal acute radiation syndrome
Gln	glutamine
GM-CSF	granulocyte/macrophage colony-stimulating factor
GPx	glutathione peroxidase
GSH	reduced glutathione
GT3	gamma-tocotrienol
H-ARS	hematopoietic acute radiation syndrome
HNC	head and neck cancer
HR	homologous recombination
HSC	hematopoietic stem cells
HSCT	hematopoietic stem cell transplantation
IGF-1	insulin-like growth factor I
IM	intramuscular
IR	ionizing radiation
ISOO	International Society of Oral Oncology
IV	intravenous
KGF	keratinocyte growth factor
LD	lethal dose
MAPK	mitogen-activated protein kinase
MASCC	Multinational Association of Supportive Care
MCM	medical countermeasures
MDA	malondialdehyde
mOS	median overall survival
MRI	magnetic resonance imaging
MSCs	mesenchymal stromal cells
NAC	N-acetylcysteine
NAD^+^	nicotinamide adenine dinucleotide
NHP	non-human primates
NIS	sodium/iodide symporter
NP	nanoparticle
NOX	NADPH oxidase
NR	nicotinamide riboside
NSCLC	non-small cell lung carcinoma
NV-ARS	neurovascular acute radiation syndrome
OM	oral mucositis
OS	overall survival
PAI-1	plasminogen activator inhibitor-1
PB	prussian blue
PBI	partial-body irradiation
PDGF	platelet-derived growth factor
PFD	pirfenidone
PFS	progression-free survival
PTX	pentoxifylline
PSA	prostate-specific antigen
QoL	quality of life
RAS	renin–angiotensin system
RCI	radiation combined injury
RCT	randomized controlled trial
rh	recombinant human
RIBE	radiation bystander effect
RID	radiation-induced dermatitis
RIF	radiation-induced fibrosis
RILI	radiation-induced pulmonary injury
RILF	radiation-induced lung fibrosis/radiation-induced pulmonary fibrosis
RIOM	radiation-induced oral mucositis
RN	radiation-induced necrosis
RNS	reactive nitrogen species
ROS	reactive oxygen species
RT	radiotherapy
RTOG	radiation therapy oncology group
SASP	senescence-associated secretory phenotype
SC	subcutaneous
SCFA	short-chain fatty acids
SCT	stem cell transplantation
SOD	superoxide dismutase
Se	selenium
SSBs	single-strand breaks
TBI	total body irradiation
TLRs	toll-like receptors
TM	thrombomodulin
TPO	thrombopoietin
VEGF	vascular endothelial growth factor
WTI	whole-thorax irradiation

## References

[1] Azzam E.I., Jay-Gerin J.-P., Pain D. (2012). Ionizing Radiation-Induced Metabolic Oxidative Stress and Prolonged Cell Injury. Cancer Lett..

[2] Zhou H., Ivanov V.N., Lien Y.-C., Davidson M., Hei T.K. (2008). Mitochondrial Function and Nuclear Factor-κB–Mediated Signaling in Radiation-Induced Bystander Effects. Cancer Res..

[3] Yakovlev V.A. (2015). Role of Nitric Oxide in the Radiation-Induced Bystander Effect. Redox Biol..

[4] Jella K.K., Moriarty R., McClean B., Byrne H.J., Lyng F.M. (2018). Reactive Oxygen Species and Nitric Oxide Signaling in Bystander Cells. PLoS ONE.

[5] Reindl J., Abrantes A.M., Ahire V., Azimzadeh O., Baatout S., Baeyens A., Baselet B., Chauhan V., Da Pieve F., Delbart W., Baatout S. (2023). Molecular Radiation Biology. Radiobiology Textbook.

[6] Christensen D.M., Iddins C.J., Parrillo S.J., Glassman E.S., Goans R.E. (2014). Management of Ionizing Radiation Injuries and Illnesses, Part 4: Acute Radiation Syndrome. J. Osteopath. Med..

[7] Obrador E., Salvador-Palmer R., Villaescusa J.I., Gallego E., Pellicer B., Estrela J.M., Montoro A. (2022). Nuclear and Radiological Emergencies: Biological Effects, Countermeasures and Biodosimetry. Antioxidants.

[8] Kazzi Z., Buzzell J., Bertelli L., Christensen D. (2015). Emergency Department Management of Patients Internally Contaminated with Radioactive Material. Emerg. Med. Clin. N. Am..

[9] Ibáñez B., Melero A., Montoro A., Onofre N.S., Soriano J.M. (2024). Molecular Insights into Radiation Effects and Protective Mechanisms: A Focus on Cellular Damage and Radioprotectors. Curr. Issues Mol. Biol..

[10] Winters T.A., Cassatt D.R., Harrison-Peters J.R., Hollingsworth B.A., Rios C.I., Satyamitra M.M., Taliaferro L.P., DiCarlo A.L. (2023). Considerations of Medical Preparedness to Assess and Treat Various Populations During a Radiation Public Health Emergency. Radiat. Res..

[11] Thomas G.A., Symonds P. (2016). Radiation Exposure and Health Effects—Is It Time to Reassess the Real Consequences?. Clin. Oncol. (R. Coll. Radiol.).

[12] Baudin C., Bernier M.-O., Klokov D., Andreassi M.G. (2021). Biomarkers of Genotoxicity in Medical Workers Exposed to Low-Dose Ionizing Radiation: Systematic Review and Meta-Analyses. Int. J. Mol. Sci..

[13] Little M.P., Wakeford R., Bouffler S.D., Abalo K., Hauptmann M., Hamada N., Kendall G.M. (2022). Cancer Risks Among Studies of Medical Diagnostic Radiation Exposure in Early Life Without Quantitative Estimates of Dose. Sci. Total Environ..

[14] Hou N., Wang Z., Ling Y., Hou G., Zhang B., Zhang X., Shi M., Chu Z., Wang Y., Hu J. (2024). Radiotherapy and Increased Risk of Second Primary Cancers in Breast Cancer Survivors: An Epidemiological and Large Cohort Study. Breast.

[15] Rump A., Becker B., Eder S., Lamkowski A., Abend M., Port M. (2018). Medical Management of Victims Contaminated with Radionuclides After a “Dirty Bomb” Attack. Mil. Med. Res..

[16] Li C., Alves dos Reis A., Ansari A., Bertelli L., Carr Z., Dainiak N., Degteva M., Efimov A., Kalinich J., Kryuchkov V. (2022). Public Health Response and Medical Management of Internal Contamination in Past Radiological or Nuclear Incidents: A Narrative Review. Environ. Int..

[17] Gasperetti T., Miller T., Gao F., Narayanan J., Jacobs E.R., Szabo A., Cox G.N., Orschell C.M., Fish B.L., Medhora M. (2021). Polypharmacy to Mitigate Acute and Delayed Radiation Syndromes. Front. Pharmacol..

[18] Obrador E., Salvador R., Villaescusa J.I., Soriano J.M., Estrela J.M., Montoro A. (2020). Radioprotection and Radiomitigation: From the Bench to Clinical Practice. Biomedicines.

[19] Liu L., Liang Z., Ma S., Li L., Liu X. (2023). Radioprotective Countermeasures for Radiation Injury (Review). Mol. Med. Rep..

[20] Liu R., Bian Y., Liu L., Liu L., Liu X., Ma S. (2022). Molecular Pathways Associated with Oxidative Stress and Their Potential Applications in Radiotherapy (Review). Int. J. Mol. Med..

[21] DiCarlo A.L., Horta Z.P., Aldrich J.T., Jakubowski A.A., Skinner W.K., Case C.M. (2019). Use of Growth Factors and Other Cytokines for Treatment of Injuries During a Radiation Public Health Emergency. Radiat. Res..

[22] Singh V.K., Seed T.M. (2021). Radiation Countermeasures for Hematopoietic Acute Radiation Syndrome: Growth Factors, Cytokines and Beyond. Int. J. Radiat. Biol..

[23] Dainiak N. (2018). Medical Management of Acute Radiation Syndrome and Associated Infections in a High-Casualty Incident. J. Radiat. Res..

[24] Dainiak N., Albanese J. (2022). Medical Management of Acute Radiation Syndrome. J. Radiol. Prot..

[25] Arnautou P., Garnier G., Maillot J., Konopacki J., Brachet M., Bonnin A., Amabile J.-C., Malfuson J.-V. (2024). Management of Acute Radiation Syndrome. Transfus. Clin. Biol..

[26] Plett P.A., Chua H.L., Sampson C.H., Katz B.P., Fam C.M., Anderson L.J., Cox G., Orschell C.M. (2014). PEGylated G-CSF (BBT-015), GM-CSF (BBT-007), and IL-11 (BBT-059) Analogs Enhance Survival and Hematopoietic Cell Recovery in a Mouse Model of the Hematopoietic Syndrome of the Acute Radiation Syndrome. Health Phys..

[27] Wong K., Chang P.Y., Fielden M., Downey A.M., Bunin D., Bakke J., Gahagen J., Iyer L., Doshi S., Wierzbicki W. (2020). Pharmacodynamics of Romiplostim Alone and in Combination with Pegfilgrastim on Acute Radiation-Induced Thrombocytopenia and Neutropenia in Non-Human Primates. Int. J. Radiat. Biol..

[28] López M., Martín M. (2011). Medical Management of the Acute Radiation Syndrome. Rep. Pract. Oncol. Radiother..

[29] Al-Ibraheem A., Moghrabi S., Abdlkadir A., Safi H., Kazzi Z., Al-Balooshi B., Salman K., Khalaf A., Zein M., Al Naemi H. (2024). An Overview of Appropriate Medical Practice and Preparedness in Radiation Emergency Response. Cureus.

[30] Vellayappan B., Lim-Fat M.J., Kotecha R., De Salles A., Fariselli L., Levivier M., Ma L., Paddick I., Pollock B.E., Regis J. (2024). A Systematic Review Informing the Management of Symptomatic Brain Radiation Necrosis After Stereotactic Radiosurgery and International Stereotactic Radiosurgery Society Recommendations. Int. J. Radiat. Oncol. Biol. Phys..

[31] Zakeri K., Narayanan D., Vikram B., Evans G., Coleman C.N., Prasanna P.G.S. (2019). Decreasing the Toxicity of Radiation Therapy: Radioprotectors and Radiomitigators Being Developed by the National Cancer Institute Through Small Business Innovation Research Contracts. Int. J. Radiat. Oncol. Biol. Phys..

[32] Stasiłowicz-Krzemień A., Gościniak A., Formanowicz D., Cielecka-Piontek J. (2024). Natural Guardians: Natural Compounds as Radioprotectors in Cancer Therapy. Int. J. Mol. Sci..

[33] Citrin D., Cotrim A.P., Hyodo F., Baum B.J., Krishna M.C., Mitchell J.B. (2010). Radioprotectors and Mitigators of Radiation-Induced Normal Tissue Injury. Oncologist.

[34] Singh V.K., Seed T.M. (2017). A Review of Radiation Countermeasures Focusing on Injury-Specific Medicinals and Regulatory Approval Status: Part I. Radiation Sub-Syndromes, Animal Models and FDA-Approved Countermeasures. Int. J. Radiat. Biol..

[35] Montoro A., Obrador E., Mistry D., Forte G.I., Bravatà V., Minafra L., Calvaruso M., Cammarata F.P., Falk M., Schettino G., Baatout S. (2023). Radioprotectors, Radiomitigators, and Radiosensitizers. Radiobiology Textbook.

[36] Lledó I., Ibáñez B., Melero A., Montoro A., Merino-Torres J.F., San Onofre N., Soriano J.M. (2023). Vitamins and Radioprotective Effect: A Review. Antioxidants.

[37] Prades-Sagarra È., Yaromina A., Dubois L.J. (2023). Polyphenols as Potential Protectors Against Radiation-Induced Adverse Effects in Patients with Thoracic Cancer. Cancers.

[38] Ji L., Cui P., Zhou S., Qiu L., Huang H., Wang C., Wang J. (2023). Advances of Amifostine in Radiation Protection: Administration and Delivery. Mol. Pharm..

[39] Singh V.K., Seed T.M. (2024). New Pharmaceutical-Based Strategies That Foster the Development and Promulgation of Globally Effective Medical Countermeasures for Radiation Exposure Injuries. Expert Opin. Pharmacother..

[40] Singh V.K., Seed T.M. (2019). The Efficacy and Safety of Amifostine for the Acute Radiation Syndrome. Expert Opin. Drug Saf..

[41] Brizel D.M., Wasserman T.H., Henke M., Strnad V., Rudat V., Monnier A., Eschwege F., Zhang J., Russell L., Oster W. (2000). Phase III Randomized Trial of Amifostine as a Radioprotector in Head and Neck Cancer. J. Clin. Oncol..

[42] FDA U.S. Food and Drug Administration Radiological and Nuclear Emergency Preparedness Information from FDA. https://www.fda.gov/emergency-preparedness-and-response/mcm-issues/radiological-and-nuclear-emergency-preparedness-information-fda.

[43] Legeza V., Grebenyuk A., Drachev I. (2019). Radiomitigators: Classification, Pharmacological Properties, and Application Prospects. Biol. Bull..

[44] Vasin M.V., Ushakov I.B. (2019). Potential Ways to Increase Body Resistance to Damaging Action of Ionizing Radiation with Radiomitigators. Biol. Bull. Rev..

[45] Yu Z., Xu C., Song B., Zhang S., Chen C., Li C., Zhang S. (2023). Tissue Fibrosis Induced by Radiotherapy: Current Understanding of the Molecular Mechanisms, Diagnosis and Therapeutic Advances. J. Transl. Med..

[46] Turnquist C., Harris B.T., Harris C.C. (2020). Radiation-Induced Brain Injury: Current Concepts and Therapeutic Strategies Targeting Neuroinflammation. Neuro-Oncol. Adv..

[47] Wang B., Wei J., Meng L., Wang H., Qu C., Chen X., Xin Y., Jiang X. (2020). Advances in Pathogenic Mechanisms and Management of Radiation-Induced Fibrosis. Biomed. Pharmacother..

[48] MacVittie T.J. (2023). Where Are the Medical Countermeasures Against the ARS and DEARE? A Current Topic Relative to an Animal Model Research Platform, Radiation Exposure Context, the Acute and Delayed Effects of Acute Exposure, and the FDA Animal Rule. Int. J. Radiat. Biol..

[49] Winters T.A., Marzella L., Molinar-Inglis O., Price P.W., Han N.C., Cohen J.E., Wang S.-J., Fotenos A.F., Sullivan J.M., Esker J.I. (2024). Gastrointestinal Acute Radiation Syndrome: Mechanisms, Models, Markers, and Medical Countermeasures. Radiat. Res..

[50] Yahyapour R., Amini P., Rezapour S., Cheki M., Rezaeyan A., Farhood B., Shabeeb D., Musa A.E., Fallah H., Najafi M. (2018). Radiation-Induced Inflammation and Autoimmune Diseases. Mil. Med. Res..

[51] Wei J., Wang B., Wang H., Meng L., Zhao Q., Li X., Xin Y., Jiang X. (2019). Radiation-Induced Normal Tissue Damage: Oxidative Stress and Epigenetic Mechanisms. Oxidative Med. Cell. Longev..

[52] Munro T.R. (1970). The Relative Radiosensitivity of the Nucleus and Cytoplasm of Chinese Hamster Fibroblasts. Radiat. Res..

[53] Thompson L.H. (2012). Recognition, Signaling, and Repair of DNA Double-Strand Breaks Produced by Ionizing Radiation in Mammalian Cells: The Molecular Choreography. Mutat. Res. Rev. Mutat. Res..

[54] Melia E., Parsons J.L. (2023). DNA Damage and Repair Dependencies of Ionising Radiation Modalities. Biosci. Rep..

[55] Lomax M.E., Folkes L.K., O’Neill P. (2013). Biological Consequences of Radiation-Induced DNA Damage: Relevance to Radiotherapy. Clin. Oncol. (R. Coll. Radiol.).

[56] Zhou L., Zhu J., Liu Y., Zhou P.-K., Gu Y. (2024). Mechanisms of Radiation-Induced Tissue Damage and Response. MedComm.

[57] Jayakumar S., Pal D., Sandur S.K. (2015). Nrf2 Facilitates Repair of Radiation Induced DNA Damage Through Homologous Recombination Repair Pathway in a ROS Independent Manner in Cancer Cells. Mutat. Res. Fundam. Mol. Mech. Mutagen..

[58] Huang R.-X., Zhou P.-K. (2020). DNA Damage Response Signaling Pathways and Targets for Radiotherapy Sensitization in Cancer. Signal Transduct. Target. Ther..

[59] Nie S., He X., Sun Z., Zhang Y., Liu T., Chen T., Zhao J. (2023). Selenium Speciation-Dependent Cancer Radiosensitization by Induction of G2/M Cell Cycle Arrest and Apoptosis. Front. Bioeng. Biotechnol..

[60] Reisz J.A., Bansal N., Qian J., Zhao W., Furdui C.M. (2014). Effects of Ionizing Radiation on Biological Molecules—Mechanisms of Damage and Emerging Methods of Detection. Antioxid. Redox Signal..

[61] Vasin M.V. (2014). Comments on the Mechanisms of Action of Radiation Protective Agents: Basis Components and Their Polyvalence. SpringerPlus.

[62] Halliwell B., Gutteridge J.M. (1992). Biologically Relevant Metal Ion-Dependent Hydroxyl Radical Generation. An Update. FEBS Lett..

[63] Qian W., Zhang C., He L., Jin S., Suo R., Li Y., Li S., Zhu L., Deng K., Wu B. (2025). X-Ray Induced in-Situ Ferroptosis Through the Fenton Reaction of Iron Supplements for the Cancer Therapy. Bioorg. Chem..

[64] Mortezaee K., Goradel N.H., Amini P., Shabeeb D., Musa A.E., Najafi M., Farhood B. (2019). NADPH Oxidase as a Target for Modulation of Radiation Response; Implications to Carcinogenesis and Radiotherapy. Curr. Mol. Pharmacol..

[65] Pahl H.L. (1999). Activators and Target Genes of Rel/NF-κB Transcription Factors. Oncogene.

[66] Pradere J.-P., Dapito D.H., Schwabe R.F. (2014). The Yin and Yang of Toll-like Receptors in Cancer. Oncogene.

[67] Venkata Narayanan I., Paulsen M.T., Bedi K., Berg N., Ljungman E.A., Francia S., Veloso A., Magnuson B., di Fagagna F.d., Wilson T.E. (2017). Transcriptional and Post-Transcriptional Regulation of the Ionizing Radiation Response by ATM and P53. Sci. Rep..

[68] Kim J.H., Brown S.L., Gordon M.N. (2023). Radiation-Induced Senescence: Therapeutic Opportunities. Radiat. Oncol..

[69] Kim J.-S., Rhim K.-J., Jang W.-S., Lee S.-J., Son Y., Lee S.-S., Park S., Lim S.M. (2015). β-Irradiation (^166^Ho Patch)-Induced Skin Injury in Mini-Pigs: Effects on NF-κB and COX-2 Expression in the Skin. J. Vet. Sci..

[70] Kiang J.G., Smith J.T., Cannon G., Anderson M.N., Ho C., Zhai M., Cui W., Xiao M. (2020). Ghrelin, a Novel Therapy, Corrects Cytokine and NF-κB-AKT-MAPK Network and Mitigates Intestinal Injury Induced by Combined Radiation and Skin-Wound Trauma. Cell Biosci..

[71] Molteni A., Wolfe L.F., Ward W.F., Ts’ao C.H., Molteni L.B., Veno P., Fish B.L., Taylor J.M., Quintanilla N., Herndon B. (2007). Effect of an Angiotensin II Receptor Blocker and Two Angiotensin Converting Enzyme Inhibitors on Transforming Growth Factor-Beta (TGF-Beta) and Alpha-Actomyosin (Alpha SMA), Important Mediators of Radiation-Induced Pneumopathy and Lung Fibrosis. Curr. Pharm. Des..

[72] Khayyal M.T., El-Ghazaly M.A., El-Hazek R.M., Nada A.S. (2009). The Effects of Celecoxib, a COX-2 Selective Inhibitor, on Acute Inflammation Induced in Irradiated Rats. Inflammopharmacology.

[73] Shadyro O.I., Yurkova I.L., Kisel M.A. (2002). Radiation-Induced Peroxidation and Fragmentation of Lipids in a Model Membrane. Int. J. Radiat. Biol..

[74] Rosen E., Kryndushkin D., Aryal B., Gonzalez Y., Chehab L., Dickey J., Rao V.A. (2020). Acute Total Body Ionizing Gamma Radiation Induces Long-Term Adverse Effects and Immediate Changes in Cardiac Protein Oxidative Carbonylation in the Rat. PLoS ONE.

[75] Möller M.N., Denicola A. (2024). Diffusion of Peroxynitrite, Its Precursors, and Derived Reactive Species, and the Effect of Cell Membranes. Redox Biochem. Chem..

[76] Reuter S., Gupta S.C., Chaturvedi M.M., Aggarwal B.B. (2010). Oxidative Stress, Inflammation, and Cancer: How Are They Linked?. Free Radic. Biol. Med..

[77] Hayes J.D., Dinkova-Kostova A.T., Tew K.D. (2020). Oxidative Stress in Cancer. Cancer Cell.

[78] Li C., Xue Y., Ba X., Wang R. (2022). The Role of 8-oxoG Repair Systems in Tumorigenesis and Cancer Therapy. Cells.

[79] Yakes F.M., Van Houten B. (1997). Mitochondrial DNA Damage Is More Extensive and Persists Longer than Nuclear DNA Damage in Human Cells Following Oxidative Stress. Proc. Natl. Acad. Sci. USA.

[80] Kim Y.-C., Barshishat-Kupper M., McCart E.A., Mueller G.P., Day R.M. (2014). Bone Marrow Protein Oxidation in Response to Ionizing Radiation in C57BL/6J Mice. Proteomes.

[81] Ayala A., Muñoz M.F., Argüelles S. (2014). Lipid Peroxidation: Production, Metabolism, and Signaling Mechanisms of Malondialdehyde and 4-Hydroxy-2-Nonenal. Oxidative Med. Cell. Longev..

[82] Marnett L.J. (1999). Lipid Peroxidation-DNA Damage by Malondialdehyde. Mutat. Res..

[83] Edwards J.C., Chapman D., Cramp W.A., Yatvin M.B. (1984). The Effects of Ionizing Radiation on Biomembrane Structure and Function. Prog. Biophys. Mol. Biol..

[84] Milkovic L., Zarkovic N., Marusic Z., Zarkovic K., Jaganjac M. (2023). The 4-Hydroxynonenal–Protein Adducts and Their Biological Relevance: Are Some Proteins Preferred Targets?. Antioxidants.

[85] Dalleau S., Baradat M., Guéraud F., Huc L. (2013). Cell Death and Diseases Related to Oxidative Stress: 4-Hydroxynonenal (HNE) in the Balance. Cell Death Differ..

[86] Livingston K., Schlaak R.A., Puckett L.L., Bergom C. (2020). The Role of Mitochondrial Dysfunction in Radiation-Induced Heart Disease: From Bench to Bedside. Front. Cardiovasc. Med..

[87] Yamamori T., Yasui H., Yamazumi M., Wada Y., Nakamura Y., Nakamura H., Inanami O. (2012). Ionizing Radiation Induces Mitochondrial Reactive Oxygen Species Production Accompanied by Upregulation of Mitochondrial Electron Transport Chain Function and Mitochondrial Content Under Control of the Cell Cycle Checkpoint. Free Radic. Biol. Med..

[88] Lafargue A., Degorre C., Corre I., Alves-Guerra M.-C., Gaugler M.-H., Vallette F., Pecqueur C., Paris F. (2017). Ionizing Radiation Induces Long-Term Senescence in Endothelial Cells Through Mitochondrial Respiratory Complex II Dysfunction and Superoxide Generation. Free Radic. Biol. Med..

[89] Paris F., Fuks Z., Kang A., Capodieci P., Juan G., Ehleiter D., Haimovitz-Friedman A., Cordon-Cardo C., Kolesnick R. (2001). Endothelial Apoptosis as the Primary Lesion Initiating Intestinal Radiation Damage in Mice. Science.

[90] Cao X., Wen P., Fu Y., Gao Y., Qi X., Chen B., Tao Y., Wu L., Xu A., Lu H. (2019). Radiation Induces Apoptosis Primarily Through the Intrinsic Pathway in Mammalian Cells. Cell. Signal..

[91] Jomova K., Alomar S.Y., Alwasel S.H., Nepovimova E., Kuca K., Valko M. (2024). Several Lines of Antioxidant Defense Against Oxidative Stress: Antioxidant Enzymes, Nanomaterials with Multiple Enzyme-Mimicking Activities, and Low-Molecular-Weight Antioxidants. Arch. Toxicol..

[92] Gao F., Fish B.L., Szabo A., Doctrow S.R., Kma L., Molthen R.C., Moulder J.E., Jacobs E.R., Medhora M. (2012). Short-Term Treatment with a SOD/Catalase Mimetic, EUK-207, Mitigates Pneumonitis and Fibrosis After Single-Dose Total-Body or Whole-Thoracic Irradiation. Radiat. Res..

[93] Shrishrimal S., Chatterjee A., Kosmacek E.A., Davis P.J., McDonald J.T., Oberley-Deegan R.E. (2020). Manganese Porphyrin, MnTE-2-PyP, Treatment Protects the Prostate from Radiation-Induced Fibrosis (RIF) by Activating the NRF2 Signaling Pathway and Enhancing SOD2 and Sirtuin Activity. Free Radic. Biol. Med..

[94] Atia A., Alrawaiq N.S., Abdullah A. (2021). Tocotrienols Activate Nrf2 Nuclear Translocation and Increase the Antioxidant- Related Hepatoprotective Mechanism in Mice Liver. Curr. Pharm. Biotechnol..

[95] Guan C., Zhou X., Li H., Ma X., Zhuang J. (2022). NF-κB Inhibitors Gifted by Nature: The Anticancer Promise of Polyphenol Compounds. Biomed. Pharmacother..

[96] Liu Z., Deng P., Liu S., Bian Y., Xu Y., Zhang Q., Wang H., Pi J. (2023). Is Nuclear Factor Erythroid 2-Related Factor 2 a Target for the Intervention of Cytokine Storms?. Antioxidants.

[97] Kim J.-H., Thimmulappa R.K., Kumar V., Cui W., Kumar S., Kombairaju P., Zhang H., Margolick J., Matsui W., Macvittie T. (2014). NRF2-Mediated Notch Pathway Activation Enhances Hematopoietic Reconstitution Following Myelosuppressive Radiation. J. Clin. Investig..

[98] Adjemian S., Oltean T., Martens S., Wiernicki B., Goossens V., Vanden Berghe T., Cappe B., Ladik M., Riquet F.B., Heyndrickx L. (2020). Ionizing Radiation Results in a Mixture of Cellular Outcomes Including Mitotic Catastrophe, Senescence, Methuosis, and Iron-Dependent Cell Death. Cell Death Dis..

[99] Steinman J., Epperly M., Hou W., Willis J., Wang H., Fisher R., Liu B., Bahar I., McCaw T., Kagan V. (2017). Improved Total-Body Irradiation Survival by Delivery of Two Radiation Mitigators That Target Distinct Cell Death Pathways. Radiat. Res..

[100] Koturbash I., Loree J., Kutanzi K., Koganow C., Pogribny I., Kovalchuk O. (2008). In Vivo Bystander Effect: Cranial X-Irradiation Leads to Elevated DNA Damage, Altered Cellular Proliferation and Apoptosis, and Increased P53 Levels in Shielded Spleen. Int. J. Radiat. Oncol. Biol. Phys..

[101] Yang H., Huang F., Tao Y., Zhao X., Liao L., Tao X. (2017). Simvastatin Ameliorates Ionizing Radiation-Induced Apoptosis in the Thymus by Activating the AKT/Sirtuin 1 Pathway in Mice. Int. J. Mol. Med..

[102] Erpolat O.P., Demircan N.V., Sarıbas G.S., Kuzucu P., Senturk E., Elmas C., Borcek A., Kurt G. (2020). A Comparison of Ramipril and Bevacizumab to Mitigate Radiation-Induced Brain Necrosis: An Experimental Study. World Neurosurg..

[103] Wei M.-F., Cheng C.-H., Wen S.-Y., Lin J.-C., Chen Y.-H., Wang C.-W., Lee Y.-H., Kuo S.-H. (2022). Atorvastatin Attenuates Radiotherapy-Induced Intestinal Damage Through Activation of Autophagy and Antioxidant Effects. Oxidative Med. Cell. Longev..

[104] Kim H.J., Kang S.U., Lee Y.S., Jang J.Y., Kang H., Kim C.-H. (2020). Protective Effects of N-Acetylcysteine Against Radiation-Induced Oral Mucositis In Vitro and In Vivo. Cancer Res. Treat..

[105] Citrin D.E., Shankavaram U., Horton J.A., Shield W., Zhao S., Asano H., White A., Sowers A., Thetford A., Chung E.J. (2013). Role of Type II Pneumocyte Senescence in Radiation-Induced Lung Fibrosis. J. Natl. Cancer Inst..

[106] Nagane M., Yasui H., Kuppusamy P., Yamashita T., Inanami O. (2021). DNA Damage Response in Vascular Endothelial Senescence: Implication for Radiation-Induced Cardiovascular Diseases. J. Radiat. Res..

[107] Yue T., Dong Y., Huo Q., Li W., Wang X., Zhang S., Fan H., Wu X., He X., Zhao Y. (2024). Nicotinamide Riboside Alleviates Ionizing Radiation-Induced Intestinal Senescence by Alleviating Oxidative Damage and Regulating Intestinal Metabolism. J. Adv. Res..

[108] Nakano T., Xu X., Salem A.M.H., Shoulkamy M.I., Ide H. (2017). Radiation-Induced DNA–Protein Cross-Links: Mechanisms and Biological Significance. Free Radic. Biol. Med..

[109] Liu C., Wei J., Wang X., Zhao Q., Lv J., Tan Z., Xin Y., Jiang X. (2024). Radiation-Induced Skin Reactions: Oxidative Damage Mechanism and Antioxidant Protection. Front. Cell Dev. Biol..

[110] Daguenet E., Louati S., Wozny A.-S., Vial N., Gras M., Guy J.-B., Vallard A., Rodriguez-Lafrasse C., Magné N. (2020). Radiation-Induced Bystander and Abscopal Effects: Important Lessons from Preclinical Models. Br. J. Cancer.

[111] Siva S., Lobachevsky P., MacManus M.P., Kron T., Möller A., Lobb R.J., Ventura J., Best N., Smith J., Ball D. (2016). Radiotherapy for Non–Small Cell Lung Cancer Induces DNA Damage Response in Both Irradiated and Out-of-Field Normal Tissues. Clin. Cancer Res..

[112] Burdak-Rothkamm S., Rothkamm K. (2018). Radiation-Induced Bystander and Systemic Effects Serve as a Unifying Model System for Genotoxic Stress Responses. Mutat. Res. Rev. Mutat. Res..

[113] Tang H., Cai L., He X., Niu Z., Huang H., Hu W., Bian H., Huang H. (2023). Radiation-Induced Bystander Effect and Its Clinical Implications. Front. Oncol..

[114] Geras’kin S.A., Fesenko S.V., Alexakhin R.M. (2008). Effects of Non-Human Species Irradiation After the Chernobyl NPP Accident. Environ. Int..

[115] Mancuso M., Pasquali E., Leonardi S., Tanori M., Rebessi S., Di Majo V., Pazzaglia S., Toni M.P., Pimpinella M., Covelli V. (2008). Oncogenic Bystander Radiation Effects in Patched Heterozygous Mouse Cerebellum. Proc. Natl. Acad. Sci. USA.

[116] Grant E.J., Brenner A., Sugiyama H., Sakata R., Sadakane A., Utada M., Cahoon E.K., Milder C.M., Soda M., Cullings H.M. (2017). Solid Cancer Incidence Among the Life Span Study of Atomic Bomb Survivors: 1958–2009. Radiat. Res..

[117] Guan X., Wei R., Yang R., Lu Z., Liu E., Zhao Z., Chen H., Yang M., Liu Z., Jiang Z. (2021). Association of Radiotherapy for Rectal Cancer and Second Gynecological Malignant Neoplasms. JAMA Netw. Open.

[118] Chai Y., Calaf G.M., Zhou H., Ghandhi S.A., Elliston C.D., Wen G., Nohmi T., Amundson S.A., Hei T.K. (2013). Radiation Induced COX-2 Expression and Mutagenesis at Non-Targeted Lung Tissues of Gpt Delta Transgenic Mice. Br. J. Cancer.

[119] Kaminaga K., Noguchi M., Narita A., Hattori Y., Usami N., Yokoya A. (2016). Cell Cycle Tracking for Irradiated and Unirradiated Bystander Cells in a Single Colony with Exposure to a Soft X-Ray Microbeam. Int. J. Radiat. Biol..

[120] Xu S., Wang J., Ding N., Hu W., Zhang X., Wang B., Hua J., Wei W., Zhu Q. (2015). Exosome-Mediated microRNA Transfer Plays a Role in Radiation-Induced Bystander Effect. RNA Biol..

[121] Zhou H., Ivanov V.N., Gillespie J., Geard C.R., Amundson S.A., Brenner D.J., Yu Z., Lieberman H.B., Hei T.K. (2005). Mechanism of Radiation-Induced Bystander Effect: Role of the Cyclooxygenase-2 Signaling Pathway. Proc. Natl. Acad. Sci. USA.

[122] Belloni P., Latini P., Palitti F. (2011). Radiation-Induced Bystander Effect in Healthy G(o) Human Lymphocytes: Biological and Clinical Significance. Mutat. Res..

[123] Peter C., Wesselborg S., Herrmann M., Lauber K. (2010). Dangerous Attraction: Phagocyte Recruitment and Danger Signals of Apoptotic and Necrotic Cells. Apoptosis.

[124] Hu Z.I., Ho A.Y., McArthur H.L. (2017). Combined Radiation Therapy and Immune Checkpoint Blockade Therapy for Breast Cancer. Int. J. Radiat. Oncol. Biol. Phys..

[125] Pouget J.-P., Georgakilas A.G., Ravanat J.-L. (2018). Targeted and Off-Target (Bystander and Abscopal) Effects of Radiation Therapy: Redox Mechanisms and Risk/Benefit Analysis. Antioxid. Redox Signal..

[126] Mole R.H. (1953). Whole Body Irradiation; Radiobiology or Medicine?. Br. J. Radiol..

[127] Chakraborty M., Abrams S.I., Camphausen K., Liu K., Scott T., Coleman C.N., Hodge J.W. (2003). Irradiation of Tumor Cells Up-Regulates Fas and Enhances CTL Lytic Activity and CTL Adoptive Immunotherapy. J. Immunol..

[128] Reits E.A., Hodge J.W., Herberts C.A., Groothuis T.A., Chakraborty M., K.Wansley E., Camphausen K., Luiten R.M., de Ru A.H., Neijssen J. (2006). Radiation Modulates the Peptide Repertoire, Enhances MHC Class I Expression, and Induces Successful Antitumor Immunotherapy. J. Exp. Med..

[129] Frey B., Rubner Y., Kulzer L., Werthmöller N., Weiss E.-M., Fietkau R., Gaipl U.S. (2014). Antitumor Immune Responses Induced by Ionizing Irradiation and Further Immune Stimulation. Cancer Immunol. Immunother..

[130] Nabrinsky E., Macklis J., Bitran J. (2022). A Review of the Abscopal Effect in the Era of Immunotherapy. Cureus.

[131] Demaria S., Ng B., Devitt M.L., Babb J.S., Kawashima N., Liebes L., Formenti S.C. (2004). Ionizing Radiation Inhibition of Distant Untreated Tumors (Abscopal Effect) Is Immune Mediated. Int. J. Radiat. Oncol. Biol. Phys..

[132] Rodríguez-Ruiz M.E., Vanpouille-Box C., Melero I., Formenti S.C., Demaria S. (2018). Immunological Mechanisms Responsible for Radiation-Induced Abscopal Effect. Trends Immunol..

[133] Abuodeh Y., Venkat P., Kim S. (2016). Systematic Review of Case Reports on the Abscopal Effect. Curr. Probl. Cancer.

[134] Formenti S.C., Demaria S. (2009). Systemic Effects of Local Radiotherapy. Lancet Oncol..

[135] von Essen C.F. (1991). Radiation Enhancement of Metastasis: A Review. Clin. Exp. Metastasis.

[136] Siva S., MacManus M.P., Martin R.F., Martin O.A. (2015). Abscopal Effects of Radiation Therapy: A Clinical Review for the Radiobiologist. Cancer Lett..

[137] Soukup K., Wang X. (2015). Radiation Meets Immunotherapy—A Perfect Match in the Era of Combination Therapy?. Int. J. Radiat. Biol..

[138] Hatten S.J., Lehrer E.J., Liao J., Sha C.M., Trifiletti D.M., Siva S., McBride S.M., Palma D., Holder S.L., Zaorsky N.G. (2022). A Patient-Level Data Meta-Analysis of the Abscopal Effect. Adv. Radiat. Oncol..

[139] Ngwa W., Irabor O.C., Schoenfeld J.D., Hesser J., Demaria S., Formenti S.C. (2018). Using Immunotherapy to Boost the Abscopal Effect. Nat. Rev. Cancer.

[140] Wang X., Zhang H., Zhang X., Liu Y. (2024). Abscopal Effect: From a Rare Phenomenon to a New Frontier in Cancer Therapy. Biomark. Res..

[141] Ng J., Dai T. (2016). Radiation Therapy and the Abscopal Effect: A Concept Comes of Age. Ann. Transl. Med..

[142] Postow M.A., Callahan M.K., Barker C.A., Yamada Y., Yuan J., Kitano S., Mu Z., Rasalan T., Adamow M., Ritter E. (2012). Immunologic Correlates of the Abscopal Effect in a Patient with Melanoma. N. Engl. J. Med..

[143] Liu Y., Dong Y., Kong L., Shi F., Zhu H., Yu J. (2018). Abscopal Effect of Radiotherapy Combined with Immune Checkpoint Inhibitors. J. Hematol. Oncol..

[144] Zhao X., Shao C. (2020). Radiotherapy-Mediated Immunomodulation and Anti-Tumor Abscopal Effect Combining Immune Checkpoint Blockade. Cancers.

[145] Golden E.B., Chhabra A., Chachoua A., Adams S., Donach M., Fenton-Kerimian M., Friedman K., Ponzo F., Babb J.S., Goldberg J. (2015). Local Radiotherapy and Granulocyte-Macrophage Colony-Stimulating Factor to Generate Abscopal Responses in Patients with Metastatic Solid Tumours: A Proof-of-Principle Trial. Lancet Oncol..

[146] Talapko J., Talapko D., Katalinić D., Kotris I., Erić I., Belić D., Vasilj Mihaljević M., Vasilj A., Erić S., Flam J. (2024). Health Effects of Ionizing Radiation on the Human Body. Medicina.

[147] Paganetti H. (2023). A Review on Lymphocyte Radiosensitivity and Its Impact on Radiotherapy. Front. Oncol..

[148] Blakely W.F., Port M., Abend M. (2021). Early-Response Multiple-Parameter Biodosimetry and Dosimetry: Risk Predictions. J. Radiol. Prot..

[149] DiCarlo A.L., Kaminski J.M., Hatchett R.J., Maidment B.W. (2016). Role of Thrombocytopenia in Radiation-Induced Mortality and Review of Therapeutic Approaches Targeting Platelet Regeneration After Radiation Exposure. J. Radiat. Oncol..

[150] Waselenko J.K., MacVittie T.J., Blakely W.F., Pesik N., Wiley A.L., Dickerson W.E., Tsu H., Confer D.L., Coleman C.N., Seed T. (2004). Medical Management of the Acute Radiation Syndrome: Recommendations of the Strategic National Stockpile Radiation Working Group. Ann. Intern. Med..

[151] Damen P.J.J., Kroese T.E., van Hillegersberg R., Schuit E., Peters M., Verhoeff J.J.C., Lin S.H., van Rossum P.S.N. (2021). The Influence of Severe Radiation-Induced Lymphopenia on Overall Survival in Solid Tumors: A Systematic Review and Meta-Analysis. Int. J. Radiat. Oncol. Biol. Phys..

[152] Carr K.E. (2001). Effects of Radiation Damage on Intestinal Morphology. Int. Rev. Cytol..

[153] Freeman M.L. (2025). Gastrointestinal Acute Radiation Syndrome: Current Knowledge and Perspectives. Cell Death Discov..

[154] Schuette W., Krzakowski M.J., Massuti B., Otterson G.A., Lizambri R., Wei H., Berger D.P., Chen Y. (2012). Randomized Phase II Study of Palifermin for Reducing Dysphagia in Patients Receiving Concurrent Chemoradiotherapy for Locally Advanced Unresectable Non-Small Cell Lung Cancer. J. Thorac. Oncol..

[155] Zheng Z., Zhao X., Zhao Q., Zhang Y., Liu S., Liu Z., Meng L., Xin Y., Jiang X. (2021). The Effects of Early Nutritional Intervention on Oral Mucositis and Nutritional Status of Patients with Head and Neck Cancer Treated with Radiotherapy. Front. Oncol..

[156] Xia J., Tao X., Hu Q., Luo W., Tong X., Zhou G., Zhou H., Hua H., Tang G., Wu T. (2025). Expert Consensus on the Prevention and Treatment of Radiochemotherapy-Induced Oral Mucositis. Int. J. Oral Sci..

[157] Peterson D.E., Boers-Doets C.B., Bensadoun R.J., Herrstedt J. (2015). Management of Oral and Gastrointestinal Mucosal Injury: ESMO Clinical Practice Guidelines for Diagnosis, Treatment, and Follow-Up. Ann. Oncol..

[158] Ibrahim S.S., Hassanein F.E.A., Zaky H.W., Gamal H. (2024). Clinical and Biochemical Assessment of the Effect of Glutamine in Management of Radiation Induced Oral Mucositis in Patients with Head and Neck Cancer: Randomized Controlled Clinical Trial. J. Stomatol. Oral Maxillofac. Surg..

[159] Cotrim A.P., Sowers A.L., Lodde B.M., Vitolo J.M., Kingman A., Russo A., Mitchell J.B., Baum B.J. (2005). Kinetics of Tempol for Prevention of Xerostomia Following Head and Neck Irradiation in a Mouse Model. Clin. Cancer Res..

[160] Chung M.K., Kim D.H., Ahn Y.C., Choi J.Y., Kim E.H., Son Y.-I. (2016). Randomized Trial of Vitamin C/E Complex for Prevention of Radiation-Induced Xerostomia in Patients with Head and Neck Cancer. Otolaryngol. Head Neck Surg..

[161] Kim S., Kim J., Jeon E., Park S., Park J., Choi J. (2025). Preventive Effect of Metformin in Radiation-Induced Xerostomia. Adv. Biol..

[162] Pathak S., Soni T.P., Sharma L.M., Patni N., Gupta A.K. (2019). A Randomized Controlled Trial to Evaluate the Role and Efficacy of Oral Glutamine in the Treatment of Chemo-Radiotherapy-Induced Oral Mucositis and Dysphagia in Patients with Oropharynx and Larynx Carcinoma. Cureus.

[163] Sayed R., El Wakeel L., Saad A.S., Kelany M., El-Hamamsy M. (2019). Pentoxifylline and Vitamin E Reduce the Severity of Radiotherapy-Induced Oral Mucositis and Dysphagia in Head and Neck Cancer Patients: A Randomized, Controlled Study. Med. Oncol..

[164] Gorbunov N.V., Kiang J.G. (2021). Brain Damage and Patterns of Neurovascular Disorder After Ionizing Irradiation. Complications in Radiotherapy and Radiation Combined Injury. Radiat. Res..

[165] Singh V.K., Seed T.M. (2020). Pharmacological Management of Ionizing Radiation Injuries: Current and Prospective Agents and Targeted Organ Systems. Expert Opin. Pharmacother..

[166] Baselet B., Sonveaux P., Baatout S., Aerts A. (2018). Pathological Effects of Ionizing Radiation: Endothelial Activation and Dysfunction. Cell. Mol. Life Sci..

[167] Zhuang H., Shi S., Yuan Z., Chang J.Y. (2019). Bevacizumab Treatment for Radiation Brain Necrosis: Mechanism, Efficacy and Issues. Mol. Cancer.

[168] Shi L., Du F.-L., Sun Z.-W., Zhang L., Chen Y.-Y., Xie T.-M., Li P.-J., Huang S., Dong B.-Q., Zhang M.-M. (2018). Radiation-Induced Gray Matter Atrophy in Patients with Nasopharyngeal Carcinoma After Intensity Modulated Radiotherapy: A MRI Magnetic Resonance Imaging Voxel-Based Morphometry Study. Quant. Imaging Med. Surg..

[169] Jaschke W., Schmuth M., Trianni A., Bartal G. (2017). Radiation-Induced Skin Injuries to Patients: What the Interventional Radiologist Needs to Know. Cardiovasc. Interv. Radiol..

[170] Iddins C.J., DiCarlo A.L., Ervin M.D., Herrera-Reyes E., Goans R.E. (2022). Cutaneous and Local Radiation Injuries. J. Radiol. Prot..

[171] Peter R.U., Braun-Falco O., Birioukov A., Hacker N., Kerscher M., Peterseim U., Ruzicka T., Konz B., Plewig G. (1994). Chronic Cutaneous Damage After Accidental Exposure to Ionizing Radiation: The Chernobyl Experience. J. Am. Acad. Dermatol..

[172] Fuzissaki M.d.A., Paiva C.E., Oliveira M.A.d., Lajolo Canto P.P., Paiva Maia Y.C. (2019). de The Impact of Radiodermatitis on Breast Cancer Patients’ Quality of Life During Radiotherapy: A Prospective Cohort Study. J. Pain Symptom Manag..

[173] Ryan Wolf J., Gewandter J.S., Bautista J., Heckler C.E., Strasser J., Dyk P., Anderson T., Gross H., Speer T., Dolohanty L. (2020). Utility of Topical Agents for Radiation Dermatitis and Pain: A Randomized Clinical Trial. Support. Care Cancer.

[174] Rübe C.E., Freyter B.M., Tewary G., Roemer K., Hecht M., Rübe C. (2024). Radiation Dermatitis: Radiation-Induced Effects on the Structural and Immunological Barrier Function of the Epidermis. Int. J. Mol. Sci..

[175] Kao Y.-S., Ma K.S.-K., Wu M.-Y., Wu Y.-C., Tu Y.-K., Hung C.-H. (2022). Topical Prevention of Radiation Dermatitis in Head and Neck Cancer Patients: A Network Meta-Analysis. In Vivo.

[176] Cui J., Wang T.-J., Zhang Y.-X., She L.-Z., Zhao Y.-C. (2024). Molecular Biological Mechanisms of Radiotherapy-Induced Skin Injury Occurrence and Treatment. Biomed. Pharmacother..

[177] Goertz O., Poettgen C., Akbari A., Kolbenschlag J., Langer S., Lehnhardt M., Stuschke M., von der Lohe L. (2015). New Model for Long-Term Investigations of Cutaneous Microcirculatory and Inflammatory Changes Following Irradiation. J. Radiat. Res..

[178] Behroozian T., Bonomo P., Patel P., Kanee L., Finkelstein S., van den Hurk C., Chow E., Wolf J.R., Multinational Association of Supportive Care in Cancer (MASCC) Oncodermatology Study Group Radiation Dermatitis Guidelines Working Group (2023). Multinational Association of Supportive Care in Cancer (MASCC) Clinical Practice Guidelines for the Prevention and Management of Acute Radiation Dermatitis: International Delphi Consensus-Based Recommendations. Lancet Oncol..

[179] Tam S., Zhou G., Trombetta M., Caini S., Ryan Wolf J., van den Hurk C., Beveridge M., Lam H., Bonomo P., Chow E. (2023). Topical Corticosteroids for the Prevention of Severe Radiation Dermatitis: A Systematic Review and Meta-Analysis. Support. Care Cancer.

[180] Dainiak N., Albanese J. (2022). Assessment and Clinical Management of Internal Contamination. J. Radiol. Prot..

[181] MacVittie T.J., Farese A.M., Parker G.A., Jackson W. (2019). The Time Course of Radiation-Induced Lung Injury in a Nonhuman Primate Model of Partial-Body Irradiation with Minimal Bone Marrow Sparing: Clinical and Radiographic Evidence and the Effect of Neupogen Administration. Health Phys..

[182] Arroyo-Hernández M., Maldonado F., Lozano-Ruiz F., Muñoz-Montaño W., Nuñez-Baez M., Arrieta O. (2021). Radiation-Induced Lung Injury: Current Evidence. BMC Pulm. Med..

[183] Mettler F.A., Gus’kova A.K., Gusev I. (2007). Health Effects in Those with Acute Radiation Sickness from the Chernobyl Accident. Health Phys..

[184] Palma D.A., Senan S., Tsujino K., Barriger R.B., Rengan R., Moreno M., Bradley J.D., Kim T.H., Ramella S., Marks L.B. (2013). Predicting Radiation Pneumonitis After Chemoradiation Therapy for Lung Cancer: An International Individual Patient Data Meta-Analysis. Int. J. Radiat. Oncol. Biol. Phys..

[185] Medhora M., Gao F., Fish B.L., Jacobs E.R., Moulder J.E., Szabo A. (2012). Dose-Modifying Factor for Captopril for Mitigation of Radiation Injury to Normal Lung. J. Radiat. Res..

[186] Fleckenstein K., Zgonjanin L., Chen L., Rabbani Z., Jackson I.L., Thrasher B., Kirkpatrick J., Foster W.M., Vujaskovic Z. (2007). Temporal Onset of Hypoxia and Oxidative Stress After Pulmonary Irradiation. Int. J. Radiat. Oncol. Biol. Phys..

[187] Käsmann L., Dietrich A., Staab-Weijnitz C.A., Manapov F., Behr J., Rimner A., Jeremic B., Senan S., De Ruysscher D., Lauber K. (2020). Radiation-Induced Lung Toxicity—Cellular and Molecular Mechanisms of Pathogenesis, Management, and Literature Review. Radiat. Oncol..

[188] Arpin D., Perol D., Blay J.-Y., Falchero L., Claude L., Vuillermoz-Blas S., Martel-Lafay I., Ginestet C., Alberti L., Nosov D. (2005). Early Variations of Circulating Interleukin-6 and Interleukin-10 Levels During Thoracic Radiotherapy Are Predictive for Radiation Pneumonitis. J. Clin. Oncol..

[189] Mungunsukh O., George J., McCart E.A., Snow A.L., Mattapallil J.J., Mog S.R., Panganiban R.A.M., Bolduc D.L., Rittase W.B., Bouten R.M. (2021). Captopril Reduces Lung Inflammation and Accelerated Senescence in Response to Thoracic Radiation in Mice. J. Radiat. Res..

[190] Xu Y., Huang Y., Chen Y., Cao K., Liu Z., Wan Z., Liao Z., Li B., Cui J., Yang Y. (2021). Grape Seed Proanthocyanidins Play the Roles of Radioprotection on Normal Lung and Radiosensitization on Lung Cancer via Differential Regulation of the MAPK Signaling Pathway. J. Cancer.

[191] Fijardo M., Kwan J.Y.Y., Bissey P.-A., Citrin D.E., Yip K.W., Liu F.-F. (2024). The Clinical Manifestations and Molecular Pathogenesis of Radiation Fibrosis. eBioMedicine.

[192] Chen Z., Wu Z., Ning W. (2018). Advances in Molecular Mechanisms and Treatment of Radiation-Induced Pulmonary Fibrosis. Transl. Oncol..

[193] Ruysscher D.D., Wauters E., Jendrossek V., Filippi A.R., Revel M.-P., Faivre-Finn C., Naidoo J., Ramella S., Guckenberger M., Ricardi U. (2025). Diagnosis and Treatment of Radiation Induced Pneumonitis in Patients with Lung Cancer: An ESTRO Clinical Practice Guideline. Radiother. Oncol..

[194] Ran X.-Z., Shi C.-M., Zheng H.-E., Su Y.-P., Cheng T.-M. (2011). Experimental Research on the Management of Combined Radiation-Burn Injury in China. Radiat. Res..

[195] Williams J.P., McBride W.H. (2011). After the Bomb Drops: A New Look at Radiation-Induced Multiple Organ Dysfunction Syndrome (MODS). Int. J. Radiat. Biol..

[196] Molinar-Inglis O., DiCarlo A.L., Lapinskas P.J., Rios C.I., Satyamitra M.M., Silverman T.A., Winters T.A., Cassatt D.R. (2024). Radiation-Induced Multi-Organ Injury. Int. J. Radiat. Biol..

[197] Wu T., Orschell C.M. (2023). The Delayed Effects of Acute Radiation Exposure (DEARE): Characteristics, Mechanisms, Animal Models, and Promising Medical Countermeasures. Int. J. Radiat. Biol..

[198] Preston D.L., Shimizu Y., Pierce D.A., Suyama A., Mabuchi K. (2003). Studies of Mortality of Atomic Bomb Survivors. Report 13: Solid Cancer and Noncancer Disease Mortality: 1950-1997. Radiat. Res..

[199] Little M.P. (2009). Cancer and Non-Cancer Effects in Japanese Atomic Bomb Survivors. J. Radiol. Prot..

[200] Ozasa K., Shimizu Y., Suyama A., Kasagi F., Soda M., Grant E.J., Sakata R., Sugiyama H., Kodama K. (2012). Studies of the Mortality of Atomic Bomb Survivors, Report 14, 1950-2003: An Overview of Cancer and Noncancer Diseases. Radiat. Res..

[201] Berrington de Gonzalez A., Gilbert E., Curtis R., Inskip P., Kleinerman R., Morton L., Rajaraman P., Little M.P. (2013). Second Solid Cancers After Radiation Therapy: A Systematic Review of the Epidemiologic Studies of the Radiation Dose-Response Relationship. Int. J. Radiat. Oncol. Biol. Phys..

[202] Kamiya K., Ozasa K., Akiba S., Niwa O., Kodama K., Takamura N., Zaharieva E.K., Kimura Y., Wakeford R. (2015). Long-Term Effects of Radiation Exposure on Health. Lancet.

[203] Duane F.K., Boekel N.B., Jacobse J.N., Wang Z., Aleman B.M.P., Darby S.C., Schaapveld M., van Leeuwen F.E., Baaijens M.H.A., Warren S. (2022). Exposure of the Heart and Cardiac Valves in Women Irradiated for Breast Cancer 1970–2009. Clin. Transl. Radiat. Oncol..

[204] Hauptmann M., Byrnes G., Cardis E., Bernier M.-O., Blettner M., Dabin J., Engels H., Istad T.S., Johansen C., Kaijser M. (2023). Brain Cancer After Radiation Exposure from CT Examinations of Children and Young Adults: Results from the EPI-CT Cohort Study. Lancet Oncol..

[205] Preston D.L., Cullings H., Suyama A., Funamoto S., Nishi N., Soda M., Mabuchi K., Kodama K., Kasagi F., Shore R.E. (2008). Solid Cancer Incidence in Atomic Bomb Survivors Exposed in Utero or as Young Children. J. Natl. Cancer Inst..

[206] Saada M., Sanchez-Jimenez E., Roguin A. (2023). Risk of Ionizing Radiation in Pregnancy: Just a Myth or a Real Concern?. Europace.

[207] Williams P.M., Fletcher S. (2010). Health Effects of Prenatal Radiation Exposure. Am. Fam. Physician.

[208] Tsou M.-W., Liu J.-T., Hammitt J.K., Lu C.-H., Kao S.-Y.Z. (2020). The Effect of Prenatal Exposure to Radiation on Birth Outcomes: Exploiting a Natural Experiment in Taiwan. Jpn. Econ. Rev..

[209] van Heijst J.W.J., Ceberio I., Lipuma L.B., Samilo D.W., Wasilewski G.D., Gonzales A.M.R., Nieves J.L., van den Brink M.R.M., Perales M.A., Pamer E.G. (2013). Quantitative Assessment of T Cell Repertoire Recovery After Hematopoietic Stem Cell Transplantation. Nat. Med..

[210] Chua H.L., Plett P.A., Fisher A., Sampson C.H., Vemula S., Feng H., Sellamuthu R., Wu T., MacVittie T.J., Orschell C.M. (2019). Lifelong Residual Bone Marrow Damage in Murine Survivors of the Hematopoietic Acute Radiation Syndrome (H-ARS): A Compilation of Studies Comprising the Indiana University Experience. Health Phys..

[211] Fike J.R., Rosi S., Limoli C.L. (2009). Neural Precursor Cells and Central Nervous System Radiation Sensitivity. Semin. Radiat. Oncol..

[212] Gondi V., Hermann B.P., Mehta M.P., Tomé W.A. (2012). Hippocampal Dosimetry Predicts Neurocognitive Function Impairment After Fractionated Stereotactic Radiotherapy for Benign or Low-Grade Adult Brain Tumors. Int. J. Radiat. Oncol. Biol. Phys..

[213] Ma T.M., Grimm J., McIntyre R., Anderson-Keightly H., Kleinberg L.R., Hales R.K., Moore J., Vannorsdall T., Redmond K.J. (2017). A Prospective Evaluation of Hippocampal Radiation Dose Volume Effects and Memory Deficits Following Cranial Irradiation. Radiother. Oncol..

[214] Chakraborti A., Allen A., Allen B., Rosi S., Fike J.R. (2012). Cranial Irradiation Alters Dendritic Spine Density and Morphology in the Hippocampus. PLoS ONE.

[215] Ashpole N.M., Warrington J.P., Mitschelen M.C., Yan H., Sosnowska D., Gautam T., Farley J.A., Csiszar A., Ungvari Z., Sonntag W.E. (2014). Systemic Influences Contribute to Prolonged Microvascular Rarefaction After Brain Irradiation: A Role for Endothelial Progenitor Cells. Am. J. Physiol. Heart Circ. Physiol..

[216] Chen H., Chong Z.Z., De Toledo S.M., Azzam E.I., Elkabes S., Souayah N. (2016). Delayed Activation of Human Microglial Cells by High Dose Ionizing Radiation. Brain Res..

[217] Andrews R.N., Caudell D.L., Metheny-Barlow L.J., Peiffer A.M., Tooze J.A., Bourland J.D., Hampson R.E., Deadwyler S.A., Cline J.M. (2018). Fibronectin Produced by Cerebral Endothelial and Vascular Smooth Muscle Cells Contributes to Perivascular Extracellular Matrix in Late-Delayed Radiation-Induced Brain Injury. Radiat. Res..

[218] Hart E., Odé Z., Derieppe M.P.P., Groenink L., Heymans M.W., Otten R., Lequin M.H., Janssens G.O.R., Hoving E.W., van Vuurden D.G. (2022). Blood-Brain Barrier Permeability Following Conventional Photon Radiotherapy—A Systematic Review and Meta-Analysis of Clinical and Preclinical Studies. Clin. Transl. Radiat. Oncol..

[219] Liu C.-W., Yang H.-C., Chiang C.-L., Shen C.-I., Wu H.-M., Luo Y.-H., Hu Y.-S., Lin C.-J., Chung W.-Y., Shiau C.-Y. (2023). Leukoencephalopathy in Patients with Brain Metastases Who Received Radiosurgery with or Without Whole Brain Radiotherapy. J. Neuro-Oncol..

[220] Pospisil P., Hynkova L., Hnidakova L., Maistryszinova J., Slampa P., Kazda T. (2024). Unilateral Hippocampal Sparing During Whole Brain Radiotherapy for Multiple Brain Metastases: Narrative and Critical Review. Front. Oncol..

[221] Monje M.L., Toda H., Palmer T.D. (2003). Inflammatory Blockade Restores Adult Hippocampal Neurogenesis. Science.

[222] Robbins M.E., Payne V., Tommasi E., Diz D.I., Hsu F.-C., Brown W.R., Wheeler K.T., Olson J., Zhao W. (2009). The AT1 Receptor Antagonist, L-158,809, Prevents or Ameliorates Fractionated Whole-Brain Irradiation–Induced Cognitive Impairment. Int. J. Radiat. Oncol. Biol. Phys..

[223] Acharya M.M., Rosi S., Jopson T., Limoli C.L. (2015). Human Neural Stem Cell Transplantation Provides Long-Term Restoration of Neuronal Plasticity in the Irradiated Hippocampus. Cell Transplant..

[224] Baulch J.E., Acharya M.M., Allen B.D., Ru N., Chmielewski N.N., Martirosian V., Giedzinski E., Syage A., Park A.L., Benke S.N. (2016). Cranial Grafting of Stem Cell-Derived Microvesicles Improves Cognition and Reduces Neuropathology in the Irradiated Brain. Proc. Natl. Acad. Sci. USA.

[225] Raber J., Davis M.J., Pfankuch T., Rosenthal R., Doctrow S.R., Moulder J.E. (2017). Mitigating Effect of EUK-207 on Radiation-Induced Cognitive Impairments. Behav. Brain Res..

[226] Zhang Y., Gao L., Cheng Z., Cai J., Niu Y., Meng W., Zhao Q. (2017). Kukoamine A Prevents Radiation-Induced Neuroinflammation and Preserves Hippocampal Neurogenesis in Rats by Inhibiting Activation of NF-κB and AP-1. Neurotox. Res..

[227] Xu X., Huang H., Tu Y., Sun J., Xiong Y., Ma C., Qin S., Hu W., Zhou J. (2021). Celecoxib Alleviates Radiation-Induced Brain Injury in Rats by Maintaining the Integrity of Blood-Brain Barrier. Dose-Response.

[228] Cheng J., Jiang J., He B., Lin W.-J., Li Y., Duan J., Li H., Huang X., Cai J., Xie J. (2023). A Phase 2 Study of Thalidomide for the Treatment of Radiation-Induced Blood-Brain Barrier Injury. Sci. Transl. Med..

[229] Goetz A., Smith S., Dressler E., Page B.R., Wefel J.S., Rapp S.R., Ip E., Gilbert M., Pugh S., Sumrall A.L. (2025). WF-1801: Cognitive Outcomes of a Single Arm Pilot Study of Ramipril for Prevention of Radiation-Induced Cognitive Decline in Glioblastoma Patients Receiving Chemoradiotherapy. Int. J. Radiat. Oncol. Biol. Phys..

[230] Zhang H., Han G., Liu H., Chen J., Ji X., Zhou F., Zhou Y., Xie C. (2011). The Development of Classically and Alternatively Activated Macrophages Has Different Effects on the Varied Stages of Radiation-Induced Pulmonary Injury in Mice. J. Radiat. Res..

[231] Venkatesulu B.P., Mahadevan L.S., Aliru M.L., Yang X., Bodd M.H., Singh P.K., Yusuf S.W., Abe J.-I., Krishnan S. (2018). Radiation-Induced Endothelial Vascular Injury: A Review of Possible Mechanisms. JACC Basic Transl. Sci..

[232] Beach C., MacLean D., Majorova D., Arnold J.N., Olcina M.M. (2022). The Effects of Radiation Therapy on the Macrophage Response in Cancer. Front. Oncol..

[233] Damm R., Pech M., Haag F., Cavalli P., Gylstorff S., Omari J., Seidensticker R., Ricke J., Seidensticker M., Relja B. (2022). TNF-α Indicates Radiation-Induced Liver Injury After Interstitial High Dose-Rate Brachytherapy. In Vivo.

[234] Rube C.E., Uthe D., Schmid K.W., Richter K.D., Wessel J., Schuck A., Willich N., Rube C. (2000). Dose-Dependent Induction of Transforming Growth Factor Beta (TGF-Beta) in the Lung Tissue of Fibrosis-Prone Mice After Thoracic Irradiation. Int. J. Radiat. Oncol. Biol. Phys..

[235] Park H.-R., Jo S.-K., Jung U. (2019). Ionizing Radiation Promotes Epithelial–to–Mesenchymal Transition in Lung Epithelial Cells by TGF-β-Producing M2 Macrophages. In Vivo.

[236] Zhang S.-M., Wei C.-Y., Wang Q., Wang L., Lu L., Qi F.-Z. (2021). M2-Polarized Macrophages Mediate Wound Healing by Regulating Connective Tissue Growth Factor via AKT, ERK1/2, and STAT3 Signaling Pathways. Mol. Biol. Rep..

[237] Sheng Y., Zhang B., Xing B., Liu Z., Chang Y., Wu G., Zhao Y. (2023). Cancer-Associated Fibroblasts Exposed to High-Dose Ionizing Radiation Promote M2 Polarization of Macrophages, Which Induce Radiosensitivity in Cervical Cancer. Cancers.

[238] Lee W.H., Cho H.J., Sonntag W.E., Lee Y.W. (2011). Radiation Attenuates Physiological Angiogenesis by Differential Expression of VEGF, Ang-1, Tie-2 and Ang-2 in Rat Brain. Radiat. Res..

[239] Dadrich M., Nicolay N.H., Flechsig P., Bickelhaupt S., Hoeltgen L., Roeder F., Hauser K., Tietz A., Jenne J., Lopez R. (2016). Combined Inhibition of TGFβ and PDGF Signaling Attenuates Radiation-Induced Pulmonary Fibrosis. Oncoimmunology.

[240] Ahamed J., Laurence J. (2017). Role of Platelet-Derived Transforming Growth Factor-Β1 and Reactive Oxygen Species in Radiation-Induced Organ Fibrosis. Antioxid. Redox Signal..

[241] Bickelhaupt S., Erbel C., Timke C., Wirkner U., Dadrich M., Flechsig P., Tietz A., Pföhler J., Gross W., Peschke P. (2017). Effects of CTGF Blockade on Attenuation and Reversal of Radiation-Induced Pulmonary Fibrosis. J. Natl. Cancer Inst..

[242] Ip W.K.E., Hoshi N., Shouval D.S., Snapper S., Medzhitov R. (2017). Anti-Inflammatory Effect of IL-10 Mediated by Metabolic Reprogramming of Macrophages. Science.

[243] de Andrade C.B.V., Ramos I.P.R., de Moraes A.C.N., do Nascimento A.L.R., Salata C., Goldenberg R.C.d.S., de Carvalho J.J., de Almeida C.E.V. (2017). Radiotherapy-Induced Skin Reactions Induce Fibrosis Mediated by TGF-Β1 Cytokine. Dose-Response.

[244] Gauldie J., Bonniaud P., Sime P., Ask K., Kolb M. (2007). TGF-β, Smad3 and the Process of Progressive Fibrosis. Biochem. Soc. Trans..

[245] Finnson K.W., Almadani Y., Philip A. (2020). Non-Canonical (Non-SMAD2/3) TGF-β Signaling in Fibrosis: Mechanisms and Targets. Semin. Cell Dev. Biol..

[246] Richter K., Konzack A., Pihlajaniemi T., Heljasvaara R., Kietzmann T. (2015). Redox-Fibrosis: Impact of TGFβ1 on ROS Generators, Mediators and Functional Consequences. Redox Biol..

[247] Meng X., Nikolic-Paterson D.J., Lan H.Y. (2016). TGF-β: The Master Regulator of Fibrosis. Nat. Rev. Nephrol..

[248] Ying H., Fang M., Hang Q.Q., Chen Y., Qian X., Chen M. (2021). Pirfenidone Modulates Macrophage Polarization and Ameliorates Radiation-Induced Lung Fibrosis by Inhibiting the TGF-Β1/Smad3 Pathway. J. Cell. Mol. Med..

[249] Piera-Velazquez S., Mendoza F.A., Jimenez S.A. (2016). Endothelial to Mesenchymal Transition (EndoMT) in the Pathogenesis of Human Fibrotic Diseases. J. Clin. Med..

[250] López-Antona I., Contreras-Jurado C., Luque-Martín L., Carpintero-Leyva A., González-Méndez P., Palmero I. (2022). Dynamic Regulation of Myofibroblast Phenotype in Cellular Senescence. Aging Cell.

[251] Chang S., Lv J., Wang X., Su J., Bian C., Zheng Z., Yu H., Bao J., Xin Y., Jiang X. (2024). Pathogenic Mechanisms and Latest Therapeutic Approaches for Radiation-Induced Lung Injury: A Narrative Review. Crit. Rev. Oncol. Hematol..

[252] Sternlicht M.D., Wirkner U., Bickelhaupt S., Lopez Perez R., Tietz A., Lipson K.E., Seeley T.W., Huber P.E. (2018). Radiation-Induced Pulmonary Gene Expression Changes Are Attenuated by the CTGF Antibody Pamrevlumab. Respir. Res..

[253] Yanagihara T., Tsubouchi K., Gholiof M., Chong S.G., Lipson K.E., Zhou Q., Scallan C., Upagupta C., Tikkanen J., Keshavjee S. (2022). Connective-Tissue Growth Factor Contributes to TGF-Β1-Induced Lung Fibrosis. Am. J. Respir. Cell Mol. Biol..

[254] Su L., Dong Y., Wang Y., Wang Y., Guan B., Lu Y., Wu J., Wang X., Li D., Meng A. (2021). Potential Role of Senescent Macrophages in Radiation-Induced Pulmonary Fibrosis. Cell Death Dis..

[255] Overstreet J.M., Samarakoon R., Meldrum K.K., Higgins P.J. (2014). Redox Control of P53 in the Transcriptional Regulation of TGF-Β1 Target Genes Through SMAD Cooperativity. Cell. Signal..

[256] Weigel C., Schmezer P., Plass C., Popanda O. (2015). Epigenetics in Radiation-Induced Fibrosis. Oncogene.

[257] Duru N., Wolfson B., Zhou Q. (2016). Mechanisms of the Alternative Activation of Macrophages and Non-Coding RNAs in the Development of Radiation-Induced Lung Fibrosis. World J. Biol. Chem..

[258] Rzeszowska-Wolny J., Hudy D., Biernacki K., Ciesielska S., Jaksik R. (2022). Involvement of miRNAs in Cellular Responses to Radiation. Int. J. Radiat. Biol..

[259] Yamazaki T., Yamashita N., Izumi Y., Nakamura Y., Shiota M., Hanatani A., Shimada K., Muro T., Iwao H., Yoshiyama M. (2012). The Antifibrotic Agent Pirfenidone Inhibits Angiotensin II-Induced Cardiac Hypertrophy in Mice. Hypertens. Res..

[260] Flechsig P., Dadrich M., Bickelhaupt S., Jenne J., Hauser K., Timke C., Peschke P., Hahn E.W., Gröne H.-J., Yingling J. (2012). LY2109761 Attenuates Radiation-Induced Pulmonary Murine Fibrosis via Reversal of TGF-β and BMP-Associated Proinflammatory and Proangiogenic Signals. Clin. Cancer Res..

[261] Mahmood J., Jelveh S., Zaidi A., Doctrow S.R., Medhora M., Hill R.P. (2014). Targeting the Renin-Angiotensin System Combined with an Antioxidant Is Highly Effective in Mitigating Radiation-Induced Lung Damage. Int. J. Radiat. Oncol. Biol. Phys..

[262] Hauer-Jensen M., Denham J.W., Andreyev H.J.N. (2014). Radiation Enteropathy—Pathogenesis, Treatment, and Prevention. Nat. Rev. Gastroenterol. Hepatol..

[263] Husebye E., Hauer-Jensen M., Kjørstad K., Skar V. (1994). Severe Late Radiation Enteropathy Is Characterized by Impaired Motility of Proximal Small Intestine. Dig. Dis. Sci..

[264] Larsen A., Reitan J.B., Aase S.T., Hauer-Jensen M. (2007). Long-Term Prognosis in Patients with Severe Late Radiation Enteropathy: A Prospective Cohort Study. World J. Gastroenterol..

[265] Takemura N., Kurashima Y., Mori Y., Okada K., Ogino T., Osawa H., Matsuno H., Aayam L., Kaneto S., Park E.J. (2018). Eosinophil Depletion Suppresses Radiation-Induced Small Intestinal Fibrosis. Sci. Transl. Med..

[266] Yeh M.-H., Chang Y.-H., Tsai Y.-C., Chen S.-L., Huang T.-S., Chiu J.-F., Ch’ang H.-J. (2016). Bone Marrow Derived Macrophages Fuse with Intestine Stromal Cells and Contribute to Chronic Fibrosis After Radiation. Radiother. Oncol..

[267] Haydont V., Gilliot O., Rivera S., Bourgier C., François A., Aigueperse J., Bourhis J., Vozenin-Brotons M.-C. (2007). Successful Mitigation of Delayed Intestinal Radiation Injury Using Pravastatin Is Not Associated with Acute Injury Improvement or Tumor Protection. Int. J. Radiat. Oncol. Biol. Phys..

[268] Shvayko L.I. (2017). Influence of Emergency Origin Ionizing Radiation on Development of Lung Diseases and Preventing Measures (Chernobyl NPP Accident). Eur. Respir. J..

[269] Sárközy M., Varga Z., Gáspár R., Szűcs G., Kovács M.G., Kovács Z.Z.A., Dux L., Kahán Z., Csont T. (2021). Pathomechanisms and Therapeutic Opportunities in Radiation-Induced Heart Disease: From Bench to Bedside. Clin. Res. Cardiol..

[270] Díaz-Gavela A.A., Figueiras-Graillet L., Luis Á.M., Salas Segura J., Ciérvide R., del Cerro Peñalver E., Couñago F., Arenas M., López-Fernández T. (2021). Breast Radiotherapy-Related Cardiotoxicity. When, How, Why. Risk Prevention and Control Strategies. Cancers.

[271] Tapio S., Little M.P., Kaiser J.C., Impens N., Hamada N., Georgakilas A.G., Simar D., Salomaa S. (2021). Ionizing Radiation-Induced Circulatory and Metabolic Diseases. Environ. Int..

[272] Wijerathne H., Langston J.C., Yang Q., Sun S., Miyamoto C., Kilpatrick L.E., Kiani M.F. (2021). Mechanisms of Radiation-Induced Endothelium Damage: Emerging Models and Technologies. Radiother. Oncol..

[273] Borghini A., Gianicolo E.A.L., Picano E., Andreassi M.G. (2013). Ionizing Radiation and Atherosclerosis: Current Knowledge and Future Challenges. Atherosclerosis.

[274] Baker J.E., Fish B.L., Su J., Haworth S.T., Strande J.L., Komorowski R.A., Migrino R.Q., Doppalapudi A., Harmann L., Allen Li X. (2009). 10 Gy Total Body Irradiation Increases Risk of Coronary Sclerosis, Degeneration of Heart Structure and Function in a Rat Model. Int. J. Radiat. Biol..

[275] Shimizu Y., Kodama K., Nishi N., Kasagi F., Suyama A., Soda M., Grant E.J., Sugiyama H., Sakata R., Moriwaki H. (2010). Radiation Exposure and Circulatory Disease Risk: Hiroshima and Nagasaki Atomic Bomb Survivor Data, 1950-2003. BMJ.

[276] Walls G.M., O’Connor J., Harbinson M., McCarron E.P., Duane F., McCann C., McKavanagh P., Johnston D.I., Erekkath J., Giacometti V. (2023). Association Between Statin Therapy Dose Intensity and Radiation Cardiotoxicity in Non-Small Cell Lung Cancer: Results from the NI-HEART Study. Radiother. Oncol..

[277] Wang B., Wang H., Zhang M., Ji R., Wei J., Xin Y., Jiang X. (2020). Radiation-Induced Myocardial Fibrosis: Mechanisms Underlying Its Pathogenesis and Therapeutic Strategies. J. Cell. Mol. Med..

[278] Moulder J.E., Cohen E.P., Fish B.L. (2011). Captopril and Losartan for Mitigation of Renal Injury Caused by Single-Dose Total-Body Irradiation. Radiat. Res..

[279] Klaus R., Niyazi M., Lange-Sperandio B. (2021). Radiation-Induced Kidney Toxicity: Molecular and Cellular Pathogenesis. Radiat. Oncol..

[280] Rump A., Stricklin D., Lamkowski A., Eder S., Abend M., Port M. (2016). Reconsidering Current Decorporation Strategies After Incorporation of Radionuclides. Health Phys..

[281] Cohen E.P., Fish B.L., Moulder J.E. (2010). Mitigation of Radiation Injuries via Suppression of the Renin-Angiotensin System: Emphasis on Radiation Nephropathy. Curr. Drug Targets.

[282] Moulder J.E., Cohen E.P., Fish B.L. (2014). Mitigation of Experimental Radiation Nephropathy by Renin-Equivalent Doses of Angiotensin Converting Enzyme Inhibitors. Int. J. Radiat. Biol..

[283] Kiang J.G., Cannon G., Singh V.K. (2024). An Overview of Radiation Countermeasure Development in Radiation Research from 1954 to 2024. Radiat. Res..

[284] World Health Organization (2023). National Stockpiles for Radiological and Nuclear Emergencies: Policy Advice.

[285] Crook J., Patil N., Wallace K., Borg J., Zhou D., Ma C., Pond G. (2010). A Phase III Randomized Trial of the Timing of Meloxicam with Iodine-125 Prostate Brachytherapy. Int. J. Radiat. Oncol. Biol. Phys..

[286] Upadhyaya A., Zhou P., Meng Z., Wang P., Zhang G., Jia Q., Tan J., Li X., Hu T., Liu N. (2017). Radioprotective Effect of Vitamin E on Salivary Glands After Radioiodine Therapy for Differentiated Thyroid Cancer: A Randomized-Controlled Trial. Nucl. Med. Commun..

[287] Jafari E., Alavi M., Zal F. (2018). The Evaluation of Protective and Mitigating Effects of Vitamin C Against Side Effects Induced by Radioiodine Therapy. Radiat. Environ. Biophys..

[288] Calcaterra V., Mameli C., Rossi V., Massini G., Gambino M., Baldassarre P., Zuccotti G. (2022). The Iodine Rush: Over- or Under-Iodination Risk in the Prophylactic Use of Iodine for Thyroid Blocking in the Event of a Nuclear Disaster. Front. Endocrinol..

[289] Zaletel K., Mihovec A., Gaberscek S. (2024). Characteristics of Exposure to Radioactive Iodine During a Nuclear Incident. Radiol. Oncol..

[290] Reiners C., Drozd V., Yamashita S. (2020). Hypothyroidism After Radiation Exposure: Brief Narrative Review. J. Neural Transm..

[291] Jing L., Zhang Q. (2022). Intrathyroidal Feedforward and Feedback Network Regulating Thyroid Hormone Synthesis and Secretion. Front. Endocrinol..

[292] World Health Organization (WHO) (2017). Iodine Thyroid Blocking: Guidelines for Use in Planning for and Responding to Radiological and Nuclear Emergencies.

[293] Zanzonico P.B., Becker D.V. (2000). Effects of Time of Administration and Dietary Iodine Levels on Potassium Iodide (KI) Blockade of Thyroid Irradiation by 131I from Radioactive Fallout. Health Phys..

[294] Eder S., Hermann C., Lamkowski A., Kinoshita M., Yamamoto T., Abend M., Shinomiya N., Port M., Rump A. (2020). A Comparison of Thyroidal Protection by Stable Iodine or Perchlorate in the Case of Acute or Prolonged Radioiodine Exposure. Arch. Toxicol..

[295] Rump A., Eder S., Hermann C., Lamkowski A., Kinoshita M., Yamamoto T., Abend M., Shinomiya N., Port M. (2021). A Comparison of Thyroidal Protection by Iodine and Perchlorate Against Radioiodine Exposure in Caucasians and Japanese. Arch. Toxicol..

[296] Ilias I., Rizzo M., Meristoudis G. (2023). Potassium Iodide in Nuclear Accidents: Give It Timely, Swiftly and Judiciously. Endocr. Metab. Immune Disord. Drug Targets.

[297] Martin J.-C., Pourcher T., Phan G., Guglielmi J., Crambes C., Caire-Maurisier F., Lebsir D., Cohen D., Rosique C., Jing L. (2024). Review of the PRIODAC Project on Thyroid Protection from Radioactive Iodine by Repeated Iodine Intake in Individuals Aged 12. Eur. Thyroid J..

[298] Wolff J. (1998). Perchlorate and the Thyroid Gland. Pharmacol. Rev..

[299] Dohán O., Portulano C., Basquin C., Reyna-Neyra A., Amzel L.M., Carrasco N. (2007). The Na+/I− Symporter (NIS) Mediates Electroneutral Active Transport of the Environmental Pollutant Perchlorate. Proc. Natl. Acad. Sci. USA.

[300] Cardis E., Howe G., Ron E., Bebeshko V., Bogdanova T., Bouville A., Carr Z., Chumak V., Davis S., Demidchik Y. (2006). Cancer Consequences of the Chernobyl Accident: 20 Years On. J. Radiol. Prot..

[301] Altagracia-Martínez M., Kravzov-Jinich J., Martínez-Núñez J.M., Ríos-Castañeda C., López-Naranjo F. (2012). Prussian Blue as an Antidote for Radioactive Thallium and Cesium Poisoning. Orphan Drugs Res. Rev..

[302] Mahar R., Sandal N. (2024). Decorporation Dilemma: Interplay of Prussian Blue and Potassium Iodide in Radioactive Contamination. J. Environ. Radioact..

[303] Nielsen C.E., Wang X., Robinson R.J., Brooks A.L., Lovaglio J., Patton K.M., McComish S.L., Tolmachev S.Y., Morgan W.F. (2014). Carcinogenic and Inflammatory Effects of Plutonium-Nitrate Retention in an Exposed Nuclear Worker and Beagle Dogs. Int. J. Radiat. Biol..

[304] Azizova T., Moseeva M., Grigoryeva E., Zhuntova G., Bannikova M., Sychugov G., Kazachkov E. (2020). Registry of Plutonium-Induced Lung Fibrosis in a Russian Nuclear Worker Cohort. Health Phys..

[305] Li C., Kenbayeva Z., Dainiak N., Yepes-Nuñez J.J., Zeeb H., Gill S., Akashi M., Alves A., Chang A., DiCarlo A. (2025). Development of Evidence-Based Guidelines on Assessment and Management of Internal Contamination with Transuranic Actinides. Disaster Med. Public Health Prep..

[306] Griffiths N.M., Van der Meeren A., Grémy O. (2021). Comparison of Local and Systemic DTPA Treatment Efficacy According to Actinide Physicochemical Properties Following Lung or Wound Contamination in the Rat. Front. Pharmacol..

[307] Sandal N., Mahar R., Sharma P. (2025). Optimizing Ca-DTPA/Zn-DTPA Therapy for Internal Decorporation: Transitioning from Intravenous to Oral Route with Insights on Safety and Toxicity. J. Radiol. Prot..

[308] Li Y., Li B., Chen L., Dong J., Xia Z., Tian Y. (2023). Chelating Decorporation Agents for Internal Contamination by Actinides: Designs, Mechanisms, and Advances. J. Inorg. Biochem..

[309] DiCarlo A., Button J., Cassatt D., Chang A., Finklea L., Iyer N., Moroni M., Rios C., Rudokas M., Satyamitra M. (2025). Advanced Medical Countermeasures and Devices for Use During a Radiological or Nuclear Emergency. Disaster Med. Public Health Prep..

[310] Farese A.M., Cohen M.V., Katz B.P., Smith C.P., Gibbs A., Cohen D.M., MacVittie T.J. (2013). Filgrastim Improves Survival in Lethally Irradiated Nonhuman Primates. Radiat. Res..

[311] Hankey K.G., Farese A.M., Blaauw E.C., Gibbs A.M., Smith C.P., Katz B.P., Tong Y., Prado K.L., MacVittie T.J. (2015). Pegfilgrastim Improves Survival of Lethally Irradiated Nonhuman Primates. Rare.

[312] MacVittie T.J., Bennett A.W., Farese A.M., Howell C.-T., Smith C.P., Gibbs A.M., Prado K., Jackson W. (2015). The Effect of Radiation Dose and Variation in Neupogen® Initiation Schedule on the Mitigation of Myelosuppression During the Concomitant GI-ARS and H-ARS in a Nonhuman Primate Model of High-Dose Exposure with Marrow Sparing. Health Phys..

[313] Zhang Y., Chen X., Wang X., Chen J., Du C., Wang J., Liao W. (2024). Insights into Ionizing Radiation-Induced Bone Marrow Hematopoietic Stem Cell Injury. Stem Cell Res. Ther..

[314] Farese A.M., Brown C.R., Smith C.P., Gibbs A.M., Katz B.P., Johnson C.S., Prado K.L., MacVittie T.J. (2014). The Ability of Filgrastim to Mitigate Mortality Following LD50/60 Total-Body Irradiation Is Administration Time-Dependent. Health Phys..

[315] Farese A.M., Bennett A.W., Gibbs A.M., Hankey K.G., Prado K., Jackson W., MacVittie T.J. (2019). Efficacy of Neulasta or Neupogen on H-ARS and GI-ARS Mortality and Hematopoietic Recovery in Nonhuman Primates After 10-Gy Irradiation with 2.5% Bone Marrow Sparing. Health Phys..

[316] Patel D.R., Fonseca X., Patel A.M. (2020). Filgrastim-Associated Pneumonitis in Cancer Patient Undergoing Hematopoietic Stem Cell (HSC) Mobilization for Autologous-HSC Transplantation. Cureus.

[317] Secreto C., Morel B., Bisbal M., Pennors W., Pouliquen C., Albanese J., Leone M., Cerrano M., Servan L., Gonzalez F. (2025). Prognostic Impact of Neutropenia Recovery and G-CSF Use in Onco-Hematological Neutropenic Patients Admitted to Intensive Care Unit for Acute Respiratory Failure: A Retrospective, Real World Analysis. Adv. Ther..

[318] Clayton N.P., Khan-Malek R.C., Dangler C.A., Zhang D., Ascah A., Gains M., Gardner B., Mockbee C., Keutzer J.M., McManus J. (2021). Sargramostim (Rhu GM-CSF) Improves Survival of Non-Human Primates with Severe Bone Marrow Suppression After Acute, High-Dose, Whole-Body Irradiation. Radiat. Res..

[319] Zhong Y., Pouliot M., Downey A.-M., Mockbee C., Roychowdhury D., Wierzbicki W., Authier S. (2021). Efficacy of Delayed Administration of Sargramostim up to 120 Hours Post Exposure in a Nonhuman Primate Total Body Radiation Model. Int. J. Radiat. Biol..

[320] Lazarus H.M., McManus J., Gale R.P. (2022). Sargramostim in Acute Radiation Syndrome. Expert Opin. Biol. Ther..

[321] Gale R.P., Armitage J.O. (2021). Use of Molecularly-Cloned Haematopoietic Growth Factors in Persons Exposed to Acute High-Dose, High-Dose Rate Whole-Body Ionizing Radiations. Blood Rev..

[322] Singh V.K., Seed T.M. (2021). Repurposing Pharmaceuticals Previously Approved by Regulatory Agencies to Medically Counter Injuries Arising Either Early or Late Following Radiation Exposure. Front. Pharmacol..

[323] Braunstein S., Kaplan G., Gottlieb A.B., Schwartz M., Walsh G., Abalos R.M., Fajardo T.T., Guido L.S., Krueger J.G. (1994). GM-CSF Activates Regenerative Epidermal Growth and Stimulates Keratinocyte Proliferation in Human Skin in Vivo. J. Investig. Dermatol..

[324] Saarilahti K., Kajanti M., Joensuu T., Kouri M., Joensuu H. (2002). Comparison of Granulocyte-Macrophage Colony-Stimulating Factor and Sucralfate Mouthwashes in the Prevention of Radiation-Induced Mucositis: A Double-Blind Prospective Randomized Phase III Study. Int. J. Radiat. Oncol. Biol. Phys..

[325] Liang G., Du W., Ke Q., Huang B., Yang J. (2017). The Effects of Recombinant Human Granulocyte Colony-Stimulating Factor Mouthwash on Radiotherapy-Induced Oral Mucositis in Locally Advanced Nasopharyngeal Carcinoma Patients. Adv. Clin. Exp. Med..

[326] Qiao Q. (2025). 1340P Effects of GM-CSF on Prevention and Treatment of Oral Mucositis in Patients Receiving Radiotherapy for Head and Neck Cancer: A Randomized Controlled Trial (ChiCTR2300068457). Ann. Oncol..

[327] McAleese J.J., Bishop K.M., A’Hern R., Henk J.M. (2006). Randomized Phase II Study of GM-CSF to Reduce Mucositis Caused by Accelerated Radiotherapy of Laryngeal Cancer. Br. J. Radiol..

[328] Ryu J.K., Swann S., LeVeque F., Scarantino C.W., Johnson D., Chen A., Fortin A., Pollock J., Kim H., Ang K.K. (2007). The Impact of Concurrent Granulocyte Macrophage-Colony Stimulating Factor on Radiation-Induced Mucositis in Head and Neck Cancer Patients: A Double-Blind Placebo-Controlled Prospective Phase III Study by Radiation Therapy Oncology Group 9901. Int. J. Radiat. Oncol. Biol. Phys..

[329] Hoffman K.E., Pugh S.L., James J.L., Scarantino C., Movsas B., Valicenti R.K., Fortin A., Pollock J., Kim H., Brachman D.G. (2014). The Impact of Concurrent Granulocyte-Macrophage Colony-Stimulating Factor on Quality of Life in Head and Neck Cancer Patients: Results of the Randomized, Placebo-Controlled Radiation Therapy Oncology Group 9901 Trial. Qual. Life Res..

[330] Yamaguchi M., Hirouchi T., Yokoyama K., Nishiyama A., Murakami S., Kashiwakura I. (2018). The Thrombopoietin Mimetic Romiplostim Leads to the Complete Rescue of Mice Exposed to Lethal Ionizing Radiation. Sci. Rep..

[331] Bunin D.I., Bakke J., Green C.E., Javitz H.S., Fielden M., Chang P.Y. (2020). Romiplostim (Nplate®) as an Effective Radiation Countermeasure to Improve Survival and Platelet Recovery in Mice. Int. J. Radiat. Biol..

[332] Yamaguchi M., Hirouchi T., Yoshioka H., Watanabe J., Kashiwakura I. (2019). Diverse Functions of the Thrombopoietin Receptor Agonist Romiplostim Rescue Individuals Exposed to Lethal Radiation. Free Radic. Biol. Med..

[333] Yamaguchi M., Suzuki M., Funaba M., Chiba A., Kashiwakura I. (2020). Mitigative Efficacy of the Clinical Dosage Administration of Granulocyte Colony-Stimulating Factor and Romiplostim in Mice with Severe Acute Radiation Syndrome. Stem Cell Res. Ther..

[334] Hirouchi T., Ito K., Nakano M., Monzen S., Yoshino H., Chiba M., Hazawa M., Nakano A., Ishikawa J., Yamaguchi M. (2015). Mitigative Effects of a Combination of Multiple Pharmaceutical Drugs on the Survival of Mice Exposed to Lethal Ionizing Radiation. Curr. Pharm. Biotechnol..

[335] Aquino-Parsons C., Lomas S., Smith K., Hayes J., Lew S., Bates A.T., Macdonald A.G. (2010). Phase III Study of Silver Leaf Nylon Dressing vs Standard Care for Reduction of Inframammary Moist Desquamation in Patients Undergoing Adjuvant Whole Breast Radiation Therapy. J. Med. Imaging Radiat. Sci..

[336] Niazi T.M., Vuong T., Azoulay L., Marijnen C., Bujko K., Nasr E., Lambert C., Duclos M., Faria S., David M. (2012). Silver Clear Nylon Dressing Is Effective in Preventing Radiation-Induced Dermatitis in Patients with Lower Gastrointestinal Cancer: Results from a Phase III Study. Int. J. Radiat. Oncol. Biol. Phys..

[337] Hemati S., Asnaashari O., Sarvizadeh M., Motlagh B.N., Akbari M., Tajvidi M., Gookizadeh A. (2012). Topical Silver Sulfadiazine for the Prevention of Acute Dermatitis During Irradiation for Breast Cancer. Support. Care Cancer.

[338] Il’in L.A., Samoilov A.S. (2021). The Role of Radiobiology and Nuclear Medicine in Protection from the Effects of Ionizing Radiation (Domestic Experience). Her. Russ. Acad. Sci..

[339] Vinnikov V., Belyakov O. (2022). Clinical Applications of Biological Dosimetry in Patients Exposed to Low Dose Radiation Due to Radiological, Imaging or Nuclear Medicine Procedures. Semin. Nucl. Med..

[340] Grebenyuk A.N., Gladkikh V.D. (2019). Modern Condition and Prospects for the Development of Medicines Towards Prevention and Early Treatment of Radiation Damage. Biol. Bull..

[341] Vasin M.V., Antipov V.V., Chernov G.A., Abramov M.M., Gavriliuk D.N., L’vova T.S., Suvorov N.N. (1997). The role of the vasoconstrictor effect in realizing the radioprotective properties of indralin in experiments on dogs. Radiatsionnaia Biol. Radioecol..

[342] Vasin M.V., Semenov L.F., Suvorov N.N., Antipov V.V., Ushakov I.B., Ilyin L.A., Lapin B.A. (2014). Protective Effect and the Therapeutic Index of Indralin in Juvenile Rhesus Monkeys. J. Radiat. Res..

[343] Vasin M.V., Ushakov I.B. (2015). Comparative Efficacy and the Window of Radioprotection for Adrenergic and Serotoninergic Agents and Aminothiols in Experiments with Small and Large Animals. J. Radiat. Res..

[344] Vasin M.V., Antipov V.V., Komarova S.N., Semenova L.A., Galkin A.A. (2011). Radioprotective properties of indralin combined with cystamine and mexamine. Radiatsionnaia Biol. Radioecol..

[345] Vasin M.V., Ushakov I.B., Kovtun V.Y., Semenova L.A., Komarova S.N., Galkin A.A., Afanas’ev R.V. (2015). Radioprotective Properties of Indralin in Combination with Monizol in the Treatment of Local Acute and Delayed Radiation Injuries Caused by Local Skin γ-Irradiation. Bull. Exp. Biol. Med..

[346] Vasin M.M., Ushakov I.B., Kovtun V.Y. (2016). Radioprotector Indralin at Early and Late Manifestations of Local Radiation Injuries. Vopr. Onkol..

[347] Rozhdestvenskiĭ L.M., Korovkina E.P., Deshevoĭ I.B. (2008). Recombinant human interleukine-1beta (betaleukine) usage for acute radiation sickness of severe degree treatment at canines. Radiatsionnaia Biol. Radioecol..

[348] Grebeniuk A.N., Zatsepin V.V., Aksenova N.V., Nazarov V.B., Vlasenko T.N. (2010). The influence of consecutive application of B-190 preparation and interleukin-1beta on survival rate and bone marrow hematopoiesis of irradiated mice. Radiatsionnaia Biol. Radioecol..

[349] Ilyin L., Ushakov I., Vasin M. Radioprotective Drugs in the System of Radiation Protection of Exposed Radiation Workers and Population in the Case of Nuclear Accidents. Proceedings of the 13th International Congress of the International Radiation Protection Association (IRPA-13).

[350] Zhang Y., Huang Y., Li Z., Wu H., Zou B., Xu Y. (2023). Exploring Natural Products as Radioprotective Agents for Cancer Therapy: Mechanisms, Challenges, and Opportunities. Cancers.

[351] He L., Edi S., Ma J., Kong Z., Dai C., Huang L., Zeng R., Gou K. (2024). Prevention and Treatment of Radiation Injury by Traditional Chinese Medicine: A Review. Chin. Herb. Med..

[352] Yoshida S., Ojino M., Ozaki T., Hatanaka T., Nomura K., Ishii M., Koriyama K., Akashi M. (2014). Guidelines for Iodine Prophylaxis as a Protective Measure: Information for Physicians. Jpn. Med. Assoc. J..

[353] Nagata T., Arishima T., Yamaguchi Y., Hirohashi N., Usa T., Hasegawa A., Hanada H., Yamamoto N., Okamoto T., Akahoshi T. (2022). Radiation Emergency Medical Preparedness in Japan: A Survey of Nuclear Emergency Core Hospitals. Disaster Med. Public Health Prep..

[354] Ishii K., Takashima Y., Akiyama M., Tominaga T., Kawai H., Suto Y. (2025). Harmonization and Strengthening of Japan’s Biodosimetry Network to Support Medical Triage in the Event of a Nuclear Disaster. Int. J. Radiat. Biol..

[355] Yamada Y., Imaoka T., Iwasaki T., Kobayashi J., Misumi M., Sakai K., Sugihara T., Suzuki K., Tauchi H., Yasuda H. (2024). Establishment and Activity of the Planning and Acting Network for Low Dose Radiation Research in Japan (PLANET): 2016–2023. J. Radiat. Res..

[356] Jiao W., Kiang J.G., Cary L., Elliott T.B., Pellmar T.C., Ledney G.D. (2009). COX-2 Inhibitors Are Contraindicated for Treatment of Combined Injury. Radiat. Res..

[357] Liu H., Ma X., Pan D., Cao M., Han Z., Wang H. (2023). Efficacy and Safety of Hetrombopag for Thrombocytopenia in Patients with Advanced Solid Tumors: A Retrospective Study. J. Clin. Pharm. Ther..

[358] Li J.-D., Han X.-L., Wu S. (2009). Effect of recombinant human interleukin 11 on recovery of platelets After peripheral blood hematopoietic stem cell transplantation. Zhongguo Shi Yan Xue Ye Xue Za Zhi.

[359] Vercellino J., Małachowska B., Kulkarni S., Bell B.I., Shajahan S., Shinoda K., Eichenbaum G., Verma A.K., Ghosh S.P., Yang W.-L. (2024). Thrombopoietin Mimetic Stimulates Bone Marrow Vascular and Stromal Niches to Mitigate Acute Radiation Syndrome. Stem Cell Res. Ther..

[360] Duic J.P., Grewal J., McConie K., Staszewski H., Haas J., Kesari S. (2012). Eltrombopag for Radiation-Induced Thrombocytopenia in a Glioblastoma Patient. J. Neuro-Oncol..

[361] Liesveld J.L., Phillips G.L., Becker M., Constine L.S., Friedberg J., Andolina J.R., Milner L.A., DeBolt J., Smudzin T., Hyrien O. (2013). A Phase 1 Trial of Eltrombopag in Patients Undergoing Stem Cell Transplantation After Total Body Irradiation. Biol. Blood Marrow Transplant..

[362] Sakthivel G., Nguyen K., Yang H., Liesveld J., Chen Y. (2022). Impact of Eltrombopag, a Thrombopoietin Mimetic on Hematopoietic Recovery After Total Body Irradiation for Stem Cell Transplantation. Int. J. Radiat. Oncol. Biol. Phys..

[363] Ahmed S., Bashir Q., Bassett R.L., Ullah F., Aung F., Valdez B., Alousi A.M., Hosing C., Kebriaei P., Khouri I. (2024). Eltrombopag Improves Platelet Engraftment After Haploidentical Bone Marrow Transplantation: Results of a Phase II Study. Am. J. Hematol..

[364] Chi Y., Hu Q., Yang C., Chen M., Han B. (2023). Avatrombopag Is Effective in Patients with Chemoradiotherapy-Induced Aplastic Anemia: A Single-Center, Retrospective Study. Exp. Hematol..

[365] Feng Y., Wang L., Ren M., Wang D., Lang T., Deng J., Lou S., Yi H., Ma L., Xing H. (2025). Efficacy and Safety of Hetrombopag Versus Thrombopoietin in Promoting Platelet Engraftment After Allogeneic Hematopoietic Stem Cell Transplantation: A Prospective, Multicenter, Randomized Controlled Clinical Trial. Am. J. Hematol..

[366] Ni J., Hong J., Liang X., Dai J., Long Z., Luan C., Yang M., Li Q. (2024). Efficacy and Safety of Hetrombopag in the Treatment of Recombinant Human Thrombopoietin–Resistant Thrombocytopenia After Allogeneic Hematopoietic Stem Cell Transplantation. Res. Pract. Thromb. Haemost..

[367] Zhou W., Jin Y., Xie X., Shi L., Sun X. (2024). 1862P A Single-Arm, Phase II Trial of Hetrombopag for the Treatment of Concurrent Chemoradiotherapy-Induced Thrombocytopenia in Patients with Advanced Solid Tumors. Ann. Oncol..

[368] Li G., Huang A., Yang Q., Chen Y., Jiang Y., Liu Z. (2023). Effectiveness and Safety of Hetrombopag in the Management of Radiotherapy-Induced Thrombocytopenia in Patients with Gynecological Malignancies. Blood.

[369] Samtani M.N., Perez-Ruixo J.J., Brown K.H., Cerneus D., Molloy C.J. (2009). Pharmacokinetic and Pharmacodynamic Modeling of Pegylated Thrombopoietin Mimetic Peptide (PEG-TPOm) After Single Intravenous Dose Administration in Healthy Subjects. J. Clin. Pharmacol..

[370] Kumar V.P., Holmes-Hampton G.P., Biswas S., Stone S., Sharma N.K., Hritzo B., Guilfoyle M., Eichenbaum G., Guha C., Ghosh S.P. (2022). Mitigation of Total Body Irradiation-Induced Mortality and Hematopoietic Injury of Mice by a Thrombopoietin Mimetic (JNJ-26366821). Sci. Rep..

[371] Holmes-Hampton G.P., Kumar V.P., Biswas S., Stone S., Sharma N.K., Legesse B., Vercellino J., Guha C., Eichenbaum G., Ghosh S.P. (2023). PEGylated Thrombopoietin Mimetic, JNJ-26366821 a Novel Prophylactic Radiation Countermeasure for Acute Radiation Injury. Sci. Rep..

[372] English J., Dhanikonda S., Tanaka K.E., Koba W., Eichenbaum G., Yang W.-L., Guha C. (2024). Thrombopoietin Mimetic Reduces Mouse Lung Inflammation and Fibrosis After Radiation by Attenuating Activated Endothelial Phenotypes. JCI Insight.

[373] Adrianzen-Herrera D., Choudhary G., Gordon-Mitchell S., Ramachandra N., Bhagat T., Zhang H., Aluri S., Shastri A., Steidl U., Will B. (2020). The Thrombopoietin Mimetic JNJ-26366821 Increases Megakaryopoiesis Without Affecting Malignant Myeloid Proliferation. Leuk. Lymphoma.

[374] Zeidler C., Kanz L., Hurkuck F., Rittmann K.L., Wildfang I., Kadoya T., Mikayama T., Souza L., Welte K. (1992). In Vivo Effects of Interleukin-6 on Thrombopoiesis in Healthy and Irradiated Primates. Blood.

[375] Hao J., Sun L., Huang H., Xiong G., Liu X., Qiu L., Chen G., Dong B., Li Y., Chen W. (2004). Effects of Recombinant Human Interleukin 11 on Thrombocytopenia and Neutropenia in Irradiated Rhesus Monkeys. Radiat. Res..

[376] Gluzman-Poltorak Z., Vainstein V., Basile L.A. (2014). Recombinant Interleukin-12, but Not Granulocyte-Colony Stimulating Factor, Improves Survival in Lethally Irradiated Nonhuman Primates in the Absence of Supportive Care: Evidence for the Development of a Frontline Radiation Medical Countermeasure. Am. J. Hematol..

[377] Chen B.J., Deoliveira D., Spasojevic I., Sempowski G.D., Jiang C., Owzar K., Wang X., Gesty-Palmer D., Cline J.M., Bourland J.D. (2010). Growth Hormone Mitigates Against Lethal Irradiation and Enhances Hematologic and Immune Recovery in Mice and Nonhuman Primates. PLoS ONE.

[378] Suzuki K. (2025). Thrombomodulin: A Key Regulator of Intravascular Blood Coagulation, Fibrinolysis, and Inflammation, and a Treatment for Disseminated Intravascular Coagulation. Proc. Jpn. Acad. Ser. B Phys. Biol. Sci..

[379] Richter K.K., Fink L.M., Hughes B.M., Sung C.C., Hauer-Jensen M. (1997). Is the Loss of Endothelial Thrombomodulin Involved in the Mechanism of Chronicity in Late Radiation Enteropathy?. Radiother. Oncol..

[380] El-Benhawy S.A., Sadek N.A., Kamel M.M., Sharaf A.M., Abderhman I.G., Morsi M.I., Abobakr A. (2020). Study the Relationship of Endothelial Damage/Dysfunction Due to Occupational Exposure to Low Dose Ionizing Radiation versus High Dose Exposure During Radiotherapy. Cancer Treat. Res. Commun..

[381] Wang J., Zheng H., Ou X., Fink L.M., Hauer-Jensen M. (2002). Deficiency of Microvascular Thrombomodulin and Up-Regulation of Protease-Activated Receptor-1 in Irradiated Rat Intestine: Possible Link Between Endothelial Dysfunction and Chronic Radiation Fibrosis. Am. J. Pathol..

[382] Wang J., Boerma M., Fu Q., Hauer-Jensen M. (2007). Significance of Endothelial Dysfunction in the Pathogenesis of Early and Delayed Radiation Enteropathy. World J. Gastroenterol..

[383] Milliat F., Sabourin J.-C., Tarlet G., Holler V., Deutsch E., Buard V., Tamarat R., Atfi A., Benderitter M., François A. (2008). Essential Role of Plasminogen Activator Inhibitor Type-1 in Radiation Enteropathy. Am. J. Pathol..

[384] Pathak R., Wang J., Garg S., Aykin-Burns N., Petersen K.-U., Hauer-Jensen M. (2016). Recombinant Thrombomodulin (Solulin) Ameliorates Early Intestinal Radiation Toxicity in a Preclinical Rat Model. Radiat. Res..

[385] Pathak R., Shao L., Ghosh S.P., Zhou D., Boerma M., Weiler H., Hauer-Jensen M. (2015). Thrombomodulin Contributes to Gamma Tocotrienol-Mediated Lethality Protection and Hematopoietic Cell Recovery in Irradiated Mice. PLoS ONE.

[386] Shrum S.A., Nukala U., Shrimali S., Pineda E.N., Krager K.J., Thakkar S., Jones D.E., Pathak R., Breen P.J., Aykin-Burns N. (2023). Tocotrienols Provide Radioprotection to Multiple Organ Systems Through Complementary Mechanisms of Antioxidant and Signaling Effects. Antioxidants.

[387] Pathak R., Kumar V.P., Hauer-Jensen M., Ghosh S.P. (2019). Enhanced Survival in Mice Exposed to Ionizing Radiation by Combination of Gamma-Tocotrienol and Simvastatin. Mil. Med..

[388] Jang H., Kwak S.-Y., Park S., Kim K., Kim Y., Na J., Kim H., Jang W.-S., Lee S.-J., Kim M.J. (2020). Pravastatin Alleviates Radiation Proctitis by Regulating Thrombomodulin in Irradiated Endothelial Cells. Int. J. Mol. Sci..

[389] Kwak S.Y., Park S., Kim H., Lee S.-J., Jang W.-S., Kim M.-J., Lee S., Jang W.I., Kim A.R., Kim E.H. (2021). Atorvastatin Inhibits Endothelial PAI-1-Mediated Monocyte Migration and Alleviates Radiation-Induced Enteropathy. Int. J. Mol. Sci..

[390] Farrell C.L., Bready J.V., Rex K.L., Chen J.N., DiPalma C.R., Whitcomb K.L., Yin S., Hill D.C., Wiemann B., Starnes C.O. (1998). Keratinocyte Growth Factor Protects Mice from Chemotherapy and Radiation-Induced Gastrointestinal Injury and Mortality. Cancer Res..

[391] Zhang L., Sun W., Wang J., Zhang M., Yang S., Tian Y., Vidyasagar S., Peña L.A., Zhang K., Cao Y. (2010). Mitigation Effect of an FGF-2 Peptide on Acute Gastrointestinal Syndrome After High-Dose Ionizing Radiation. Int. J. Radiat. Oncol. Biol. Phys..

[392] Zhang K., Tian Y., Yin L., Zhang M., Beck L.A., Zhang B., Okunieff P., Zhang L., Vidyasagar S. (2011). Fibroblast Growth Factor-Peptide Improves Barrier Function and Proliferation in Human Keratinocytes After Radiation. Int. J. Radiat. Oncol. Biol. Phys..

[393] Farrell C.L., Rex K.L., Kaufman S.A., Dipalma C.R., Chen J.N., Scully S., Lacey D.L. (1999). Effects of Keratinocyte Growth Factor in the Squamous Epithelium of the Upper Aerodigestive Tract of Normal and Irradiated Mice. Int. J. Radiat. Biol..

[394] Chen L., Brizel D.M., Rabbani Z.N., Samulski T.V., Farrell C.L., Larrier N., Anscher M.S., Vujaskovic Z. (2004). The Protective Effect of Recombinant Human Keratinocyte Growth Factor on Radiation-Induced Pulmonary Toxicity in Rats. Int. J. Radiat. Oncol. Biol. Phys..

[395] Beaven A.W., Shea T.C. (2007). The Effect of Palifermin on Chemotherapyand Radiation Therapy-Induced Mucositis: A Review of the Current Literature. Support. Cancer Ther..

[396] Choi J.-S., Shin H.-S., An H.-Y., Kim Y.-M., Lim J.-Y. (2017). Radioprotective Effects of Keratinocyte Growth Factor-1 Against Irradiation-Induced Salivary Gland Hypofunction. Oncotarget.

[397] Min D., Taylor P.A., Panoskaltsis-Mortari A., Chung B., Danilenko D.M., Farrell C., Lacey D.L., Blazar B.R., Weinberg K.I. (2002). Protection from Thymic Epithelial Cell Injury by Keratinocyte Growth Factor: A New Approach to Improve Thymic and Peripheral T-Cell Reconstitution After Bone Marrow Transplantation. Blood.

[398] Seggewiss R., Loré K., Guenaga F.J., Pittaluga S., Mattapallil J., Chow C.K., Koup R.A., Camphausen K., Nason M.C., Meier-Schellersheim M. (2007). Keratinocyte Growth Factor Augments Immune Reconstitution After Autologous Hematopoietic Progenitor Cell Transplantation in Rhesus Macaques. Blood.

[399] Spielberger R., Stiff P., Bensinger W., Gentile T., Weisdorf D., Kewalramani T., Shea T., Yanovich S., Hansen K., Noga S. (2004). Palifermin for Oral Mucositis After Intensive Therapy for Hematologic Cancers. N. Engl. J. Med..

[400] Stiff P.J., Leinonen M., Kullenberg T., Rudebeck M., de Chateau M., Spielberger R. (2016). Long-Term Safety Outcomes in Patients with Hematological Malignancies Undergoing Autologous Hematopoietic Stem Cell Transplantation Treated with Palifermin to Prevent Oral Mucositis. Biol. Blood Marrow Transplant..

[401] Goldberg J.D., Zheng J., Castro-Malaspina H., Jakubowski A.A., Heller G., van den Brink M.R.M., Perales M.-A. (2013). Palifermin Is Efficacious in Recipients of TBI-Based but Not Chemotherapy-Based Allogeneic Hematopoietic Stem Cell Transplants. Bone Marrow Transplant..

[402] Lucchese A., Matarese G., Ghislanzoni L.H., Gastaldi G., Manuelli M., Gherlone E. (2016). Efficacy and Effects of Palifermin for the Treatment of Oral Mucositis in Patients Affected by Acute Lymphoblastic Leukemia. Leuk. Lymphoma.

[403] Lucchese A., Matarese G., Manuelli M., Ciuffreda C., Bassani L., Isola G., Cordasco G., Gherlone E. (2016). Reliability and Efficacy of Palifermin in Prevention and Management of Oral Mucositis in Patients with Acute Lymphoblastic Leukemia: A Randomized, Double-Blind Controlled Clinical Trial. Minerva Stomatol..

[404] Schmidt V., Niederwieser D., Schenk T., Behre G., Klink A., Pfrepper C., Hinke A., Beelen D.W., Junghanss C., Uharek L. (2018). Efficacy and Safety of Keratinocyte Growth Factor (Palifermin) for Prevention of Oral Mucositis in TBI-Based Allogeneic Hematopoietic Stem Cell Transplantation. Bone Marrow Transplant..

[405] Nguyen D.T., Shayani S., Palmer J., Dagis A., Forman S.J., Epstein J., Spielberger R. (2015). Palifermin for Prevention of Oral Mucositis in Allogeneic Hematopoietic Stem Cell Transplantation: A Single-Institution Retrospective Evaluation. Support. Care Cancer.

[406] Elad S., Cheng K.K.F., Lalla R.V., Yarom N., Hong C., Logan R.M., Bowen J., Gibson R., Saunders D.P., Zadik Y. (2020). MASCC/ISOO Clinical Practice Guidelines for the Management of Mucositis Secondary to Cancer Therapy. Cancer.

[407] Brizel D.M., Murphy B.A., Rosenthal D.I., Pandya K.J., Glück S., Brizel H.E., Meredith R.F., Berger D., Chen M.-G., Mendenhall W. (2008). Phase II Study of Palifermin and Concurrent Chemoradiation in Head and Neck Squamous Cell Carcinoma. J. Clin. Oncol..

[408] Le Q.-T., Kim H.E., Schneider C.J., Muraközy G., Skladowski K., Reinisch S., Chen Y., Hickey M., Mo M., Chen M.-G. (2011). Palifermin Reduces Severe Mucositis in Definitive Chemoradiotherapy of Locally Advanced Head and Neck Cancer: A Randomized, Placebo-Controlled Study. J. Clin. Oncol..

[409] Henke M., Alfonsi M., Foa P., Giralt J., Bardet E., Cerezo L., Salzwimmer M., Lizambri R., Emmerson L., Chen M.-G. (2011). Palifermin Decreases Severe Oral Mucositis of Patients Undergoing Postoperative Radiochemotherapy for Head and Neck Cancer: A Randomized, Placebo-Controlled Trial. J. Clin. Oncol..

[410] Hamzi H., Binhassan A., Najmeldin A., Alhariri E., Elhussein B., Althibani N., Alenazi A., Alsharif S., Alshahrani M., Alsharif O. (2024). Efficacy of Palifermin in the Treatment of Oral Mucositis in Non-Hematopoietic Stem Cell Transplant Pediatric Patients: Experience of a Single Tertiary Hospital. J. Egypt. Natl. Cancer Inst..

[411] Coutsouvelis J., Corallo C., Spencer A., Avery S., Dooley M., Kirkpatrick C.M. (2022). A Meta-Analysis of Palifermin Efficacy for the Management of Oral Mucositis in Patients with Solid Tumours and Haematological Malignancy. Crit. Rev. Oncol. Hematol..

[412] Schwertschlag U.S., Trepicchio W.L., Dykstra K.H., Keith J.C., Turner K.J., Dorner A.J. (1999). Hematopoietic, Immunomodulatory and Epithelial Effects of Interleukin-11. Leukemia.

[413] Singh V.K., Seed T.M. (2023). The Safety and Efficacy of Interleukin 11 for Radiation Injury. Expert Opin. Drug Saf..

[414] Sonis S.T., Peterson R.L., Edwards L.J., Lucey C.A., Wang L., Mason L., Login G., Ymamkawa M., Moses G., Bouchard P. (2000). Defining Mechanisms of Action of Interleukin-11 on the Progression of Radiation-Induced Oral Mucositis in Hamsters. Oral Oncol..

[415] Du X.X., Doerschuk C.M., Orazi A., Williams D.A. (1994). A Bone Marrow Stromal-Derived Growth Factor, Interleukin-11, Stimulates Recovery of Small Intestinal Mucosal Cells After Cytoablative Therapy. Blood.

[416] Burnett A.F., Biju P.G., Lui H., Hauer-Jensen M. (2013). Oral Interleukin 11 as a Countermeasure to Lethal Total-Body Irradiation in a Murine Model. Radiat. Res..

[417] Pan Y., Wu H., Li Y. (2017). Effect of recombinant human interleukin-11 treatment on prognosis of patients with radiochemoradiotherapy in the treatment of nasopharyngeal carcinoma. Lin Chuang Er Bi Yan Hou Tou Jing Wai Ke Za Zhi.

[418] Cook S.A. (2023). Understanding Interleukin 11 as a Disease Gene and Therapeutic Target. Biochem. J..

[419] Kumar V.P., Biswas S., Sharma N.K., Stone S., Fam C.M., Cox G.N., Ghosh S.P. (2018). PEGylated IL-11 (BBT-059): A Novel Radiation Countermeasure for Hematopoietic Acute Radiation Syndrome. Health Phys..

[420] Taliaferro L.P., Cassatt D.R., Horta Z.P., Satyamitra M.M. (2021). Meeting Report: A Poly-Pharmacy Approach to Mitigate Acute Radiation Syndrome. Radiat. Res..

[421] Chen T., Burke K.A., Zhan Y., Wang X., Shibata D., Zhao Y. (2007). IL-12 Facilitates Both the Recovery of Endogenous Hematopoiesis and the Engraftment of Stem Cells After Ionizing Radiation. Exp. Hematol..

[422] Li P., Zhang H., Ji L., Wang Z. (2020). A Review of Clinical and Preclinical Studies on Therapeutic Strategies Using Interleukin-12 in Cancer Therapy and the Protective Role of Interleukin-12 in Hematological Recovery in Chemoradiotherapy. Med. Sci. Monit..

[423] Gerber S.A., Cummings R.J., Judge J.L., Barlow M.L., Nanduri J., Milano Johnson D.E., Palis J., Pentland A.P., Lord E.M., Ryan J.L. (2015). Interleukin-12 Preserves the Cutaneous Physical and Immunological Barrier After Radiation Exposure. Radiat. Res..

[424] Basile L.A., Ellefson D., Gluzman-Poltorak Z., Junes-Gill K., Mar V., Mendonca S., Miller J.D., Tom J., Trinh A., Gallaher T.K. (2012). HemaMax^TM^, a Recombinant Human Interleukin-12, Is a Potent Mitigator of Acute Radiation Injury in Mice and Non-Human Primates. PLoS ONE.

[425] Gluzman-Poltorak Z., Mendonca S.R., Vainstein V., Kha H., Basile L.A. (2014). Randomized Comparison of Single Dose of Recombinant Human IL-12 versus Placebo for Restoration of Hematopoiesis and Improved Survival in Rhesus Monkeys Exposed to Lethal Radiation. J. Hematol. Oncol..

[426] Gokhale M.S., Vainstein V., Tom J., Thomas S., Lawrence C.E., Gluzman-Poltorak Z., Siebers N., Basile L.A. (2014). Single Low-Dose rHuIL-12 Safely Triggers Multilineage Hematopoietic and Immune-Mediated Effects. Exp. Hematol. Oncol..

[427] Qian L., Cen J. (2020). Hematopoietic Stem Cells and Mesenchymal Stromal Cells in Acute Radiation Syndrome. Oxidative Med. Cell. Longev..

[428] Wang K.-X., Cui W.-W., Yang X., Tao A.-B., Lan T., Li T.-S., Luo L. (2021). Mesenchymal Stem Cells for Mitigating Radiotherapy Side Effects. Cells.

[429] François S., Bensidhoum M., Mouiseddine M., Mazurier C., Allenet B., Semont A., Frick J., Saché A., Bouchet S., Thierry D. (2006). Local Irradiation Not Only Induces Homing of Human Mesenchymal Stem Cells at Exposed Sites but Promotes Their Widespread Engraftment to Multiple Organs: A Study of Their Quantitative Distribution After Irradiation Damage. Stem Cells.

[430] Christy B.A., Herzig M.C., Wu X., Mohammadipoor A., McDaniel J.S., Bynum J.A. (2024). Cell Therapies for Acute Radiation Syndrome. Int. J. Mol. Sci..

[431] Hu K.X., Sun Q.Y., Guo M., Ai H.S. (2010). The Radiation Protection and Therapy Effects of Mesenchymal Stem Cells in Mice with Acute Radiation Injury. Br. J. Radiol..

[432] Lange C., Brunswig-Spickenheier B., Cappallo-Obermann H., Eggert K., Gehling U.M., Rudolph C., Schlegelberger B., Cornils K., Zustin J., Spiess A.-N. (2011). Radiation Rescue: Mesenchymal Stromal Cells Protect from Lethal Irradiation. PLoS ONE.

[433] François M., Birman E., Forner K.-A., Gaboury L., Galipeau J. (2012). Adoptive Transfer of Mesenchymal Stromal Cells Accelerates Intestinal Epithelium Recovery of Irradiated Mice in an Interleukin-6-Dependent Manner. Cytotherapy.

[434] Shim S., Lee S.B., Lee J., Jang W.-S., Lee S.-J., Park S., Lee S.-S. (2013). Mitigating Effects of hUCB-MSCs on the Hematopoietic Syndrome Resulting from Total Body Irradiation. Exp. Hematol..

[435] Kim M.-J., Moon W., Heo J., Lim S., Lee S.-H., Jeong J.-Y., Lee S.J. (2022). Optimization of Adipose Tissue-Derived Mesenchymal Stromal Cells Transplantation for Bone Marrow Repopulation Following Irradiation. World J. Stem Cells.

[436] Pinzur L., Akyuez L., Levdansky L., Blumenfeld M., Volinsky E., Aberman Z., Reinke P., Ofir R., Volk H.-D., Gorodetsky R. (2018). Rescue from Lethal Acute Radiation Syndrome (ARS) with Severe Weight Loss by Secretome of Intramuscularly Injected Human Placental Stromal Cells. J. Cachexia Sarcopenia Muscle.

[437] Bernardo M.E., Cometa A.M., Locatelli F. (2012). Mesenchymal Stromal Cells: A Novel and Effective Strategy for Facilitating Engraftment and Accelerating Hematopoietic Recovery After Transplantation?. Bone Marrow Transplant..

[438] Chen X., Wang C., Yin J., Xu J., Wei J., Zhang Y. (2015). Efficacy of Mesenchymal Stem Cell Therapy for Steroid-Refractory Acute Graft-Versus-Host Disease Following Allogeneic Hematopoietic Stem Cell Transplantation: A Systematic Review and Meta-Analysis. PLoS ONE.

[439] Jiang E., Qian K., Wang L., Yang D., Shao Y., Hu L., Li Y., Yao C., Han M., Hou X. (2024). Efficacy and Safety of Human Umbilical Cord-Derived Mesenchymal Stem Cells versus Placebo Added to Second-Line Therapy in Patients with Steroid-Refractory Acute Graft-versus-Host Disease: A Multicentre, Randomized, Double-Blind, Phase 2 Trial. BMC Med..

[440] Saha S., Bhanja P., Kabarriti R., Liu L., Alfieri A.A., Guha C. (2011). Bone Marrow Stromal Cell Transplantation Mitigates Radiation-Induced Gastrointestinal Syndrome in Mice. PLoS ONE.

[441] Sémont A., Demarquay C., Bessout R., Durand C., Benderitter M., Mathieu N. (2013). Mesenchymal Stem Cell Therapy Stimulates Endogenous Host Progenitor Cells to Improve Colonic Epithelial Regeneration. PLoS ONE.

[442] Gong W., Guo M., Han Z., Wang Y., Yang P., Xu C., Wang Q., Du L., Li Q., Zhao H. (2016). Mesenchymal Stem Cells Stimulate Intestinal Stem Cells to Repair Radiation-Induced Intestinal Injury. Cell Death Dis..

[443] Accarie A., l’Homme B., Benadjaoud M.A., Lim S.K., Guha C., Benderitter M., Tamarat R., Sémont A. (2020). Extracellular Vesicles Derived from Mesenchymal Stromal Cells Mitigate Intestinal Toxicity in a Mouse Model of Acute Radiation Syndrome. Stem Cell Res. Ther..

[444] Bandekar M., Maurya D.K., Sharma D., Checker R., Gota V., Mishra N., Sandur S.K. (2020). Xenogeneic Transplantation of Human WJ-MSCs Rescues Mice from Acute Radiation Syndrome via Nrf-2-Dependent Regeneration of Damaged Tissues. Am. J. Transplant..

[445] Maria O.M., Shalaby M., Syme A., Eliopoulos N., Muanza T. (2016). Adipose Mesenchymal Stromal Cells Minimize and Repair Radiation-Induced Oral Mucositis. Cytotherapy.

[446] Gundestrup A.K., Lynggaard C.D., Forner L., Heino T.J., Jakobsen K.K., Fischer-Nielsen A., Grønhøj C., von Buchwald C. (2020). Mesenchymal Stem Cell Therapy for Osteoradionecrosis of the Mandible: A Systematic Review of Preclinical and Human Studies. Stem Cell Rev. Rep..

[447] Chen Y., Liu X., Tong Z. (2022). Mesenchymal Stem Cells in Radiation-Induced Pulmonary Fibrosis: Future Prospects. Cells.

[448] Wang R., Zhu C., Qiao P., Liu J., Zhao Q., Wang K., Zhao T. (2014). Experimental Treatment of Radiation Pneumonitis with Human Umbilical Cord Mesenchymal Stem Cells. Asian Pac. J. Trop. Med..

[449] Jiang X., Jiang X., Qu C., Chang P., Zhang C., Qu Y., Liu Y. (2015). Intravenous Delivery of Adipose-Derived Mesenchymal Stromal Cells Attenuates Acute Radiation-Induced Lung Injury in Rats. Cytotherapy.

[450] Zanoni M., Cortesi M., Zamagni A., Tesei A. (2019). The Role of Mesenchymal Stem Cells in Radiation-Induced Lung Fibrosis. Int. J. Mol. Sci..

[451] Wang G.-H., Liu Y., Wu X.-B., Lu Y., Liu J., Qin Y.-R., Li T., Duan H.-F. (2016). Neuroprotective Effects of Human Umbilical Cord-Derived Mesenchymal Stromal Cells Combined with Nimodipine Against Radiation-Induced Brain Injury Through Inhibition of Apoptosis. Cytotherapy.

[452] Soria B., Martin-Montalvo A., Aguilera Y., Mellado-Damas N., López-Beas J., Herrera-Herrera I., López E., Barcia J.A., Alvarez-Dolado M., Hmadcha A. (2019). Human Mesenchymal Stem Cells Prevent Neurological Complications of Radiotherapy. Front. Cell. Neurosci..

[453] Wang G., Ren X., Yan H., Gui Y., Guo Z., Song J., Zhang P. (2020). Neuroprotective Effects of Umbilical Cord-Derived Mesenchymal Stem Cells on Radiation-Induced Brain Injury in Mice. Ann. Clin. Lab. Sci..

[454] Mouiseddine M., François S., Souidi M., Chapel A. (2012). Intravenous Human Mesenchymal Stem Cells Transplantation in NOD/SCID Mice Preserve Liver Integrity of Irradiation Damage. Methods Mol. Biol..

[455] Francois S., Mouiseddine M., Allenet-Lepage B., Voswinkel J., Douay L., Benderitter M., Chapel A. (2013). Human Mesenchymal Stem Cells Provide Protection Against Radiation-Induced Liver Injury by Antioxidative Process, Vasculature Protection, Hepatocyte Differentiation, and Trophic Effects. BioMed Res. Int..

[456] Horton J.A., Hudak K.E., Chung E.J., White A.O., Scroggins B.T., Burkeen J.F., Citrin D.E. (2013). Mesenchymal Stem Cells Inhibit Cutaneous Radiation-Induced Fibrosis by Suppressing Chronic Inflammation. Stem Cells.

[457] Zheng K., Wu W., Yang S., Huang L., Chen J., Gong C., Fu Z., Zhang L., Tan J. (2015). Bone Marrow Mesenchymal Stem Cell Implantation for the Treatment of Radioactivity-induced Acute Skin Damage in Rats. Mol. Med. Rep..

[458] Sproull M., Jackson L.R., Citrin D.E., Camphausen K. (2025). Mesenchymal Stem Cell Treatment of Cutaneous Radiation Injury. Int. J. Radiat. Oncol. Biol. Phys..

[459] Uchiki T., Hamada N., Fujita A., Kawano K., Hirota S., Maeda M., Sasaki A., Nagamatsu S., Yoshinaga S., Nakashima A. (2025). Mesenchymal Stem Cell Implantation Mitigates Ionizing Radiation-Induced Vascular Damage in the Murine Aorta. Sci. Rep..

[460] Kotenko K., Moroz B., Nadezhina N., Galstyan I., Eremin I., Deshevoy J., Lebedev V., Slobodina T., Grinakovskaya D., Zhgutov Y. (2012). Successful Treatment of Localised Radiation Lesions in Rats and Humans by Mesenchymal Stem Cell Transplantation. Radiat. Prot. Dosim..

[461] Foubert P., Doyle-Eisele M., Gonzalez A., Berard F., Weber W., Zafra D., Alfonso Z., Zhao S., Tenenhaus M., Fraser J.K. (2017). Development of a Combined Radiation and Full Thickness Burn Injury Minipig Model to Study the Effects of Uncultured Adipose-Derived Regenerative Cell Therapy in Wound Healing. Int. J. Radiat. Biol..

[462] Klein D., Steens J., Wiesemann A., Schulz F., Kaschani F., Röck K., Yamaguchi M., Wirsdörfer F., Kaiser M., Fischer J.W. (2017). Mesenchymal Stem Cell Therapy Protects Lungs from Radiation-Induced Endothelial Cell Loss by Restoring Superoxide Dismutase 1 Expression. Antioxid. Redox Signal..

[463] Ejaz A., Epperly M.W., Hou W., Greenberger J.S., Rubin J.P. (2019). Adipose-Derived Stem Cell Therapy Ameliorates Ionizing Irradiation Fibrosis via Hepatocyte Growth Factor-Mediated Transforming Growth Factor-β Downregulation and Recruitment of Bone Marrow Cells. Stem Cells.

[464] Miura Y., Fujii S., Ichinohe T. (2024). Cell-Based and Extracellular Vesicle-Based MSC Therapies for Acute Radiation Syndrome Affecting Organ Systems. J. Radiat. Res..

[465] Huang C., Li H., Zhang Z., Mou T., Wang D., Li C., Tian L., Zong C. (2025). From Mechanism to Therapy: The Role of MSC-EVs in Alleviating Radiation-Induced Injuries. Pharmaceutics.

[466] Wen S., Dooner M., Cheng Y., Papa E., Del Tatto M., Pereira M., Deng Y., Goldberg L., Aliotta J., Chatterjee D. (2016). Mesenchymal Stromal Cell-Derived Extracellular Vesicles Rescue Radiation Damage to Murine Marrow Hematopoietic Cells. Leukemia.

[467] Wen S., Dooner M., Pereira M., Del Tatto M., Quesenberry P. (2025). Mesenchymal Stem Cell-Derived Extracellular Vesicles Improve Survival and Enhance Hematopoietic Recovery in Mice Exposed to High-Dose Irradiation. Stem Cells Dev..

[468] Zuo R., Liu M., Wang Y., Li J., Wang W., Wu J., Sun C., Li B., Wang Z., Lan W. (2019). BM-MSC-Derived Exosomes Alleviate Radiation-Induced Bone Loss by Restoring the Function of Recipient BM-MSCs and Activating Wnt/β-Catenin Signaling. Stem Cell Res. Ther..

[469] He N., Dong M., Sun Y., Yang M., Wang Y., Du L., Ji K., Wang J., Zhang M., Gu Y. (2024). Mesenchymal Stem Cell-Derived Extracellular Vesicles Targeting Irradiated Intestine Exert Therapeutic Effects. Theranostics.

[470] Lei X., He N., Zhu L., Zhou M., Zhang K., Wang C., Huang H., Chen S., Li Y., Liu Q. (2021). Mesenchymal Stem Cell-Derived Extracellular Vesicles Attenuate Radiation-Induced Lung Injury via miRNA-214-3p. Antioxid. Redox Signal..

[471] Lynggaard C.D., Grønhøj C., Jensen S.B., Christensen R., Specht L., Andersen E., Andersen T.T., Ciochon U.M., Rathje G.S., Hansen A.E. (2022). Long-Term Safety of Treatment with Autologous Mesenchymal Stem Cells in Patients with Radiation-Induced Xerostomia: Primary Results of the MESRIX Phase I/II Randomized Trial. Clin. Cancer Res..

[472] Fenger Carlander A.-L., Jakobsen K.K., Todsen T., Paaske N., Østergaard Madsen A.K., Bendtsen S.K., Kastrup J., Friborg J., Duch Lynggaard C., Hauge A.W. (2025). Long-Term Effectiveness and Safety of Mesenchymal Stromal Cell Therapy for Radiation-Induced Hyposalivation in Head and Neck Cancer Survivors: A Randomized Phase II Trial. Clin. Cancer Res..

[473] Blitzer G.C., Glazer T., Burr A., Gustafson S., Ganz O., Meyers R., McDowell K.A., Nickel K.P., Mattison R.J., Weiss M. (2023). Marrow-Derived Autologous Stromal Cells for the Restoration of Salivary Hypofunction (MARSH): A Pilot, First-in-Human Study of Interferon Gamma-Stimulated Marrow Mesenchymal Stromal Cells for Treatment of Radiation-Induced Xerostomia. Cytotherapy.

[474] Lynggaard C.D., Jersie-Christensen R., Juhl M., Jensen S.B., Grønhøj C., Melchiors J., Jacobsen S., Møller-Hansen M., Herly M., Ekblond A. (2022). Intraglandular Mesenchymal Stem Cell Treatment Induces Changes in the Salivary Proteome of Irradiated Patients. Commun. Med..

[475] Kursova L.V., Konoplyannikov A.G., Pasov V.V., Ivanova I.N., Poluektova M.V., Konoplyannikova O.A. (2009). Possibilities for the Use of Autologous Mesenchymal Stem Cells in the Therapy of Radiation-Induced Lung Injuries. Bull. Exp. Biol. Med..

[476] Voswinkel J., Francois S., Simon J.-M., Benderitter M., Gorin N.-C., Mohty M., Fouillard L., Chapel A. (2013). Use of Mesenchymal Stem Cells (MSC) in Chronic Inflammatory Fistulizing and Fibrotic Diseases: A Comprehensive Review. Clin. Rev. Allergy Immunol..

[477] Gan J., Meng F., Zhou X., Li C., He Y., Zeng X., Jiang X., Liu J., Zeng G., Tang Y. (2015). Hematopoietic Recovery of Acute Radiation Syndrome by Human Superoxide Dismutase-Expressing Umbilical Cord Mesenchymal Stromal Cells. Cytotherapy.

[478] Chen H.-X., Xiang H., Xu W.-H., Li M., Yuan J., Liu J., Sun W.-J., Zhang R., Li J., Ren Z.-Q. (2017). Manganese Superoxide Dismutase Gene-Modified Mesenchymal Stem Cells Attenuate Acute Radiation-Induced Lung Injury. Hum. Gene Ther..

[479] Ghani F., Huang P., Zhang C., Zubair A.C. (2025). EGFR mRNA-Engineered Mesenchymal Stem Cells (MSCs) Demonstrate Radioresistance to Moderate Dose of Simulated Cosmic Radiation. Cells.

[480] Rühle A., Lies M., Strack M., Perez R.L., Bieber B., Thomsen A.R., Bronsert P., Huber P.E., Hess J., Knopf A. (2022). Human Mesenchymal Stromal Cells Do Not Cause Radioprotection of Head-and-Neck Squamous Cell Carcinoma. Int. J. Mol. Sci..

[481] Emerit J., Michelson A.M., Robert H.G., Chomette G., Guérin R.A., Blondon J., Bertrand M. (1983). Superoxide dismutase treatment of 2 cases of radiation-induced sclerosis. Sem. Hop..

[482] Yücel S., Şahin B., Güral Z., Olgaç V., Aksu G., Ağaoğlu F., Sağlam E., Aslay I., Darendeliler E. (2016). Impact of Superoxide Dismutase-Gliadin on Radiation-Induced Fibrosis: An Experimental Study. In Vivo.

[483] Delanian S., Baillet F., Huart J., Lefaix J.L., Maulard C., Housset M. (1994). Successful Treatment of Radiation-Induced Fibrosis Using Liposomal Cu/Zn Superoxide Dismutase: Clinical Trial. Radiother. Oncol..

[484] Vujaskovic Z., Batinic-Haberle I., Rabbani Z.N., Feng Q., Kang S.K., Spasojevic I., Samulski T.V., Fridovich I., Dewhirst M.W., Anscher M.S. (2002). A Small Molecular Weight Catalytic Metalloporphyrin Antioxidant with Superoxide Dismutase (SOD) Mimetic Properties Protects Lungs from Radiation-Induced Injury. Free Radic. Biol. Med..

[485] Campana F., Zervoudis S., Perdereau B., Gez E., Fourquet A., Badiu C., Tsakiris G., Koulaloglou S. (2004). Topical Superoxide Dismutase Reduces Post-Irradiation Breast Cancer Fibrosis. J. Cell. Mol. Med..

[486] Rabbani Z.N., Salahuddin F.K., Yarmolenko P., Batinic-Haberle I., Thrasher B.A., Gauter-Fleckenstein B., Dewhirst M.W., Anscher M.S., Vujaskovic Z. (2007). Low Molecular Weight Catalytic Metalloporphyrin Antioxidant AEOL 10150 Protects Lungs from Fractionated Radiation. Free Radic. Res..

[487] Yan S., Brown S.L., Kolozsvary A., Freytag S.O., Lu M., Kim J.H. (2008). Mitigation of Radiation-Induced Skin Injury by AAV2-Mediated MnSOD Gene Therapy. J. Gene Med..

[488] Gauter-Fleckenstein B., Fleckenstein K., Owzar K., Jian C., Batinic-Haberle I., Vujaskovic Z. (2008). Comparison of Two Mn Porphyrin-Based Mimics of Superoxide Dismutase (SOD) in Pulmonary Radioprotection. Free Radic. Biol. Med..

[489] Gauter-Fleckenstein B., Fleckenstein K., Owzar K., Jiang C., Rebouças J.S., Batinic-Haberle I., Vujaskovic Z. (2010). Early and Late Administration of MnTE-2-PyP5+ in Mitigation and Treatment of Radiation-Induced Lung Damage. Free Radic. Biol. Med..

[490] Li H., Wang Y., Pazhanisamy S.K., Shao L., Batinic-Haberle I., Meng A., Zhou D. (2011). Mn(III) Meso-Tetrakis-(N-Ethylpyridinium-2-Yl) Porphyrin Mitigates Total Body Irradiation-Induced Long-Term Bone Marrow Suppression. Free Radic. Biol. Med..

[491] Archambeau J.O., Tovmasyan A., Pearlstein R.D., Crapo J.D., Batinic-Haberle I. (2013). Superoxide Dismutase Mimic, MnTE-2-PyP(5+) Ameliorates Acute and Chronic Proctitis Following Focal Proton Irradiation of the Rat Rectum. Redox Biol..

[492] Doctrow S.R., Lopez A., Schock A.M., Duncan N.E., Jourdan M.M., Olasz E.B., Moulder J.E., Fish B.L., Mäder M., Lazar J. (2013). A Synthetic Superoxide Dismutase/Catalase Mimetic EUK-207 Mitigates Radiation Dermatitis and Promotes Wound Healing in Irradiated Rat Skin. J. Investig. Dermatol..

[493] Mahmood J., Jelveh S., Zaidi A., Doctrow S.R., Hill R.P. (2013). Mitigation of Radiation-Induced Lung Injury with EUK-207 and Genistein: Effects in Adolescent Rats. Radiat. Res..

[494] Garofalo M.C., Ward A.A., Farese A.M., Bennett A., Taylor-Howell C., Cui W., Gibbs A., Prado K.L., MacVittie T.J. (2014). A Pilot Study in Rhesus Macaques to Assess the Treatment Efficacy of a Small Molecular Weight Catalytic Metalloporphyrin Antioxidant (AEOL 10150) in Mitigating Radiation-Induced Lung Damage. Health Phys..

[495] Gauter-Fleckenstein B., Reboucas J.S., Fleckenstein K., Tovmasyan A., Owzar K., Jiang C., Batinic-Haberle I., Vujaskovic Z. (2014). Robust Rat Pulmonary Radioprotection by a Lipophilic Mn N-Alkylpyridylporphyrin, MnTnHex-2-PyP5+. Redox Biol..

[496] Murigi F.N., Mohindra P., Hung C., Salimi S., Goetz W., Pavlovic R., Jackson I.L., Vujaskovic Z. (2015). Dose Optimization Study of AEOL 10150 as a Mitigator of Radiation-Induced Lung Injury in CBA/J Mice. Radiat. Res..

[497] MacVittie T.J., Gibbs A., Farese A.M., Barrow K., Bennett A., Taylor-Howell C., Kazi A., Prado K., Parker G., Jackson W. (2017). AEOL 10150 Mitigates Radiation-Induced Lung Injury in the Nonhuman Primate: Morbidity and Mortality Are Administration Schedule-Dependent. Radiat. Res..

[498] Shrishrimal S., Kosmacek E.A., Chatterjee A., Tyson M.J., Oberley-Deegan R.E. (2017). The SOD Mimic, MnTE-2-PyP, Protects from Chronic Fibrosis and Inflammation in Irradiated Normal Pelvic Tissues. Antioxidants.

[499] Cline J.M., Dugan G., Bourland J.D., Perry D.L., Stitzel J.D., Weaver A.A., Jiang C., Tovmasyan A., Owzar K., Spasojevic I. (2018). Post-Irradiation Treatment with a Superoxide Dismutase Mimic, MnTnHex-2-PyP5+, Mitigates Radiation Injury in the Lungs of Non-Human Primates After Whole-Thorax Exposure to Ionizing Radiation. Antioxidants.

[500] Mapuskar K.A., Anderson C.M., Spitz D.R., Batinic-Haberle I., Allen B.G., Oberley-Deegan R. (2019). Utilizing Superoxide Dismutase Mimetics to Enhance Radiation Therapy Response While Protecting Normal Tissues. Semin. Radiat. Oncol..

[501] Ciuba D.F., Curtis A., Dunlap N., Siegel R.D., Biswas T., Wisbeck W.M., Berk L., Miller D.A., Holmlund J., Allen B.G. (2022). AESOP: Phase 2 Open-Label Trial of Avasopasem Manganese (GC4419) for Reduction of Esophagitis in Patients Receiving Chemoradiotherapy for Nonmetastatic Lung Cancer. Int. J. Radiat. Oncol. Biol. Phys..

[502] Anderson C.M., Lee C.M., Kelley J.R., Walker G.V., Dunlap N.E., Bar-Ad V.C., Miller D.A., King V.J., Peddada A.V., Ciuba D.F. (2022). ROMAN: Phase 3 Trial of Avasopasem Manganese (GC4419) for Severe Oral Mucositis (SOM) in Patients Receiving Chemoradiotherapy (CRT) for Locally Advanced, Nonmetastatic Head and Neck Cancer (LAHNC). J. Clin. Oncol..

[503] Brizel D.M., Mowery Y.M., Zhen W., Chan J., Macleod D., Yom S.S. (2021). A Phase 1-2 Trial of Concurrent Radiation Therapy, Cisplatin and BMX-001 in Locally Advanced Head and Neck Cancer. Int. J. Radiat. Oncol. Biol. Phys..

[504] Peters K., Cohen A., Butowski N., Villano J., Mendez J., Giglio P., McGranahan T., Zhang C., Smeltzer J., Raza S. (2023). LTBK-09. Results of BMX-HGG Study: A Multi-Institutional, Randomized Phase 2 Clinical Trial of Concurrent Chemoradiation with or Without BMX-001 in Patients with Newly Diagnosed High Grade Glioma. Neuro-oncology.

[505] Lin C., Grem J., Klute K., Crapo J., Filler-Katz A., Doherty E., Penchev S., Chatterjee A., Kosmacek E.A., Oberley-Deegan R.E. (2021). The Phase I Results of a Phase 1/2 Trial for Patients with Newly Diagnosed Anal Cancer Treated with Concurrent Radiation Therapy, 5-Fluorouracil, Mitomycin and BMX-001. Int. J. Radiat. Oncol. Biol. Phys..

[506] Landeen K.C., Spanos W.C., Gromer L. (2018). Topical Superoxide Dismutase in Posttreatment Fibrosis in Patients with Head and Neck Cancer. Head Neck.

[507] Anderson C., Lee C.M., Kelley J.R., Walker G.V., Dunlap N.E., Bar-Ad V.C., Miller D.A., King V.J., Peddada A.V., Ciuba D.F. (2025). Avasopasem Manganese Treatment for Severe Oral Mucositis from Chemoradiotherapy for Locally Advanced Head and Neck Cancer: Phase 3 Randomized Controlled Trial (ROMAN). eClinicalMedicine.

[508] Metz J.M., Smith D., Mick R., Lustig R., Mitchell J., Cherakuri M., Glatstein E., Hahn S.M. (2004). A Phase I Study of Topical Tempol for the Prevention of Alopecia Induced by Whole Brain Radiotherapy. Clin. Cancer Res..

[509] Citrin D., Valle L., Camphausen K., Cooley-Zgela T., Smart D., Yao M., Mitchell J.B., Thompson W., Sereti I., Uldrick T. (2022). Pilot Trial of Topical MTS-01 Application to Reduce Dermatitis in Patients Receiving Chemoradiotherapy for Stage I–III Carcinoma of the Anal Canal. Int. J. Oncol..

[510] Gu Q., Feng T., Cao H., Tang Y., Ge X., Luo J., Xue J., Wu J., Yang H., Zhang S. (2013). HIV-TAT Mediated Protein Transduction of Cu/Zn-Superoxide Dismutase-1 (SOD1) Protects Skin Cells from Ionizing Radiation. Radiat. Oncol..

[511] Vozenin-Brotons M.-C., Sivan V., Gault N., Renard C., Geffrotin C., Delanian S., Lefaix J.-L., Martin M. (2001). Antifibrotic Action of Cu/Zn SOD Is Mediated by TGF-Β1 Repression and Phenotypic Reversion of Myofibroblasts. Free Radic. Biol. Med..

[512] Batinic-Haberle I., Tovmasyan A., Spasojevic I. (2018). Mn Porphyrin-Based Redox-Active Drugs: Differential Effects as Cancer Therapeutics and Protectors of Normal Tissue Against Oxidative Injury. Antioxid. Redox Signal..

[513] Oberley-Deegan R.E., Steffan J.J., Rove K.O., Pate K.M., Weaver M.W., Spasojevic I., Frederick B., Raben D., Meacham R.B., Crapo J.D. (2012). The Antioxidant, MnTE-2-PyP, Prevents Side-Effects Incurred by Prostate Cancer Irradiation. PLoS ONE.

[514] Chatterjee A., Kosmacek E.A., Shrishrimal S., McDonald J.T., Oberley-Deegan R.E. (2020). MnTE-2-PyP, a Manganese Porphyrin, Reduces Cytotoxicity Caused by Irradiation in a Diabetic Environment Through the Induction of Endogenous Antioxidant Defenses. Redox Biol..

[515] Coudriet G.M., Delmastro-Greenwood M.M., Previte D.M., Marré M.L., O’Connor E.C., Novak E.A., Vincent G., Mollen K.P., Lee S., Dong H.H. (2017). Treatment with a Catalytic Superoxide Dismutase (SOD) Mimetic Improves Liver Steatosis, Insulin Sensitivity, and Inflammation in Obesity-Induced Type 2 Diabetes. Antioxidants.

[516] Chatterjee A., Zhu Y., Tong Q., Kosmacek E.A., Lichter E.Z., Oberley-Deegan R.E. (2018). The Addition of Manganese Porphyrins During Radiation Inhibits Prostate Cancer Growth and Simultaneously Protects Normal Prostate Tissue from Radiation Damage. Antioxidants.

[517] Cui W., Zhang P., Hankey K.G., Xiao M., Farese A.M., MacVittie T.J. (2021). AEOL 10150 Alleviates Radiation-Induced Innate Immune Responses in Non-Human Primate Lung Tissue. Health Phys..

[518] Zhao Y., Carroll D.W., You Y., Chaiswing L., Wen R., Batinic-Haberle I., Bondada S., Liang Y., St Clair D.K. (2017). A Novel Redox Regulator, MnTnBuOE-2-PyP5+, Enhances Normal Hematopoietic Stem/Progenitor Cell Function. Redox Biol..

[519] Ashcraft K.A., Boss M.-K., Tovmasyan A., Roy Choudhury K., Fontanella A.N., Young K.H., Palmer G.M., Birer S.R., Landon C.D., Park W. (2015). Novel Manganese-Porphyrin Superoxide Dismutase-Mimetic Widens the Therapeutic Margin in a Preclinical Head and Neck Cancer Model. Int. J. Radiat. Oncol. Biol. Phys..

[520] Birer S.R., Lee C.-T., Choudhury K.R., Young K.H., Spasojevic I., Batinic-Haberle I., Crapo J.D., Dewhirst M.W., Ashcraft K.A. (2017). Inhibition of the Continuum of Radiation-Induced Normal Tissue Injury by a Redox-Active Mn Porphyrin. Radiat. Res..

[521] Leu D., Spasojevic I., Nguyen H., Deng B., Tovmasyan A., Weitner T., Sampaio R.S., Batinic-Haberle I., Huang T.-T. (2017). CNS Bioavailability and Radiation Protection of Normal Hippocampal Neurogenesis by a Lipophilic Mn Porphyrin-Based Superoxide Dismutase Mimic, MnTnBuOE-2-PyP5. Redox Biol..

[522] Weitzel D.H., Tovmasyan A., Ashcraft K.A., Rajic Z., Weitner T., Liu C., Li W., Buckley A.F., Prasad M.R., Young K.H. (2015). Radioprotection of the Brain White Matter by Mn(III) n-Butoxyethylpyridylporphyrin-Based Superoxide Dismutase Mimic MnTnBuOE-2-PyP5+. Mol. Cancer Ther..

[523] Weitzel D.H., Tovmasyan A., Ashcraft K.A., Boico A., Birer S.R., Roy Choudhury K., Herndon J., Rodriguiz R.M., Wetsel W.C., Peters K.B. (2016). Neurobehavioral Radiation Mitigation to Standard Brain Cancer Therapy Regimens by Mn(III) n-Butoxyethylpyridylporphyrin-Based Redox Modifier. Environ. Mol. Mutagen..

[524] Kosmacek E.A., Chatterjee A., Tong Q., Lin C., Oberley-Deegan R.E. (2016). MnTnBuOE-2-PyP Protects Normal Colorectal Fibroblasts from Radiation Damage and Simultaneously Enhances Radio/Chemotherapeutic Killing of Colorectal Cancer Cells. Oncotarget.

[525] Patel A., Kosmacek E.A., Fisher K.W., Goldner W., Oberley-Deegan R.E. (2020). MnTnBuOE-2-PyP Treatment Protects from Radioactive Iodine (I-131) Treatment-Related Side Effects in Thyroid Cancer. Radiat. Environ. Biophys..

[526] Schlichte S.L., Romanova S., Katsurada K., Kosmacek E.A., Bronich T.K., Patel K.P., Oberley-Deegan R.E., Zimmerman M.C. (2020). Nanoformulation of the Superoxide Dismutase Mimic, MnTnBuOE-2-PyP5+, Prevents Its Acute Hypotensive Response. Redox Biol..

[527] El-Mahdy M.A., Alzarie Y.A., Hemann C., Badary O.A., Nofal S., Zweier J.L. (2020). The Novel SOD Mimetic GC4419 Increases Cancer Cell Killing with Sensitization to Ionizing Radiation While Protecting Normal Cells. Free Radic. Biol. Med..

[528] Sishc B.J., Polsdofer E., Bloom D.A., Heer C., Spitz D.R., Saha D., Story M.D. (2018). Abstract 667: The Radioprotector GC4419 Ameliorates Radiation Induced Lung Fibrosis While Enhancing the Response of Non-Small Cell Lung Cancer Tumors to High Dose per Fraction Radiation Exposures. Cancer Res..

[529] Sishc B.J., Ramnarain D., Shang Z., Alves E.M., Bloom D.A., Hughes K., Saha D., Riley D.P., Keene J.L., Beardsley R.A. (2026). Avasopasem Manganese Acts as Both a Radioprotector and a Radiomitigator of Radiation-Induced Acute or Late Effects. Front. Oncol..

[530] Anderson C.M., Lee C.M., Saunders D.P., Curtis A., Dunlap N., Nangia C., Lee A.S., Gordon S.M., Kovoor P., Arevalo-Araujo R. (2019). Phase IIb, Randomized, Double-Blind Trial of GC4419 Versus Placebo to Reduce Severe Oral Mucositis Due to Concurrent Radiotherapy and Cisplatin For Head and Neck Cancer. J. Clin. Oncol..

[531] Anderson C.M., Lee C.M., Saunders D., Curtis A.E., Dunlap N.E., Nangia C., Lee A.S., Kovoor P., Bar-Ad V., Pedadda A.V. (2022). Two-Year Tumor Outcomes of a Phase 2B, Randomized, Double-Blind Trial of Avasopasem Manganese (GC4419) Versus Placebo to Reduce Severe Oral Mucositis Owing to Concurrent Radiation Therapy and Cisplatin for Head and Neck Cancer. Int. J. Radiat. Oncol. Biol. Phys..

[532] Anderson C., Salvaggio S., De Backer M., Chiem J.-C., Walker G., Saunders D., Lee C.M., Dunlap N., Kennedy E., Beardsley R. (2025). Benefit of Avasopasem Manganese on Severe Oral Mucositis in Head and Neck Cancer in the ROMAN Trial: Unplanned Secondary Analysis. Adv. Radiat. Oncol..

[533] Anderson C.M., Lee C.M., Kelley J.R., Walker G.V., Dunlap N., Bar-Ad V., Miller D.A., King V.J., Peddada A., Ciuba D.F. (2022). Tumor Outcomes for ROMAN: Phase 3 Trial of Avasopasem Manganese (GC4419) for Severe Oral Mucositis (SOM) in Patients Receiving Chemoradiotherapy (CRT) for Locally Advanced Head and Neck Cancer (LAHNC). Int. J. Radiat. Oncol. Biol. Phys..

[534] Sishc B.J., Ding L., Nam T.-K., Heer C.D., Rodman S.N., Schoenfeld J.D., Fath M.A., Saha D., Pulliam C.F., Langen B. (2021). Avasopasem Manganese Synergizes with Hypofractionated Radiation to Ablate Tumors Through the Generation of Hydrogen Peroxide. Sci. Transl. Med..

[535] Taniguchi C.M., Frakes J.M., Aguilera T.A., Palta M., Czito B., Bhutani M.S., Colbert L.E., Abi Jaoude J., Bernard V., Pant S. (2023). Stereotactic Body Radiotherapy with or Without Selective Dismutase Mimetic in Pancreatic Adenocarcinoma: An Adaptive, Randomised, Double-Blind, Placebo-Controlled, Phase 1b/2 Trial. Lancet Oncol..

[536] Zaher A., Mapuskar K.A., Petronek M.S., Tanas M.R., Isaacson A.L., Dodd R.D., Milhem M., Furqan M., Spitz D.R., Miller B.J. (2024). Superoxide Dismutase Mimetic Avasopasem Manganese Enhances Radiation Therapy Effectiveness in Soft Tissue Sarcomas and Accelerates Wound Healing. Antioxidants.

[537] Batinic-Haberle I., Tovmasyan A., Huang Z., Duan W., Du L., Siamakpour-Reihani S., Cao Z., Sheng H., Spasojevic I., Alvarez Secord A. (2021). H2O2-Driven Anticancer Activity of Mn Porphyrins and the Underlying Molecular Pathways. Oxidative Med. Cell. Longev..

[538] Borrelli A., Schiattarella A., Mancini R., Morrica B., Cerciello V., Mormile M., d’Alesio V., Bottalico L., Morelli F., D’Armiento M. (2009). A Recombinant MnSOD Is Radioprotective for Normal Cells and Radiosensitizing for Tumor Cells. Free Radic. Biol. Med..

[539] Cuscela D., Coffin D., Lupton G.P., Cook J.A., Krishna M.C., Bonner R.F., Mitchell J.B. (1996). Protection from Radiation-Induced Alopecia with Topical Application of Nitroxides: Fractionated Studies. Cancer J. Sci. Am..

[540] Ramachandran L., Nair C.K.K. (2012). Prevention of γ-Radiation Induced Cellular Genotoxicity by Tempol: Protection of Hematopoietic System. Environ. Toxicol. Pharmacol..

[541] Goff J.P., Epperly M.W., Dixon T., Wang H., Franicola D., Shields D., Wipf P., Li S., Gao X., Greenberger J.S. (2011). Radiobiologic Effects of GS-Nitroxide (JP4-039) on the Hematopoietic Syndrome. In Vivo.

[542] Epperly M.W., Sacher J.R., Krainz T., Zhang X., Wipf P., Liang M., Fisher R., Li S., Wang H., Greenberger J.S. (2017). Effectiveness of Analogs of the GS-Nitroxide, JP4-039, as Total Body Irradiation Mitigators. In Vivo.

[543] Wei L., Leibowitz B.J., Epperly M., Bi C., Li A., Steinman J., Wipf P., Li S., Zhang L., Greenberger J. (2018). The GS-Nitroxide JP4-039 Improves Intestinal Barrier and Stem Cell Recovery in Irradiated Mice. Sci. Rep..

[544] Wu Y.L., Christodoulou A.G., Beumer J.H., Rigatti L.H., Fisher R., Ross M., Watkins S., Cortes D.R.E., Ruck C., Manzoor S. (2024). Mitigation of Fetal Radiation Injury from Mid-Gestation Total-Body Irradiation by Maternal Administration of Mitochondrial-Targeted GS-Nitroxide JP4-039. Radiat. Res..

[545] Soule B.P., Hyodo F., Matsumoto K., Simone N.L., Cook J.A., Krishna M.C., Mitchell J.B. (2007). The Chemistry and Biology of Nitroxide Compounds. Free Radic. Biol. Med..

[546] Mitchell J.B., Anver M.R., Sowers A.L., Rosenberg P.S., Figueroa M., Thetford A., Krishna M.C., Albert P.S., Cook J.A. (2012). The Antioxidant Tempol Reduces Carcinogenesis and Enhances Survival in Mice When Administered After Non-Lethal Total Body Radiation. Cancer Res..

[547] Georgakilas A.G., Redon C.E., Ferguson N.F., Kryston T.B., Parekh P., Dickey J.S., Nakamura A.J., Mitchell J.B., Bonner W.M., Martin O.A. (2014). Systemic DNA Damage Accumulation Under in Vivo Tumor Growth Can Be Inhibited by the Antioxidant Tempol. Cancer Lett..

[548] Brand R.M., Epperly M.W., Stottlemyer J.M., Skoda E.M., Gao X., Li S., Huq S., Wipf P., Kagan V.E., Greenberger J.S. (2017). A Topical Mitochondria-Targeted Redox Cycling Nitroxide Mitigates Oxidative Stress Induced Skin Damage. J. Investig. Dermatol..

[549] Hofer M., Hoferová Z., Falk M. (2019). Brief Story on Prostaglandins, Inhibitors of Their Synthesis, Hematopoiesis, and Acute Radiation Syndrome. Molecules.

[550] Pospísil M., Netíková J., Kozubík A., Pipalová I. (1989). Effect of Indomethacin, Diclofenac Sodium and Sodium Salicylate on Peripheral Blood Cell Counts in Sublethally Gamma-Irradiated Mice. Strahlenther. Onkol..

[551] Hofer M., Pospísil M., Pipalová I., Holá J. (1996). Modulation of Haemopoietic Radiation Response of Mice by Diclofenac in Fractionated Treatment. Physiol. Res..

[552] Alok A., Adhikari J.S., Chaudhury N.K. (2013). Radioprotective Role of Clinical Drug Diclofenac Sodium. Mutat. Res. Genet. Toxicol. Environ. Mutagen..

[553] Gilman K.E., Camden J.M., Woods L.T., Weisman G.A., Limesand K.H. (2021). Indomethacin Treatment Post-Irradiation Improves Mouse Parotid Salivary Gland Function via Modulation of Prostaglandin E2 Signaling. Front. Bioeng. Biotechnol..

[554] Pillsbury H.C., Webster W.P., Rosenman J. (1986). Prostaglandin Inhibitor and Radiotherapy in Advanced Head and Neck Cancers. Arch. Otolaryngol. Head Neck Surg..

[555] Nagaoka H., Momo K., Hamano J., Miyaji T., Oyamada S., Kawaguchi T., Homma M., Yamaguchi T., Morita T., Kizawa Y. (2021). Effects of an Indomethacin Oral Spray on Pain Due to Oral Mucositis in Cancer Patients Treated with Radiotherapy and Chemotherapy: A Double-Blind, Randomized, Placebo-Controlled Trial (JORTC-PAL04). J. Pain Symptom Manag..

[556] Epstein J.B., Silverman S., Paggiarino D.A., Crockett S., Schubert M.M., Senzer N.N., Lockhart P.B., Gallagher M.J., Peterson D.E., Leveque F.G. (2001). Benzydamine HCl for Prophylaxis of Radiation-Induced Oral Mucositis: Results from a Multicenter, Randomized, Double-Blind, Placebo-Controlled Clinical Trial. Cancer.

[557] Kazemian A., Kamian S., Aghili M., Hashemi F.A., Haddad P. (2009). Benzydamine for Prophylaxis of Radiation-Induced Oral Mucositis in Head and Neck Cancers: A Double-Blind Placebo-Controlled Randomized Clinical Trial. Eur. J. Cancer Care.

[558] Sheibani K.M., Mafi A.R., Moghaddam S., Taslimi F., Amiran A., Ameri A. (2015). Efficacy of Benzydamine Oral Rinse in Prevention and Management of Radiation-Induced Oral Mucositis: A Double-Blind Placebo-Controlled Randomized Clinical Trial. Asia-Pac. J. Clin. Oncol..

[559] Chitapanarux I., Tungkasamit T., Petsuksiri J., Kannarunimit D., Katanyoo K., Chakkabat C., Setakornnukul J., Wongsrita S., Jirawatwarakul N., Lertbusayanukul C. (2018). Randomized Control Trial of Benzydamine HCl versus Sodium Bicarbonate for Prophylaxis of Concurrent Chemoradiation-Induced Oral Mucositis. Support. Care Cancer.

[560] Elsaadany B., Anayb S.M., Mashhour K., Yossif M., Zahran F. (2024). Rebamipide Gargle and Benzydamine Gargle in Prevention and Management of Chemo-Radiotherapy and Radiotherapy-Induced Oral Mucositis in Head and Neck Cancer Patients (Randomized Clinical Trial). BMC Oral Health.

[561] Wang L.-W., Hsiao C.-F., Chen W.T.-L., Lee H.-H., Lin T.-C., Chen H.-C., Chen H.-H., Chien C.-R., Lin T.-Y., Liu T.-W. (2014). Celecoxib plus Chemoradiotherapy for Locally Advanced Rectal Cancer: A Phase II TCOG Study. J. Surg. Oncol..

[562] Araujo-Mino E.P., Patt Y.Z., Murray-Krezan C., Hanson J.A., Bansal P., Liem B.J., Rajput A., Fekrazad M.H., Heywood G., Lee F.C. (2018). Phase II Trial Using a Combination of Oxaliplatin, Capecitabine, and Celecoxib with Concurrent Radiation for Newly Diagnosed Resectable Rectal Cancer. Oncologist.

[563] Gaber Ayad K.A., Azmy A.M., Ezz El Din M.M.A., Mosalam N.A., Saleh El-Sayed M.E. (2024). A Phase II Trial of Celecoxib with Neoadjuvant Concurrent Chemoradiation for Patients with Localized Rectal Adenocarcinoma Stage II and III. QJM.

[564] Ghasemi A., Danesh B., Yazdani-Charati J., Hosseinimehr S.J. (2018). Randomized Double-Blind Placebo-Controlled Trial of Celecoxib for the Prevention of Skin Toxicity in Patients Receiving Radiation Therapy for Breast Cancer. Anti-Inflamm. Anti-Allergy Agents Med. Chem..

[565] Bi N., Liang J., Zhou Z., Chen D., Fu Z., Yang X., Feng Q., Hui Z., Xiao Z., Lv J. (2019). Effect of Concurrent Chemoradiation with Celecoxib vs Concurrent Chemoradiation Alone on Survival Among Patients with Non-Small Cell Lung Cancer with and Without Cyclooxygenase 2 Genetic Variants: A Phase 2 Randomized Clinical Trial. JAMA Netw. Open.

[566] Lalla R.V., Choquette L.E., Curley K.F., Dowsett R.J., Feinn R.S., Hegde U.P., Pilbeam C.C., Salner A.L., Sonis S.T., Peterson D.E. (2014). Randomized Double-Blind Placebo-Controlled Trial of Celecoxib for Oral Mucositis in Patients Receiving Radiation Therapy for Head and Neck Cancer. Oral Oncol..

[567] Aghili M., Ghalehtaki R., Rayzan E., Farzin M., Mojahed M.M., Izadi S., Kazemian A. (2021). The Efficacy of Celecoxib During Chemoradiation in Locally Advanced Head and Neck Carcinoma; A Phase 2 Randomized Placebo-Controlled Clinical Trial. Int. J. Cancer Manag..

[568] Dajani E.Z., Islam K. (2008). Cardiovascular and Gastrointestinal Toxicity of Selective Cyclo-Oxygenase-2 Inhibitors in Man. J. Physiol. Pharmacol..

[569] Hofer M., Pospíšil M., Znojil V., Holá J., Vacek A., Weiterová L., Štreitová D., Kozubík A. (2006). Meloxicam, a Cyclooxygenase 2 Inhibitor, Supports Hematopoietic Recovery in Gamma-Irradiated Mice. Radiat. Res..

[570] Hofer M., Pospíšil M., Znojil V., Holá J., Vacek A., Štreitová D. (2008). Meloxicam, an Inhibitor of Cyclooxygenase-2, Increases the Level of Serum G-CSF and Might Be Usable as an Auxiliary Means in G-CSF Therapy. Physiol. Res..

[571] Hofer M., Pospíšil M., Dušek L., Hoferová Z., Weiterová L. (2011). A Single Dose of an Inhibitor of Cyclooxygenase 2, Meloxicam, Administered Shortly After Irradiation Increases Survival of Lethally Irradiated Mice. Radiat. Res..

[572] Hoggatt J., Singh P., Stilger K.N., Plett P.A., Sampson C.H., Chua H.L., Orschell C.M., Pelus L.M. (2013). Recovery from Hematopoietic Injury by Modulating Prostaglandin E(2) Signaling Post-Irradiation. Blood Cells Mol. Dis..

[573] Hofer M., Pospíšil M., Dušek L., Hoferová Z., Komůrková D. (2014). Agonist of the Adenosine A3 Receptor, IB-MECA, and Inhibitor of Cyclooxygenase-2, Meloxicam, given Alone or in a Combination Early After Total Body Irradiation Enhance Survival of γ-Irradiated Mice. Radiat. Environ. Biophys..

[574] Scripcariu D.V., Caratașu C.C., Ciorpac M., Alexa-Stratulat T., Szilagyi A., Buga C.R., Dobrovăț B.I., Eva L., Timofte A.D., Lozneanu L. (2025). Comparative Efficacy of Pirfenidone and Meloxicam on Early Radiotherapy-Induced Anal Sphincter Dysfunction in Rats. Front. Pharmacol..

[575] Han L., Ren Q. (2014). Protective effect of meloxicam against acute radiation-induced brain injury in rats. Xi Bao Yu Fen Zi Mian Yi Xue Za Zhi.

[576] Liang L., Hu D., Liu W., Williams J.P., Okunieff P., Ding I. (2003). Celecoxib Reduces Skin Damage After Radiation: Selective Reduction of Chemokine and Receptor mRNA Expression in Irradiated Skin but Not in Irradiated Mammary Tumor. Am. J. Clin. Oncol..

[577] Hunter N.R., Valdecanas D., Liao Z., Milas L., Thames H.D., Mason K.A. (2013). Mitigation and Treatment of Radiation-Induced Thoracic Injury with a Cyclooxygenase-2 Inhibitor, Celecoxib. Int. J. Radiat. Oncol. Biol. Phys..

[578] Feigenberg S.J., Wolk K.L., Yang C.-H., Morris C.G., Zlotecki R.A. (2003). Celecoxib to Decrease Urinary Retention Associated with Prostate Brachytherapy. Brachytherapy.

[579] Mohammadianpanah M., Razmjou-Ghalaei S., Shafizad A., Ashouri-Taziani Y., Khademi B., Ahmadloo N., Ansari M., Omidvari S., Mosalaei A., Mosleh-Shirazi M.A. (2011). Efficacy and Safety of Concurrent Chemoradiation with Weekly Cisplatin ± Low-Dose Celecoxib in Locally Advanced Undifferentiated Nasopharyngeal Carcinoma: A Phase II-III Clinical Trial. J. Cancer Res. Ther..

[580] Cheki M., Yahyapour R., Farhood B., Rezaeyan A., Shabeeb D., Amini P., Rezapoor S., Najafi M. (2018). COX-2 in Radiotherapy: A Potential Target for Radioprotection and Radiosensitization. Curr. Mol. Pharmacol..

[581] Rosas C., Sinning M., Ferreira A., Fuenzalida M., Lemus D. (2014). Celecoxib Decreases Growth and Angiogenesis and Promotes Apoptosis in a Tumor Cell Line Resistant to Chemotherapy. Biol. Res..

[582] Zhang P., He D., Song E., Jiang M., Song Y. (2019). Celecoxib Enhances the Sensitivity of Non-Small-Cell Lung Cancer Cells to Radiation-Induced Apoptosis Through Downregulation of the Akt/mTOR Signaling Pathway and COX-2 Expression. PLoS ONE.

[583] Nikolaeva-Koleva M., Espinosa A., Vergassola M., Polenzani L., Mangano G., Ragni L., Zucchi S., Ferrer-Montiel A., Devesa I. (2023). Benzydamine Plays a Role in Limiting Inflammatory Pain Induced by Neuronal Sensitization. Mol. Pain.

[584] Shah S., Rath H., Sharma G., Senapati S.N., Mishra E. (2020). Effectiveness of Curcumin Mouthwash on Radiation-Induced Oral Mucositis Among Head and Neck Cancer Patients: A Triple-Blind, Pilot Randomised Controlled Trial. Indian J. Dent. Res..

[585] Thomas P.L., Kang H.K., Rishi K.S. (2023). Randomized Control Study of the Effects of Turmeric Mouthwash on Oral Health Status, Treatment-Induced Mucositis, and Associated Oral Dysfunctions Among Patients with Head and Neck Cancer. Cancer Nurs..

[586] Mohandas R., Mohapatra S. (2025). Comparative Evaluation of the Efficacy of Herbal and Benzydamine Mouthwashes in Preventing Radiation-Induced Oral Mucositis Among Head and Neck Cancer Patients: A Systematic Review and Network Meta-Analysis. Evid. Based Dent..

[587] Xu Y., Rong X., Hu W., Huang X., Li Y., Zheng D., Cai Z., Zuo Z., Tang Y. (2018). Bevacizumab Monotherapy Reduces Radiation-Induced Brain Necrosis in Nasopharyngeal Carcinoma Patients: A Randomized Controlled Trial. Int. J. Radiat. Oncol. Biol. Phys..

[588] Kroon L.L., Dieleman E.M.T., Keil V.C., van Barreveld M., Dijkgraaf M.G.W., Topff L., Wagemakers S.N., Snijders T.J., Vos M.J., Koekkoek J.A.F. (2025). Bevacizumab for Symptomatic Cerebral Radiation Necrosis After Radiation of High-Grade Glioma or Brain Metastases—When and for Whom?. Neuro-Oncol. Pract..

[589] Aslan A., Kaya Z.B., Bulduk E.B., Ocal O., Ucar M., Erpolat O.P., Kaymaz F., Borcek A.O. (2018). Prophylactic Bevacizumab May Mitigate Radiation Injury: An Experimental Study. World Neurosurg..

[590] Zhang J., Yu J., Yang D., Jiang L., Dong X., Liu Z., Yu R., Yu H., Shi A. (2024). Bevacizumab Reduces Cerebral Radiation Necrosis Due to Stereotactic Radiotherapy in Non-Small Cell Lung Cancer Patients with Brain Metastases: An Inverse Probability of Treatment Weighting Analysis. Front. Immunol..

[591] Handa E., Puspitasari I.M., Abdulah R., Yamazaki C., Kameo S., Nakano T., Koyama H. (2020). Recent Advances in Clinical Studies of Selenium Supplementation in Radiotherapy. J. Trace Elem. Med. Biol..

[592] Sieber F., Muir S.A., Cohen E.P., North P.E., Fish B.L., Irving A.A., Mäder M., Moulder J.E. (2009). High-Dose Selenium for the Mitigation of Radiation Injury: A Pilot Study in a Rat Model. Radiat. Res..

[593] Sieber F., Muir S.A., Cohen E.P., Fish B.L., Mäder M., Schock A.M., Althouse B.J., Moulder J.E. (2011). Dietary Selenium for the Mitigation of Radiation Injury: Effects of Selenium Dose Escalation and Timing of Supplementation. Radiat. Res..

[594] Kolivand S., Amini P., Saffar H., Rezapoor S., Najafi M., Motevaseli E., Nouruzi F., Shabeeb D., Eleojo Musa A. (2019). Selenium-L-Methionine Modulates Radiation Injury and Duox1 and Duox2 Upregulation in Rat’s Heart Tissues. J. Cardiovasc. Thorac. Res..

[595] Amini P., Kolivand S., Saffar H., Rezapoor S., Motevaseli E., Najafi M., Nouruzi F., Shabeeb D., Musa A.E. (2019). Protective Effect of Selenium-L-Methionine on Radiation-Induced Acute Pneumonitis and Lung Fibrosis in Rat. Curr. Clin. Pharmacol..

[596] Yazdi M.H., Masoudifar M., Varastehmoradi B., Mohammadi E., Kheradmand E., Homayouni S., Shahverdi A.R. (2013). Effect of Oral Supplementation of Biogenic Selenium Nanoparticles on White Blood Cell Profile of BALB/c Mice and Mice Exposed to X-Ray Radiation. Avicenna J. Med. Biotechnol..

[597] Karami M., Asri-Rezaei S., Dormanesh B., Nazarizadeh A. (2018). Comparative Study of Radioprotective Effects of Selenium Nanoparticles and Sodium Selenite in Irradiation-Induced Nephropathy of Mice Model. Int. J. Radiat. Biol..

[598] Sohrabi A., Tehrani A.A., Asri-Rezaei S., Zeinali A., Norouzi M. (2020). Histopathological Assessment of Protective Effects of Selenium Nanoparticles on Rat Hepatocytes Exposed to Gamma Radiation. Vet. Res. Forum.

[599] Azmoonfar R., Moslehi M., Shahbazi-Gahrouei D. (2024). Radioprotective Effect of Selenium Nanoparticles: A Mini Review. IET Nanobiotechnol..

[600] Büntzel J., Riesenbeck D., Glatzel M., Berndt-Skorka R., Riedel T., Mücke R., Kisters K., Schönekaes K.G., Schäfer U., Bruns F. (2010). Limited Effects of Selenium Substitution in the Prevention of Radiation-Associated Toxicities. Results of a Randomized Study in Head and Neck Cancer Patients. Anticancer. Res..

[601] Muecke R., Micke O., Schomburg L., Glatzel M., Reichl B., Kisters K., Schaefer U., Huebner J., Eich H.T., Fakhrian K. (2014). Multicenter, Phase III Trial Comparing Selenium Supplementation with Observation in Gynecologic Radiation Oncology: Follow-up Analysis of the Survival Data 6 Years After Cessation of Randomization. Integr. Cancer Ther..

[602] Muecke R., Micke O., Schomburg L., Buentzel J., Kisters K., Adamietz I.A. (2018). AKTE Selenium in Radiation Oncology—15 Years of Experiences in Germany. Nutrients.

[603] Micke O., Bruns F., Mücke R., Schäfer U., Glatzel M., DeVries A.F., Schönekaes K., Kisters K., Büntzel J. (2003). Selenium in the Treatment of Radiation-Associated Secondary Lymphedema. Int. J. Radiat. Oncol. Biol. Phys..

[604] Muecke R., Schomburg L., Glatzel M., Berndt-Skorka R., Baaske D., Reichl B., Buentzel J., Kundt G., Prott F.J., Devries A. (2010). Multicenter, Phase 3 Trial Comparing Selenium Supplementation with Observation in Gynecologic Radiation Oncology. Int. J. Radiat. Oncol. Biol. Phys..

[605] Jahangard-Rafsanjani Z., Gholami K., Hadjibabaie M., Shamshiri A.R., Alimoghadam K., Sarayani A., Mojtahedzadeh M., Ostadali-Dehaghi M., Ghavamzadeh A. (2013). The Efficacy of Selenium in Prevention of Oral Mucositis in Patients Undergoing Hematopoietic SCT: A Randomized Clinical Trial. Bone Marrow Transplant..

[606] Elango S., Subbiah U. (2015). Influence of Selenium on Radiogenic Collagen Destruction and the Degree of Collagen Tissue Maturation in Stage III Oral Squamous Cell Carcinoma Patients Undergoing Therapeutic Irradiation. J. Cancer Res. Ther..

[607] Son H., Lee S.M., Yoon R.G., Lee H., Lee I., Kim S., Chung W.Y., Lee J.W. (2017). Effect of Selenium Supplementation for Protection of Salivary Glands from Iodine-131 Radiation Damage in Patients with Differentiated Thyroid Cancer. Hell. J. Nucl. Med..

[608] Pakdaman A. (1998). Symptomatic Treatment of Brain Tumor Patients with Sodium Selenite, Oxygen, and Other Supportive Measures. Biol. Trace Elem. Res..

[609] Mix M., Singh A.K., Tills M., Dibaj S., Groman A., Jaggernauth W., Rustum Y., Jameson M.B. (2015). Randomized Phase II Trial of Selenomethionine as a Modulator of Efficacy and Toxicity of Chemoradiation in Squamous Cell Carcinoma of the Head and Neck. World J. Clin. Oncol..

[610] Mix M., Ramnath N., Gomez J., de Groot C., Rajan S., Dibaj S., Tan W., Rustum Y., Jameson M.B., Singh A.K. (2015). Effects of Selenomethionine on Acute Toxicities from Concurrent Chemoradiation for Inoperable Stage III Non-Small Cell Lung Cancer. World J. Clin. Oncol..

[611] Laali E., Manifar S., Kazemian A., Jahangard-Rafsanjani Z., Gholami K. (2020). Effect of Selenium on Incidence and Severity of Mucositis During Radiotherapy in Patients with Head and Neck Cancer. Oral Health Prev. Dent..

[612] Ripamonti C., Zecca E., Brunelli C., Fulfaro F., Villa S., Balzarini A., Bombardieri E., De Conno F. (1998). A Randomized, Controlled Clinical Trial to Evaluate the Effects of Zinc Sulfate on Cancer Patients with Taste Alterations Caused by Head and Neck Irradiation. Cancer.

[613] Ertekin M.V., Koç M., Karslioglu I., Sezen O. (2004). Zinc Sulfate in the Prevention of Radiation-Induced Oropharyngeal Mucositis: A Prospective, Placebo-Controlled, Randomized Study. Int. J. Radiat. Oncol. Biol. Phys..

[614] Halyard M.Y., Jatoi A., Sloan J.A., Bearden J.D., Vora S.A., Atherton P.J., Perez E.A., Soori G., Zalduendo A.C., Zhu A. (2007). Does Zinc Sulfate Prevent Therapy-Induced Taste Alterations in Head and Neck Cancer Patients? Results of Phase III Double-Blind, Placebo-Controlled Trial from the North Central Cancer Treatment Group (N01C4). Int. J. Radiat. Oncol. Biol. Phys..

[615] Mosalaei A., Nasrolahi H., Shafizad A., Ahmadloo N., Ansari M., Mosleh-Shirazi M.A., Omidvari S., Mohammadianpanah M. (2010). Effect of Oral Zinc Sulphate in Prevention of Radiation Induced Oropharyngeal Mucositis During and After Radiotherapy in Patients with Head and Neck Cancers. Middle East J. Cancer.

[616] Gorgu S.Z., Ilknur A.F., Sercan O., Rahsan H., Nalan A. (2013). The Effect of Zinc Sulphate in the Prevention of Radiation Induced Oral Mucositis in Patents with Head and Neck Cancer. Int. J. Radiat. Res..

[617] Najafizade N., Hemati S., Gookizade A., Berjis N., Hashemi M., Vejdani S., Ghannadi A., Shahsanaee A., Arbab N. (2013). Preventive Effects of Zinc Sulfate on Taste Alterations in Patients Under Irradiation for Head and Neck Cancers: A Randomized Placebo-Controlled Trial. J. Res. Med. Sci..

[618] Sangthawan D., Phungrassami T., Sinkitjarurnchai W. (2013). A Randomized Double-Blind, Placebo-Controlled Trial of Zinc Sulfate Supplementation for Alleviation of Radiation-Induced Oral Mucositis and Pharyngitis in Head and Neck Cancer Patients. J. Med. Assoc. Thai.

[619] Anandhi P., Sharief R.M., Rahila C. (2020). The Benefit of Zinc Sulfate in Oropharyngeal Mucositis During Hyperfractionated Accelerated Concomitant Boost Radiotherapy with Concurrent Cisplatin for Advanced-Stage Oropharyngeal and Hypopharyngeal Cancers. Indian J. Palliat. Care.

[620] Sahebnasagh M., Aksi V., Eslami F., Lashkardoost H., Kasaian J., Golmohammadzadeh S., Parkam B., Negarandeh R., Saghafi F., Sahebnasagh A. (2023). Prevention of Radiotherapy-Related Oral Mucositis with Zinc and Polyherbal Mouthwash: A Double-Blind, Randomized Clinical Trial. Eur. J. Med. Res..

[621] Aflatoonian S., Shamsi S., Ilaghi M., Yazdani S., Eghbalian M., Bahador M. (2025). Zinc Sulphate for Prevention of Breast Cancer Radiation Therapy-Induced Dermatitis: A Three-Arm Triple-Blinded Randomized Clinical Trial. Iran. J. Blood Cancer.

[622] Moslemi D., Babaee N., Damavandi M., Pourghasem M., Moghadamnia A.A. (2014). Oral Zinc Sulphate and Prevention of Radiation-Induced Oropharyngealmucositis in Patients with Head and Neck Cancers: A Double Blind, Randomized Controlled Clinical Trial. Int. J. Radiat. Res..

[623] Watanabe T., Ishihara M., Matsuura K., Mizuta K., Itoh Y. (2010). Polaprezinc Prevents Oral Mucositis Associated with Radiochemotherapy in Patients with Head and Neck Cancer. Int. J. Cancer.

[624] Doi H., Fujiwara M., Suzuki H., Niwa Y., Nakayama M., Shikata T., Odawara S., Takada Y., Kimura T., Kamikonya N. (2015). Polaprezinc Reduces the Severity of Radiation-Induced Mucositis in Head and Neck Cancer Patients. Mol. Clin. Oncol..

[625] Suzuki A., Kobayashi R., Shakui T., Kubota Y., Fukita M., Kuze B., Aoki M., Sugiyama T., Mizuta K., Itoh Y. (2016). Effect of Polaprezinc on Oral Mucositis, Irradiation Period, and Time to Discharge in Patients with Head and Neck Cancer. Head Neck.

[626] Yanase K., Funaguchi N., Iihara H., Yamada M., Kaito D., Endo J., Ito F., Ohno Y., Tanaka H., Itoh Y. (2015). Prevention of Radiation Esophagitis by Polaprezinc (Zinc L-Carnosine) in Patients with Non-Small Cell Lung Cancer Who Received Chemoradiotherapy. Int. J. Clin. Exp. Med..

[627] Saldi S., Perrucci E., Fulcheri C.P.L., Mariucci C., Chierchini S., Ingrosso G., Falcinelli L., Podlesko A.M., Merluzzi M., Bini V. (2020). Zinc-L-Carnosine Prevented Dysphagia in Breast Cancer Patients Undergoing Adjuvant Radiotherapy: Results of a Phase III Randomized Trial. Breast J..

[628] Nakagaki M., Kennedy G.A., Gavin N.C., Butler J., Clavarino A., Whitfield K. (2023). A Randomised Trial of Topical Polaprezinc to Prevent Oral Mucositis in Patients Undergoing Haematopoietic Stem Cell Transplantation (ToPaZ Study). Support. Care Cancer.

[629] Lin L.-C., Que J., Lin L.-K., Lin F.-C. (2006). Zinc Supplementation to Improve Mucositis and Dermatitis in Patients After Radiotherapy for Head-and-Neck Cancers: A Double-Blind, Randomized Study. Int. J. Radiat. Oncol. Biol. Phys..

[630] Chaitanya N., Badam R., Aryasri A.S., Pallarla S., Garlpati K., Akhila M., Soni P., Gali S., Inamdar P., Parinita B. (2020). Efficacy of Improvised Topical Zinc (1%) Ora-Base on Oral Mucositis During Cancer Chemo-Radiation-A Randomized Study. J. Nutr. Sci. Vitaminol..

[631] Ehudin M.A., Golla U., Trivedi D., Potlakayala S.D., Rudrabhatla S.V., Desai D., Dovat S., Claxton D., Sharma A. (2022). Therapeutic Benefits of Selenium in Hematological Malignancies. Int. J. Mol. Sci..

[632] Tayal S., Kaur N., Kaur T., Chadha V.D. (2025). Zinc as an Adjunct in Radiation-Based Therapies: Evidences of Radioprotection and Mechanistic Insights. Nutr. Health.

[633] Prasad A.S. (2014). Zinc Is an Antioxidant and Anti-Inflammatory Agent: Its Role in Human Health. Front. Nutr..

[634] Jarosz M., Olbert M., Wyszogrodzka G., Młyniec K., Librowski T. (2017). Antioxidant and Anti-Inflammatory Effects of Zinc. Zinc-Dependent NF-κB Signaling. Inflammopharmacology.

[635] Saadat I., Shakibaie M., Jomehzadeh A., Salimi A., Rahimi H.-R., Torabizadeh S.A. (2023). Radioprotective Effect of Zinc Nanoparticles on Ionizing Radiation-Induced Nephrotoxicity in Mice. Pharmacology.

[636] Cai L., Cherian M.G. (2003). Zinc-Metallothionein Protects from DNA Damage Induced by Radiation Better than Glutathione and Copper- or Cadmium-Metallothioneins. Toxicol. Lett..

[637] Marreiro D.d.N., Cruz K.J.C., Morais J.B.S., Beserra J.B., Severo J.S., de Oliveira A.R.S. (2017). Zinc and Oxidative Stress: Current Mechanisms. Antioxidants.

[638] Zhang Y., Wei X., Xu Y., Xia W., Zheng C., Zhang H., Chen W., Xu K., Huang Q. (2025). Zinc Sulfate Gel Reshapes the Wound Microenvironment to Promote Full-Thickness Wound Healing in Mice. Regen. Ther..

[639] Ertekin M.V., Tekin S.B., Erdogan F., Karslioglu I., Gepdiremen A., Sezen O., Balci E., Gündogdu C. (2004). The Effect of Zinc Sulphate in the Prevention of Radiation-Induced Dermatitis. J. Radiat. Res..

[640] Dhawan D., Singh Baweja M., Dani V. (2007). Zinc Sulphate Following the Administration of Iodine-131 on the Regulation of Thyroid Function, in Rats. Hell. J. Nucl. Med..

[641] Doi H., Kamikonya N., Takada Y., Fujiwara M., Tsuboi K., Inoue H., Tanooka M., Nakamura T., Shikata T., Tsujimura T. (2011). Efficacy of Polaprezinc for Acute Radiation Proctitis in a Rat Model. Int. J. Radiat. Oncol. Biol. Phys..

[642] Taysi S., Okumus S., Akyuz M., Uzun N., Aksoy A., Demir E., Orkmez M., Tarakcioglu M., Adli M. (2012). Zinc Administration Modulates Radiation-Induced Oxidative Injury in Lens of Rat. Pharmacogn. Mag..

[643] Kandaz M., Ertekin M.V., Karslıoğlu İ., Erdoğan F., Sezen O., Gepdiremen A., Gündoğdu C. (2017). Zinc Sulfate and/or Growth Hormone Administration for the Prevention of Radiation-Induced Dermatitis: A Placebo-Controlled Rat Model Study. Biol. Trace Elem. Res..

[644] Sharma P., Singla N., Dhawan D.K. (2017). Evidence of Zinc in Affording Protection Against X-Ray-Induced Brain Injury in Rats. Biol. Trace Elem. Res..

[645] Askar M.A., Guida M.S., AbuNour S.M., Ragab E.A., Ali E.N., Abdel-Magied N., Mansour N.A., Elmasry S.A. (2022). Nanoparticles for Active Combination Radio Mitigating Agents of Zinc Coumarate and Zinc Caffeinate in a Rat Model. Environ. Sci. Pollut. Res..

[646] Ertekin M.V., Karslioğlu I., Erdem F., Sezen O., Gepdiremen A., Serifoğlu K. (2004). Zinc Sulfate in the Prevention of Total-Body Irradiation-Induced Early Hematopoietic Toxicity: A Controlled Study in a Rat Model. Biol. Trace Elem. Res..

[647] Odawara S., Doi H., Shikata T., Kitajima K., Suzuki H., Niwa Y., Kosaka K., Tarutani K., Tsujimura T., Kamikonya N. (2016). Polaprezinc Protects Normal Intestinal Epithelium Against Exposure to Ionizing Radiation in Mice. Mol. Clin. Oncol..

[648] Abdel-Magied N., Shedid S.M. (2020). Impact of Zinc Oxide Nanoparticles on Thioredoxin-Interacting Protein and Asymmetric Dimethylarginine as Biochemical Indicators of Cardiovascular Disorders in Gamma-Irradiated Rats. Environ. Toxicol..

[649] Abd Elmonem H.A., Mahmoud A.H., Abbas M.M. (2021). Ameliorative Effect of Zinc Oxide Nanoparticles and Vitamin E on Some Biochemical and Histological Changes in Irradiated Albino Rats. Egypt. J. Radiat. Sci. Appl..

[650] Ceballos-Gutiérrez A., Rodríguez-Hernández A., Álvarez-Valadez M.D.R., Limón-Miranda S., Andrade F., Figueroa-Gutiérrez A., Díaz-Reval I., Apolinar-Iribe A., Castro-Sánchez L., Alamilla J. (2021). ZnO Nanoparticles Induce Dyslipidemia and Atherosclerotic Lesions Leading to Changes in Vascular Contractility and Cannabinoid Receptors Expression as Well as Increased Blood Pressure. Nanomaterials.

[651] Ishihama H., Sayo S., Yokoyama T., Ueno M., Ebihara N., Doi Y., Asano K., Kawamata H., Imai H., Ueki K. (2013). Preventive and Therapeutic Effects of Polaprezinc Suspension on Oral Mucosal Injury. Ann. Oncol..

[652] Agare G.I., Chidike Ezeorba T.P., Michael D.C., Agbamu E., Aghoja O.C., Alalor C.A. (2025). Zinc Supplementation for Mitigating Oral Mucositis in Head and Neck Cancer Patients Undergoing Radiotherapy and Chemoradiotherapy—A Systematic Review. Clin. Nutr. ESPEN.

[653] Hayashi H., Kobayashi R., Suzuki A., Ishihara M., Nakamura N., Kitagawa J., Kanemura N., Kasahara S., Kitaichi K., Hara T. (2014). Polaprezinc Prevents Oral Mucositis in Patients Treated with High-Dose Chemotherapy Followed by Hematopoietic Stem Cell Transplantation. Anticancer Res..

[654] Yamaguchi S. (2019). Clinical Outcome of Sodium Alginate Therapy in Radiation-Induced Pharyngeal Mucositis: Experience of a Single Japanese Institution. Appl. Cancer Res..

[655] Yu J., Huang W., Liu T., Defnet A.E., Zalesak-Kravec S., Farese A.M., MacVittie T.J., Kane M.A. (2021). Effect of Radiation on the Essential Nutrient Homeostasis and Signaling of Retinoids in a Non-Human Primate Model with Minimal Bone Marrow Sparing. Health Phys..

[656] Levenson S.M., Gruber C.A., Rettura G., Gruber D.K., Demetriou A.A., Seifter E. (1984). Supplemental Vitamin A Prevents the Acute Radiation-Induced Defect in Wound Healing. Ann. Surg..

[657] Beyzadeoğlu M., Balkan M., Demiriz M., Tibet H., Dirican B., Oner K., Pak Y. (1997). Protective Effect of Vitamin A on Acute Radiation Injury in the Small Intestine. Radiat. Med..

[658] Okoshi K., Kubo H., Nagayama S., Tabata C., Kadokawa Y., Hisamori S., Yonenaga Y., Fujimoto A., Mori A., Onodera H. (2008). All-Trans-Retinoic Acid Attenuates Radiation-Induced Intestinal Fibrosis in Mice. J. Surg. Res..

[659] Tabata C., Kadokawa Y., Tabata R., Takahashi M., Okoshi K., Sakai Y., Mishima M., Kubo H. (2006). All-Trans-Retinoic Acid Prevents Radiation- or Bleomycin-Induced Pulmonary Fibrosis. Am. J. Respir. Crit. Care Med..

[660] Cohen G., Elad S., Or R., Galili D., Garfunkel A. (1997). The Use of Tretinoin as Oral Mucositis Prophylaxis in Bone Marrow Transplantation Patients: A Preliminary Study. Oral Dis..

[661] Gebicki J.M., Nauser T., Domazou A., Steinmann D., Bounds P.L., Koppenol W.H. (2010). Reduction of Protein Radicals by GSH and Ascorbate: Potential Biological Significance. Amino Acids.

[662] Sato T., Kinoshita M., Yamamoto T., Ito M., Nishida T., Takeuchi M., Saitoh D., Seki S., Mukai Y. (2015). Treatment of Irradiated Mice with High-Dose Ascorbic Acid Reduced Lethality. PLoS ONE.

[663] Ito Y., Kinoshita M., Yamamoto T., Sato T., Obara T., Saitoh D., Seki S., Takahashi Y. (2013). A Combination of Pre- and Post-Exposure Ascorbic Acid Rescues Mice from Radiation-Induced Lethal Gastrointestinal Damage. Int. J. Mol. Sci..

[664] Jagetia G.C., Rajanikant G.K., Baliga M.S., Rao K.V.N.M., Kumar P. (2004). Augmentation of Wound Healing by Ascorbic Acid Treatment in Mice Exposed to Gamma-Radiation. Int. J. Radiat. Biol..

[665] Schoenfeld J.D., Alexander M.S., Waldron T.J., Sibenaller Z.A., Spitz D.R., Buettner G.R., Allen B.G., Cullen J.J. (2019). Pharmacological Ascorbate as a Means of Sensitizing Cancer Cells to Radio-Chemotherapy While Protecting Normal Tissue. Semin. Radiat. Oncol..

[666] Rawal M., Schroeder S.R., Wagner B.A., Cushing C.M., Welsh J.L., Button A.M., Du J., Sibenaller Z.A., Buettner G.R., Cullen J.J. (2013). Manganoporphyrins Increase Ascorbate-Induced Cytotoxicity by Enhancing H_2_O_2_ Generation. Cancer Res..

[667] Evans M.K., Tovmasyan A., Batinic-Haberle I., Devi G.R. (2014). Mn Porphyrin in Combination with Ascorbate Acts as a Pro-Oxidant and Mediates Caspase-Independent Cancer Cell Death. Free Radic. Biol. Med..

[668] Alexander M.S., Wilkes J.G., Schroeder S.R., Buettner G.R., Wagner B.A., Du J., Gibson-Corley K., O’Leary B.R., Spitz D.R., Buatti J.M. (2018). Pharmacologic Ascorbate Reduces Radiation-Induced Normal Tissue Toxicity and Enhances Tumor Radiosensitization in Pancreatic Cancer. Cancer Res..

[669] Welsh J.L., Wagner B.A., van’t Erve T.J., Zehr P.S., Berg D.J., Halfdanarson T.R., Yee N.S., Bodeker K.L., Du J., Roberts L.J. (2013). Pharmacological Ascorbate with Gemcitabine for the Control of Metastatic and Node-Positive Pancreatic Cancer (PACMAN): Results from a Phase I Clinical Trial. Cancer Chemother. Pharmacol..

[670] Rosário P.W., Batista K.C.S., Calsolari M.R. (2016). Radioiodine-Induced Oxidative Stress in Patients with Differentiated Thyroid Carcinoma and Effect of Supplementation with Vitamins C and E and Selenium (Antioxidants). Arch. Endocrinol. Metab..

[671] Tao S.M., Zhou F., Joseph Schoepf U., Fischer A.M., Giovagnoli D., Lin Z.X., Zhou C.S., Lu G.M., Zhang L.J. (2019). The Effect of Prophylactic Oral Vitamin C Use on DNA Double-Strand Breaks After Abdominal Contrast-Enhanced CT: A Preliminary Study. Eur. J. Radiol..

[672] Vollbracht C., Schneider B., Leendert V., Weiss G., Auerbach L., Beuth J. (2011). Intravenous Vitamin C Administration Improves Quality of Life in Breast Cancer Patients During Chemo-/Radiotherapy and Aftercare: Results of a Retrospective, Multicentre, Epidemiological Cohort Study in Germany. In Vivo.

[673] Chaitanya N.C., Muthukrishnan A., Rao K.P., Priyanka D.R., Ujwala P., Abhijeeth H., Kovur A., Kumar A.N. (2018). Oral Mucositis Severity Assessment by Supplementation of High Dose Ascorbic Acid During Chemo and/or Radiotherapy of Oro-Pharyngeal Cancers—A Pilot Project. Indian J. Pharm. Educ. Res..

[674] Abdel-Latif M.M.M., Babar M., Kelleher D., Reynolds J.V. (2019). A Pilot Study of the Impact of Vitamin C Supplementation with Neoadjuvant Chemoradiation on Regulators of Inflammation and Carcinogenesis in Esophageal Cancer Patients. J. Cancer Res. Ther..

[675] Lawenda B.D., Kelly K.M., Ladas E.J., Sagar S.M., Vickers A., Blumberg J.B. (2008). Should Supplemental Antioxidant Administration Be Avoided During Chemotherapy and Radiation Therapy?. J. Natl. Cancer Inst..

[676] Khazaei S., Nilsson L., Adrian G., Tryggvadottir H., Konradsson E., Borgquist S., Isaksson K., Ceberg C., Jernström H. (2022). Impact of Combining Vitamin C with Radiation Therapy in Human Breast Cancer: Does It Matter?. Oncotarget.

[677] Li W., Wang X., Dong Y., Huo Q., Yue T., Wu X., Lu L., Zhang J., Zhao Y., Dong H. (2023). Nicotinamide Riboside Intervention Alleviates Hematopoietic System Injury of Ionizing Radiation-induced Premature Aging Mice. Aging Cell.

[678] Obrador E., Salvador-Palmer R., Pellicer B., López-Blanch R., Sirerol J.A., Villaescusa J.I., Montoro A., Dellinger R.W., Estrela J.M. (2023). Combination of Natural Polyphenols with a Precursor of NAD+ and a TLR2/6 Ligand Lipopeptide Protects Mice Against Lethal γ Radiation. J. Adv. Res..

[679] Zhou Q., Liu L., Lin X., Shen B., Huang W., Zhang Y., Li Y., Yang Y., Liu H., Zhang W. (2025). Nicotinamide Riboside Attenuates Radiation-Induced Intestinal Injury by Suppressing Gasdermin E-Mediated Pyroptosis in Intestinal Epithelial Cells. J. Transl. Med..

[680] Zhao X., Ji K., Zhang M., Huang H., Wang F., Liu Y., Liu Q. (2023). NMN Alleviates Radiation-Induced Intestinal Fibrosis by Modulating Gut Microbiota. Int. J. Radiat. Biol..

[681] Singh V.K., Kulkarni S., Fatanmi O.O., Wise S.Y., Newman V.L., Romaine P.L.P., Hendrickson H., Gulani J., Ghosh S.P., Kumar K.S. (2016). Radioprotective Efficacy of Gamma-Tocotrienol in Nonhuman Primates. Radiat. Res..

[682] Garg S., Sadhukhan R., Banerjee S., Savenka A.V., Basnakian A.G., McHargue V., Wang J., Pawar S.A., Ghosh S.P., Ware J. (2019). Gamma-Tocotrienol Protects the Intestine from Radiation Potentially by Accelerating Mesenchymal Immune Cell Recovery. Antioxidants.

[683] Garg T.K., Garg S., Miousse I.R., Wise S.Y., Carpenter A.D., Fatanmi O.O., van Rhee F., Singh V.K., Hauer-Jensen M. (2022). Gamma-Tocotrienol Modulates Total-Body Irradiation-Induced Hematopoietic Injury in a Nonhuman Primate Model. Int. J. Mol. Sci..

[684] Roy R.M., Petrella M., Shateri H. (1988). Effects of Administering Tocopherol After Irradiation on Survival and Proliferation of Murine Lymphocytes. Pharmacol. Ther..

[685] Sarma L., Kesavan P.C. (1993). Protective Effects of Vitamins C and E Against Gamma-Ray-Induced Chromosomal Damage in Mouse. Int. J. Radiat. Biol..

[686] Bese N.S., Munzuroglu F., Uslu B., Arbak S., Yesiladali G., Sut N., Altug T., Ober A. (2007). Vitamin E Protects Against the Development of Radiation-Induced Pulmonary Fibrosis in Rats. Clin. Oncol..

[687] Li X.H., Fu D., Latif N.H., Mullaney C.P., Ney P.H., Mog S.R., Whitnall M.H., Srinivasan V., Xiao M. (2010). Delta-Tocotrienol Protects Mouse and Human Hematopoietic Progenitors from Gamma-Irradiation Through Extracellular Signal-Regulated Kinase/Mammalian Target of Rapamycin Signaling. Haematologica.

[688] Satyamitra M.M., Kulkarni S., Ghosh S.P., Mullaney C.P., Condliffe D., Srinivasan V. (2011). Hematopoietic Recovery and Amelioration of Radiation-Induced Lethality by the Vitamin E Isoform δ-Tocotrienol. Radiat. Res..

[689] Satyamitra M., Ney P., Graves J., Mullaney C., Srinivasan V. (2012). Mechanism of Radioprotection by δ-Tocotrienol: Pharmacokinetics, Pharmacodynamics and Modulation of Signalling Pathways. Br. J. Radiol..

[690] Lee S., Kalidindi T.M., Lou H., Gangangari K., Punzalan B., Bitton A., Lee C.J., Vargas H.A., Park S., Bodei L. (2021). γ-Tocotrienol–Loaded Liposomes for Radioprotection from Hematopoietic Side Effects Caused by Radiotherapeutic Drugs. J. Nucl. Med..

[691] Nemec-Bakk A.S., Sridharan V., Landes R.D., Singh P., Cao M., Seawright J.W., Liu X., Zheng G., Dominic P., Pathak R. (2021). Mitigation of Late Cardiovascular Effects of Oxygen Ion Radiation by γ-Tocotrienol in a Mouse Model. Life Sci. Space Res..

[692] Wiegman E.M., van Gameren M.M., Kampinga H.H., Szabó B.G., Coppes R.P. (2004). Post-Irradiation Dietary Vitamin E Does Not Affect the Development of Radiation-Induced Lung Damage in Rats. Radiother. Oncol..

[693] Redlich C.A., Rockwell S., Chung J.S., Sikora A.G., Kelley M., Mayne S.T. (1998). Vitamin A Inhibits Radiation-Induced Pneumonitis in Rats. J. Nutr..

[694] Nukala U., Thakkar S., Krager K.J., Breen P.J., Compadre C.M., Aykin-Burns N. (2018). Antioxidant Tocols as Radiation Countermeasures (Challenges to Be Addressed to Use Tocols as Radiation Countermeasures in Humans). Antioxidants.

[695] Ahsan H., Ahad A., Iqbal J., Siddiqui W.A. (2014). Pharmacological Potential of Tocotrienols: A Review. Nutr. Metab..

[696] Berbée M., Fu Q., Boerma M., Wang J., Kumar K.S., Hauer-Jensen M. (2009). Gamma-Tocotrienol Ameliorates Intestinal Radiation Injury and Reduces Vascular Oxidative Stress After Total-Body Irradiation by an HMG-CoA Reductase-Dependent Mechanism. Radiat. Res..

[697] Suman S., Datta K., Chakraborty K., Kulkarni S.S., Doiron K., Fornace A.J., Sree Kumar K., Hauer-Jensen M., Ghosh S.P. (2013). Gamma Tocotrienol, a Potent Radioprotector, Preferentially Upregulates Expression of Anti-Apoptotic Genes to Promote Intestinal Cell Survival. Food Chem. Toxicol..

[698] Berbée M., Fu Q., Boerma M., Sree Kumar K., Loose D.S., Hauer-Jensen M. (2012). Mechanisms Underlying the Radioprotective Properties of γ-Tocotrienol: Comparative Gene Expression Profiling in Tocol-Treated Endothelial Cells. Genes Nutr..

[699] Pathak R., Bachri A., Ghosh S.P., Koturbash I., Boerma M., Binz R.K., Sawyer J.R., Hauer-Jensen M. (2016). The Vitamin E Analog Gamma-Tocotrienol (GT3) Suppresses Radiation-Induced Cytogenetic Damage. Pharm. Res..

[700] Tasanarong A., Kongkham S., Duangchana S., Thitiarchakul S., Eiam-Ong S. (2011). Vitamin E Ameliorates Renal Fibrosis by Inhibition of TGF-Beta/Smad2/3 Signaling Pathway in UUO Mice. J. Med. Assoc. Thail..

[701] Li X.H., Ghosh S.P., Ha C.T., Fu D., Elliott T.B., Bolduc D.L., Villa V., Whitnall M.H., Landauer M.R., Xiao M. (2013). Delta-Tocotrienol Protects Mice from Radiation-Induced Gastrointestinal Injury. Radiat. Res..

[702] Singh V.K., Wise S.Y., Scott J.R., Romaine P.L.P., Newman V.L., Fatanmi O.O. (2014). Radioprotective Efficacy of Delta-Tocotrienol, a Vitamin E Isoform, Is Mediated Through Granulocyte Colony-Stimulating Factor. Life Sci..

[703] Kulkarni S.S., Cary L.H., Gambles K., Hauer-Jensen M., Kumar K.S., Ghosh S.P. (2012). Gamma-Tocotrienol, a Radiation Prophylaxis Agent, Induces High Levels of Granulocyte Colony-Stimulating Factor. Int. Immunopharmacol..

[704] Singh V.K., Hauer-Jensen M. (2016). γ-Tocotrienol as a Promising Countermeasure for Acute Radiation Syndrome: Current Status. Int. J. Mol. Sci..

[705] Ferreira P.R., Fleck J.F., Diehl A., Barletta D., Braga-Filho A., Barletta A., Ilha L. (2004). Protective Effect of Alpha-Tocopherol in Head and Neck Cancer Radiation-Induced Mucositis: A Double-Blind Randomized Trial. Head Neck.

[706] Kumar D.D., Aggarwal D.A.K., Shukla D.D.K., Thimothy G., Rani S. (2017). To Study and Evaluation of Role of Gamma and Delta Tocotrienol in Radiation Induced Fibrosis. Pharma Innov..

[707] Solduzian M., Hadji Babaei M., Goodarzi N., Honarmand H., Shabanir N., Labbani Motlagh Z., Taghvaye-Masoumi H., Jahangard-Rafsanjani Z., Kamran Zadeh H., Sadeghi K. (2021). Effects of Topical Vitamin E Mouthwash in Preventing Oral Mucositis in Allogenic Hematopoietic Stem Cell Transplant Patients: A Double-Blind, Placebo-Controlled Trial. Iran. J. Blood Cancer.

[708] Fallahi B., Beiki D., Abedi S.M., Saghari M., Fard-Esfahani A., Akhzari F., Mokarami B., Eftekhari M. (2013). Does Vitamin E Protect Salivary Glands from I-131 Radiation Damage in Patients with Thyroid Cancer?. Nucl. Med. Commun..

[709] Bairati I., Meyer F., Gélinas M., Fortin A., Nabid A., Brochet F., Mercier J.-P., Têtu B., Harel F., Abdous B. (2005). Randomized Trial of Antioxidant Vitamins to Prevent Acute Adverse Effects of Radiation Therapy in Head and Neck Cancer Patients. J. Clin. Oncol..

[710] Bjelakovic G., Nikolova D., Gluud L.L., Simonetti R.G., Gluud C. (2012). Antioxidant Supplements for Prevention of Mortality in Healthy Participants and Patients with Various Diseases. Cochrane Database Syst. Rev..

[711] Queiroz Schmidt F.M., Serna González C.V., Mattar R.C., Lopes L.B., Santos M.F., Santos V.L.C.d.G. (2022). Topical Application of a Cream Containing Nanoparticles with Vitamin E for Radiodermatitis Prevention in Women with Breast Cancer: A Randomized, Triple-Blind, Controlled Pilot Trial. Eur. J. Oncol. Nurs..

[712] Futran N.D., Trotti A., Gwede C. (1997). Pentoxifylline in the Treatment of Radiation-Related Soft Tissue Injury: Preliminary Observations. Laryngoscope.

[713] Chua D.T., Lo C., Yuen J., Foo Y.C. (2001). A Pilot Study of Pentoxifylline in the Treatment of Radiation-Induced Trismus. Am. J. Clin. Oncol..

[714] Ozturk B., Egehan I., Atavci S., Kitapci M. (2004). Pentoxifylline in Prevention of Radiation-Induced Lung Toxicity in Patients with Breast and Lung Cancer: A Double-Blind Randomized Trial. Int. J. Radiat. Oncol. Biol. Phys..

[715] Okunieff P., Augustine E., Hicks J.E., Cornelison T.L., Altemus R.M., Naydich B.G., Ding I., Huser A.K., Abraham E.H., Smith J.J. (2004). Pentoxifylline in the Treatment of Radiation-Induced Fibrosis. J. Clin. Oncol..

[716] Delanian S., Porcher R., Balla-Mekias S., Lefaix J.-L. (2003). Randomized, Placebo-Controlled Trial of Combined Pentoxifylline and Tocopherol for Regression of Superficial Radiation-Induced Fibrosis. J. Clin. Oncol..

[717] Delanian S., Porcher R., Rudant J., Lefaix J.-L. (2005). Kinetics of Response to Long-Term Treatment Combining Pentoxifylline and Tocopherol in Patients with Superficial Radiation-Induced Fibrosis. J. Clin. Oncol..

[718] Haddad P., Kalaghchi B., Amouzegar-Hashemi F. (2005). Pentoxifylline and Vitamin E Combination for Superficial Radiation-Induced Fibrosis: A Phase II Clinical Trial. Radiother. Oncol..

[719] Magnusson M., Höglund P., Johansson K., Jönsson C., Killander F., Malmström P., Weddig A., Kjellén E. (2009). Pentoxifylline and Vitamin E Treatment for Prevention of Radiation-Induced Side-Effects in Women with Breast Cancer: A Phase Two, Double-Blind, Placebo-Controlled Randomised Clinical Trial (Ptx-5). Eur. J. Cancer.

[720] Jacobson G., Bhatia S., Smith B.J., Button A.M., Bodeker K., Buatti J. (2013). Randomized Trial of Pentoxifylline and Vitamin E vs Standard Follow-up After Breast Irradiation to Prevent Breast Fibrosis, Evaluated by Tissue Compliance Meter. Int. J. Radiat. Oncol. Biol. Phys..

[721] Misirlioglu C.H., Demirkasimoglu T., Kucukplakci B., Sanri E., Altundag K. (2007). Pentoxifylline and Alpha-Tocopherol in Prevention of Radiation-Induced Lung Toxicity in Patients with Lung Cancer. Med. Oncol..

[722] Willett A., Silverman C., Woo S., Gaskins J.T., Dunlap N. (2025). The Effect of Pentoxifylline and Vitamin E in Preventing Grade 3 Radiation Pneumonitis: A Single-Arm, Phase II Prospective Study. Appl. Radiat. Oncol..

[723] Gothard L., Cornes P., Earl J., Hall E., MacLaren J., Mortimer P., Peacock J., Peckitt C., Woods M., Yarnold J. (2004). Double-Blind Placebo-Controlled Randomised Trial of Vitamin E and Pentoxifylline in Patients with Chronic Arm Lymphoedema and Fibrosis After Surgery and Radiotherapy for Breast Cancer. Radiother. Oncol..

[724] Gothard L., Cornes P., Brooker S., Earl J., Glees J., Hall E., Peckitt C., Tait D., Yarnold J. (2005). Phase II Study of Vitamin E and Pentoxifylline in Patients with Late Side Effects of Pelvic Radiotherapy. Radiother. Oncol..

[725] Andreyev H.J.N., Matthews J., Adams C., Gothard L., Lucy C., Tovey H., Boyle S., Anbalagan S., Musallam A., Yarnold J. (2022). Randomised Single Centre Double-Blind Placebo Controlled Phase II Trial of Tocovid SupraBio in Combination with Pentoxifylline in Patients Suffering Long-Term Gastrointestinal Adverse Effects of Radiotherapy for Pelvic Cancer: The PPALM Study. Radiother. Oncol..

[726] Delanian S., Chatel C., Porcher R., Depondt J., Lefaix J.-L. (2011). Complete Restoration of Refractory Mandibular Osteoradionecrosis by Prolonged Treatment with a Pentoxifylline-Tocopherol-Clodronate Combination (PENTOCLO): A Phase II Trial. Int. J. Radiat. Oncol. Biol. Phys..

[727] Robard L., Louis M.-Y., Blanchard D., Babin E., Delanian S. (2014). Medical Treatment of Osteoradionecrosis of the Mandible by PENTOCLO: Preliminary Results. Eur. Ann. Otorhinolaryngol. Head Neck Dis..

[728] Aggarwal K., Goutam M., Singh M., Kharat N., Singh V., Vyas S., Singh H.P. (2017). Prophylactic Use of Pentoxifylline and Tocopherol in Patients Undergoing Dental Extractions Following Radiotherapy for Head and Neck Cancer. Niger. J. Surg..

[729] Delanian S.E., Lenglet T., Maisonobe T., Resche-Rigon M., Pradat P.-F. (2020). Randomized, Placebo-Controlled Clinical Trial Combining Pentoxifylline-Tocopherol and Clodronate in the Treatment of Radiation-Induced Plexopathy. Int. J. Radiat. Oncol. Biol. Phys..

[730] Rübe C.E., Wilfert F., Uthe D., Schmid K.W., Knoop R., Willich N., Schuck A., Rübe C. (2002). Modulation of Radiation-Induced Tumour Necrosis Factor Alpha (TNF-Alpha) Expression in the Lung Tissue by Pentoxifylline. Radiother. Oncol..

[731] Lee J.-G., Shim S., Kim M.-J., Myung J.K., Jang W.-S., Bae C.-H., Lee S.-J., Kim K.M., Jin Y.-W., Lee S.-S. (2017). Pentoxifylline Regulates Plasminogen Activator Inhibitor-1 Expression and Protein Kinase A Phosphorylation in Radiation-Induced Lung Fibrosis. Biomed. Res. Int..

[732] Stelzer K.J., Koh W.J., Peterson L.M., Griffin T.W. (1996). Effect of High-Dose Pentoxifylline on Acute Radiation-Induced Lung Toxicity in a Rat Lung Perfusion Model. Int. J. Radiat. Oncol. Biol. Phys..

[733] Hepgül G., Tanrikulu S., Unalp H.R., Akguner T., Erbil Y., Olgaç V., Ademoğlu E. (2010). Preventive Effect of Pentoxifylline on Acute Radiation Damage via Antioxidant and Anti-Inflammatory Pathways. Dig. Dis. Sci..

[734] Patel V., McGurk M. (2017). Use of Pentoxifylline and Tocopherol in Radiation-Induced Fibrosis and Fibroatrophy. Br. J. Oral Maxillofac. Surg..

[735] Lefaix J.L., Delanian S., Vozenin M.C., Leplat J.J., Tricaud Y., Martin M. (1999). Striking Regression of Subcutaneous Fibrosis Induced by High Doses of Gamma Rays Using a Combination of Pentoxifylline and Alpha-Tocopherol: An Experimental Study. Int. J. Radiat. Oncol. Biol. Phys..

[736] Liu H., Xiong M., Xia Y.-F., Cui N.-J., Lu R.-B., Deng L., Lin Y.-H., Rong T.-H. (2009). Studies on Pentoxifylline and Tocopherol Combination for Radiation-Induced Heart Disease in Rats. Int. J. Radiat. Oncol. Biol. Phys..

[737] Berbée M., Fu Q., Garg S., Kulkarni S., Kumar K.S., Hauer-Jensen M. (2011). Pentoxifylline Enhances the Radioprotective Properties of γ-Tocotrienol: Differential Effects on the Hematopoietic, Gastrointestinal and Vascular Systems. Radiat. Res..

[738] Boerma M., Roberto K.A., Hauer-Jensen M. (2008). Prevention and Treatment of Functional and Structural Radiation Injury in the Rat Heart by Pentoxifylline and Alpha-Tocopherol. Int. J. Radiat. Oncol. Biol. Phys..

[739] Demircan V., Guzel C., Sarıbas G.S., Dinc S.C., Cetin S., Gulbahar O., Erpolat P., Elmas C., Bora H. (2024). Evaluation of Therapeutic Use of a Combination of Pentoxifylline and Vitamin E in Radiation-Induced Renal Fibrosis. Sci. Rep..

[740] Hayashi M., Pellecer M., Chung E., Sung E. (2015). The Efficacy of Pentoxifylline/Tocopherol Combination in the Treatment of Osteoradionecrosis. Spec. Care Dent..

[741] Delanian S., Lefaix J.-L., Maisonobe T., Salachas F., Pradat P.-F. (2008). Significant Clinical Improvement in Radiation-Induced Lumbosacral Polyradiculopathy by a Treatment Combining Pentoxifylline, Tocopherol, and Clodronate (Pentoclo). J. Neurol. Sci..

[742] Hille A., Schmidberger H., Christiansen H., Pradier O., Weiss E., Hess C.F. (2004). Effect of Pentoxifylline and Tocopherol on Chronic Radiation Proctitis/Enteritis. Strahlenther. Onkol..

[743] Averill-Bates D.A. (2023). The Antioxidant Glutathione. Vitam. Horm..

[744] Muranaka H., Akinsola R., Billet S., Pandol S.J., Hendifar A.E., Bhowmick N.A., Gong J. (2024). Glutamine Supplementation as an Anticancer Strategy: A Potential Therapeutic Alternative to the Convention. Cancers.

[745] Newsholme P. (2001). Why Is L-Glutamine Metabolism Important to Cells of the Immune System in Health, Postinjury, Surgery or Infection?. J. Nutr..

[746] Anderson P.M., Lalla R.V. (2020). Glutamine for Amelioration of Radiation and Chemotherapy Associated Mucositis During Cancer Therapy. Nutrients.

[747] Kozelsky T.F., Meyers G.E., Sloan J.A., Shanahan T.G., Dick S.J., Moore R.L., Engeler G.P., Frank A.R., McKone T.K., Urias R.E. (2003). Phase III Double-Blind Study of Glutamine versus Placebo for the Prevention of Acute Diarrhea in Patients Receiving Pelvic Radiation Therapy. J. Clin. Oncol..

[748] Rotovnik Kozjek N., Kompan L., Soeters P., Oblak I., Mlakar Mastnak D., Možina B., Zadnik V., Anderluh F., Velenik V. (2011). Oral Glutamine Supplementation During Preoperative Radiochemotherapy in Patients with Rectal Cancer: A Randomised Double Blinded, Placebo Controlled Pilot Study. Clin. Nutr..

[749] Vidal-Casariego A., Calleja-Fernández A., de Urbina-González J.J.O., Cano-Rodríguez I., Cordido F., Ballesteros-Pomar M.D. (2014). Efficacy of Glutamine in the Prevention of Acute Radiation Enteritis: A Randomized Controlled Trial. JPEN J. Parenter. Enter. Nutr..

[750] Cao D., Xu H., Xu M., Qian X., Yin Z., Ge W. (2017). Therapeutic Role of Glutamine in Management of Radiation Enteritis: A Meta-Analysis of 13 Randomized Controlled Trials. Oncotarget.

[751] Yuce Sari S., Yazici G., Yuce D., Karabulut E., Cengiz M., Ozyigit G. (2016). The Effect of Glutamine and Arginine-Enriched Nutritional Support on Quality of Life in Head and Neck Cancer Patients Treated with IMRT. Clin. Nutr. ESPEN.

[752] Hassanein F.E.A., Mikhail C., Elkot S., Abou-Bakr A. (2025). L-Arginine vs. L-Glutamine Oral Suspensions for Radiation-Induced Oral Mucositis: A Triple-Blind Randomized Trial. J. Cancer Res. Clin. Oncol..

[753] Imai T., Matsuura K., Asada Y., Sagai S., Katagiri K., Ishida E., Saito D., Sadayasu R., Wada H., Saijo S. (2014). Effect of HMB/Arg/Gln on the Prevention of Radiation Dermatitis in Head and Neck Cancer Patients Treated with Concurrent Chemoradiotherapy. Jpn. J. Clin. Oncol..

[754] Yokota T., Hamauchi S., Yoshida Y., Yurikusa T., Suzuki M., Yamashita A., Ogawa H., Onoe T., Mori K., Onitsuka T. (2018). A Phase II Study of HMB/Arg/Gln Against Oral Mucositis Induced by Chemoradiotherapy for Patients with Head and Neck Cancer. Support. Care Cancer.

[755] Kuroki K., Rikimaru F., Kunitake N., Toh S., Higaki Y., Masuda M. (2023). Efficacy of Beta-Hydroxy-Beta-Methylbutyrate, Arginine, and Glutamine for the Prevention of Mucositis Induced by Platinum-Based Chemoradiation in Head and Neck Cancer: A Phase II Study. Clin. Nutr. ESPEN.

[756] Huang E.Y., Leung S.W., Wang C.J., Chen H.C., Sun L.M., Fang F.M., Yeh S.A., Hsu H.C., Hsiung C.Y. (2000). Oral Glutamine to Alleviate Radiation-Induced Oral Mucositis: A Pilot Randomized Trial. Int. J. Radiat. Oncol. Biol. Phys..

[757] Cerchietti L.C.A., Navigante A.H., Lutteral M.A., Castro M.A., Kirchuk R., Bonomi M., Cabalar M.E., Roth B., Negretti G., Sheinker B. (2006). Double-Blinded, Placebo-Controlled Trial on Intravenous l-Alanyl-l-Glutamine in the Incidence of Oral Mucositis Following Chemoradiotherapy in Patients with Head-and-Neck Cancer. Int. J. Radiat. Oncol. Biol. Phys..

[758] Chattopadhyay S., Saha A., Azam M., Mukherjee A., Sur P.K. (2014). Role of Oral Glutamine in Alleviation and Prevention of Radiation-Induced Oral Mucositis: A Prospective Randomized Study. South Asian J. Cancer.

[759] Tsujimoto T., Yamamoto Y., Wasa M., Takenaka Y., Nakahara S., Takagi T., Tsugane M., Hayashi N., Maeda K., Inohara H. (2015). L-Glutamine Decreases the Severity of Mucositis Induced by Chemoradiotherapy in Patients with Locally Advanced Head and Neck Cancer: A Double-Blind, Randomized, Placebo-Controlled Trial. Oncol. Rep..

[760] Pachón Ibáñez J., Pereira Cunill J.L., Osorio Gómez G.F., Irles Rocamora J.A., Serrano Aguayo P., Quintana Ángel B., Fuentes Pradera J., Chaves Conde M., Ortiz Gordillo M.J., García Luna P.P. (2018). Prevention of Oral Mucositis Secondary to Antineoplastic Treatments in Head and Neck Cancer by Supplementation with Oral Glutamine. Nutr. Hosp..

[761] Harada K., Minami H., Ferdous T., Kato Y., Umeda H., Horinaga D., Uchida K., Park S.C., Hanazawa H., Takahashi S. (2019). The Elental® Elemental Diet for Chemoradiotherapy-induced Oral Mucositis: A Prospective Study in Patients with Oral Squamous Cell Carcinoma. Mol. Clin. Oncol..

[762] Lopez-Vaquero D., Gutierrez-Bayard L., Rodriguez-Ruiz J.-A., Saldaña-Valderas M., Infante-Cossio P. (2017). Double-Blind Randomized Study of Oral Glutamine on the Management of Radio/Chemotherapy-Induced Mucositis and Dermatitis in Head and Neck Cancer. Mol. Clin. Oncol..

[763] Huang C.-J., Huang M.-Y., Fang P.-T., Chen F., Wang Y.-T., Chen C.-H., Yuan S.-S., Huang C.-M., Luo K.-H., Chuang H.-Y. (2019). Randomized Double-Blind, Placebo-Controlled Trial Evaluating Oral Glutamine on Radiation-Induced Oral Mucositis and Dermatitis in Head and Neck Cancer Patients. Am. J. Clin. Nutr..

[764] Anderson P.M., Ramsay N.K., Shu X.O., Rydholm N., Rogosheske J., Nicklow R., Weisdorf D.J., Skubitz K.M. (1998). Effect of Low-Dose Oral Glutamine on Painful Stomatitis During Bone Marrow Transplantation. Bone Marrow Transplant..

[765] Schloerb P.R., Skikne B.S. (1999). Oral and Parenteral Glutamine in Bone Marrow Transplantation: A Randomized, Double-Blind Study. JPEN J. Parenter. Enter. Nutr..

[766] Blijlevens N.M.A., Donnelly J.P., Naber A.H.J., Schattenberg A.V.M.B., DePauw B.E. (2005). A Randomised, Double-Blinded, Placebo-Controlled, Pilot Study of Parenteral Glutamine for Allogeneic Stem Cell Transplant Patients. Support. Care Cancer.

[767] Pytlík R., Benes P., Patorková M., Chocenská E., Gregora E., Procházka B., Kozák T. (2002). Standardized Parenteral Alanyl-Glutamine Dipeptide Supplementation Is Not Beneficial in Autologous Transplant Patients: A Randomized, Double-Blind, Placebo Controlled Study. Bone Marrow Transplant..

[768] Aquino V.M., Harvey A.R., Garvin J.H., Godder K.T., Nieder M.L., Adams R.H., Jackson G.B., Sandler E.S. (2005). A Double-Blind Randomized Placebo-Controlled Study of Oral Glutamine in the Prevention of Mucositis in Children Undergoing Hematopoietic Stem Cell Transplantation: A Pediatric Blood and Marrow Transplant Consortium Study. Bone Marrow Transplant..

[769] Uderzo C., Rebora P., Marrocco E., Varotto S., Cichello F., Bonetti M., Maximova N., Zanon D., Fagioli F., Nesi F. (2011). Glutamine-Enriched Nutrition Does Not Reduce Mucosal Morbidity or Complications After Stem-Cell Transplantation for Childhood Malignancies: A Prospective Randomized Study. Transplantation.

[770] Yoshida S., Matsui M., Shirouzu Y., Fujita H., Yamana H., Shirouzu K. (1998). Effects of Glutamine Supplements and Radiochemotherapy on Systemic Immune and Gut Barrier Function in Patients with Advanced Esophageal Cancer. Ann. Surg..

[771] Chang S.-C., Lai Y.-C., Hung J.-C., Chang C.-Y. (2019). Oral Glutamine Supplements Reduce Concurrent Chemoradiotherapy-Induced Esophagitis in Patients with Advanced Non-Small Cell Lung Cancer. Medicine.

[772] Alshawa A., Cadena A.P., Stephen B., Reddy A., Mendoza T.R., McQuinn L., Lawhorn K., Zarifa A., Bernhardt A.M., Fessaheye S. (2021). Effects of Glutamine for Prevention of Radiation-Induced Esophagitis: A Double-Blind Placebo-Controlled Trial. Investig. New Drugs.

[773] Papanikolopoulou A., Syrigos N., Vini L., Papasavva M., Lazopoulos G., Kteniadakis S., Spandidos D.A., Charpidou A., Drakoulis N. (2022). Use of Oral Glutamine in Radiation-Induced Adverse Effects in Patients with Thoracic and Upper Aerodigestive Malignancies: Results of a Prospective Observational Study. Oncol. Lett..

[774] Rubio I., Suva L.J., Todorova V., Bhattacharyya S., Kaufmann Y., Maners A., Smith M., Klimberg V.S. (2013). Oral Glutamine Reduces Radiation Morbidity in Breast Conservation Surgery. J. Parenter. Enter. Nutr..

[775] Eda K., Uzer K., Murat T., Cenk U. (2016). The Effects of Enteral Glutamine on Radiotherapy Induced Dermatitis in Breast Cancer. Clin. Nutr..

[776] Peña Vivas J.d.C., Orduz Arena A.C., Alonso García A., Carrascal Gordillo C.F., Martínez Gutiérrez R., Rodríguez-Acosta Caballero C., Fernández Freije I., Paino Martínez A.B., Belloso Cuesta T., Juan Rijo G. (2024). Clinical, Functional, and Nutritional Efficacy of a Glutamine-Enriched Oligomeric Diet in Patients with Rectal Cancer. Nutr. Cancer.

[777] Salas-Salas B.G., Ferrera-Alayón L., Calleja-Fernández A., Chicas-Sett R., Nogués-Ramia E., Zafra-Martín J., Lloret M. (2024). Impact of a Glutamine-Enriched Peptide Formula on Gastrointestinal Toxicity and on the Interruption of Oncologic Treatment in Patients with Adenocarcinoma of the Rectum. Front. Nutr..

[778] Chambers M.S., Welsh D.V., Scrimger R.A., Zehn W., Epstein J.B., Troha J., Sonis S.T. (2006). RK-0202 for Radiation-Induced Oral Mucositis. J. Clin. Oncol..

[779] Sio T.T., Blanchard M.J., Novotny P.J., Patel S.H., Rwigema J.-C.M., Pederson L.D., McGee L.A., Gamez M.E., Seeger G.R., Martenson J.A. (2019). N-Acetylcysteine Rinse for Thick Secretion and Mucositis of Head and Neck Chemoradiotherapy (Alliance MC13C2): A Double-Blind Randomized Clinical Trial. Mayo Clin. Proc..

[780] Won H.-R., ho Lee G., Kim J.H., Lee S.H., Kwon S.Y., Baek S.-K., Ryu C.H., Lee S.J., Park I.-S., Shin S.-C. (2021). Effects of N-Acetylcysteine Inhalation Therapy on the Quality of Life of Patients with Head and Neck Cancer Who Are Receiving Radiation Therapy: A Prospective Non-Randomized Controlled Multi-Center Study. J. Cancer Res. Clin. Oncol..

[781] Yoshida S., Kaibara A., Ishibashi N., Shirouzu K. (2001). Glutamine Supplementation in Cancer Patients. Nutrition.

[782] Nan D., Yao W., Huang L., Liu R., Chen X., Xia W., Sheng H., Zhang H., Liang X., Lu Y. (2025). Glutamine and Cancer: Metabolism, Immune Microenvironment, and Therapeutic Targets. Cell Commun. Signal..

[783] Wang C.-C., Hwang T.-Z., Yang C.-C., Lien C.-F., Wang C.-C., Shih Y.-C., Yeh S.-A., Hsieh M.-C. (2022). Impact of Parenteral Glutamine Supplement on Oncologic Outcomes in Patients with Nasopharyngeal Cancer Treated with Concurrent Chemoradiotherapy. Nutrients.

[784] Chang H.-C., Huang W.-Y., Chen P.-H., Huang T.-W., Gautama M.S.N. (2024). Effectiveness of Glutamine for the Treatment of Radiodermatitis in Cancer Patients: A Meta-Analysis of Randomized Controlled Trials. Support. Care Cancer.

[785] Topkan E., Parlak C., Topuk S., Pehlivan B. (2012). Influence of Oral Glutamine Supplementation on Survival Outcomes of Patients Treated with Concurrent Chemoradiotherapy for Locally Advanced Non-Small Cell Lung Cancer. BMC Cancer.

[786] Alsubaie H.M., Alsini A.Y., Alsubaie K.M., Abu-Zaid A., Alzahrani F.R., Sayed S., Pathak A.K., Alqahtani K.H. (2021). Glutamine for Prevention and Alleviation of Radiation-Induced Oral Mucositis in Patients with Head and Neck Squamous Cell Cancer: Systematic Review and Meta-Analysis of Controlled Trials. Head Neck.

[787] Yarom N., Hovan A., Bossi P., Ariyawardana A., Jensen S.B., Gobbo M., Saca-Hazboun H., Kandwal A., Majorana A., Ottaviani G. (2019). Systematic Review of Natural and Miscellaneous Agents for the Management of Oral Mucositis in Cancer Patients and Clinical Practice Guidelines—Part 1: Vitamins, Minerals, and Nutritional Supplements. Support. Care Cancer.

[788] Tang G., Huang W., Zhang L., Wei Z. (2022). Role of Glutamine in the Management of Oral Mucositis in Patients with Cancer: A Meta-Analysis of Randomized Controlled Trials. Nutr. Cancer.

[789] Wang D., Duan J.-J., Guo Y.-F., Chen J.-J., Chen T.-Q., Wang J., Yu S.-C. (2024). Targeting the Glutamine-Arginine-Proline Metabolism Axis in Cancer. J. Enzym. Inhib. Med. Chem..

[790] Carretero J., Obrador E., Pellicer J.A., Pascual A., Estrela J.M. (2000). Mitochondrial Glutathione Depletion by Glutamine in Growing Tumor Cells. Free Radic. Biol. Med..

[791] Obrador E., Carretero J., Esteve J.M., Pellicer J.A., Pascual A., Petschen I., Estrela J.M. (2001). Glutamine Potentiates TNF-Alpha-Induced Tumor Cytotoxicity. Free Radic. Biol. Med..

[792] Benlloch M., Mena S., Ferrer P., Obrador E., Asensi M., Pellicer J.A., Carretero J., Ortega A., Estrela J.M. (2006). Bcl-2 and Mn-SOD Antisense Oligodeoxynucleotides and a Glutamine-Enriched Diet Facilitate Elimination of Highly Resistant B16 Melanoma Cells by Tumor Necrosis Factor-Alpha and Chemotherapy. J. Biol. Chem..

[793] Ishak Gabra M.B., Yang Y., Li H., Senapati P., Hanse E.A., Lowman X.H., Tran T.Q., Zhang L., Doan L.T., Xu X. (2020). Dietary Glutamine Supplementation Suppresses Epigenetically-Activated Oncogenic Pathways to Inhibit Melanoma Tumour Growth. Nat. Commun..

[794] Jia D., Koonce N.A., Griffin R.J., Jackson C., Corry P.M. (2010). Prevention and Mitigation of Acute Death of Mice After Abdominal Irradiation by the Antioxidant N-Acetyl-Cysteine (NAC). Radiat. Res..

[795] Wang Y., Zhang Z., Chen S., Zou Z., Tu X., Wang L. (2010). Protective effect of N-acetylcysteine on the intestinal barrier dysfunction After radiation injury in rats. Zhonghua Wei Chang Wai Ke Za Zhi.

[796] Li J., Meng Z., Zhang G., Xing Y., Feng L., Fan S., Fan F., Buren B., Liu Q. (2015). N-Acetylcysteine Relieves Oxidative Stress and Protects Hippocampus of Rat from Radiation-Induced Apoptosis by Inhibiting Caspase-3. Biomed. Pharmacother..

[797] Gao W., Liang J.-X., Ma C., Dong J.-Y., Yan Q. (2017). The Protective Effect of N-Acetylcysteine on Ionizing Radiation Induced Ovarian Failure and Loss of Ovarian Reserve in Female Mouse. BioMed Res. Int..

[798] Mercantepe F., Topcu A., Rakici S., Tumkaya L., Yilmaz A. (2019). The Effects of N-Acetylcysteine on Radiotherapy-Induced Small Intestinal Damage in Rats. Exp. Biol. Med..

[799] Barlaz Us S., Vezir O., Yildirim M., Bayrak G., Yalin S., Balli E., Yalin A.E., Çömelekoğlu Ü. (2020). Protective Effect of N-Acetyl Cysteine Against Radiotherapy-Induced Cardiac Damage. Int. J. Radiat. Biol..

[800] Motallebzadeh E., Suliman Maashi M., Mahmoud M.Z., Aliasgharzedeh A., Vakili Z., Talaei S.A., Mohseni M. (2022). Radioprotective Effects of N-Acetylcysteine on Rats’ Brainstem Following Megavoltage X-Irradiations. Appl. Radiat. Isot..

[801] Demirel C., Kilciksiz S., Evirgen-Ayhan S., Gurgul S., Erdal N. (2010). The Preventive Effect of N-Acetylcysteine on Radiation-Induced Dermatitis in a Rat Model. J. BUON.

[802] Demirel C., Kilciksiz S., Gurgul S., Erdal N., Yildiz A. (2011). N-Acetylcysteine Ameliorates γ-Radiation-Induced Deterioration of Bone Quality in the Rat Femur. J. Int. Med. Res..

[803] Brown S.L., Kolozsvary A., Liu J., Jenrow K.A., Ryu S., Kim J.H. (2010). Antioxidant Diet Supplementation Starting 24 Hours After Exposure Reduces Radiation Lethality. Radiat. Res..

[804] Demir E.O., Cakmak G.K., Bakkal H., Turkcu U.O., Kandemir N., Demir A.S., Tascılar O. (2011). N-Acetyl-Cysteine Improves Anastomotic Wound Healing After Radiotherapy in Rats. J. Investig. Surg..

[805] Tascilar O., Cakmak G., Emre A., Bakkal H., Kandemir N., Turkcu U., Demir E. (2014). N-Acetylcycsteine Attenuates the Deleterious Effects of Radiation Therapy on Inci-Sional Wound Healing in Rats. Hippokratia.

[806] Catto V., Stronati G., Porro B., Fiorelli S., Ricci V., Vavassori C., Russo E., Guerra F., Gasperetti A., Ribatti V. (2021). Cardiac Arrhythmia Catheter Ablation Procedures Guided by X-Ray Imaging: N-Acetylcysteine Protection Against Radiation-Induced Cellular Damage (CARAPACE Study): Study Design. J. Interv. Card. Electrophysiol..

[807] Sayin V.I., Ibrahim M.X., Larsson E., Nilsson J.A., Lindahl P., Bergo M.O. (2014). Antioxidants Accelerate Lung Cancer Progression in Mice. Sci. Transl. Med..

[808] Kalyanaraman B. (2022). NAC, NAC, Knockin’ on Heaven’s Door: Interpreting the Mechanism of Action of N-Acetylcysteine in Tumor and Immune Cells. Redox Biol..

[809] Obrador E., Salvador-Palmer R., López-Blanch R., Oriol-Caballo M., Moreno-Murciano P., Estrela J.M. (2022). N-Acetylcysteine Promotes Metastatic Spread of Melanoma in Mice. Cancers.

[810] Sharma G.P., Fish B.L., Frei A.C., Narayanan J., Gasperetti T., Scholler D., Pierce L., Szalewski N., Blue N., Medhora M. (2022). Pharmacologic ACE-Inhibition Mitigates Radiation-Induced Pneumonitis by Suppressing ACE-Expressing Lung Myeloid Cells. Int. J. Radiat. Oncol. Biol. Phys..

[811] Molteni A., Moulder J.E., Cohen E.F., Ward W.F., Fish B.L., Taylor J.M., Wolfe L.F., Brizio-Molteni L., Veno P. (2000). Control of Radiation-Induced Pneumopathy and Lung Fibrosis by Angiotensin-Converting Enzyme Inhibitors and an Angiotensin II Type 1 Receptor Blocker. Int. J. Radiat. Biol..

[812] Ghosh S.N., Zhang R., Fish B.L., Semenenko V.A., Li X.A., Moulder J.E., Jacobs E.R., Medhora M. (2009). Renin-Angiotensin System Suppression Mitigates Experimental Radiation Pneumonitis. Int. J. Radiat. Oncol. Biol. Phys..

[813] Medhora M., Gao F., Jacobs E.R., Moulder J.E. (2012). Radiation Damage to the Lung: Mitigation by Angiotensin-Converting Enzyme (ACE) Inhibitors. Respirology.

[814] Kma L., Gao F., Fish B.L., Moulder J.E., Jacobs E.R., Medhora M. (2012). Angiotensin Converting Enzyme Inhibitors Mitigate Collagen Synthesis Induced by a Single Dose of Radiation to the Whole Thorax. J. Radiat. Res..

[815] Gao F., Fish B.L., Moulder J.E., Jacobs E.R., Medhora M. (2013). Enalapril Mitigates Radiation-Induced Pneumonitis and Pulmonary Fibrosis If Started 35 Days After Whole-Thorax Irradiation. Radiat. Res..

[816] Moulder J.E., Cohen E.P., Medhora M., Fish B.L. (2022). Angiotensin Converting Enzyme (ACE) Inhibitors as Radiation Countermeasures for Long-Duration Space Flights. Life Sci. Space Res..

[817] Kanugula A.K., Kaur J., Batra J., Ankireddypalli A.R., Velagapudi R. (2023). Renin-Angiotensin System: Updated Understanding and Role in Physiological and Pathophysiological States. Cureus.

[818] Liao W.-C., Shokr H., Faivre-Finn C., Dempsey C., Williams K.J., Chen L.-C. (2025). The Effect of the Concurrent Use of Angiotensin-Converting Enzyme Inhibitors or Receptor Blockers on Toxicity and Outcomes in Patients Treated with Radiotherapy: A Systematic Review and Meta-Analysis. Pharmaceuticals.

[819] Cong C., Niu S., Jiang Y., Zhang X., Jing W., Zheng Y., Zhang X., Su G., Zhang Y., Sun M. (2023). Renin-Angiotensin System Inhibitors Mitigate Radiation Pneumonitis by Activating ACE2-Angiotensin-(1-7) Axis via NF-κB/MAPK Pathway. Sci. Rep..

[820] Sharma G.P., Frei A., Fish B., Gasperetti T., Veley D., Szalewski N., Nissen A., Himburg H.A. (2023). Biological Sex Differences in Renin Angiotensin System Enzymes ACE and ACE2 Regulate Normal Tissue Response to Radiation Injury. Front. Physiol..

[821] Hasan H.F., Elgazzar E.M., Mostafa D.M. (2020). Diminazene Aceturate Extenuate the Renal Deleterious Consequences of Angiotensin-II Induced by γ-Irradiation Through Boosting ACE2 Signaling Cascade. Life Sci..

[822] Gasperetti T., Prasad Sharma G., Frei A.C., Pierce L., Veley D., Szalewski N., Narayanan J., Fish B.L., Himburg H.A. (2022). Mitigation of Multi-Organ Radiation Injury with ACE2 Agonist Diminazene Aceturate. Radiat. Res..

[823] Wang H., Liao Z., Zhuang Y., Xu T., Nguyen Q.-N., Levy L.B., O’Reilly M., Gold K.A., Gomez D.R. (2013). Do Angiotensin-Converting Enzyme Inhibitors Reduce the Risk of Symptomatic Radiation Pneumonitis in Patients with Non-Small Cell Lung Cancer After Definitive Radiation Therapy? Analysis of a Single-Institution Database. Int. J. Radiat. Oncol. Biol. Phys..

[824] Harder E.M., Park H.S., Nath S.K., Mancini B.R., Decker R.H. (2015). Angiotensin-Converting Enzyme Inhibitors Decrease the Risk of Radiation Pneumonitis After Stereotactic Body Radiation Therapy. Pract. Radiat. Oncol..

[825] Bracci S., Valeriani M., Agolli L., De Sanctis V., Maurizi Enrici R., Osti M.F. (2016). Renin-Angiotensin System Inhibitors Might Help to Reduce the Development of Symptomatic Radiation Pneumonitis After Stereotactic Body Radiotherapy for Lung Cancer. Clin. Lung Cancer.

[826] Alite F., Balasubramanian N., Adams W., Surucu M., Mescioglu I., Harkenrider M.M. (2018). Decreased Risk of Radiation Pneumonitis with Coincident Concurrent Use of Angiotensin-Converting Enzyme Inhibitors in Patients Receiving Lung Stereotactic Body Radiation Therapy. Am. J. Clin. Oncol..

[827] Zheng Y., Cong C., Wang Z., Liu Y., Zhang M., Zhou H., Su C., Sun M. (2023). Decreased Risk of Radiation Pneumonitis with Concurrent Use of Renin-Angiotensin System Inhibitors in Thoracic Radiation Therapy of Lung Cancer. Front. Med..

[828] Carpentier A.F., Ferrari D., Bailon O., Ursu R., Banissi C., Dubessy A.-L., Belin C., Levy C. (2012). Steroid-Sparing Effects of Angiotensin-II Inhibitors in Glioblastoma Patients. Eur. J. Neurol..

[829] Januel E., Ursu R., Alkhafaji A., Marantidou A., Doridam J., Belin C., Levy-Piedbois C., Carpentier A.F. (2015). Impact of Renin-Angiotensin System Blockade on Clinical Outcome in Glioblastoma. Eur. J. Neurol..

[830] Kourilsky A., Bertrand G., Ursu R., Doridam J., Barlog C., Faillot T., Mandonnet E., Belin C., Levy C., Carpentier A.F. (2016). Impact of Angiotensin-II Receptor Blockers on Vasogenic Edema in Glioblastoma Patients. J. Neurol..

[831] Chowdhary M., Okwan-Duodu D., Switchenko J.M., Press R.H., Jhaveri J., Buchwald Z.S., Zhong J., Chapman B.V., Bindra R.S., Contessa J.N. (2018). Angiotensin Receptor Blockade: A Novel Approach for Symptomatic Radiation Necrosis After Stereotactic Radiosurgery. J. Neuro-Oncol..

[832] Wang L.W., Fu X.L., Clough R., Sibley G., Fan M., Bentel G.C., Marks L.B., Anscher M.S. (2000). Can Angiotensin-Converting Enzyme Inhibitors Protect Against Symptomatic Radiation Pneumonitis?. Radiat. Res..

[833] Kharofa J., Cohen E.P., Tomic R., Xiang Q., Gore E. (2012). Decreased Risk of Radiation Pneumonitis with Incidental Concurrent Use of Angiotensin-Converting Enzyme Inhibitors and Thoracic Radiation Therapy. Int. J. Radiat. Oncol. Biol. Phys..

[834] Maloney L.T., Latour E., Chen Y., Rice D., Grossblatt-Wait A., Nabavizadeh N., Thomas C.R., Young K.H., Walker J.M., Holland J. (2022). Angiotensin Receptor Blockade and Stereotactic Body Radiation Therapy for Early Stage Lung Cancer ARB & SBRT for Early Stage Lung Cancer. Cancer Biol. Ther..

[835] Wedlake L.J., Silia F., Benton B., Lalji A., Thomas K., Dearnaley D.P., Blake P., Tait D., Khoo V.S., Andreyev H.J.N. (2012). Evaluating the Efficacy of Statins and ACE-Inhibitors in Reducing Gastrointestinal Toxicity in Patients Receiving Radiotherapy for Pelvic Malignancies. Eur. J. Cancer.

[836] Alashkham A., Paterson C., Rauchhaus P., Nabi G. (2016). Can Angiotensin-Converting Enzyme Inhibitors Reduce the Incidence, Severity, and Duration of Radiation Proctitis?. Int. J. Radiat. Oncol. Biol. Phys..

[837] Kerns S.L., Amidon Morlang A., Lee S.M., Peterson D.R., Marples B., Zhang H., Bylund K., Rosenzweig D., Hall W., De Ruyck K. (2022). Use of Angiotensin Converting Enzyme Inhibitors Is Associated with Reduced Risk of Late Bladder Toxicity Following Radiotherapy for Prostate Cancer. Radiother. Oncol..

[838] Ward W.F., Molteni A., Ts’ao C.-h., Hinz J.M. (1990). Captopril Reduces Collagen and Mast Cell Accumulation in Irradiated Rat Lung. Int. J. Radiat. Oncol. Biol. Phys..

[839] Medhora M., Gao F., Wu Q., Molthen R.C., Jacobs E.R., Moulder J.E., Fish B.L. (2014). Model Development and Use of ACE Inhibitors for Preclinical Mitigation of Radiation-Induced Injury to Multiple Organs. Radiat. Res..

[840] Molthen R.C., Wu Q., Fish B.L., Moulder J.E., Jacobs E.R., Medhora M.M. (2012). Mitigation of Radiation Induced Pulmonary Vascular Injury by Delayed Treatment with Captopril. Respirology.

[841] van der Veen S.J., Ghobadi G., de Boer R.A., Faber H., Cannon M.V., Nagle P.W., Brandenburg S., Langendijk J.A., van Luijk P., Coppes R.P. (2015). ACE Inhibition Attenuates Radiation-Induced Cardiopulmonary Damage. Radiother. Oncol..

[842] Fish B.L., Gao F., Narayanan J., Bergom C., Jacobs E.R., Cohen E.P., Moulder J.E., Orschell C.M., Medhora M. (2016). Combined Hydration and Antibiotics with Lisinopril to Mitigate Acute and Delayed High-Dose Radiation Injuries to Multiple Organs. Health Phys..

[843] Jacobs E.R., Narayanan J., Fish B.L., Gao F., Harmann L.M., Bergom C., Gasperetti T., Strande J.L., Medhora M. (2019). Cardiac Remodeling and Reversible Pulmonary Hypertension During Pneumonitis in Rats After 13-Gy Partial-Body Irradiation with Minimal Bone Marrow Sparing: Effect of Lisinopril. Health Phys..

[844] Medhora M., Gao F., Gasperetti T., Narayanan J., Khan A.H., Jacobs E.R., Fish B.L. (2019). Delayed Effects of Acute Radiation Exposure (Deare) in Juvenile and Old Rats: Mitigation by Lisinopril. Health Phys..

[845] Small W., James J.L., Moore T.D., Fintel D.J., Lutz S.T., Movsas B., Suntharalingam M., Garces Y.I., Ivker R., Moulder J. (2018). Utility of the ACE Inhibitor Captopril in Mitigating Radiation-Associated Pulmonary Toxicity in Lung Cancer: Results From NRG Oncology RTOG 0123. Am. J. Clin. Oncol..

[846] Sio T.T., Atherton P.J., Pederson L.D., Zhen W.K., Mutter R.W., Garces Y.I., Ma D.J., Leenstra J.L., Rwigema J.-C.M., Dakhil S. (2019). Daily Lisinopril vs Placebo for Prevention of Chemoradiation-Induced Pulmonary Distress in Patients with Lung Cancer (Alliance MC1221): A Pilot Double-Blind Randomized Trial. Int. J. Radiat. Oncol. Biol. Phys..

[847] Cohen E.P., Bedi M., Irving A.A., Jacobs E., Tomic R., Klein J., Lawton C.A., Moulder J.E. (2012). Mitigation of Late Renal and Pulmonary Injury After Hematopoietic Stem Cell Transplantation. Int. J. Radiat. Oncol. Biol. Phys..

[848] Cohen E.P., Irving A.A., Drobyski W.R., Klein J.P., Passweg J., Talano J.-A.M., Juckett M.B., Moulder J.E. (2008). Captopril to Mitigate Chronic Renal Failure After Hematopoietic Stem Cell Transplantation: A Randomized Controlled Trial. Int. J. Radiat. Oncol. Biol. Phys..

[849] Moulder J.E., Fish B.L., Cohen E.P. (2007). Treatment of Radiation Nephropathy with ACE Inhibitors and AII Type-1 and Type-2 Receptor Antagonists. Curr. Pharm. Des..

[850] Ilhan H., Wang H., Gildehaus F.J., Wängler C., Herrler T., Todica A., Schlichtiger J., Cumming P., Bartenstein P., Hacker M. (2016). Nephroprotective Effects of Enalapril After [177Lu]-DOTATATE Therapy Using Serial Renal Scintigraphies in a Murine Model of Radiation-Induced Nephropathy. EJNMMI Res..

[851] Cohen E.P., Fish B.L., Sharma M., Li X.A., Moulder J.E. (2007). Role of the Angiotensin II Type-2 Receptor in Radiation Nephropathy. Transl. Res..

[852] Davis T.A., Landauer M.R., Mog S.R., Barshishat-Kupper M., Zins S.R., Amare M.F., Day R.M. (2010). Timing of Captopril Administration Determines Radiation Protection or Radiation Sensitization in a Murine Model of Total Body Irradiation. Exp. Hematol..

[853] Day R.M., Davis T.A., Barshishat-Kupper M., McCart E.A., Tipton A.J., Landauer M.R. (2013). Enhanced Hematopoietic Protection from Radiation by the Combination of Genistein and Captopril. Int. Immunopharmacol..

[854] McCart E.A., Lee Y.H., Jha J., Mungunsukh O., Rittase W.B., Summers T.A., Muir J., Day R.M. (2019). Delayed Captopril Administration Mitigates Hematopoietic Injury in a Murine Model of Total Body Irradiation. Sci. Rep..

[855] Rittase W.B., McCart E.A., Muir J.M., Bouten R.M., Slaven J.E., Mungunsukh O., Bylicky M.A., Wilkins W.L., Lee S.-H., Gudmundsson K.O. (2021). Effects of Captopril Against Radiation Injuries in the Göttingen Minipig Model of Hematopoietic-Acute Radiation Syndrome. PLoS ONE.

[856] Ward W.F., Molteni A., Ts’ao C., Hinz J.M. (1990). The Effect of Captopril on Benign and Malignant Reactions in Irradiated Rat Skin. Br. J. Radiol..

[857] Ryu S., Kolozsvary A., Jenrow K.A., Brown S.L., Kim J.H. (2007). Mitigation of Radiation-Induced Optic Neuropathy in Rats by ACE Inhibitor Ramipril: Importance of Ramipril Dose and Treatment Time. J. Neuro-Oncol..

[858] Lee T.C., Greene-Schloesser D., Payne V., Diz D.I., Hsu F.-C., Kooshki M., Mustafa R., Riddle D.R., Zhao W., Chan M.D. (2012). Chronic Administration of the Angiotensin-Converting Enzyme Inhibitor, Ramipril, Prevents Fractionated Whole-Brain Irradiation-Induced Perirhinal Cortex-Dependent Cognitive Impairment. Radiat. Res..

[859] Jenrow K.A., Liu J., Brown S.L., Kolozsvary A., Lapanowski K., Kim J.H. (2011). Combined Atorvastatin and Ramipril Mitigate Radiation-Induced Impairment of Dentate Gyrus Neurogenesis. J. Neuro-Oncol..

[860] Saager M., Hahn E.W., Peschke P., Brons S., Huber P.E., Debus J., Karger C.P. (2020). Ramipril Reduces Incidence and Prolongates Latency Time of Radiation-Induced Rat Myelopathy After Photon and Carbon Ion Irradiation. J. Radiat. Res..

[861] Jenrow K.A., Brown S.L., Liu J., Kolozsvary A., Lapanowski K., Kim J.H. (2010). Ramipril Mitigates Radiation-Induced Impairment of Neurogenesis in the Rat Dentate Gyrus. Radiat. Oncol..

[862] Robbins M.E., Zhao W., Garcia-Espinosa M.A., Diz D.I. (2010). Renin-Angiotensin System Blockers and Modulation of Radiation-Induced Brain Injury. Curr. Drug Targets.

[863] Moore E.D., Kooshki M., Metheny-Barlow L.J., Gallagher P.E., Robbins M.E. (2013). Angiotensin-(1–7) Prevents Radiation-Induced Inflammation in Rat Primary Astrocytes Through Regulation of MAP Kinase Signaling. Free Radic. Biol. Med..

[864] Dixon S., O’connor A.T., Brooks-Noreiga C., Clark M.A., Levy A., Castejon A.M. (2024). Role of Renin Angiotensin System Inhibitors and Metformin in Glioblastoma Therapy: A Review. Cancer Chemother. Pharmacol..

[865] Ursu R., Thomas L., Psimaras D., Chinot O., Le Rhun E., Ricard D., Charissoux M., Cuzzubbo S., Sejalon F., Quillien V. (2019). Angiotensin II Receptor Blockers, Steroids and Radiotherapy in Glioblastoma—A Randomised Multicentre Trial (ASTER Trial). An ANOCEF Study. Eur. J. Cancer.

[866] Rodgers K.E., Espinoza T., Roda N., Meeks C.J., Hill C., Louie S.G., Dizerega G.S. (2012). Accelerated Hematopoietic Recovery with Angiotensin-(1–7) After Total Body Radiation. Int. J. Radiat. Biol..

[867] Willey J.S., Bracey D.N., Gallagher P.E., Tallant E.A., Wiggins W.F., Callahan M.F., Smith T.L., Emory C.L. (2016). Angiotensin-(1-7) Attenuates Skeletal Muscle Fibrosis and Stiffening in a Mouse Model of Extremity Sarcoma Radiation Therapy. JBJS.

[868] McFall A., Nicklin S.A., Work L.M. (2020). The Counter Regulatory Axis of the Renin Angiotensin System in the Brain and Ischaemic Stroke: Insight from Preclinical Stroke Studies and Therapeutic Potential. Cell. Signal..

[869] Sharma G.P., Nissen A., Gasperetti T., Foeckler J., Frei A.C., Kuehn R., Gerhartz M., Heinzelman P., Romero P.A., Zenga J. (2025). Activation of Angiotensin-Converting Enzyme 2 Mitigates Gastrointestinal Acute Radiation Syndrome. Int. J. Radiat. Oncol. Biol. Phys..

[870] Gaugler M.-H., Vereycken-Holler V., Squiban C., Vandamme M., Vozenin-Brotons M.-C., Benderitter M. (2005). Pravastatin Limits Endothelial Activation After Irradiation and Decreases the Resulting Inflammatory and Thrombotic Responses. Radiat. Res..

[871] Holler V., Buard V., Gaugler M.-H., Guipaud O., Baudelin C., Sache A., Perez M.d.R., Squiban C., Tamarat R., Milliat F. (2009). Pravastatin Limits Radiation-Induced Vascular Dysfunction in the Skin. J. Investig. Dermatol..

[872] Doi H., Matsumoto S., Odawara S., Shikata T., Kitajima K., Tanooka M., Takada Y., Tsujimura T., Kamikonya N., Hirota S. (2017). Pravastatin Reduces Radiation-Induced Damage in Normal Tissues. Exp. Ther. Med..

[873] Ait-Aissa K., Guo X., Klemmensen M., Juhr D., Leng L.N., Koval O.M., Grumbach I.M. (2024). Short-Term Statin Treatment Reduces, and Long-Term Statin Treatment Abolishes, Chronic Vascular Injury by Radiation Therapy. J. Am. Heart Assoc..

[874] Bourgier C., Auperin A., Rivera S., Boisselier P., Petit B., Lang P., Lassau N., Taourel P., Tetreau R., Azria D. (2019). Pravastatin Reverses Established Radiation-Induced Cutaneous and Subcutaneous Fibrosis in Patients with Head and Neck Cancer: Results of the Biology-Driven Phase 2 Clinical Trial Pravacur. Int. J. Radiat. Oncol. Biol. Phys..

[875] Zhang K., He X., Zhou Y., Gao L., Qi Z., Chen J., Gao X. (2015). Atorvastatin Ameliorates Radiation-Induced Cardiac Fibrosis in Rats. Radiat. Res..

[876] Haydont V., Bourgier C., Pocard M., Lusinchi A., Aigueperse J., Mathé D., Bourhis J., Vozenin-Brotons M.-C. (2007). Pravastatin Inhibits the Rho/CCN2/Extracellular Matrix Cascade in Human Fibrosis Explants and Improves Radiation-Induced Intestinal Fibrosis in Rats. Clin. Cancer Res..

[877] Monceau V., Pasinetti N., Schupp C., Pouzoulet F., Opolon P., Vozenin M.-C. (2010). Modulation of the Rho/ROCK Pathway in Heart and Lung After Thorax Irradiation Reveals Targets to Improve Normal Tissue Toxicity. Curr. Drug Targets.

[878] Williams J.P., Hernady E., Johnston C.J., Reed C.M., Fenton B., Okunieff P., Finkelstein J.N. (2004). Effect of Administration of Lovastatin on the Development of Late Pulmonary Effects After Whole-Lung Irradiation in a Murine Model. Radiat. Res..

[879] Lenarczyk M., Su J., Haworth S.T., Komorowski R., Fish B.L., Migrino R.Q., Harmann L., Hopewell J.W., Kronenberg A., Patel S. (2015). Simvastatin Mitigates Increases in Risk Factors for and the Occurrence of Cardiac Disease Following 10 Gy Total Body Irradiation. Pharmacol. Res. Perspect..

[880] Walls G.M., Ghita M., Herron B., Edgar K.S., Kuburas R., Watson C.J., Grieve D.J., Cole A.J., Jain S., Butterworth K.T. (2024). A Multimodality Assessment of the Protective Capacity of Statin Therapy in a Mouse Model of Radiation Cardiotoxicity. Radiother. Oncol..

[881] Ziegler V., Henninger C., Simiantonakis I., Buchholzer M., Ahmadian M.R., Budach W., Fritz G. (2017). Rho Inhibition by Lovastatin Affects Apoptosis and DSB Repair of Primary Human Lung Cells in Vitro and Lung Tissue in Vivo Following Fractionated Irradiation. Cell Death Dis..

[882] Boulet J., Peña J., Hulten E.A., Neilan T.G., Dragomir A., Freeman C., Lambert C., Hijal T., Nadeau L., Brophy J.M. (2019). Statin Use and Risk of Vascular Events Among Cancer Patients After Radiotherapy to the Thorax, Head, and Neck. J. Am. Heart Assoc..

[883] Atkins K.M., Bitterman D.S., Chaunzwa T.L., Williams C.L., Rahman R., Kozono D.E., Baldini E.H., Aerts H.J.W.L., Tamarappoo B.K., Hoffmann U. (2021). Statin Use, Heart Radiation Dose, and Survival in Locally Advanced Lung Cancer. Pract. Radiat. Oncol..

[884] Huang Y., Lin J., Chen W., Shia B., Wu S. (2024). Statin Therapy Reduces Radiation-Induced Cardiotoxicity in Patients With Breast Cancer Receiving Adjuvant Radiotherapy. J. Am. Heart Assoc..

[885] Lin C.-Y., Chang C.-L., Lin K.-C., Chen W.-M., Shia B.-C., Kuo P.-H., Wu S.-Y. (2024). Statin Use Reduces Radiation-Induced Stroke Risk in Advanced Nasopharyngeal Carcinoma Patients. Radiother. Oncol..

[886] Yu J.-M., Chang C.-L., Lin K.-C., Chen W.-M., Shia B.-C., Wu S.-Y. (2024). Statin Use During Concurrent Chemoradiotherapy for Advanced Nasopharyngeal Cancer. J. Natl. Compr. Cancer Netw..

[887] Jang H., Lee J., Park S., Myung H., Kang J., Kim K., Kim H., Jang W.-S., Lee S.-J., Shim S. (2018). Pravastatin Attenuates Acute Radiation-Induced Enteropathy and Improves Epithelial Cell Function. Front. Pharmacol..

[888] Kim J.M., Kim H., Oh S.H., Jang W.I., Lee S.B., Park M., Kim S., Park S., Shim S., Jang H. (2022). Combined Administration of Pravastatin and Metformin Attenuates Acute Radiation-Induced Intestinal Injury in Mouse and Minipig Models. Int. J. Mol. Sci..

[889] Cui M., Xiao H., Li Y., Zhang S., Dong J., Wang B., Zhu C., Jiang M., Zhu T., He J. (2019). Sexual Dimorphism of Gut Microbiota Dictates Therapeutics Efficacy of Radiation Injuries. Adv. Sci..

[890] Ghasemi A., Ghashghai Z., Akbari J., Yazdani-Charati J., Salehifar E., Hosseinimehr S.J. (2019). Topical Atorvastatin 1% for Prevention of Skin Toxicity in Patients Receiving Radiation Therapy for Breast Cancer: A Randomized, Double-Blind, Placebo-Controlled Trial. Eur. J. Clin. Pharmacol..

[891] Danesh M., Ghasemi A., Hamzehgardeshi Z., Charati J.Y., Hosseinimehr S.J. (2025). The Efficacy of Oral Atorvastatin for Skin Toxicity in Breast Cancer Patients Undergoing Radiotherapy: A Randomized, Double-Blind, Placebo-Controlled Trial. Curr. Pharm. Des..

[892] Anscher M.S., Chang M.G., Moghanaki D., Rosu M., Mikkelsen R.B., Holdford D., Skinner V., Grob B.M., Sanyal A., Wang A. (2018). A Phase II Study to Prevent Radiation-Induced Rectal Injury with Lovastatin. Am. J. Clin. Oncol..

[893] Ricco N., Kron S.J. (2023). Statins in Cancer Prevention and Therapy. Cancers.

[894] Rabbani Z.N., Anscher M.S., Zhang X., Chen L., Samulski T.V., Li C.-Y., Vujaskovic Z. (2003). Soluble TGFbeta Type II Receptor Gene Therapy Ameliorates Acute Radiation-Induced Pulmonary Injury in Rats. Int. J. Radiat. Oncol. Biol. Phys..

[895] Du S.-S., Qiang M., Zeng Z.-C., Zhou J., Tan Y.-S., Zhang Z.-Y., Zeng H.-Y., Liu Z.-S. (2010). Radiation-Induced Liver Fibrosis Is Mitigated by Gene Therapy Inhibiting Transforming Growth Factor-β Signaling in the Rat. Int. J. Radiat. Oncol. Biol. Phys..

[896] Anscher M.S., Thrasher B., Zgonjanin L., Rabbani Z.N., Corbley M.J., Fu K., Sun L., Lee W.-C., Ling L.E., Vujaskovic Z. (2008). Small Molecular Inhibitor of Transforming Growth Factor-β Protects Against Development of Radiation-Induced Lung Injury. Int. J. Radiat. Oncol. Biol. Phys..

[897] Park J., Ryu S.-H., Choi E.K., Ahn S.D., Park E., Choi K.-C., Lee S. (2015). SKI2162, an Inhibitor of the TGF-β Type I Receptor (ALK5), Inhibits Radiation-Induced Fibrosis in Mice. Oncotarget.

[898] Park J., Choi J., Cho I., Sheen Y.Y. (2022). Radiotherapy-Induced Oxidative Stress and Fibrosis in Breast Cancer Are Suppressed by Vactosertib, a Novel, Orally Bioavailable TGF-β/ALK5 Inhibitor. Sci. Rep..

[899] Cruz-Morande S., Dotor J., San-Julian M. (2022). P144 a Transforming Growth Factor Beta Inhibitor Peptide, Generates Antifibrogenic Effects in a Radiotherapy Induced Fibrosis Model. Curr. Oncol..

[900] Rabender C., Mezzaroma E., Mauro A.G., Mullangi R., Abbate A., Anscher M., Hart B., Mikkelsen R. (2016). IPW-5371 Proves Effective as a Radiation Countermeasure by Mitigating Radiation-Induced Late Effects. Radiat. Res..

[901] Fish B.L., Hart B., Gasperetti T., Narayanan J., Gao F., Veley D., Pierce L., Himburg H.A., MacVittie T., Medhora M. (2023). IPW-5371 Mitigates the Delayed Effects of Acute Radiation Exposure in WAG/RijCmcr Rats When Started 15 Days After PBI with Bone Marrow Sparing. Int. J. Radiat. Biol..

[902] Nicolay N.H., Bickelhaupt S., Perez R.L., Bostel T., Roeder F., Debus J., Peschke P., Huber P.E. (2014). Multi-Pathway Inhibition as a Novel Strategy to Attenuate Radiation-Induced Pulmonary Fibrosis. Int. J. Radiat. Oncol. Biol. Phys..

[903] Xavier S., Piek E., Fujii M., Javelaud D., Mauviel A., Flanders K.C., Samuni A.M., Felici A., Reiss M., Yarkoni S. (2004). Amelioration of Radiation-Induced Fibrosis: Inhibition of Transforming Growth Factor-Beta Signaling by Halofuginone. J. Biol. Chem..

[904] Calik M., Yavas G., Calik S., Yavas C., Celik Z., Sargon M., Esme H. (2017). Amelioration of Radiation-Induced Lung Injury by Halofuginone: An Experimental Study in Wistar–Albino Rats. Hum. Exp. Toxicol..

[905] Aimo A., Spitaleri G., Panichella G., Lupón J., Emdin M., Bayes-Genis A. (2022). Pirfenidone as a Novel Cardiac Protective Treatment. Heart Fail. Rev..

[906] Qin W., Liu B., Yi M., Li L., Tang Y., Wu B., Yuan X. (2018). Antifibrotic Agent Pirfenidone Protects Against Development of Radiation-Induced Pulmonary Fibrosis in a Murine Model. Radiat. Res..

[907] Sun Y.-W., Zhang Y.-Y., Ke X.-J., Wu X.-J., Chen Z.-F., Chi P. (2018). Pirfenidone Prevents Radiation-Induced Intestinal Fibrosis in Rats by Inhibiting Fibroblast Proliferation and Differentiation and Suppressing the TGF-Β1/Smad/CTGF Signaling Pathway. Eur. J. Pharmacol..

[908] Simone N.L., Soule B.P., Gerber L., Augustine E., Smith S., Altemus R.M., Mitchell J.B., Camphausen K.A. (2007). Oral Pirfenidone in Patients with Chronic Fibrosis Resulting from Radiotherapy: A Pilot Study. Radiat. Oncol..

[909] Chen C., Zeng B., Xue D., Cao R., Liao S., Yang Y., Li Z., Kang M., Chen C., Xu B. (2022). Pirfenidone for the Prevention of Radiation-Induced Lung Injury in Patients with Locally Advanced Oesophageal Squamous Cell Carcinoma: A Protocol for a Randomised Controlled Trial. BMJ Open.

[910] Chen M., Hou Z., Yao Q., Chen H., Shao Q., Li M., Wang J., Zhu Z., Peng F., Wei S. (2024). Pirfenidone in the Treatment of Radiation-Induced Lung Injury: A Randomized, Controlled, Multicenter Clinical Trial. Int. J. Radiat. Oncol. Biol. Phys..

[911] Tu J., Chen X., Li C., Liu C., Huang Y., Wang X., Liang H., Yuan X. (2024). Nintedanib Mitigates Radiation-Induced Pulmonary Fibrosis by Suppressing Epithelial Cell Inflammatory Response and Inhibiting Fibroblast-to-Myofibroblast Transition. Int. J. Biol. Sci..

[912] Zhang K., Ren L., Zhai Y. (2025). Effect and Mechanism of Nintedanib on Acute and Chronic Radiation-Induced Lung Injury in Mice. PLoS ONE.

[913] Dy G.K., Prasad D., Kumar P., Attwood K., Adjei A.A. (2021). A Phase 2 Randomized, Double-Blind, Placebo-Controlled Study Evaluating Nintedanib Versus Placebo as Prophylaxis Against Radiation Pneumonitis in Patients with Unresectable NSCLC Undergoing Chemoradiation Therapy. J. Thorac. Oncol..

[914] Rimner A., Moore Z.R., Lobaugh S., Geyer A., Gelblum D.Y., Abdulnour R.-E.E., Shepherd A.F., Shaverdian N., Wu A.J., Cuaron J. (2023). Randomized Phase 2 Placebo-Controlled Trial of Nintedanib for the Treatment of Radiation Pneumonitis. Int. J. Radiat. Oncol. Biol. Phys..

[915] Lipson K., Moustafa M., Akbarpour M., Fouse S., Kriegsmann M., Zhou C., Liu K., Lasitschka F., Weichert W., Seeley T. (2017). Therapeutic Pamrevlumab (FG-3019) Is More Effective than Pirfenidone or Nintedanib in a Mouse Radiation-Induced Lung Fibrosis Model. Eur. Respir. J..

[916] Wang J., Zheng H., Hauer-Jensen M. (2001). Influence of Short-Term Octreotide Administration on Chronic Tissue Injury, Transforming Growth Factor Beta (TGF-Beta) Overexpression, and Collagen Accumulation in Irradiated Rat Intestine. J. Pharmacol. Exp. Ther..

[917] Young K.H., Gough M.J., Crittenden M. (2015). Tumor Immune Remodeling by TGFβ Inhibition Improves the Efficacy of Radiation Therapy. Oncoimmunology.

[918] Kim B.-G., Malek E., Choi S.H., Ignatz-Hoover J.J., Driscoll J.J. (2021). Novel Therapies Emerging in Oncology to Target the TGF-β Pathway. J. Hematol. Oncol..

[919] Yamazaki T., Gunderson A.J., Gilchrist M., Whiteford M., Kiely M.X., Hayman A., O’Brien D., Ahmad R., Manchio J.V., Fox N. (2022). Galunisertib plus Neoadjuvant Chemoradiotherapy in Patients with Locally Advanced Rectal Cancer: A Single-Arm, Phase 2 Trial. Lancet Oncol..

[920] Whitnall M.H., Elliott T.B., Harding R.A., Inal C.E., Landauer M.R., Wilhelmsen C.L., McKinney L., Miner V.L., Jackson W.E., Loria R.M. (2000). Androstenediol Stimulates Myelopoiesis and Enhances Resistance to Infection in Gamma-Irradiated Mice. Int. J. Immunopharmacol..

[921] Singh V.K., Shafran R.L., Inal C.E., Jackson W.E., Whitnall M.H. (2005). Effects of Whole-Body Gamma Irradiation and 5-Androstenediol Administration on Serum G-CSF. Immunopharmacol. Immunotoxicol..

[922] Stickney D.R., Dowding C., Authier S., Garsd A., Onizuka-Handa N., Frincke J. (2005). Novel Hormone HE2100 Protects Rhesus Macaques From Lethal Total Body Irradiation (TBI)- Induced Severe Neutropenia and Thrombocytopenia. Int. J. Radiat. Oncol. Biol. Phys..

[923] Stickney D.R., Dowding C., Garsd A., Ahlem C., Whitnall M., McKeon M., Reading C., Frincke J. (2006). 5-Androstenediol Stimulates Multilineage Hematopoiesis in Rhesus Monkeys with Radiation-Induced Myelosuppression. Int. Immunopharmacol..

[924] Stickney D.R., Dowding C., Authier S., Garsd A., Onizuka-Handa N., Reading C., Frincke J.M. (2007). 5-Androstenediol Improves Survival in Clinically Unsupported Rhesus Monkeys with Radiation-Induced Myelosuppression. Int. Immunopharmacol..

[925] Kim J.S., Jang W.S., Lee S., Son Y., Park S., Lee S.S. (2015). A Study of the Effect of Sequential Injection of 5-Androstenediol on Irradiation-Induced Myelosuppression in Mice. Arch. Pharmacal Res..

[926] Wu T., Liu W., Fan T., Zhong H., Zhou H., Guo W., Zhu X. (2020). 5-Androstenediol Prevents Radiation Injury in Mice by Promoting NF-κB Signaling and Inhibiting AIM2 Inflammasome Activation. Biomed. Pharmacother..

[927] Singh V.K., Seed T.M. (2024). The Potential Value of 5-Androstenediol in Countering Acute Radiation Syndrome. Drug Discov. Today.

[928] Grace M.B., Singh V.K., Rhee J.G., Jackson W.E., Kao T.-C., Whitnall M.H. (2012). 5-AED Enhances Survival of Irradiated Mice in a G-CSF-Dependent Manner, Stimulates Innate Immune Cell Function, Reduces Radiation-Induced DNA Damage and Induces Genes That Modulate Cell Cycle Progression and Apoptosis. J. Radiat. Res..

[929] Aerts-Kaya F.S.F., Visser T.P., Arshad S., Frincke J., Stickney D.R., Reading C.L., Wagemaker G. (2012). 5-Androstene-3β,17β-Diol Promotes Recovery of Immature Hematopoietic Cells Following Myelosuppressive Radiation and Synergizes with Thrombopoietin. Int. J. Radiat. Oncol. Biol. Phys..

[930] Stickney D.R., Groothuis J.R., Ahlem C., Kennedy M., Miller B.S., Onizuka-Handa N., Schlangen K.M., Destiche D., Reading C., Garsd A. (2010). Preliminary Clinical Findings on NEUMUNE as a Potential Treatment for Acute Radiation Syndrome. J. Radiol. Prot..

[931] Cheng Y., Li W., Gui R., Wang C., Song J., Wang Z., Wang X., Shen Y., Wang Z., Hao L. (2021). Dual Characters of GH-IGF1 Signaling Pathways in Radiotherapy and Post-Radiotherapy Repair of Cancers. Front. Cell Dev. Biol..

[932] Boguszewski M.C.S., Boguszewski C.L., Chemaitilly W., Cohen L.E., Gebauer J., Higham C., Hoffman A.R., Polak M., Yuen K.C.J., Alos N. (2022). Safety of Growth Hormone Replacement in Survivors of Cancer and Intracranial and Pituitary Tumours: A Consensus Statement. Eur. J. Endocrinol..

[933] Carlo-Stella C., Di Nicola M., Milani R., Longoni P., Milanesi M., Bifulco C., Stucchi C., Guidetti A., Cleris L., Formelli F. (2004). Age- and Irradiation-Associated Loss of Bone Marrow Hematopoietic Function in Mice Is Reversed by Recombinant Human Growth Hormone. Exp. Hematol..

[934] Chen S., Xu Y., Wang S., Shen M., Chen F., Chen M., Wang A., Cheng T., Su Y., Wang J. (2012). Subcutaneous Administration of rhIGF-I Post Irradiation Exposure Enhances Hematopoietic Recovery and Survival in BALB/c Mice. J. Radiat. Res..

[935] Valenciano A., Henríquez-Hernández L.A., Moreno M., Lloret M., Lara P.C. (2012). Role of IGF-1 Receptor in Radiation Response. Transl. Oncol..

[936] Caz V., Elvira M., Tabernero M., Grande A.G., Lopez-Plaza B., de Miguel E., Largo C., Santamaria M. (2015). Growth Hormone Protects the Intestine Preserving Radiotherapy Efficacy on Tumors: A Short-Term Study. PLoS ONE.

[937] Chen B.J., Cui X., Sempowski G.D., Chao N.J. (2003). Growth Hormone Accelerates Immune Recovery Following Allogeneic T-Cell–Depleted Bone Marrow Transplantation in Mice. Exp. Hematol..

[938] Mylonas P.G., Matsouka P.T., Papandoniou E.V., Vagianos C., Kalfarentzos F., Alexandrides T.K. (2000). Growth Hormone and Insulin-like Growth Factor I Protect Intestinal Cells from Radiation Induced Apoptosis. Mol. Cell. Endocrinol..

[939] Raguso C.A., Leverve X., Pichard C. (2002). Protective Effects of Recombinant Growth Hormone on Intestinal Mucosa in Rats Receiving Abdominal Radiotherapy. Clin. Nutr..

[940] Tekin S.B., Ertekin M.V., Erdogan F., Sezen O., Karslioglu I., Gepdiremen A., Serifoglu K., Altas S. (2006). Is Growth Hormone a Radioprotective Agent?. J. Eur. Acad. Dermatol. Venereol..

[941] Zhou D., Deoliveira D., Kang Y., Choi S.S., Li Z., Chao N.J., Chen B.J. (2013). Insulin-like Growth Factor 1 Mitigates Hematopoietic Toxicity After Lethal Total Body Irradiation. Int. J. Radiat. Oncol. Biol. Phys..

[942] Pejchal J., Tichy A., Kmochova A., Fikejzlova L., Kubelkova K., Milanova M., Lierova A., Filipova A., Muckova L., Cizkova J. (2022). Mitigation of Ionizing Radiation-Induced Gastrointestinal Damage by Insulin-Like Growth Factor-1 in Mice. Front. Pharmacol..

[943] Chitnis M.M., Lodhia K.A., Aleksic T., Gao S., Protheroe A.S., Macaulay V.M. (2014). IGF-1R Inhibition Enhances Radiosensitivity and Delays Double-Strand Break Repair by Both Non-Homologous End-Joining and Homologous Recombination. Oncogene.

[944] Qiu W., Leibowitz B., Zhang L., Yu J. (2010). Growth Factors Protect Intestinal Stem Cells from Radiation-Induced Apoptosis by Suppressing PUMA Through the PI3K/AKT/P53 Axis. Oncogene.

[945] Liao W., Chen X., Zhang S., Chen J., Liu C., Yu K., Zhang Y., Chen M., Chen F., Shen M. (2024). Megakaryocytic IGF1 Coordinates Activation and Ferroptosis to Safeguard Hematopoietic Stem Cell Regeneration After Radiation Injury. Cell Commun. Signal..

[946] Bohin N., McGowan K.P., Keeley T.M., Carlson E.A., Yan K.S., Samuelson L.C. (2020). Insulin-like Growth Factor-1 and mTORC1 Signaling Promote the Intestinal Regenerative Response After Irradiation Injury. Cell. Mol. Gastroenterol. Hepatol..

[947] Yang S.Y., Hoy M., Fuller B., Sales K.M., Seifalian A.M., Winslet M.C. (2010). Pretreatment with Insulin-like Growth Factor I Protects Skeletal Muscle Cells Against Oxidative Damage via PI3K/Akt and ERK1/2 MAPK Pathways. Lab. Investig..

[948] Grundmann O., Fillinger J.L., Victory K.R., Burd R., Limesand K.H. (2010). Restoration of Radiation Therapy-Induced Salivary Gland Dysfunction in Mice by Post Therapy IGF-1 Administration. BMC Cancer.

[949] Gunning J.A., Gilman K.E., Zúñiga T.M., Simpson R.J., Limesand K.H. (2024). Parotid Glands Have a Dysregulated Immune Response Following Radiation Therapy. PLoS ONE.

[950] Bahamondes Lorca V.A., Wu S. (2024). Growth Hormone and Radiation Therapy: Friend, Foe, or Both?. Endocr. Relat. Cancer.

[951] Kiang J.G., Zhai M., Liao P.-J., Elliott T.B., Gorbunov N.V. (2014). Ghrelin Therapy Improves Survival After Whole-Body Ionizing Irradiation or Combined with Burn or Wound: Amelioration of Leukocytopenia, Thrombocytopenia, Splenomegaly, and Bone Marrow Injury. Oxidative Med. Cell. Longev..

[952] Wang Z., Yang W.L., Jacob A., Aziz M., Wang P. (2015). Human Ghrelin Mitigates Intestinal Injury and Mortality After Whole Body Irradiation in Rats. PLoS ONE.

[953] Kiang J.G., Anderson M.N., Smith J.T. (2018). Ghrelin Therapy Mitigates Bone Marrow Injury and Splenocytopenia by Sustaining Circulating G-CSF and KC Increases After Irradiation Combined with Wound. Cell Biosci..

[954] Kwak S.-Y., Shim S., Park S., Kim H., Lee S.-J., Kim M.-J., Jang W.-S., Kim Y., Jang H. (2021). Ghrelin Reverts Intestinal Stem Cell Loss Associated with Radiation-Induced Enteropathy by Activating Notch Signaling. Phytomedicine.

[955] Yamaga S., Murao A., Chaung W., Lapin D., Lee Y., Wang P., Brenner M. (2025). Ghrelin Mitigates Partial Body Irradiation-Induced Gastrointestinal Acute Radiation Syndrome by Promoting Intestinal Stem Cell Regeneration. Mol. Med..

[956] Shah K.G., Wu R., Jacob A., Blau S.A., Ji Y., Dong W., Marini C.P., Ravikumar T.S., Coppa G.F., Wang P. (2009). Human Ghrelin Ameliorates Organ Injury and Improves Survival After Radiation Injury Combined with Severe Sepsis. Mol. Med..

[957] Gorbunov N.V., Kiang J.G. (2017). Ghrelin Therapy Decreases Incidents of Intracranial Hemorrhage in Mice After Whole-Body Ionizing Irradiation Combined with Burn Trauma. Int. J. Mol. Sci..

[958] Kiang J.G., Smith J.T., Anderson M.N., Umali M.V., Ho C., Zhai M., Lin B., Jiang S. (2019). A Novel Therapy, Using Ghrelin with Pegylated G-CSF, Inhibits Brain Hemorrhage from Ionizing Radiation or Combined Radiation Injury. Pharm. Pharmacol. Int. J..

[959] Morgenstern L., Patin C.S., Krohn H.L., Hiatt N. (1970). Prolongation of Survival in Lethally Irradiated Dogs by Pancreatic Duct Ligation. Arch. Surg..

[960] Hauer Jensen M., Sauer T., Berstad T., Nygaard K. (1985). Influence of Pancreatic Secretion on Late Radiation Enteropathy in the Rat. Acta Radiol. Oncol..

[961] Fu Q., Berbée M., Wang W., Boerma M., Wang J., Schmid H.A., Hauer-Jensen M. (2011). Preclinical Evaluation of Som230 as a Radiation Mitigator in a Mouse Model: Postexposure Time Window and Mechanisms of Action. Radiat. Res..

[962] Fu Q., Berbée M., Boerma M., Wang J., Schmid H.A., Hauer-Jensen M. (2009). The Somatostatin Analog SOM230 (Pasireotide) Ameliorates Injury of the Intestinal Mucosa and Increases Survival After Total-Body Irradiation by Inhibiting Exocrine Pancreatic Secretion. Radiat. Res..

[963] Martenson J.A., Halyard M.Y., Sloan J.A., Proulx G.M., Miller R.C., Deming R.L., Dick S.J., Johnson H.A., Tai T.H.P., Zhu A.W. (2008). Phase III, Double-Blind Study of Depot Octreotide versus Placebo in the Prevention of Acute Diarrhea in Patients Receiving Pelvic Radiation Therapy: Results of North Central Cancer Treatment Group N00CA. J. Clin. Oncol..

[964] Yavuz M.N., Yavuz A.A., Aydin F., Can G., Kavgaci H. (2002). The Efficacy of Octreotide in the Therapy of Acute Radiation-Induced Diarrhea: A Randomized Controlled Study. Int. J. Radiat. Oncol. Biol. Phys..

[965] Zachariah B., Gwede C.K., James J., Ajani J., Chin L.J., Donath D., Rosenthal S.A., Kane B.L., Rotman M., Berk L. (2010). Octreotide Acetate in Prevention of Chemoradiation-Induced Diarrhea in Anorectal Cancer: Randomized RTOG Trial 0315. J. Natl. Cancer Inst..

[966] Ma D.-J., Li Z.-J., Wang X.-Y., Zhu X.-J., Sun Y.-L. (2019). Octreotide Treatment of Cancer Chemoradiotherapy-Induced Diarrhoea: A Meta-Analysis of Randomized Controlled Trials. Transl. Cancer Res..

[967] Sun J.-X., Yang N. (2014). Role of Octreotide in Post Chemotherapy and/or Radiotherapy Diarrhea: Prophylaxis or Therapy?. Asia-Pac. J. Clin. Oncol..

[968] Foster R.G. (2021). Melatonin. Curr. Biol..

[969] Amini P., Mirtavoos-Mahyari H., Motevaseli E., Shabeeb D., Musa A.E., Cheki M., Farhood B., Yahyapour R., Shirazi A., Goushbolagh N.A. (2019). Mechanisms for Radioprotection by Melatonin; Can It Be Used as a Radiation Countermeasure?. Curr. Mol. Pharmacol..

[970] Motallebzadeh E., Tameh A.A., Zavareh S.A.T., Farhood B., Aliasgharzedeh A., Mohseni M. (2020). Neuroprotective Effect of Melatonin on Radiation-Induced Oxidative Stress and Apoptosis in the Brainstem of Rats. J. Cell. Physiol..

[971] Tripathi A.M., Khan S., Chaudhury N.K. (2022). Radiomitigation by Melatonin in C57BL/6 Mice: Possible Implications as Adjuvant in Radiotherapy and Chemotherapy. In Vivo.

[972] Eskandari A., Mahmoudzadeh A., Shirazi A., Esmaely F., Carnovale C., Cheki M. (2020). Melatonin a Promising Candidate for DNA Double-Stranded Breaks Reduction in Patients Undergoing Abdomen-Pelvis Computed Tomography Examinations. Anti-Cancer Agents Med. Chem..

[973] Fernández-Gil B., Moneim A.E.A., Ortiz F., Shen Y.-Q., Soto-Mercado V., Mendivil-Perez M., Guerra-Librero A., Acuña-Castroviejo D., Molina-Navarro M.M., García-Verdugo J.M. (2017). Melatonin Protects Rats from Radiotherapy-Induced Small Intestine Toxicity. PLoS ONE.

[974] Rezapoor S., Shirazi A., Abbasi S., Bazzaz J.T., Izadi P., Rezaeejam H., Valizadeh M., Soleimani-Mohammadi F., Najafi M. (2017). Modulation of Radiation-Induced Base Excision Repair Pathway Gene Expression by Melatonin. J. Med. Phys..

[975] Esmaely F., Mahmoudzadeh A., Cheki M., Shirazi A. (2020). The Radioprotective Effect of Melatonin Against Radiation-Induced DNA Double-Strand Breaks in Radiology. J. Cancer Res. Ther..

[976] Dehdari Ebrahimi N., Sadeghi A., Falamarzi K., Shahlaee M.A., Azarpira N. (2024). Radio-Protective Effects of Melatonin Therapy Against Testicular Oxidative Stress: A Systematic Review and Meta-Analysis of Rodent Models. Ann. Med. Surg..

[977] Shedid S.M., Abdel-Aziz N., Algeda F.R., Saada H.N. (2025). The Mitigating Effect of Melatonin Against Radiation-Induced Inflammation and Disturbance of Reproductive Hormones in Female Albino Rats. Dose Response.

[978] Erol F.S., Topsakal C., Ozveren M.F., Kaplan M., Ilhan N., Ozercan I.H., Yildiz O.G. (2004). Protective Effects of Melatonin and Vitamin E in Brain Damage Due to Gamma Radiation: An Experimental Study. Neurosurg. Rev..

[979] Karslioglu I., Ertekin M.V., Taysi S., Koçer I., Sezen O., Gepdiremen A., Koç M., Bakan N. (2005). Radioprotective Effects of Melatonin on Radiation-Induced Cataract. J. Radiat. Res..

[980] Amini P., Ashrafizadeh M., Motevaseli E., Najafi M., Shirazi A. (2020). Mitigation of Radiation-Induced Hematopoietic System Injury by Melatonin. Environ. Toxicol..

[981] Sadeghi H., Bagheri H., Shekarchi B., Javadi A., Najafi M. (2020). Mitigation of Radiation-Induced Gastrointestinal System Injury by Melatonin: A Histopathological Study. Curr. Drug Res. Rev..

[982] Farhood B., Aliasgharzadeh A., Amini P., Rezaeyan A., Tavassoli A., Motevaseli E., Shabeeb D., Eleojo Musa A., Najafi M. (2019). Mitigation of Radiation-Induced Lung Pneumonitis and Fibrosis Using Metformin and Melatonin: A Histopathological Study. Medicina.

[983] Wu X., Ji H., Wang Y., Gu C., Gu W., Hu L., Zhu L. (2019). Melatonin Alleviates Radiation-Induced Lung Injury via Regulation of miR-30e/NLRP3 Axis. Oxidative Med. Cell. Longev..

[984] Ortiz F., Acuña-Castroviejo D., Doerrier C., Dayoub J.C., López L.C., Venegas C., García J.A., López A., Volt H., Luna-Sánchez M. (2015). Melatonin Blunts the Mitochondrial/NLRP3 Connection and Protects Against Radiation-Induced Oral Mucositis. J. Pineal Res..

[985] Vijayalaxmi, Meltz M.L., Reiter R.J., Herman T.S. (1999). Melatonin and Protection from Genetic Damage in Blood and Bone Marrow: Whole-Body Irradiation Studies in Mice. J. Pineal Res..

[986] Khan S., Adhikari J.S., Rizvi M.A., Chaudhury N.K. (2017). Melatonin Attenuates 60 Co γ-Ray-Induced Hematopoietic, Immunological and Gastrointestinal Injuries in C57BL/6 Male Mice. Environ. Toxicol..

[987] Vasin M.V., Ushakov I.B., Kovtun V.Y., Semenova L.A., Koroleva L.V., Galkin A.A., Afanas’ev R.V. (2014). Therapeutic Effect of Long-Term Melatonin Treatment on the Course and Fatal Outcome of Modeled Acute Radiation Sickness. Bull. Exp. Biol. Med..

[988] Abdullaev S.A., Glukhov S.I., Gaziev A.I. (2021). Radioprotective and Radiomitigative Effects of Melatonin in Tissues with Different Proliferative Activity. Antioxidants.

[989] Lissoni P., Meregalli S., Nosetto L., Barni S., Tancini G., Fossati V., Maestroni G. (1996). Increased Survival Time in Brain Glioblastomas by a Radioneuroendocrine Strategy with Radiotherapy plus Melatonin Compared to Radiotherapy Alone. Oncology.

[990] Lissoni P., Rovelli F., Brivio F., Fumagalli L., Brera G. (2008). A Study of Immunoendocrine Strategies with Pineal Indoles and Interleukin-2 to Prevent Radiotherapy-Induced Lymphocytopenia in Cancer Patients. In Vivo.

[991] Ben-David M.A., Elkayam R., Gelernter I., Pfeffer R.M. (2016). Melatonin for Prevention of Breast Radiation Dermatitis: A Phase II, Prospective, Double-Blind Randomized Trial. Isr. Med. Assoc. J..

[992] Onseng K., Johns N.P., Khuayjarernpanishk T., Subongkot S., Priprem A., Hurst C., Johns J. (2017). Beneficial Effects of Adjuvant Melatonin in Minimizing Oral Mucositis Complications in Head and Neck Cancer Patients Receiving Concurrent Chemoradiation. J. Altern. Complement. Med..

[993] Elsabagh H.H., Moussa E., Mahmoud S.A., Elsaka R.O., Abdelrahman H. (2020). Efficacy of Melatonin in Prevention of Radiation-Induced Oral Mucositis: A Randomized Clinical Trial. Oral Dis..

[994] Lozano A., Marruecos J., Rubió J., Farré N., Gómez-Millán J., Morera R., Planas I., Lanzuela M., Vázquez-Masedo M.G., Cascallar L. (2021). Randomized Placebo-Controlled Phase II Trial of High-Dose Melatonin Mucoadhesive Oral Gel for the Prevention and Treatment of Oral Mucositis in Patients with Head and Neck Cancer Undergoing Radiation Therapy Concurrent with Systemic Treatment. Clin. Transl. Oncol..

[995] Kouhi Habibi N., Shabestani Monfared A., Ebrahimnejad Gorji K., Karimi M., Moghadamnia A.A., Tourani M., Borzoueisileh S., Niksirat F. (2019). The Protective Effects of Melatonin on Blood Cell Counts of Rectal Cancer Patients Following Radio-Chemotherapy: A Randomized Controlled Trial. Clin. Transl. Oncol..

[996] Alidadi S., Shabestani Monfared A., Amiri M., Zabihi E., Assadollahi E., Gholami A., Moazezi Z., Abedian Z. (2022). The Efficacy of Melatonin Against Radiotoxicity of Iodine-131 and Its Response to Treatment in Hyperthyroid Patients: A Randomized Controlled Trial. Nucl. Med. Rev. Cent. East. Eur..

[997] Zetner D., Kamby C., Christophersen C., Gülen S., Paulsen C.B., Piga E., Hoffmeyer B., Mahmood F., Rosenberg J. (2023). Effect of Melatonin Cream on Acute Radiation Dermatitis in Patients with Primary Breast Cancer: A Double-Blind, Randomized, Placebo-Controlled Trial. J. Pineal Res..

[998] Cakmak Karaer I., Simsek G., Yildiz A., Vardi N., Polat A., Tanbek K., Gurocak S., Parlakpinar H. (2016). Melatonin’s Protective Effect on the Salivary Gland Against Ionized Radiation Damage in Rats. J. Oral Pathol. Med..

[999] Sedighi Pashaki A., Sheida F., Moaddab Shoar L., Hashem T., Fazilat-Panah D., Nemati Motehaver A., Ghanbari Motlagh A., Nikzad S., Bakhtiari M., Tapak L. (2023). A Randomized, Controlled, Parallel-Group, Trial on the Long-Term Effects of Melatonin on Fatigue Associated with Breast Cancer and Its Adjuvant Treatments. Integr. Cancer Ther..

[1000] Sadeghi Yazdankhah S., Javadinia S.A., Welsh J.S., Mosalaei A. (2025). Efficacy of Melatonin in Alleviating Radiotherapy-Induced Fatigue, Anxiety, and Depression in Breast Cancer Patients: A Randomized, Triple-Blind, Placebo-Controlled Trial. Integr. Cancer Ther..

[1001] Mukhopadhyay N.D., Khorasanchi A., Pandey S., Nemani S., Parker G., Deng X., Arthur D.W., Urdaneta A., Del Fabbro E. (2024). Melatonin Supplementation for Cancer-Related Fatigue in Patients with Early Stage Breast Cancer Receiving Radiotherapy: A Double-Blind Placebo-Controlled Trial. Oncologist.

[1002] Farhood B., Goradel N.H., Mortezaee K., Khanlarkhani N., Salehi E., Nashtaei M.S., Mirtavoos-mahyari H., Motevaseli E., Shabeeb D., Musa A.E. (2019). Melatonin as an Adjuvant in Radiotherapy for Radioprotection and Radiosensitization. Clin. Transl. Oncol..

[1003] Talib W.H., Alsayed A.R., Abuawad A., Daoud S., Mahmod A.I. (2021). Melatonin in Cancer Treatment: Current Knowledge and Future Opportunities. Molecules.

[1004] Wang Y., Jin B., Ai F., Duan C., Lu Y., Dong T., Fu Q. (2012). The Efficacy and Safety of Melatonin in Concurrent Chemotherapy or Radiotherapy for Solid Tumors: A Meta-Analysis of Randomized Controlled Trials. Cancer Chemother. Pharmacol..

[1005] Zharinov G.M., Bogomolov O.A., Chepurnaya I.V., Neklasova N.Y., Anisimov V.N. (2020). Melatonin Increases Overall Survival of Prostate Cancer Patients with Poor Prognosis After Combined Hormone Radiation Treatment. Oncotarget.

[1006] Hrushesky W.J.M., Lis C.G., Levin R.D., Grutsch J.F., Birdsall T., Wood P.A., Huff D.F.Q., Reynolds J.L., Pearl D.K., Shen X. (2022). Daily Evening Melatonin Prolongs Survival Among Patients with Advanced Non-Small-Cell Lung Cancer. Biol. Rhythm Res..

[1007] Sookprasert A., Johns N.P., Phunmanee A., Pongthai P., Cheawchanwattana A., Johns J., Konsil J., Plaimee P., Porasuphatana S., Jitpimolmard S. (2014). Melatonin in Patients with Cancer Receiving Chemotherapy: A Randomized, Double-Blind, Placebo-Controlled Trial. Anticancer Res..

[1008] Xu G., Wu H., Zhang J., Li D., Wang Y., Wang Y., Zhang H., Lu L., Li C., Huang S. (2015). Metformin Ameliorates Ionizing Irradiation-Induced Long-Term Hematopoietic Stem Cell Injury in Mice. Free Radic. Biol. Med..

[1009] Karmanova E., Chernikov A., Usacheva A., Ivanov V., Bruskov V. (2023). Metformin Counters Oxidative Stress and Mitigates Adverse Effects of Radiation Exposure: An Overview. Fundam. Clin. Pharmacol..

[1010] Abdullaev S., Minkabirova G., Karmanova E., Bruskov V., Gaziev A. (2018). Metformin Prolongs Survival Rate in Mice and Causes Increased Excretion of Cell-Free DNA in the Urine of X-Irradiated Rats. Mutat. Res. Genet. Toxicol. Environ. Mutagen..

[1011] Mortezaee K., Shabeeb D., Musa A.E., Najafi M., Farhood B. (2019). Metformin as a Radiation Modifier; Implications to Normal Tissue Protection and Tumor Sensitization. Curr. Clin. Pharmacol..

[1012] Jang H., Kim S., Kim H., Oh S.H., Kwak S.Y., Joo H.-W., Lee S.B., Jang W.I., Park S., Shim S. (2022). Metformin Protects the Intestinal Barrier by Activating Goblet Cell Maturation and Epithelial Proliferation in Radiation-Induced Enteropathy. Int. J. Mol. Sci..

[1013] Karmanova E.E., Chernikov A.V., Popova N.R., Sharapov M.G., Ivanov V.E., Bruskov V.I. (2023). Metformin Mitigates Radiation Toxicity Exerting Antioxidant and Genoprotective Properties. Naunyn Schmiedeb. Arch. Pharmacol..

[1014] Malekzadeh H., Surucu Y., Chinnapaka S., Yang K.S., Arellano J.A., Samadi Y., Epperly M.W., Greenberger J.S., Rubin J.P., Ejaz A. (2024). Metformin and Adipose-Derived Stem Cell Combination Therapy Alleviates Radiation-Induced Skin Fibrosis in Mice. Stem Cell Res. Ther..

[1015] Siteni S., Barron S., Luitel K., Shay J.W. (2024). Radioprotective Effect of the Anti-Diabetic Drug Metformin. PLoS ONE.

[1016] Da F., Guo J., Yao L., Gao Q., Jiao S., Miao X., Liu J. (2021). Pretreatment with Metformin Protects Mice from Whole-Body Irradiation. J. Radiat. Res..

[1017] Miller R.C., Murley J.S., Grdina D.J. (2014). Metformin Exhibits Radiation Countermeasures Efficacy When Used Alone or in Combination with Sulfhydryl Containing Drugs. Radiat. Res..

[1018] Yang J.-Y., Liu M.-J., Lv L., Guo J.-R., He K.-Y., Zhang H., Wang K.-K., Cui C.-Y., Yan B.-Z., Du D.-D. (2022). Metformin Alleviates Irradiation-Induced Intestinal Injury by Activation of FXR in Intestinal Epithelia. Front. Microbiol..

[1019] Wang J., Wang Y., Han J., Mei H., Yu D., Ding Q., Zhang T., Wu G., Peng G., Lin Z. (2017). Metformin Attenuates Radiation-Induced Pulmonary Fibrosis in a Murine Model. Radiat. Res..

[1020] Kim J.-M., Yoo H., Kim J.-Y., Oh S.H., Kang J.W., Yoo B.R., Han S.Y., Kim C.S., Choi W.H., Lee E.-J. (2018). Metformin Alleviates Radiation-Induced Skin Fibrosis via the Downregulation of FOXO3. Cell Physiol. Biochem..

[1021] Cao J., Liu H., An Q., Han F. (2023). Metformin alleviates pathologic pain in mice with radiation dermatitis by inhibiting p38MAPK/NF-κB signaling pathway. Nan Fang Yi Ke Da Xue Xue Bao.

[1022] Ayoub R., Ruddy R.M., Cox E., Oyefiade A., Derkach D., Laughlin S., Ades-aron B., Shirzadi Z., Fieremans E., Macintosh B.J. (2020). Assessment of Cognitive and Neural Recovery in Survivors of Pediatric Brain Tumors in a Pilot Clinical Trial Using Metformin. Nat. Med..

[1023] Jang W.I., Kim M.-S., Lim J.S., Yoo H.J., Seo Y.S., Han C.J., Park S.C., Kay C.S., Kim M., Jang H.S. (2015). Survival Advantage Associated with Metformin Usage in Hepatocellular Carcinoma Patients Receiving Radiotherapy: A Propensity Score Matching Analysis. Anticancer. Res..

[1024] Bikas A., Van Nostrand D., Jensen K., Desale S., Mete M., Patel A., Wartofsky L., Vasko V., Burman K.D. (2016). Metformin Attenuates 131I-Induced Decrease in Peripheral Blood Cells in Patients with Differentiated Thyroid Cancer. Thyroid.

[1025] Yu J.-M., Hsieh M.-C., Qin L., Zhang J., Wu S.-Y. (2019). Metformin Reduces Radiation-Induced Cardiac Toxicity Risk in Patients Having Breast Cancer. Am. J. Cancer Res..

[1026] Razmjoo S., Cheki M., Hosseini M., Bagheri A., Razaghi S., Hosseini S. (2025). Investigation of the Radiation Protection Effect of Metformin Against Complications Caused by Radiation Therapy in Patients with Prostate Cancer: Randomized Clinical Trial. J. Iran. Med. Counc..

[1027] Kim J.O., McDonald M.O., Ong A., Koul R., Dubey A., Hunter W., Ahmed S., Quon H., Yee D., Parliament M. (2021). Gastrointestinal and Genitourinary Toxicity Profiles of Metformin versus Placebo in Men with Prostate Cancer Receiving Prostate Radiotherapy: Interim Toxicity Results of a Double-Blinded, Multicenter, Phase II Randomized Controlled Trial. Radiat. Oncol..

[1028] Tsakiridis T., Pond G.R., Wright J., Ellis P.M., Ahmed N., Abdulkarim B., Roa W., Robinson A., Swaminath A., Okawara G. (2021). Metformin in Combination with Chemoradiotherapy in Locally Advanced Non-Small Cell Lung Cancer: The OCOG-ALMERA Randomized Clinical Trial. JAMA Oncol..

[1029] Wheaton W.W., Weinberg S.E., Hamanaka R.B., Soberanes S., Sullivan L.B., Anso E., Glasauer A., Dufour E., Mutlu G.M., Budigner G.S. (2014). Metformin Inhibits Mitochondrial Complex I of Cancer Cells to Reduce Tumorigenesis. Elife.

[1030] Galal M.A., Al-Rimawi M., Hajeer A., Dahman H., Alouch S., Aljada A. (2024). Metformin: A Dual-Role Player in Cancer Treatment and Prevention. Int. J. Mol. Sci..

[1031] Skinner H., Hu C., Tsakiridis T., Santana-Davila R., Lu B., Erasmus J.J., Doemer A.J., Videtic G.M.M., Coster J., Yang A.X. (2021). Addition of Metformin to Concurrent Chemoradiation in Patients with Locally Advanced Non-Small Cell Lung Cancer: The NRG-LU001 Phase 2 Randomized Clinical Trial. JAMA Oncol..

[1032] Liu Z., Lei X., Li X., Cai J.-M., Gao F., Yang Y.-Y. (2018). Toll-like Receptors and Radiation Protection. Eur. Rev. Med. Pharmacol. Sci..

[1033] Wang Z.-D., Qiao Y.-L., Tian X.-F., Zhang X.-Q., Zhou S.-X., Liu H.-X., Chen Y. (2012). Toll-like Receptor 5 Agonism Protects Mice from Radiation Pneumonitis and Pulmonary Fibrosis. Asian Pac. J. Cancer Prev..

[1034] Bai H., Sun F., Yang G., Wang L., Zhang Q., Zhang Q., Zhan Y., Chen J., Yu M., Li C. (2019). CBLB502, a Toll-like Receptor 5 Agonist, Offers Protection Against Radiation-Induced Male Reproductive System Damage in Mice. Biol. Reprod..

[1035] Kim J.-Y., Park J.-H., Seo S.-M., Park J.-I., Jeon H.-Y., Lee H.-K., Yoo R.-J., Lee Y.-J., Woo S.-K., Lee W.-J. (2019). Radioprotective Effect of Newly Synthesized Toll-like Receptor 5 Agonist, KMRC011, in Mice Exposed to Total-Body Irradiation. J. Radiat. Res..

[1036] Burdelya L.G., Krivokrysenko V.I., Tallant T.C., Strom E., Gleiberman A.S., Gupta D., Kurnasov O.V., Fort F.L., Osterman A.L., DiDonato J.A. (2008). An Agonist of Toll-Like Receptor 5 Has Radioprotective Activity in Mouse and Primate Models. Science.

[1037] Burdelya L.G., Gleiberman A.S., Toshkov I., Aygun-Sunar S., Bapardekar M., Manderscheid-Kern P., Bellnier D., Krivokrysenko V.I., Feinstein E., Gudkov A.V. (2012). Toll-like Receptor 5 Agonist Protects Mice from Dermatitis and Oral Mucositis Caused by Local Radiation: Implications for Head and Neck Cancer Radiotherapy. Int. J. Radiat. Oncol. Biol. Phys..

[1038] Krivokrysenko V.I., Toshkov I.A., Gleiberman A.S., Krasnov P., Shyshynova I., Bespalov I., Maitra R.K., Narizhneva N.V., Singh V.K., Whitnall M.H. (2015). The Toll-Like Receptor 5 Agonist Entolimod Mitigates Lethal Acute Radiation Syndrome in Non-Human Primates. PLoS ONE.

[1039] Krivokrysenko V.I., Shakhov A.N., Singh V.K., Bone F., Kononov Y., Shyshynova I., Cheney A., Maitra R.K., Purmal A., Whitnall M.H. (2012). Identification of Granulocyte Colony-Stimulating Factor and Interleukin-6 as Candidate Biomarkers of CBLB502 Efficacy as a Medical Radiation Countermeasure. J. Pharmacol. Exp. Ther..

[1040] Brackett C.M., Greene K.F., Aldrich A.R., Trageser N.H., Pal S., Molodtsov I., Kandar B.M., Burdelya L.G., Abrams S.I., Gudkov A.V. (2021). Signaling Through TLR5 Mitigates Lethal Radiation Damage by Neutrophil-Dependent Release of MMP-9. Cell Death Discov..

[1041] Wang Q., Duan J., Hong J., Ding K., Tai F., Zhu J., Fu H., Zheng X., Ge C. (2024). Toll-like Receptor Agonist CBLB502 Protects Against Radiation-Induced Intestinal Injury in Mice. In Vivo.

[1042] Toshkov I.A., Gleiberman A.S., Mett V.L., Hutson A.D., Singh A.K., Gudkov A.V., Burdelya L.G. (2017). Mitigation of Radiation-Induced Epithelial Damage by the TLR5 Agonist Entolimod in a Mouse Model of Fractionated Head and Neck Irradiation. Radiat. Res..

[1043] Burdelya L.G., Brackett C.M., Kojouharov B., Gitlin I.I., Leonova K.I., Gleiberman A.S., Aygun-Sunar S., Veith J., Johnson C., Haderski G.J. (2013). Central Role of Liver in Anticancer and Radioprotective Activities of Toll-like Receptor 5 Agonist. Proc. Natl. Acad. Sci. USA.

[1044] Howell B.A., Siler S.Q., Shoda L.K.M., Yang Y., Woodhead J.L., Watkins P.B. (2014). A Mechanistic Model of Drug-Induced Liver Injury AIDS the Interpretation of Elevated Liver Transaminase Levels in a Phase I Clinical Trial. CPT Pharmacomet. Syst. Pharmacol..

[1045] Lee H.-S., Cho D.-W., Han J.-S., Han S.-C., Woo S.K., Jun S.-Y., Lee W.-J., Yoon S., Pak S.-I., Lee S.-J. (2020). KMRC011, an Agonist of Toll-like Receptor 5, Mitigates Irradiation-Induced Tissue Damage and Mortality in Cynomolgus Monkeys. J. Immunotoxicol..

[1046] Ko J., Kim J., Choi Y.-K., Nahm S.-S., Kim J., Seo S.-M., Seo J.-S., Lee W., Chung W.K., Eom K. (2022). Clinical Evaluation of Toll-like Receptor-5 Agonist for Radiation-Induced Oral Mucositis in Beagle Dogs. Front. Vet. Sci..

[1047] Yang E., Choi H., Park J.-S., Noh Y.-W., Choi C.-M., Lee W.-J., Ko J.-W., Kim J. (2021). A First-in-Human Study of KMRC011, a Potential Treatment for Acute Radiation Syndrome, to Explore Tolerability, Pharmacokinetics, and Pharmacodynamics. Clin. Transl. Sci..

[1048] Saha S., Bhanja P., Liu L., Alfieri A.A., Yu D., Kandimalla E.R., Agrawal S., Guha C. (2012). TLR9 Agonist Protects Mice from Radiation-Induced Gastrointestinal Syndrome. PLoS ONE.

[1049] Kurkjian C.J., Guo H., Montgomery N.D., Cheng N., Yuan H., Merrill J.R., Sempowski G.D., Brickey W.J., Ting J.P.-Y. (2017). The Toll–Like Receptor 2/6 Agonist, FSL–1 Lipopeptide, Therapeutically Mitigates Acute Radiation Syndrome. Sci. Rep..

[1050] Brickey W.J., Caudell D.L., Macintyre A.N., Olson J.D., Dai Y., Li S., Dugan G.O., Bourland J.D., O’Donnell L.M., Tooze J.A. (2023). The TLR2/TLR6 Ligand FSL-1 Mitigates Radiation-Induced Hematopoietic Injury in Mice and Nonhuman Primates. Proc. Natl. Acad. Sci. USA.

[1051] Holmes-Hampton G.P., Kumar V.P., Valenzia K., Ghosh S.P. (2024). FSL-1: A Synthetic Peptide Increases Survival in a Murine Model of Hematopoietic Acute Radiation Syndrome. Radiat. Res..

[1052] Estrela J.M., Mena S., Obrador E., Benlloch M., Castellano G., Salvador R., Dellinger R.W. (2017). Polyphenolic Phytochemicals in Cancer Prevention and Therapy: Bioavailability versus Bioefficacy. J. Med. Chem..

[1053] Li K., Ji M., Sun X., Shan J., Su G. (2024). Food Polyphenols in Radiation-Related Diseases: The Roles and Possible Mechanisms. Curr. Nutr. Rep..

[1054] Pradeep K., Park S.H., Ko K.C. (2008). Hesperidin a Flavanoglycone Protects Against Gamma-Irradiation Induced Hepatocellular Damage and Oxidative Stress in Sprague-Dawley Rats. Eur. J. Pharmacol..

[1055] Singh V.K., Grace M.B., Parekh V.I., Whitnall M.H., Landauer M.R. (2009). Effects of Genistein Administration on Cytokine Induction in Whole-Body Gamma Irradiated Mice. Int. Immunopharmacol..

[1056] Ozgen S.Ç., Dökmeci D., Akpolat M., Karadağ C.H., Gündüz O., Erbaş H., Benian O., Uzal C., Turan F.N. (2012). The Protective Effect of Curcumin on Ionizing Radiation-Induced Cataractogenesis in Rats. Balk. Med. J..

[1057] Abernathy L.M., Fountain M.D., Rothstein S.E., David J.M., Yunker C.K., Rakowski J., Lonardo F., Joiner M.C., Hillman G.G. (2015). Soy Isoflavones Promote Radioprotection of Normal Lung Tissue by Inhibition of Radiation-Induced Activation of Macrophages and Neutrophils. J. Thorac. Oncol..

[1058] Zhang H., Yan H., Zhou X., Wang H., Yang Y., Zhang J., Wang H. (2017). The Protective Effects of Resveratrol Against Radiation-Induced Intestinal Injury. BMC Complement. Altern. Med..

[1059] Xie L.-W., Cai S., Zhao T.-S., Li M., Tian Y. (2020). Green Tea Derivative (-)-Epigallocatechin-3-Gallate (EGCG) Confers Protection Against Ionizing Radiation-Induced Intestinal Epithelial Cell Death Both in Vitro and in Vivo. Free Radic. Biol. Med..

[1060] Miko Enomoto T., Johnson T., Peterson N., Homer L., Walts D., Johnson N. (2005). Combination Glutathione and Anthocyanins as an Alternative for Skin Care During External-Beam Radiation. Am. J. Surg..

[1061] Brooker S., Martin S., Pearson A., Bagchi D., Earl J., Gothard L., Hall E., Porter L., Yarnold J. (2006). Double-Blind, Placebo-Controlled, Randomised Phase II Trial of IH636 Grape Seed Proanthocyanidin Extract (GSPE) in Patients with Radiation-Induced Breast Induration. Radiother. Oncol..

[1062] Rao S., Dinkar C., Vaishnav L.K., Rao P., Rai M.P., Fayad R., Baliga M.S. (2014). The Indian Spice Turmeric Delays and Mitigates Radiation-Induced Oral Mucositis in Patients Undergoing Treatment for Head and Neck Cancer: An Investigational Study. Integr. Cancer Ther..

[1063] Belcaro G., Hosoi M., Pellegrini L., Appendino G., Ippolito E., Ricci A., Ledda A., Dugall M., Cesarone M.R., Maione C. (2014). A Controlled Study of a Lecithinized Delivery System of Curcumin (Meriva®) to Alleviate the Adverse Effects of Cancer Treatment. Phytotherapy Res..

[1064] Patil K., Guledgud M.V., Kulkarni P.K., Keshari D., Tayal S. (2015). Use of Curcumin Mouthrinse in Radio-Chemotherapy Induced Oral Mucositis Patients: A Pilot Study. J. Clin. Diagn. Res..

[1065] Mansourian A., Amanlou M., Shirazian S., Moosavian Jahromi Z., Amirian A. (2015). The Effect of “Curcuma Longa” Topical Gel on Radiation-Induced Oral Mucositis in Patients with Head and Neck Cancer. Int. J. Radiat. Res..

[1066] Charantimath S. (2016). Use of Curcumin in Radiochemotherapy Induced Oral Mucositis Patients: A Control Trial Study. World Acad. Sci. Eng. Technol..

[1067] Rao S., Hegde S.K., Baliga-Rao M.P., Lobo J., Palatty P.L., George T., Baliga M.S. (2017). Sandalwood Oil and Turmeric-Based Cream Prevents Ionizing Radiation-Induced Dermatitis in Breast Cancer Patients: Clinical Study. Medicines.

[1068] Vakharia B., Adhvaryu M., Reddy N. (2018). Curcumin Prevents Mucositis and Improves Patient Compliance in Head & Neck Cancer Patients Undergoing Radio-Chemotherapy. Ann. Med. Chem. Res..

[1069] Alsalim S.A., Diajil A.R. (2024). The Effect of Curcumin Oral Gel on Radiation-Induced Oral Mucositis in Relation to Salivary Epidermal Growth Factor. J. Emerg. Med. Trauma Acute Care.

[1070] Delavarian Z., Pakfetrat A., Ghazi A., Jaafari M.R., Homaei Shandiz F., Dalirsani Z., Mohammadpour A.H., Rahimi H.R. (2019). Oral Administration of Nanomicelle Curcumin in the Prevention of Radiotherapy-Induced Mucositis in Head and Neck Cancers. Spec. Care Dent..

[1071] Arun P., Sagayaraj A., Azeem Mohiyuddin S.M., Santosh D. (2020). Role of Turmeric Extract in Minimising Mucositis in Patients Receiving Radiotherapy for Head and Neck Squamous Cell Cancer: A Randomised, Placebo-Controlled Trial. J. Laryngol. Otol..

[1072] Soni T.P., Gupta A.K., Sharma L.M., Singhal H., Sharma S., Gothwal R.S. (2021). A Randomized, Placebo-Controlled Study to Evaluate the Effect of Bio-Enhanced Turmeric Formulation on Radiation-Induced Oral Mucositis. ORL.

[1073] Kia S.J., Basirat M., Saedi H.S., Arab S.A. (2021). Effects of Nanomicelle Curcumin Capsules on Prevention and Treatment of Oral Mucosits in Patients Under Chemotherapy with or Without Head and Neck Radiotherapy: A Randomized Clinical Trial. BMC Complement. Med. Ther..

[1074] Ramezani V., Ghadirian S., Shabani M., Boroumand M.A., Daneshvar R., Saghafi F. (2023). Efficacy of Curcumin for Amelioration of Radiotherapy-Induced Oral Mucositis: A Preliminary Randomized Controlled Clinical Trial. BMC Cancer.

[1075] Ryan J.L., Heckler C.E., Ling M., Katz A., Williams J.P., Pentland A.P., Morrow G.R. (2013). Curcumin for Radiation Dermatitis: A Randomized, Double-Blind, Placebo-Controlled Clinical Trial of Thirty Breast Cancer Patients. Radiat. Res..

[1076] Ryan Wolf J., Heckler C.E., Guido J.J., Peoples A.R., Gewandter J.S., Ling M., Vinciguerra V.P., Anderson T., Evans L., Wade J. (2018). Oral Curcumin for Radiation Dermatitis: A URCC NCORP Study of 686 Breast Cancer Patients. Support. Care Cancer.

[1077] Palatty P.L., Azmidah A., Rao S., Jayachander D., Thilakchand K.R., Rai M.P., Haniadka R., Simon P., Ravi R., Jimmy R. (2014). Topical Application of a Sandal Wood Oil and Turmeric Based Cream Prevents Radiodermatitis in Head and Neck Cancer Patients Undergoing External Beam Radiotherapy: A Pilot Study. Br. J. Radiol..

[1078] Talakesh T., Tabatabaee N., Atoof F., Aliasgharzadeh A., Sarvizade M., Farhood B., Najafi M. (2022). Effect of Nano-Curcumin on Radiotherapy-Induced Skin Reaction in Breast Cancer Patients: A Randomized, Triple-Blind, Placebo-Controlled Trial. Curr. Radiopharm..

[1079] Heydari B., Sheikhalishahi S., Hoseinzade F., Shabani M., Ramezani V., Saghafi F. (2025). Topical Curcumin for Prevention of Radiation-Induced Dermatitis: A Pilot Double-Blind, Placebo-Controlled Trial. Cancer Investig..

[1080] Hejazi J., Rastmanesh R., Taleban F.-A., Molana S.-H., Hejazi E., Ehtejab G., Hara N. (2016). Effect of Curcumin Supplementation During Radiotherapy on Oxidative Status of Patients with Prostate Cancer: A Double Blinded, Randomized, Placebo-Controlled Study. Nutr. Cancer.

[1081] Saadipoor A., Razzaghdoust A., Simforoosh N., Mahdavi A., Bakhshandeh M., Moghadam M., Abdollahi H., Mofid B. (2019). Randomized, Double-Blind, Placebo-Controlled Phase II Trial of Nanocurcumin in Prostate Cancer Patients Undergoing Radiotherapy. Phytotherapy Res..

[1082] Najafizade N., Ebrahimi A., Hemati S. (2024). A Randomized, Double-Blinded, Placebo-Controlled Study on the Protective Effects of Curcumin Against Chemoradiotherapy-Induced Enteritis. Middle East J. Cancer.

[1083] Zhao H., Xie P., Li X., Zhu W., Sun X., Sun X., Chen X., Xing L., Yu J. (2015). A Prospective Phase II Trial of EGCG in Treatment of Acute Radiation-Induced Esophagitis for Stage III Lung Cancer. Radiother. Oncol..

[1084] Zhao H., Jia L., Chen G., Li X., Meng X., Zhao X., Xing L., Zhu W. (2019). A Prospective, Three-Arm, Randomized Trial of EGCG for Preventing Radiation-Induced Esophagitis in Lung Cancer Patients Receiving Radiotherapy. Radiother. Oncol..

[1085] Zhu W., Zhao Y., Zhang S., Li X., Xing L., Zhao H., Yu J. (2021). Evaluation of Epigallocatechin-3-Gallate as a Radioprotective Agent During Radiotherapy of Lung Cancer Patients: A 5-Year Survival Analysis of a Phase 2 Study. Front. Oncol..

[1086] Li X., Xing L., Zhang Y., Xie P., Zhu W., Meng X., Wang Y., Kong L., Zhao H., Yu J. (2020). Phase II Trial of Epigallocatechin-3-Gallate in Acute Radiation-Induced Esophagitis for Esophagus Cancer. J. Med. Food.

[1087] Zhu W., Jia L., Chen G., Zhao H., Sun X., Meng X., Zhao X., Xing L., Yu J., Zheng M. (2016). Epigallocatechin-3-Gallate Ameliorates Radiation-Induced Acute Skin Damage in Breast Cancer Patients Undergoing Adjuvant Radiotherapy. Oncotarget.

[1088] Zhao H., Zhu W., Zhao X., Li X., Zhou Z., Zheng M., Meng X., Kong L., Zhang S., He D. (2022). Efficacy of Epigallocatechin-3-Gallate in Preventing Dermatitis in Patients with Breast Cancer Receiving Postoperative Radiotherapy: A Double-Blind, Placebo-Controlled, Phase 2 Randomized Clinical Trial. JAMA Dermatol..

[1089] Xie J., Jia L., Xie P., Yin X., Zhu W., Zhao H., Wang X., Meng X., Xing L., Zhao H. (2023). Efficacy and Safety of Epigallocatechin-3-Gallate in Treatment Acute Severe Dermatitis in Patients with Cancer Receiving Radiotherapy: A Phase I Clinical Trial. Sci. Rep..

[1090] Zhu W., Mei H., Jia L., Zhao H., Li X., Meng X., Zhao X., Xing L., Yu J. (2020). Epigallocatechin-3-Gallate Mouthwash Protects Mucosa from Radiation-Induced Mucositis in Head and Neck Cancer Patients: A Prospective, Non-Randomised, Phase 1 Trial. Investig. New Drugs.

[1091] Lu H., Xie L., Guo L., Gu X., Zhu R., Yang Y., Tang F., Li M., Liu C., Wang D. (2025). EGCG Protects Intestines of Mice and Pelvic Cancer Patients Against Radiation Injury via the Gut Microbiota/D-Tagatose/AMPK Axis. Radiother. Oncol..

[1092] Emami H., Nikoobin F., Roayaei M., Ziya H.R. (2014). Double-Blinded, Randomized, Placebo-Controlled Study to Evaluate the Effectiveness of Green Tea in Preventing Acute Gastrointestinal Complications Due to Radiotherapy. J. Res. Med. Sci..

[1093] Di Franco R., Calvanese M., Murino P., Manzo R., Guida C., Di Gennaro D., Anania C., Ravo V. (2012). Skin Toxicity from External Beam Radiation Therapy in Breast Cancer Patients: Protective Effects of Resveratrol, Lycopene, Vitamin C and Anthocianin (Ixor®). Radiat. Oncol..

[1094] Becker-Schiebe M., Mengs U., Schaefer M., Bulitta M., Hoffmann W. (2011). Topical Use of a Silymarin-Based Preparation to Prevent Radiodermatitis: Results of a Prospective Study in Breast Cancer Patients. Strahlenther. Onkol..

[1095] Karbasforooshan H., Hosseini S., Elyasi S., Fani Pakdel A., Karimi G. (2019). Topical Silymarin Administration for Prevention of Acute Radiodermatitis in Breast Cancer Patients: A Randomized, Double-Blind, Placebo-Controlled Clinical Trial. Phytother. Res..

[1096] Elyasi S., Hosseini S., Niazi Moghadam M.R., Aledavood S.A., Karimi G. (2016). Effect of Oral Silymarin Administration on Prevention of Radiotherapy Induced Mucositis: A Randomized, Double-Blinded, Placebo-Controlled Clinical Trial. Phytother. Res..

[1097] Ahmad I.U., Forman J.D., Sarkar F.H., Hillman G.G., Heath E., Vaishampayan U., Cher M.L., Andic F., Rossi P.J., Kucuk O. (2010). Soy Isoflavones in Conjunction with Radiation Therapy in Patients with Prostate Cancer. Nutr. Cancer.

[1098] Hillman G.G., Singh-Gupta V., Runyan L., Yunker C.K., Rakowski J.T., Sarkar F.H., Miller S., Gadgeel S.M., Sethi S., Joiner M.C. (2011). Soy Isoflavones Radiosensitize Lung Cancer While Mitigating Normal Tissue Injury. Radiother. Oncol..

[1099] Hillman G.G., Singh-Gupta V., Hoogstra D.J., Abernathy L., Rakowski J., Yunker C.K., Rothstein S.E., Sarkar F.H., Gadgeel S., Konski A.A. (2013). Differential Effect of Soy Isoflavones in Enhancing High Intensity Radiotherapy and Protecting Lung Tissue in a Pre-Clinical Model of Lung Carcinoma. Radiother. Oncol..

[1100] Fountain M.D., McLellan L.A., Smith N.L., Loughery B.F., Rakowski J.T., Tse H.Y., Hillman G.G. (2020). Isoflavone-Mediated Radioprotection Involves Regulation of Early Endothelial Cell Death and Inflammatory Signaling in Radiation-Induced Lung Injury. Int. J. Radiat. Biol..

[1101] Dominello M.M., Fountain M.D., Rothstein S.E., Cannon A.C., Abernathy L.M., Hoogstra D., Chen W., Joiner M.C., Hillman G.G. (2017). Radiation Injury to Cardiac Arteries and Myocardium Is Reduced by Soy Isoflavones. J. Radiat. Oncol..

[1102] Hillman G.G., Singh-Gupta V., Lonardo F., Hoogstra D.J., Abernathy L.M., Yunker C.K., Rothstein S.E., Rakowski J., Sarkar F.H., Gadgeel S. (2013). Radioprotection of Lung Tissue by Soy Isoflavones. J. Thorac. Oncol..

[1103] Fountain M.D., Abernathy L.M., Lonardo F., Rothstein S.E., Dominello M.M., Yunker C.K., Chen W., Gadgeel S., Joiner M.C., Hillman G.G. (2015). Radiation-Induced Esophagitis Is Mitigated by Soy Isoflavones. Front. Oncol..

[1104] Calveley V.L., Jelveh S., Langan A., Mahmood J., Yeung I.W.T., Van Dyk J., Hill R.P. (2010). Genistein Can Mitigate the Effect of Radiation on Rat Lung Tissue. Radiat. Res..

[1105] Davis T.A., Clarke T.K., Mog S.R., Landauer M.R. (2007). Subcutaneous Administration of Genistein Prior to Lethal Irradiation Supports Multilineage, Hematopoietic Progenitor Cell Recovery and Survival. Int. J. Radiat. Biol..

[1106] Para A.E., Bezjak A., Yeung I.W.T., Van Dyk J., Hill R.P. (2009). Effects of Genistein Following Fractionated Lung Irradiation in Mice. Radiother. Oncol..

[1107] Son T.G., Gong E.J., Bae M.J., Kim S.D., Heo K., Moon C., Yang K., Kim J.S. (2013). Protective Effect of Genistein on Radiation-Induced Intestinal Injury in Tumor Bearing Mice. BMC Complement. Altern. Med..

[1108] Landauer M.R., Harvey A.J., Kaytor M.D., Day R.M. (2019). Mechanism and Therapeutic Window of a Genistein Nanosuspension to Protect Against Hematopoietic-Acute Radiation Syndrome. J. Radiat. Res..

[1109] Singh V.K., Fatanmi O.O., Wise S.Y., Carpenter A., Nakamura-Peek S., Serebrenik A.A., Kaytor M.D. (2022). A Novel Oral Formulation of BIO 300 Confers Prophylactic Radioprotection from Acute Radiation Syndrome in Mice. Int. J. Radiat. Biol..

[1110] Jackson I.L., Pavlovic R., Alexander A.A., Connors C.Q., Newman D., Mahmood J., Eley J., Harvey A.J., Kaytor M.D., Vujaskovic Z. (2019). BIO 300, a Nanosuspension of Genistein, Mitigates Radiation-Induced Erectile Dysfunction and Sensitizes Human Prostate Cancer Xenografts to Radiation Therapy. Int. J. Radiat. Oncol. Biol. Phys..

[1111] Kaytor M.D., Serebrenik A.A., Lapanowski K., McFall D., Jones M., Movsas B., Simone C.B., Brown S.L. (2023). The Radioprotectant Nano-Genistein Enhances Radiotherapy Efficacy of Lung Tumors in Mice. Transl. Lung Cancer Res..

[1112] Singh V.K., Serebrenik A.A., Wise S.Y., Petrus S.A., Fatanmi O.O., Kaytor M.D. (2024). BIO 300: A Prophylactic Radiation Countermeasure for Acute Radiation Syndrome. Mil. Med..

[1113] Fatanmi O.O., Wise S.Y., Petrus S.A., Melendez-Miranda I., Carpenter A.D., Olson H.M., Serebrenik A.A., Lugo-Roman L.A., Kaytor M.D., Singh V.K. (2025). Prophylactic Administration of BIO 300, A Radiation Medical Countermeasure, Improves Erythrocyte-Associated Parameters in the Rhesus Macaque Model. Mil. Med..

[1114] Tiwari M., Dixit B., Parvez S., Agrawala P.K. (2017). EGCG, a Tea Polyphenol, as a Potential Mitigator of Hematopoietic Radiation Injury in Mice. Biomed. Pharmacother..

[1115] López-Burillo S., Tan D.-X., Mayo J.C., Sainz R.M., Manchester L.C., Reiter R.J. (2003). Melatonin, Xanthurenic Acid, Resveratrol, EGCG, Vitamin C and Alpha-Lipoic Acid Differentially Reduce Oxidative DNA Damage Induced by Fenton Reagents: A Study of Their Individual and Synergistic Actions. J. Pineal Res..

[1116] Cai S., Xie L.-W., Xu J.-Y., Zhou H., Yang C., Tang L.-F., Tian Y., Li M. (2022). (-)-Epigallocatechin-3-Gallate (EGCG) Modulates the Composition of the Gut Microbiota to Protect Against Radiation-Induced Intestinal Injury in Mice. Front. Oncol..

[1117] You H., Wei L., Sun W.-L., Wang L., Yang Z.-L., Liu Y., Zheng K., Wang Y., Zhang W.-J. (2014). The Green Tea Extract Epigallocatechin-3-Gallate Inhibits Irradiation-Induced Pulmonary Fibrosis in Adult Rats. Int. J. Mol. Med..

[1118] Han X., Xu R., Xia Y., Liu Y., Chen S., Shi M., Zou Z., Liang Y., Chen T., Tang Y. (2024). Self-Assembled EGCG Nanoparticles with Enhanced Intracellular ROS Scavenging for Skin Radioprotection. Int. J. Nanomed..

[1119] Bagchi D., Bagchi M., Stohs S.J., Ray S.D., Sen C.K., Preuss H.G. (2002). Cellular Protection with Proanthocyanidins Derived from Grape Seeds. Ann. N. Y. Acad. Sci..

[1120] Said U.Z., Soliman S.M., Azab K.S., El-Tahawy N.A. (2005). Oligomeric Proanthocyanidins (OPCs) Modulating Radiation-Induced Oxidative Stress on Functional and Structural Performance of Eye in Male Rats. Isot. Radiat. Res..

[1121] Huang Y., Zhao H., Cao K., Sun D., Yang Y., Liu C., Cui J., Cheng Y., Li B., Cai J. (2016). Radioprotective Effect of Grape Seed Proanthocyanidins In Vitro and In Vivo. Oxidative Med. Cell. Longev..

[1122] Shen H., Han J., Liu C., Cao F., Huang Y. (2022). Grape Seed Proanthocyanidins Exert a Radioprotective Effect on the Testes and Intestines Through Antioxidant Effects and Inhibition of MAPK Signal Pathways. Front. Med..

[1123] Okunieff P., Xu J., Hu D., Liu W., Zhang L., Morrow G., Pentland A., Ryan J.L., Ding I. (2006). Curcumin Protects Against Radiation-Induced Acute and Chronic Cutaneous Toxicity in Mice and Decreases mRNA Expression of Inflammatory and Fibrogenic Cytokines. Int. J. Radiat. Oncol. Biol. Phys..

[1124] Jagetia G.C., Rajanikant G.K. (2012). Acceleration of Wound Repair by Curcumin in the Excision Wound of Mice Exposed to Different Doses of Fractionated γ Radiation. Int. Wound J..

[1125] Cho Y.J., Yi C.O., Jeon B.T., Jeong Y.Y., Kang G.M., Lee J.E., Roh G.S., Lee J.D. (2013). Curcumin Attenuates Radiation-Induced Inflammation and Fibrosis in Rat Lungs. Korean J. Physiol. Pharmacol..

[1126] Farhood B., Mortezaee K., Goradel N.H., Khanlarkhani N., Salehi E., Nashtaei M.S., Najafi M., Sahebkar A. (2019). Curcumin as an Anti-Inflammatory Agent: Implications to Radiotherapy and Chemotherapy. J. Cell. Physiol..

[1127] Ghanbarzadeh A., Farhood B., Noodeh F.A., Mosaed R., Hassanzadeh G., Bagheri H., Najafi M. (2023). Histopathological Evaluation of Nanocurcumin for Mitigation of Radiation- Induced Small Intestine Injury. Curr. Radiopharm..

[1128] Shen J., Jiao W., Yuan B., Xie H., Chen Z., Wei M., Sun Y., Wu Y., Zhang F., Li Z. (2024). Oral Curcumin-Thioketal-Inulin Conjugate Micelles Against Radiation-Induced Enteritis. Antioxidants.

[1129] Hajimirzaei P., Eyni H., Razmgir M., Abolfazli S., Pirzadeh S., Ahmadi Tabatabaei F.S., Vasigh A., Yazdanian N., Ramezani F., Janzadeh A. (2025). The Analgesic Effect of Curcumin and Nano-Curcumin in Clinical and Preclinical Studies: A Systematic Review and Meta-Analysis. Naunyn Schmiedeb. Arch. Pharmacol..

[1130] Amini P., Saffar H., Nourani M.R., Motevaseli E., Najafi M., Ali Taheri R., Qazvini A. (2018). Curcumin Mitigates Radiation-Induced Lung Pneumonitis and Fibrosis in Rats. Int. J. Mol. Cell. Med..

[1131] Kolivand S., Amini P., Saffar H., Rezapoor S., Motevaseli E., Najafi M., Nouruzi F., Shabeeb D., Musa A.E. (2019). Evaluating the Radioprotective Effect of Curcumin on Rat’s Heart Tissues. Curr. Radiopharm..

[1132] Shi H., Gao X., Li D., Zhang Q., Wang Y., Zheng Y., Cai L., Zhong R., Rui A., Li Z. (2012). A Systemic Administration of Liposomal Curcumin Inhibits Radiation Pneumonitis and Sensitizes Lung Carcinoma to Radiation. Int. J. Nanomed..

[1133] Li W., Jiang L., Lu X., Liu X., Ling M. (2021). Curcumin Protects Radiation-Induced Liver Damage in Rats Through the NF-κB Signaling Pathway. BMC Complement. Med. Ther..

[1134] Chen T., Zhuang B., Huang Y., Liu Y., Yuan B., Wang W., Yuan T., Du L., Jin Y. (2022). Inhaled Curcumin Mesoporous Polydopamine Nanoparticles Against Radiation Pneumonitis. Acta Pharm. Sin. B.

[1135] Abdel-Magied N., Elkady A.A. (2019). Possible Curative Role of Curcumin and Silymarin Against Nephrotoxicity Induced by Gamma-Rays in Rats. Exp. Mol. Pathol..

[1136] Kim J., Park S., Jeon B.-S., Jang W.-S., Lee S.-J., Son Y., Rhim K.-J., Lee S.I., Lee S.-S. (2016). Therapeutic Effect of Topical Application of Curcumin During Treatment of Radiation Burns in a Mini-Pig Model. J. Vet. Sci..

[1137] Dvoretskiy S., Pereira S.L., Das T. (2022). Efficacy of Nutrients in Reducing the Symptoms of Radiation Induced Oral Mucositis in a Hamster Model. Nutr. Cancer.

[1138] Dipalma G., Inchingolo A.M., Latini G., Ferrante L., Nardelli P., Malcangi G., Trilli I., Inchingolo F., Palermo A., Inchingolo A.D. (2024). The Effectiveness of Curcumin in Treating Oral Mucositis Related to Radiation and Chemotherapy: A Systematic Review. Antioxidants.

[1139] Chen Z.-X., Qin Y.-S., Shi B.-H., Gao B.-Y., Tao R.-C., Yong X.-Z. (2024). Effects of Curcumin on Radiation/Chemotherapy-Induced Oral Mucositis: Combined Meta-Analysis, Network Pharmacology, Molecular Docking, and Molecular Dynamics Simulation. Curr. Issues Mol. Biol..

[1140] Wu C.-F., Wu H.-J., Shih C.-L., Yeh T.-P., Ma W.-F. (2024). Efficacy of Turmeric in the Treatment of Oral Mucositis in Patients with Head and Neck Cancer After Radiotherapy or Chemoradiotherapy: A Systematic Review and Meta-Analysis. Front. Pharmacol..

[1141] Amatto P.d.P.G., Chaves L., França S.d.C., Carvalho J.C.T., Carmona F., Pereira A.M.S. (2025). Efficacy of Different Pharmaceutical Forms of Curcuma Longa or Curcumin in Reducing Oral Mucositis Severity and Incidence in Cancer Patients: A Systematic Review and Meta-Analysis. Front. Pharmacol..

[1142] Son Y., Lee H.J., Rho J.K., Chung S.Y., Lee C.G., Yang K., Kim S.H., Lee M., Shin I.S., Kim J.S. (2015). The Ameliorative Effect of Silibinin Against Radiation-Induced Lung Injury: Protection of Normal Tissue Without Decreasing Therapeutic Efficacy in Lung Cancer. BMC Pulm. Med..

[1143] Arghidash F., Gheybi F., Gholamhosseinian H., Kesharwani P., Sahebkar A. (2025). Radioprotective and Radiosensitizing Properties of Silymarin/Silibinin in Response to Ionizing Radiation. Pathol. Res. Pract..

[1144] Ramadan L.A., Roushdy H.M., Abu Senna G.M., Amin N.E., El-Deshw O.A. (2002). Radioprotective Effect of Silymarin Against Radiation Induced Hepatotoxicity. Pharmacol. Res..

[1145] Adhikari M., Arora R. (2016). The Flavonolignan-Silymarin Protects Enzymatic, Hematological, and Immune System Against γ-Radiation-Induced Toxicity. Environ. Toxicol..

[1146] Marzban M., Anjamshoa M., Jafari P., Masoumi H., Ahadi R., Fatehi D. (2017). Effects of Gamma Rays on Rat Testis Tissue According to the Morphological Parameters and Immunohistochemistry: Radioprotective Role of Silymarin. Electron. Physician.

[1147] Mahmoud A.Z., Ibrahim H.A., El-Sawi M.R., Habza M.N. (2020). Effects of Silymarin and Mesenchymal Stem Cells on Hematological and Some Biochemical Changes Induced by Gamma Radiation in Albino Rats. Int. J. Radiat. Biol..

[1148] Kim J.S., Han N.-K., Kim S.-H., Lee H.-J. (2017). Silibinin Attenuates Radiation-Induced Intestinal Fibrosis and Reverses Epithelial-to-Mesenchymal Transition. Oncotarget.

[1149] Agbele A.T., Fasoro O.J., Fabamise O.M., Oluyide O.O., Idolor O.R., Bamise E.A. (2020). Protection Against Ionizing Radiation-Induced Normal Tissue Damage by Resveratrol: A Systematic Review. Eurasian J. Med..

[1150] Dobrzyńska M.M., Gajowik A., Radzikowska J. (2016). The Effect of in Vivo Resveratrol Supplementation in Irradiated Mice on the Induction of Micronuclei in Peripheral Blood and Bone Marrow Reticulocytes. Mutagenesis.

[1151] Farhood B., Hassanzadeh G., Amini P., Shabeeb D., Musa A.E., Khodamoradi E., Mohseni M., Aliasgharzadeh A., Moradi H., Najafi M. (2020). Mitigation of Radiation-Induced Gastrointestinal System Injury Using Resveratrol or Alpha-Lipoic Acid: A Pilot Histopathological Study. Anti-Inflamm. Anti-Allergy Agents Med. Chem..

[1152] Radwan R.R., Karam H.M. (2020). Resveratrol Attenuates Intestinal Injury in Irradiated Rats via PI3K/Akt/mTOR Signaling Pathway. Environ. Toxicol..

[1153] Mohammadi M., Kiani A., Aghaz F., Arkan E., Rashidi K., Najafi M. (2025). Mitigation of Radiation-Induced Acute Hematopoietic System and Intestine Injury by Resveratrol-Loaded Polymeric Nanoparticles After Whole Body Irradiation in Mice. Curr. Radiopharm..

[1154] Zhang H., Zhai Z., Wang Y., Zhang J., Wu H., Wang Y., Li C., Li D., Lu L., Wang X. (2013). Resveratrol Ameliorates Ionizing Irradiation-Induced Long-Term Hematopoietic Stem Cell Injury in Mice. Free Radic. Biol. Med..

[1155] Qin H., Zhang H., Zhang X., Zhang S., Zhu S., Wang H. (2021). Resveratrol Attenuates Radiation Enteritis Through the SIRT1/FOXO3a and PI3K/AKT Signaling Pathways. Biochem. Biophys. Res. Commun..

[1156] Said R.S., El-Demerdash E., Nada A.S., Kamal M.M. (2016). Resveratrol Inhibits Inflammatory Signaling Implicated in Ionizing Radiation-Induced Premature Ovarian Failure Through Antagonistic Crosstalk Between Silencing Information Regulator 1 (SIRT1) and Poly(ADP-Ribose) Polymerase 1 (PARP-1). Biochem. Pharmacol..

[1157] Yahyapour R., Amini P., Saffar H., Motevaseli E., Farhood B., Pooladvand V., Shabeeb D., Musa A.E., Najafi M. (2019). Protective Effect of Metformin, Resveratrol and Alpha-Lipoic Acid on Radiation- Induced Pneumonitis and Fibrosis: A Histopathological Study. Curr. Drug Res. Rev..

[1158] Azmoonfar R., Amini P., Yahyapour R., Rezaeyan A., Tavassoli A., Motevaseli E., Khodamoradi E., Shabeeb D., Musa A.E., Najafi M. (2020). Mitigation of Radiation-Induced Pneumonitis and Lung Fibrosis Using Alpha-Lipoic Acid and Resveratrol. Anti-Inflamm. Anti-Allergy Agents Med. Chem..

[1159] Sheikholeslami S., Khodaverdian S., Dorri-Giv M., Mohammad Hosseini S., Souri S., Abedi-Firouzjah R., Zamani H., Dastranj L., Farhood B. (2021). The Radioprotective Effects of Alpha-Lipoic Acid on Radiotherapy-Induced Toxicities: A Systematic Review. Int. Immunopharmacol..

[1160] Peng R.-M., Lin G.-R., Ting Y., Hu J.-Y. (2018). Oral Delivery System Enhanced the Bioavailability of Stilbenes: Resveratrol and Pterostilbene. Biofactors.

[1161] Garcia-Peris P., Velasco C., Hernandez M., Lozano M.A., Paron L., de la Cuerda C., Breton I., Camblor M., Guarner F. (2016). Effect of Inulin and Fructo-Oligosaccharide on the Prevention of Acute Radiation Enteritis in Patients with Gynecological Cancer and Impact on Quality-of-Life: A Randomized, Double-Blind, Placebo-Controlled Trial. Eur. J. Clin. Nutr..

[1162] Zhao T.-S., Xie L.-W., Cai S., Xu J.-Y., Zhou H., Tang L.-F., Yang C., Fang S., Li M., Tian Y. (2021). Dysbiosis of Gut Microbiota Is Associated with the Progression of Radiation-Induced Intestinal Injury and Is Alleviated by Oral Compound Probiotics in Mouse Model. Front. Cell. Infect. Microbiol..

[1163] Lu L., Li F., Gao Y., Kang S., Li J., Guo J. (2024). Microbiome in Radiotherapy: An Emerging Approach to Enhance Treatment Efficacy and Reduce Tissue Injury. Mol. Med..

[1164] Ferreira M.R., Andreyev H.J.N., Mohammed K., Truelove L., Gowan S.M., Li J., Gulliford S.L., Marchesi J.R., Dearnaley D.P. (2019). Microbiota- and Radiotherapy-Induced Gastrointestinal Side-Effects (MARS) Study: A Large Pilot Study of the Microbiome in Acute and Late-Radiation Enteropathy. Clin. Cancer Res..

[1165] Yi Y., Lu W., Shen L., Wu Y., Zhang Z. (2023). The Gut Microbiota as a Booster for Radiotherapy: Novel Insights into Radio-Protection and Radiation Injury. Exp. Hematol. Oncol..

[1166] Wang W., Cui B., Nie Y., Sun L., Zhang F. (2023). Radiation Injury and Gut Microbiota-Based Treatment. Protein Cell.

[1167] Sharma A., Rath G.K., Chaudhary S.P., Thakar A., Mohanti B.K., Bahadur S. (2012). Lactobacillus Brevis CD2 Lozenges Reduce Radiation- and Chemotherapy-Induced Mucositis in Patients with Head and Neck Cancer: A Randomized Double-Blind Placebo-Controlled Study. Eur. J. Cancer.

[1168] DE Sanctis V., Belgioia L., Cante D., LA Porta M.R., Caspiani O., Guarnaccia R., Argenone A., Muto P., Musio D., DE Felice F. (2019). Lactobacillus Brevis CD2 for Prevention of Oral Mucositis in Patients with Head and Neck Tumors: A Multicentric Randomized Study. Anticancer Res..

[1169] Jiang C., Wang H., Xia C., Dong Q., Chen E., Qiu Y., Su Y., Xie H., Zeng L., Kuang J. (2019). A Randomized, Double-Blind, Placebo-Controlled Trial of Probiotics to Reduce the Severity of Oral Mucositis Induced by Chemoradiotherapy for Patients with Nasopharyngeal Carcinoma. Cancer.

[1170] Xia C., Jiang C., Li W., Wei J., Hong H., Li J., Feng L., Wei H., Xin H., Chen T. (2021). A Phase II Randomized Clinical Trial and Mechanistic Studies Using Improved Probiotics to Prevent Oral Mucositis Induced by Concurrent Radiotherapy and Chemotherapy in Nasopharyngeal Carcinoma. Front. Immunol..

[1171] Doppalapudi R., Vundavalli S., Prabhat M.P.V. (2020). Effect of Probiotic Bacteria on Oral Candida in Head- and Neck-Radiotherapy Patients: A Randomized Clinical Trial. J. Cancer Res. Ther..

[1172] Mirza M.A., Aruna D., Irukulla M. (2022). Efficacy of Bacillus Clausii UBBC-07 Spores in the Amelioration of Oral Mucositis in Head and Neck Cancer Patients Undergoing Radiation Therapy. Cancer Treat. Res. Commun..

[1173] Peng X., Li Z., Pei Y., Zheng S., Liu J., Wang J., Li R., Xu X. (2024). Streptococcus Salivarius K12 Alleviates Oral Mucositis in Patients Undergoing Radiotherapy for Malignant Head and Neck Tumors: A Randomized Controlled Trial. J. Clin. Oncol..

[1174] Li Z., He D., Zhang Y., Shi Z., Tang Q., Li Z., Peng X., Li D., Zhou D. (2025). Targeted Prevention of Radiation-Induced Oral Mucositis by Glutathione-Modified Liposome Coated K12 Probiotics and Clinical Study. Mater. Today Bio.

[1175] Goh C.E., Williams R., Gupta N., Ho F., Islam I., Lai C.W.M., Tan K.S., Hong C.H.L. (2025). Effect of Limosilactobacillus Reuteri Probiotic on Oral Mucositis in Patients Undergoing Head and Neck Radiation: A Randomised Trial. Oral Dis..

[1176] Urbancsek H., Kazar T., Mezes I., Neumann K. (2001). Results of a Double-Blind, Randomized Study to Evaluate the Efficacy and Safety of Antibiophilus® in Patients with Radiation-Induced Diarrhoea. Eur. J. Gastroenterol. Hepatol..

[1177] Delia P., Sansotta G., Donato V., Frosina P., Messina G., De Renzis C., Famularo G. (2007). Use of Probiotics for Prevention of Radiation-Induced Diarrhea. World J. Gastroenterol..

[1178] Giralt J., Regadera J.P., Verges R., Romero J., de la Fuente I., Biete A., Villoria J., Cobo J.M., Guarner F. (2008). Effects of Probiotic *Lactobacillus Casei* DN-114 001 in Prevention of Radiation-Induced Diarrhea: Results From Multicenter, Randomized, Placebo-Controlled Nutritional Trial. Int. J. Radiat. Oncol. Biol. Phys..

[1179] Chitapanarux I., Chitapanarux T., Traisathit P., Kudumpee S., Tharavichitkul E., Lorvidhaya V. (2010). Randomized Controlled Trial of Live Lactobacillus Acidophilus plus Bifidobacterium Bifidum in Prophylaxis of Diarrhea During Radiotherapy in Cervical Cancer Patients. Radiat. Oncol..

[1180] Demers M., Dagnault A., Desjardins J. (2014). A Randomized Double-Blind Controlled Trial: Impact of Probiotics on Diarrhea in Patients Treated with Pelvic Radiation. Clin. Nutr..

[1181] Linn Y.H., Thu K.K., Win N.H.H. (2019). Effect of Probiotics for the Prevention of Acute Radiation-Induced Diarrhoea Among Cervical Cancer Patients: A Randomized Double-Blind Placebo-Controlled Study. Probiotics Antimicrob. Proteins.

[1182] Ahrén I.L., Bjurberg M., Steineck G., Bergmark K., Jeppsson B. (2022). Decreasing the Adverse Effects in Pelvic Radiation Therapy: A Randomized Controlled Trial Evaluating the Use of Probiotics. Adv. Radiat. Oncol..

[1183] Du S.-X., Jia Y.-R., Ren S.-Q., Gong X.-J., Tang H., Wan-Shui W., Li-Ming S. (2018). The Protective Effects of Bacillus Licheniformis Preparation on Gastrointestinal Disorders and Inflammation Induced by Radiotherapy in Pediatric Patients with Central Nervous System Tumor. Adv. Med. Sci..

[1184] Murphy J., Stacey D., Crook J., Thompson B., Panetta D. (2000). Testing Control of Radiation-Induced Diarrhea with a Psyllium Bulking Agent: A Pilot Study. Can. Oncol. Nurs. J..

[1185] Itoh Y., Mizuno M., Ikeda M., Nakahara R., Kubota S., Ito J., Okada T., Kawamura M., Kikkawa F., Naganawa S. (2015). A Randomized, Double-Blind Pilot Trial of Hydrolyzed Rice Bran versus Placebo for Radioprotective Effect on Acute Gastroenteritis Secondary to Chemoradiotherapy in Patients with Cervical Cancer. Evid.-Based Complement. Altern. Med..

[1186] Sasidharan B.K., Ramadass B., Viswanathan P.N., Samuel P., Gowri M., Pugazhendhi S., Ramakrishna B.S. (2019). A Phase 2 Randomized Controlled Trial of Oral Resistant Starch Supplements in the Prevention of Acute Radiation Proctitis in Patients Treated for Cervical Cancer. J. Cancer Res. Ther..

[1187] Wedlake L., Shaw C., McNair H., Lalji A., Mohammed K., Klopper T., Allan L., Tait D., Hawkins M., Somaiah N. (2017). Randomized Controlled Trial of Dietary Fiber for the Prevention of Radiation-Induced Gastrointestinal Toxicity During Pelvic Radiotherapy. Am. J. Clin. Nutr..

[1188] Rosli D., Shahar S., Manaf Z.A., Lau H.J., Yusof N.Y.M., Haron M.R., Majid H.A. (2021). Randomized Controlled Trial on the Effect of Partially Hydrolyzed Guar Gum Supplementation on Diarrhea Frequency and Gut Microbiome Count Among Pelvic Radiation Patients. J. Parenter. Enter. Nutr..

[1189] Forslund M., Ottenblad A., Ginman C., Johansson S., Nygren P., Johansson B. (2020). Effects of a Nutrition Intervention on Acute and Late Bowel Symptoms and Health-Related Quality of Life up to 24 Months Post Radiotherapy in Patients with Prostate Cancer: A Multicentre Randomised Controlled Trial. Support. Care Cancer.

[1190] García-Peris P., Velasco C., Lozano M.A., Moreno Y., Paron L., de la Cuerda C., Bretón I., Camblor M., García-Hernández J., Guarner F. (2012). Effect of a Mixture of Inulin and Fructo-Oligosaccharide on Lactobacillus and Bifidobacterium Intestinal Microbiota of Patients Receiving Radiotherapy: A Randomised, Double-Blind, Placebo-Controlled Trial. Nutr. Hosp..

[1191] Yoshifuji K., Inamoto K., Kiridoshi Y., Takeshita K., Sasajima S., Shiraishi Y., Yamashita Y., Nisaka Y., Ogura Y., Takeuchi R. (2020). Prebiotics Protect Against Acute Graft-versus-Host Disease and Preserve the Gut Microbiota in Stem Cell Transplantation. Blood Adv..

[1192] Salminen E., Elomaa I., Minkkinen J., Vapaatalo H., Salminen S. (1988). Preservation of Intestinal Integrity During Radiotherapy Using Live Lactobacillus Acidophilus Cultures. Clin. Radiol..

[1193] Nascimento M., Caporossi C., Eduardo Aguilar-Nascimento J., Michelon Castro-Barcellos H., Teixeira Motta R., Reis Lima S. (2020). Efficacy of Synbiotics to Reduce Symptoms and Rectal Inflammatory Response in Acute Radiation Proctitis: A Randomized, Double-Blind, Placebo-Controlled Pilot Trial. Nutr. Cancer.

[1194] Scartoni D., Desideri I., Giacomelli I., Cataldo V.D., Brina L.D., Mancuso A., Furfaro I., Bonomo P., Simontacchi G., Livi L. (2015). Nutritional Supplement Based on Zinc, Prebiotics, Probiotics and Vitamins to Prevent Radiation-Related Gastrointestinal Disorders. Anticancer Res..

[1195] Farshi Radvar F., Mohammad-Zadeh M., Mahdavi R., Andersen V., Nasirimotlagh B., Faramarzi E., Lotfi Yagin N. (2020). Effect of Synbiotic Supplementation on Matrix Metalloproteinase Enzymes, Quality of Life and Dietary Intake and Weight Changes in Rectal Cancer Patients Undergoing Neoadjuvant Chemoradiotherapy. Mediterr. J. Nutr. Metab..

[1196] Manifar S., Koopaie M., Jahromi Z.M., Kolahdooz S. (2022). Effect of Synbiotic Mouthwash on Oral Mucositis Induced by Radiotherapy in Oral Cancer Patients: A Double-Blind Randomized Clinical Trial. Support. Care Cancer.

[1197] Mortazavi S.M.J., Nowroozi S., Haghani M., Zarrini-Monfared Z., Gheisari F., Sihver L. (2023). Probiotic Bacteria Cannot Mitigate the Adverse Effects of Radioactive Iodine-131 Treatment. Cancers.

[1198] Koopaie M., Vaziri S., Manifar S., Younespour S., Kolahdooz S. (2025). Efficacy of Synbiotic Mouthwash on Salivary TLR2 Levels and Oral Mucositis in Head and Neck Cancer Patients Undergoing Radiotherapy: A Randomized Clinical Trial. Support. Care Cancer.

[1199] Stene C., Xu J., Andrade S.F.d., Palmquist I., Molin G., Ahrné S., Thorlacius H., Johnson L.B., Jeppsson B. (2025). Synbiotics Protected Radiation-Induced Tissue Damage in Rectal Cancer Patients: A Controlled Trial. Clin. Nutr..

[1200] Wang Z., Wang Q., Wang X., Zhu L., Chen J., Zhang B., Chen Y., Yuan Z. (2019). Gut Microbial Dysbiosis Is Associated with Development and Progression of Radiation Enteritis During Pelvic Radiotherapy. J. Cell. Mol. Med..

[1201] Liu J., Liu C., Yue J. (2021). Radiotherapy and the Gut Microbiome: Facts and Fiction. Radiat. Oncol..

[1202] Liu Q., Nobaek S., Adawi D., Mao Y., Wang M., Molin G., Ekelund M., Jeppsson B. (2001). Administration of Lactobacillus Plantarum 299v Reduces Side-Effects of External Radiation on Colon Anastomotic Healing in an Experimental Model. Color. Dis..

[1203] Demirer S., Ulusu N.N., Aslim B., Kepenekci I., Ulusoy C., Andrieu M.N., Erkek B., Aydintug S. (2007). Protective Effects of *Lactobacillus Delbrueckii* Subsp *Bulgaricus* B3 on Intestinal Enzyme Activities After Abdominal Irradiation in Rats. Nutr. Res..

[1204] Espinal A., Epperly M.W., Mukherjee A., Fisher R., Shields D., Wang H., Huq M.S., Hamade D.F., Vlad A.M., Coffman L. (2022). Intestinal Radiation Protection and Mitigation by Second-Generation Probiotic Lactobacillus-Reuteri Engineered to Deliver Interleukin-22. Int. J. Mol. Sci..

[1205] Jian Y.-P., Yang G., Zhang L.-H., Liang J.-Y., Zhou H.-L., Wang Y.-S., Xu Z.-X. (2022). Lactobacillus Plantarum Alleviates Irradiation-Induced Intestinal Injury by Activation of FXR-FGF15 Signaling in Intestinal Epithelia. J. Cell Physiol..

[1206] Riehl T.E., Alvarado D., Ee X., Zuckerman A., Foster L., Kapoor V., Thotala D., Ciorba M.A., Stenson W.F. (2019). Lactobacillus Rhamnosus GG Protects the Intestinal Epithelium from Radiation Injury Through Release of Lipoteichoic Acid, Macrophage Activation, and the Migration of Mesenchymal Stem Cells. Gut.

[1207] Wang B., Jin Y., Dong J., Xiao H., Zhang S., Li Y., Chen Z., Yang X., Fan S., Cui M. (2021). Low-Intensity Exercise Modulates Gut Microbiota to Fight Against Radiation-Induced Gut Toxicity in Mouse Models. Front. Cell Dev. Biol..

[1208] He K.-Y., Lei X.-Y., Wu D.-H., Zhang L., Li J.-Q., Li Q.-T., Yin W.-T., Zhao Z.-L., Liu H., Xiang X.-Y. (2023). Akkermansia Muciniphila Protects the Intestine from Irradiation-Induced Injury by Secretion of Propionic Acid. Gut Microbes.

[1209] Rodríguez-Daza M.C., de Vos W.M. (2023). Polyphenols as Drivers of a Homeostatic Gut Microecology and Immuno-Metabolic Traits of Akkermansia Muciniphila: From Mouse to Man. Int. J. Mol. Sci..

[1210] Zhang X., Cui Q., Yin L., Zhu J., Mao Y., Yin R., Shao H., Wang W., Sun X., Zhang Z. (2025). Ginger-Derived Vesicle-like Nanoparticles Loaded with Curcumin to Alleviate Ionizing Radiation-Induced Intestinal Damage via Gut Microbiota Regulation. Gut Microbes.

[1211] Devaraj N.K., Suppiah S., Veettil S.K., Ching S.M., Lee K.W., Menon R.K., Soo M.J., Deuraseh I., Hoo F.K., Sivaratnam D. (2019). The Effects of Probiotic Supplementation on the Incidence of Diarrhea in Cancer Patients Receiving Radiation Therapy: A Systematic Review with Meta-Analysis and Trial Sequential Analysis of Randomized Controlled Trials. Nutrients.

[1212] Venkidesh B.S., Shankar S.R., Narasimhamurthy R.K., Rao S.B.S., Mumbrekar K.D. (2023). Radioprotective Potential of Probiotics Against Gastrointestinal and Neuronal Toxicity: A Preclinical Study. Clin. Transl. Oncol..

[1213] Zhang X., Fisher R., Hou W., Shields D., Epperly M.W., Wang H., Wei L., Leibowitz B.J., Yu J., Alexander L.M. (2020). Second-Generation Probiotics Producing IL-22 Increase Survival of Mice After Total Body Irradiation. In Vivo.

[1214] Hamade D.F., Espinal A., Yu J., Leibowitz B.J., Fisher R., Hou W., Shields D., van Pijkeren J.-P., Mukherjee A., Epperly M.W. (2022). Lactobacillus Reuteri Releasing IL-22 (LR-IL-22) Facilitates Intestinal Radioprotection for Whole-Abdomen Irradiation (WAI) of Ovarian Cancer. Radiat. Res..

[1215] Minervini G., Franco R., Marrapodi M.M., Fiorillo L., Badnjević A., Cervino G., Cicciù M. (2023). Probiotics in the Treatment of Radiotherapy-Induced Oral Mucositis: Systematic Review with Meta-Analysis. Pharmaceuticals.

[1216] Wang Y., Li J., Zhang H., Zheng X., Wang J., Jia X., Peng X., Xie Q., Zou J., Zheng L. (2021). Probiotic Streptococcus Salivarius K12 Alleviates Radiation-Induced Oral Mucositis in Mice. Front. Immunol..

[1217] Yang J., Ding C., Dai X., Lv T., Xie T., Zhang T., Gao W., Gong J., Zhu W., Li N. (2017). Soluble Dietary Fiber Ameliorates Radiation-Induced Intestinal Epithelial-to-Mesenchymal Transition and Fibrosis. J. Parenter. Enter. Nutr..

[1218] Eaton S.E., Kaczmarek J., Mahmood D., McDiarmid A.M., Norarfan A.N., Scott E.G., Then C.K., Tsui H.Y., Kiltie A.E. (2022). Exploiting Dietary Fibre and the Gut Microbiota in Pelvic Radiotherapy Patients. Br. J. Cancer.

[1219] Liu D., Zhuang B., Wei M., Yuan T., Li J., Deng P., Du L., Yuan B., Jin Y. (2023). Oral Konjac Glucomannan for Prevention of Ionizing Radiation-Induced Injury by Regulating Gut Microbiota and Increasing Short Chain Fatty Acids. Int. J. Biol. Macromol..

[1220] Sanguri S., Gupta D. (2021). Prebiotic Mannan Oligosaccharide Pretreatment Improves Mice Survival Against Lethal Effects of Gamma Radiation by Protecting GI Tract and Hematopoietic Systems. Front. Oncol..

[1221] Sureban S.M., May R., Qu D., Chandrakesan P., Weygant N., Ali N., Lightfoot S.A., Ding K., Umar S., Schlosser M.J. (2015). Dietary Pectin Increases Intestinal Crypt Stem Cell Survival Following Radiation Injury. PLoS ONE.

[1222] Then C.K., Paillas S., Moomin A., Misheva M.D., Moir R.A., Hay S.M., Bremner D., Roberts (nee Nellany) K.S., Smith E.E., Heidari Z. (2024). Dietary Fibre Supplementation Enhances Radiotherapy Tumour Control and Alleviates Intestinal Radiation Toxicity. Microbiome.

[1223] Iyama S., Sato T., Tatsumi H., Hashimoto A., Tatekoshi A., Kamihara Y., Horiguchi H., Ibata S., Ono K., Murase K. (2014). Efficacy of Enteral Supplementation Enriched with Glutamine, Fiber, and Oligosaccharide on Mucosal Injury Following Hematopoietic Stem Cell Transplantation. Case Rep. Oncol..

[1224] Akmansu M., Dincer S., Saglam E.K., Akgun Z. (2023). Probiotic-Prebiotic Usage Effects on Acute Inflammation Parameters Receiving Pelvic Radiotherapy Patients: A Prospective Multicentric Study. Clin. Nutr. ESPEN.

[1225] Cui M., Xiao H., Li Y., Zhou L., Zhao S., Luo D., Zheng Q., Dong J., Zhao Y., Zhang X. (2017). Faecal Microbiota Transplantation Protects Against Radiation-Induced Toxicity. EMBO Mol. Med..

[1226] Guo H., Chou W.-C., Lai Y., Liang K., Tam J.W., Brickey W.J., Chen L., Montgomery N.D., Li X., Bohannon L.M. (2020). Multi-Omics Analyses of Radiation Survivors Identify Radioprotective Microbes and Metabolites. Science.

[1227] Xiao H.-W., Cui M., Li Y., Dong J.-L., Zhang S.-Q., Zhu C.-C., Jiang M., Zhu T., Wang B., Wang H.-C. (2020). Gut Microbiota-Derived Indole 3-Propionic Acid Protects Against Radiation Toxicity via Retaining Acyl-CoA-Binding Protein. Microbiome.

[1228] Ding X., Li Q., Li P., Chen X., Xiang L., Bi L., Zhu J., Huang X., Cui B., Zhang F. (2020). Fecal Microbiota Transplantation: A Promising Treatment for Radiation Enteritis?. Radiother. Oncol..

[1229] Liu T., Su D., Lei C., Liu Z. (2023). Treatment of Radiation Enteritis with Fecal Transplantation. Am. Surg..

[1230] Cui J.Q., Tian H.L., Wang X.J., Wang L., Liu Y.K., Ye C., Ding L.F., Li N., Chen Q.Y. (2023). Analysis of short-term efficacy of perioperative fecal microbiota transplantation combined with nutritional support in patients with radiation-induced enteritis complicated by intestinal obstruction. Zhonghua Wei Chang Wai Ke Za Zhi.

[1231] Menêses A.G.d., Reis P.E.D.D., Guerra E.N.S., Canto G.D.L., Ferreira E.B. (2018). Use of trolamine to prevent and treat acute radiation dermatitis: A systematic review and meta-analysis. Rev. Lat. Am. Enferm..

[1232] Lee C.-J., Fang H.-F., Wang C.-Y., Chou K.-R., Huang T.-W. (2022). Effect of Hyaluronic Acid on Radiodermatitis in Patients with Breast Cancer: A Meta-Analysis of Randomized Controlled Trials. Support. Care Cancer.

[1233] Wang T., Liao J., Zheng L., Zhou Y., Jin Q., Wu Y. (2022). Aloe Vera for Prevention of Radiation-Induced Dermatitis: A Systematic Review and Cumulative Analysis of Randomized Controlled Trials. Front. Pharmacol..

[1234] Behroozian T., Goldshtein D., Wolf J.R., van den Hurk C., Finkelstein S., Lam H., Patel P., Kanee L., Lee S.F., Chan A.W. (2023). MASCC Clinical Practice Guidelines for the Prevention and Management of Acute Radiation Dermatitis: Part 1) Systematic Review. eClinicalMedicine.

[1235] Cao H., Li W., Cai H. (2024). The Effect of Various Interventions on the Prevention of Radiation Dermatitis: A Network Meta-Analysis. Am. J. Transl. Res..

[1236] Kuszaj O., Day M., Tse S.S.W., Lee S.F., Wang A.J., Bayrakdarian S., Vesprini D., Corbin K., Karam I., Choi J.I. (2025). A Critical Review of Randomized Controlled Trials on Topical Corticosteroids for the Prevention of Radiation Dermatitis in Breast Cancer. Support. Care Cancer.

[1237] Deantonio L., Borgonovo G., Caverzasio S., Piliero M.A., Canino P., Puliatti A., Zilli T., Valli M.C., Richetti A. (2025). Hyaluronic Acid 0.2% Cream for Preventing Radiation Dermatitis in Breast Cancer Patients Treated with Postoperative Radiotherapy: A Randomized, Double-Blind, Placebo-Controlled Study. Breast.

[1238] Goyal P.K., Gehlot P. (2009). Radioprotective Effects of Aloe Vera Leaf Extract on Swiss Albino Mice Against Whole-Body Gamma Irradiation. J. Environ. Pathol. Toxicol. Oncol..

[1239] Bala S., Chugh N.A., Bansal S.C., Garg M.L., Koul A. (2018). Radiomodulatory Effects of *Aloe Vera* on Hepatic and Renal Tissues of X-Ray Irradiated Mice. Mutat. Res. Fundam. Mol. Mech. Mutagen..

[1240] Cui Z., Xin M., Yin H., Zhang J., Han F. (2015). Topical Use of Olive Oil Preparation to Prevent Radiodermatitis: Results of a Prospective Study in Nasopharyngeal Carcinoma Patients. Int. J. Clin. Exp. Med..

[1241] Chitapanarux I., Tovanabutra N., Chiewchanvit S., Sripan P., Chumachote A., Nobnop W., Tippanya D., Khamchompoo D. (2019). Emulsion of Olive Oil and Calcium Hydroxide for the Prevention of Radiation Dermatitis in Hypofractionation Post-Mastectomy Radiotherapy: A Randomized Controlled Trial. Breast Care.

[1242] Herst P.M., Bennett N.C., Sutherland A.E., Peszynski R.I., Paterson D.B., Jasperse M.L. (2014). Prophylactic Use of Mepitel Film Prevents Radiation-Induced Moist Desquamation in an Intra-Patient Randomised Controlled Clinical Trial of 78 Breast Cancer Patients. Radiother. Oncol..

[1243] Behroozian T., Milton L., Karam I., Zhang L., Ding K., Lou J., Gallant F., Rakovitch E., Tran W., Soliman H. (2023). Mepitel Film for the Prevention of Acute Radiation Dermatitis in Breast Cancer: A Randomized Multicenter Open-Label Phase III Trial. J. Clin. Oncol..

[1244] Narvaez C., Doemer C., Idel C., Setter C., Olbrich D., Ujmajuridze Z., Carl J.H., Rades D. (2018). Radiotherapy Related Skin Toxicity (RAREST-01): Mepitel® Film versus Standard Care in Patients with Locally Advanced Head-and-Neck Cancer. BMC Cancer.

[1245] Yan J., Yuan L., Wang J., Li S., Yao M., Wang K., Herst P.M. (2020). Mepitel Film Is Superior to Biafine Cream in Managing Acute Radiation-Induced Skin Reactions in Head and Neck Cancer Patients: A Randomised Intra-Patient Controlled Clinical Trial. J. Med. Radiat. Sci..

[1246] Kazmierska-Grebowska P., Jankowski M.M., Obrador E., Kolodziejczyk-Czepas J., Litwinienko G., Grebowski J. (2025). Nanotechnology Meets Radiobiology: Fullerenols and Metallofullerenols as Nano-Shields in Radiotherapy. Biomed. Pharmacother..

[1247] Zhao M., Wang C., Xie J., Ji C., Gu Z. (2021). Eco-Friendly and Scalable Synthesis of Fullerenols with High Free Radical Scavenging Ability for Skin Radioprotection. Small.

[1248] Peng H., Jiang H., Li Y., Wang X., Ma D., Guo J., Li B., Wang H., Liao Y., Gu C. (2024). A Transdermal Fullerenol Emulsion-Mediated Angiogenesis Mitigates Radiation-Induced Skin Injury. Chem. Eng. J..

[1249] Wang C., Zhao M., Xie J., Wang H., Gu Z., Sun F. (2023). Colon-Targeted Release of Gel Microspheres Loaded with Antioxidative Fullerenol for Relieving Radiation-Induced Colon Injury and Regulating Intestinal Flora. Adv. Healthc. Mater..

[1250] Zhao M., Wang C., Ji C., Liu R., Xie J., Wang Y., Gu Z. (2023). Ascidian-Inspired Temperature-Switchable Hydrogels with Antioxidant Fullerenols for Protecting Radiation-Induced Oral Mucositis and Maintaining the Homeostasis of Oral Microbiota. Small.

[1251] Yin H., Gao Y., Chen W., Tang C., Zhu Z., Li K., Xia S., Han C., Ding X., Ruan F. (2023). Topically Applied Fullerenols Protect Against Radiation Dermatitis by Scavenging Reactive Oxygen Species. Discov. Nano.

[1252] Wang Q., Shi X., Guo J., Gu Z., Dong X., Qin Y. (2025). Topical EOSSKY Fullerene Moisturizing and Repairing Cream for Preventing Acute Radiation Dermatitis in Breast Cancer Patients Undergoing Radiotherapy: A Randomized Controlled Trial. Front. Med..

[1253] Wang X., Li Y., Zhuang W. (2025). Safety Analysis of Romiplostim, Eltrombopag, and Avatrombopag Post-Market Approval: A Pharmacovigilance Study Based on the FDA Adverse Event Reporting System. BMC Pharmacol. Toxicol..

[1254] Akbari S., Kariznavi E., Jannati M., Elyasi S., Tayarani-Najaran Z. (2020). Curcumin as a Preventive or Therapeutic Measure for Chemotherapy and Radiotherapy Induced Adverse Reaction: A Comprehensive Review. Food Chem. Toxicol..

[1255] Jackson I.L., Zodda A., Gurung G., Pavlovic R., Kaytor M.D., Kuskowski M.A., Vujaskovic Z. (2017). BIO 300, a Nanosuspension of Genistein, Mitigates Pneumonitis/Fibrosis Following High-dose Radiation Exposure in the C57L/J Murine Model. Br. J. Pharmacol..

[1256] Faramarzi S., Piccolella S., Manti L., Pacifico S. (2021). Could Polyphenols Really Be a Good Radioprotective Strategy?. Molecules.

[1257] Farhood B., Mortezaee K., Motevaseli E., Mirtavoos-Mahyari H., Shabeeb D., Eleojo Musa A., Sanikhani N.S., Najafi M., Ahmadi A. (2019). Selenium as an Adjuvant for Modification of Radiation Response. J. Cell. Biochem..

[1258] Hewlings S., Kalman D. (2020). A Review of Zinc-L-Carnosine and Its Positive Effects on Oral Mucositis, Taste Disorders, and Gastrointestinal Disorders. Nutrients.

[1259] Fish B.L., MacVittie T.J., Gao F., Narayanan J., Gasperetti T., Scholler D., Sheinin Y., Himburg H.A., Hart B., Medhora M. (2021). Rat Models of Partial-Body Irradiation with Bone Marrow-Sparing (Leg-out PBI) Designed for FDA Approval of Countermeasures for Mitigation of Acute and Delayed Injuries by Radiation. Health Phys..

[1260] Orschell C.M., Wu T., Patterson A.M. (2022). Impact of Age, Sex, and Genetic Diversity in Murine Models of the Hematopoietic Acute Radiation Syndrome (H-ARS) and the Delayed Effects of Acute Radiation Exposure (DEARE). Curr. Stem Cell Rep..

[1261] Patterson A.M., Vemula S., Plett P.A., Sampson C.H., Chua H.L., Fisher A., Wu T., Sellamuthu R., Feng H., Katz B.P. (2022). Age and Sex Divergence in Hematopoietic Radiosensitivity in Aged Mouse Models of the Hematopoietic Acute Radiation Syndrome. Radiat. Res..

[1262] Andruska N., Schlaak R.A., Frei A., Schottstaedt A.M., Lin C.-Y., Fish B.L., Gasperetti T., Mpoy C., Pipke J.L., Pedersen L.N. (2023). Differences in Radiation-Induced Heart Dysfunction in Male versus Female Rats. Int. J. Radiat. Biol..

[1263] Singh V.K., Carpenter A.D., Janocha B.L., Petrus S.A., Fatanmi O.O., Wise S.Y., Seed T.M. (2023). Radiosensitivity of Rhesus Nonhuman Primates: Consideration of Sex, Supportive Care, Body Weight, and Age at Time of Exposure. Expert Opin. Drug Discov..

